# Genome-wide analysis of *Aux/IAA *and *ARF *gene families in *Populus trichocarpa*

**DOI:** 10.1186/1471-2229-7-59

**Published:** 2007-11-06

**Authors:** Udaya C Kalluri, Stephen P DiFazio, Amy M Brunner, Gerald A Tuskan

**Affiliations:** 1 Environmental Sciences Division, Oak Ridge National Laboratory, PO Box 2008, Oak Ridge, TN 37831, USA; 2 Department of Biology, West Virginia University, PO Box 6057, Morgantown, WV 26506, USA; 3 Department of Forestry, Virginia Polytechnic Institute and State University, 448 Latham Hall, Blacksburg, VA 24061, USA

## Abstract

**Background:**

Auxin/Indole-3-Acetic Acid (Aux/IAA) and Auxin Response Factor (ARF) transcription factors are key regulators of auxin responses in plants. We identified the suites of genes in the two gene families in *Populus *and performed comparative genomic analysis with *Arabidopsis *and rice.

**Results:**

A total of 35 *Aux/IAA *and 39 *ARF *genes were identified in the *Populus *genome. Comparative phylogenetic analysis revealed that several Aux/IAA and ARF subgroups have differentially expanded or contracted between the two dicotyledonous plants. Activator *ARF *genes were found to be two fold-overrepresented in the *Populus *genome. *PoptrIAA *and *PoptrARF *gene families appear to have expanded due to high segmental and low tandem duplication events. Furthermore, expression studies showed that genes in the expanded *PoptrIAA3 *subgroup display differential expression.

**Conclusion:**

The present study examines the extent of conservation and divergence in the structure and evolution of *Populus Aux/IAA *and *ARF *gene families with respect to *Arabidopsis *and rice. The gene-family analysis reported here will be useful in conducting future functional genomics studies to understand how the molecular roles of these large gene families translate into a diversity of biologically meaningful auxin effects.

## Background

*Aux/IAA*s are auxin response genes that code for nuclear localized proteins [1]. Aux/IAA proteins generally have four characteristic domains; an N-terminal repression domain, an adjacent domain involved in protein stability, and two C-terminal domains (CTD), III and IV, through which Aux/IAA proteins form homo- and heterodimers with Aux/IAA or ARF proteins [2]. Most ARF proteins contain an N-terminal B3-like DNA binding domain that includes an ARF family-specific domain, a variable middle region that confers activator or repressor activity, and domains III and IV that are also found in Aux/IAA [3]. ARF proteins are capable, irrespective of auxin status, of binding to auxin responsive cis-elements (AuxRE; TGTCTC) present upstream to the coding sequence of auxin responsive genes [4,5]. Aux/IAA proteins bind to the DNA-bound ARF partner proteins via domains III and IV and repress ARF activity. In the auxin activated status, Aux/IAA proteins are ubiquitinated via interactions with the auxin-modified SCF^TIR1^complex and subsequently degraded by 26S proteasome action [6-8]. While some ARFs possess a characteristic glutamine (Q)-rich middle region which confers an activator activity, ARFs with a proline, serine and threonine-rich middle region are found to be associated with repressor activity [3,9]. The complexity of auxin regulatory activity is due in part to the large sizes of the *ARF *and *Aux/IAA *gene families, as well as variations in activation or repression activity among ARFs, heterodimerization affinities, expression patterns, and auxin-mediated transcriptional and posttranscriptional regulation.

Despite the knowledge that Aux/IAAs and ARFs influence apical dominance, vascular development, tropic movements, root growth, tissue and organ patterning, and flower and fruit development [10], many questions still linger. For example, these gene families remain largely uncharacterized in forest tree species and the degree of conservation of gene families between annual and perennial woody plants is unknown. Furthermore, the mechanisms of Aux/IAA and ARF interaction and regulation are not completely understood and much remains to be learned about their roles in the contexts of a cell and the whole organism. Identification of *Aux/IAA *and *ARF *gene families from distinct model plants is a necessary step in formulating better hypotheses related to physiological and developmental processes. The recent sequencing of the *Populus *genome has provided an additional reference genome for testing inferences on auxin signal transduction events obtained previously through functional genomic studies of *Arabidopsis*.

The present paper summarizes findings from bioinformatics-based comparative genomic studies to identify the total number of *Aux/IAA *and *ARF *genes in *Populus*, to predict the protein domain architectures, and to assess the extent of conservation and divergence between *Populus*, *Arabidopsis *and rice gene families. Targeted RT-PCR and whole genome microarray analyses, and EST database surveys were also undertaken to explore differential expression of closely grouping co-orthologs. Furthermore, we have reflected on the possible implications of differential patterns of retention, loss, and expansion of duplicated homeologous genes.

## Results and discussion

### Identification and sequence analysis of *Populus Aux/IAA *genes

**Figure 1:**
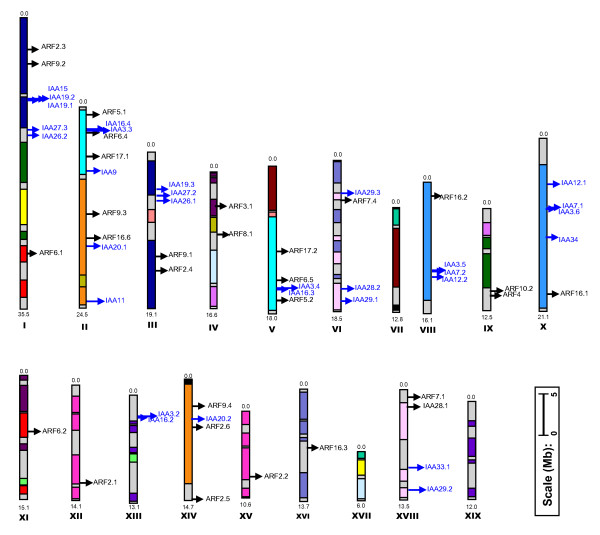
**Chromosomal positions of *PoptrIAA *and *PoptrARF *genes**. Scale represents a 5 Mb chromosomal distance. Colors indicate the chimeric nature of most linkage groups. Common colors refer to homeologous genome blocks, presumed to have arisen from the salicoid genome duplication 65 Mya and shared by two chromosomes [13]. Chromosome numbers (linkage group number I-XIX) and sizes (Mb) are indicated at the bottom end of each chromosome. *PoptrIAA *and *PoptrARF *genes are represented in blue and black font colors, respectively. 4 *PoptrIAA *and 11 *PoptrARF *genes reside on unassembled scaffolds (See Additional file 1).

The *Populus *genome has 35 predicted *Aux/IAA *genes (henceforth referred to as *PoptrIAA*) compared to 29 genes in the *Arabidopsis *genome (henceforth referred to as *AtIAA*) [11] and to 31 genes in the rice genome (henceforth referred to as *OsIAA*) [12]. *In silico *mapping of the gene loci showed that *PoptrIAA *genes are present on 10 of the 19 chromosomes (Figure 1; See Additional file 1).

Any analysis of *Populus *gene family evolution must take into account the most significant event in the recent evolution of the genus; a genome-wide duplication event that occurred approximately 65 Mya and which is still detectable over approximately 92% of the genome [13]. Based on the age estimates of duplicate genes and homology-microsynteny analysis (See Additional file 2), 14 *PoptrIAA *genes pairs (80% of total genes) are represented within segmental duplication regions associated with the recent salicoid duplication event. It is intriguing that 6 pairs of tandem duplicates are represented as 'ancient tandem genes' within more recent whole genome or segmental duplicate clusters. These 15 *PoptrIAA *genes (~43% of total) are represented in 7 distinct tandem duplicate gene clusters, with 6 clusters containing two tandem genes and 1 cluster containing 3 tandem genes (*PoptrIAA15*, *PoptrIAA19.1 *and *PoptrIAA19.2*).

It is possible that some of the apparently closely-related genes are in fact alleles from unassembled haplotypes, which are potential artifacts from shotgun assembly of this highly heterozygous genome. For example, *PoptrIAA3.1 *and *PoptrIAA3.2 *group closely with their tandem duplicates, *PoptrIAA16.1 *and *PoptrIAA16*.2, and *PoptrIAA3.1 *and *PoptrIAA16.1 *genes are located on a presently unassembled scaffold (scaffold_70). However, the apparent co-orthologs are divergent at the amino acid level as well as in the flanking gene order and identity in the syntenic blocks, which argue against the classification of the scaffold as a haplotype.

Further analyses of conserved domains and multiple sequence alignments of predicted proteins showed that some *Populus *genes contain modifications in the conserved domain architecture (See Additional files 3, 4 and 5). Based on MEME-MAST protein motif analysis, domain I was found to be missing in PoptrIAA29.1, PoptrIAA29.2 and PoptrIAA29.3 and also in the *Arabidopsis *ortholog, AtIAA29. Similarly, domains I and II appear to be missing in PoptrIAA33.1, PoptrIAA33.2 and PoptrIAA34 as well as AtIAA33 and AtIAA34. Multiple sequence alignment shows that the "idLgLsLrt" sequence reported as domain I motif in AtIAA34 (LxLxL) [14] is conserved with AtIAA14, AtIAA15, AtIAA16 and AtIAA17 (See Additional file 3) in which the consensus sequence of the reported domain I is "TELxLxLPG" [14].

Domain II enables the SCF^TIR1^-dependent proteasome-mediated degradation of Aux/IAA [15]. Rapid basal degradation rate and auxin responsiveness of Aux/IAA proteins are found to be associated with alterations in the highly conserved domain II motif, 'VGWPPI/V' [15] and the 'KR' motif between domains I and II [16]. Studies in *Arabidopsis *show that mutations in the VGWPPV motif render the repressor protein stable [17-23]. Multiple sequence alignments of full-length amino acid sequences revealed deviations in both the 'VGWPPI/V' and 'KR' motif sequences in PoptrIAA7.1, PoptrIAA20.1, PoptrIAA 20.2, PoptrIAA 33.1, PoptrIAA 33.2, PoptrIAA 34 and AtIAA20, AtIAA30, AtIAA31, AtIAA32, AtIAA33 and AtIAA34. Deviations in the conserved 'KR' motif alone were observed in PoptrIAA27.1, PoptrIAA26.2 and AtIAA28 sequences. However, PoptrIAA7.1 was found to be unique in that it contains a tandem duplication of the domain II region (See Additional file 5). This unique feature has not been detected in *Aux/IAA *genes reported from any other genera including *Arabidopsis*, *Oryza*, *Vitis*, *Nicotiana *and *Coffea*, but was found in GenBank ESTs derived from *P*. *trichocarpa*, *P*. *deltoides*, *P*. *tremuloides*, *P. tremula *× *tremuloides *[24], *P*. *alba *× *tremula*, *P*. *trichocarpa *× *deltoides *and *P*. *fremontii *× *angustifolia*, suggesting that this is a *Populus*-specific acquisition.

### Comparative analysis of *Populus *and *Arabidopsis Aux/IAA *gene families

**Figure 2:**
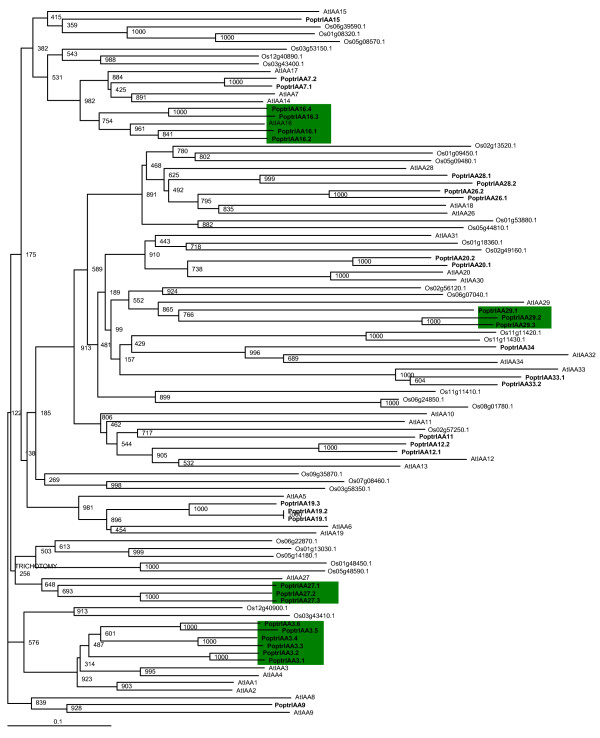
**Phylogenetic analysis of predicted full-length Aux/IAA protein sequences using Neighbor-Joining method**. Amino acid sequences of full-length predicted proteins were aligned using MUSCLE program. Tree was produced as described in methods. Bootstrap support is indicated at each node. Green boxes represent sustained expansion of subgroups in *Populus*.

Phylogenetic reconstruction at the molecular level using all available and predicted *Populus*, *Arabidopsis *and rice Aux/IAA amino acid sequences shows that four groups of *PoptrIAA*s (*PoptrIAA3*, *16*, *27 *and *29*) have expanded to contain three or more members each (Figure 2). Three *AtIAA*s did not have a representative sequence ortholog in *Populus*, indicating acquisition or perpetuation of distinct *Aux/IAA*s unique to *Arabidopsis *and its relatively recent ancestors.

There are four homologs of *AtIAA16*, *PoptrIAA16.1–16.4*, predicted in the *Populus *genome. Phylogenetic groupings suggest absence of *AtIAA16 *orthologs from rice. *PoptrIAA16.3 *and *PoptrIAA16.4 *are likely co-orthologs of an ancestral gene lost in Arabidopsis. These co-orthologs have intact conserved domains and EST evidence supporting their functionality (See Additional files 1 and 5). Based on EST data, *PoptrIAA16.1 *appears to express during wood formation (in cambial zone and tension wood), whereas *PoptrIAA16.3 *and *16.4 *appear to express in young leaves (See Additional file 6). A *PoptrIAA16.1-*like EST was previously reported from hybrid aspen to be expressed in the context of wood formation and a *PoptrIAA16.3*-like EST was shown to be expressed in dividing and expanding cells [24]. These findings are partially supported by our whole genome microarray data, which showed that *PoptrIAA16.1 *was expressed in xylem, phloem, cortex, root and seed (See Additional files 7, 8 and 9).

However, there was no array evidence to support expression of *PoptrIAA16.3 *and *PoptrIAA16.4 *in young leaf tissues, though *PoptrIAA16.3 *was expressed at the stem apex, in apical vegetative buds, newly set reproductive buds of both genders, and in older leaves.

Two Aux/IAA proteins, IAA26/PAP1 (Phytochrome associated protein1) and IAA27 are known to interact with phytochrome A (PHYA) [25,26] and TMV replicase [1,27,28]. *Populus *has three IAA27-like *Aux/IAA *genes. EST and microarray data suggest expression in shoot meristem, floral buds and dormant and active cambia (See Additional files 6, 7, 8 and 9). Possible affinity for interaction with PHYA protein suggests a role for PoptrIAA27 in external-stimuli-dependent cambium activation or growth status. *PoptrIAA26.1 *and *PoptrIAA26.2 *genes cluster closely with *AtIAA18 *and *AtIAA26 *and are expressed in shoot meristem, young and senescing leaves, male catkins, and floral buds. RT-PCR survey of *PoptrIAA26.1 *and *PoptrIAA26.2 *genes shows highest expression in young leaves. Since AtIAA26 and AtIAA27 proteins display binding affinity towards PHYA, it is likely that these *Populus *sequence orthologs are also involved in mediating the photoregulation of various tree developmental processes.

**Figure 3:**
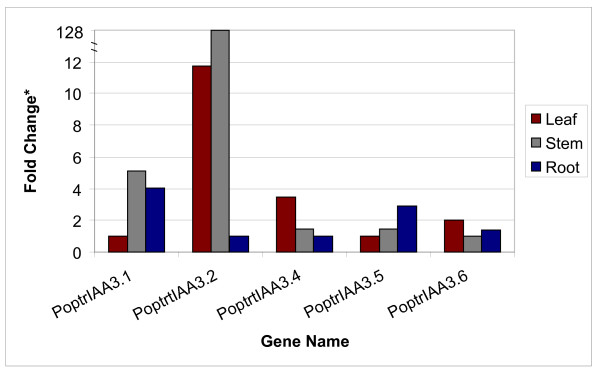
**Expression analysis of *PoptrIAA3 *subgroup genes using real-time RT- PCR**. Fold Change (*) is represented relative to lowest value observed for the gene. Lowest value was determined by comparison of relative threshold cycle values for a specific gene across leaf, stem and root samples. Fold change was calculated by the formula 2^-ΔΔCt^, where ΔΔCt is the difference between ΔCt of a gene is a given tissue and the lowest value ΔCt observed for that gene in any of the three tissue types. ΔCt was estimated by the formula; (Ct of gene of interest) – (geometric mean of ΔCt of 18S RNA gene, control gene). Note that the relative fold change in expression of *PoptrIAA3.2 *gene in stem with respect to root (tissue with lowest *PoptrIAA3.2 *expression level) is 128, which has been represented on a discontinuous y-axis to capture the other lower fold change values observed.

**Figure 4:**
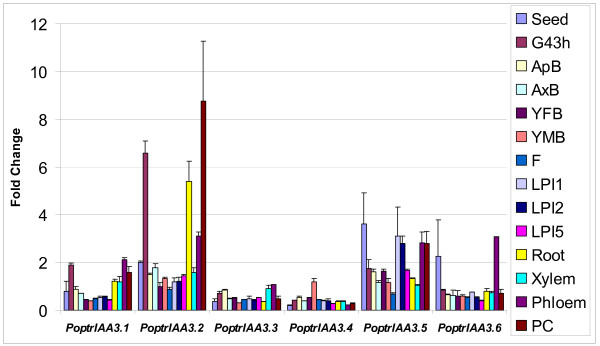
**Microarray expression support for *PoptrIAA3 *subgroup**. Data are expressed as fold change from negative controls, which consisted of the 95^th ^percentile signal from presumably unexpressed transposable element target sequences. Error bars represent standard errors from two biological replicates. G43h, germinant 43 hours after imbibition; ApB, apical bud; AxB, axillary bud; YFB, young female bud; YMB, young male bud; F, female catkin, post-fertilization; LPI1, leaf plastochron index 1; LPI2, leaf plastochron index 2; LPI5, leaf plastochron index 5; PC, phloem plus cortex. Samples are further defined in Additional file 17.

*PoptrIAA3.1–3.6 *represent a six-member *PoptrIAA3 *subgroup that groups closely with *AtIAA1–4*. This subgroup provides striking evidence for functional divergence following selective retention of duplicated genes. While the *Arabidopsis shy2 *mutant, carrying a gain-of-function mutation in *AtIAA3*, has upcurled leaves, slower gravitropic response, shorter hypocotyls and fewer lateral roots [22], a functional role for *AtIAA4 *is yet to be assigned. Gene-specific real-time RT-PCR showed that genes in the *PoptrIAA3 *subgroup display differential expression between leaf, stem and root tissues (Figure 3). *PoptrIAA3.2 *was found to have a higher expression level by several fold in stem than in roots. In an earlier study of aspen *Aux/IAA *genes, the *PoptrIAA3.2*-like gene had highest expression in developing xylem [24], which is also supported by our microarray results (Figure 4). EST data suggest that *PoptrIAA3.1 *and *3.2 *appear to be preferentially expressed in the cambial zone and during wood formation (See Additional file 6), and the microarray data suggest that these genes are also strongly expressed in newly germinated seedlings. *PoptrIAA3.6*-like ESTs are found in libraries of dormant buds and senescing leaves. *PoptrIAA3.4 *has a distinct expression pattern compared to other *PoptrIAA3 *genes, with detectable ESTs in male and female catkins, and highest expression in floral buds as determined by the microarray (Figure 4).

### Identification and sequence analysis of *Populus ARF *genes

A total of 39 predicted *ARF *genes (henceforth referred to as *PoptrARF*) were found in the *Populus *genome, compared to 23 genes reported from the *Arabidopsis *genome (henceforth referred to as *AtARF*) [2] and to 25 genes reported from the rice genome (henceforth referred to as *OsARF*) [29]. *In silico *mapping of gene loci showed *PoptrARF *genes are present on all chromosomes except VII, XIII, XVII and XIX (Figure 1). Eleven out of 39 genes lie within unassembled scaffolds. Conserved domain evaluations showed that four gene models (*PoptrARF3.3, 6.3, 16.5 and 16.6*) appear to lack one or more domains that are otherwise conserved in their closest sequence ortholog (See Additional files 10, 11 and 12).

Sixteen *PoptrARF *gene pairs (82% of total) are estimated to be represented in chromosomal segmental duplications arising out of the salicoid whole genome duplication event. Two genes, *PoptrARF16.4 *and *PoptrARF16.5*, (5% of total) are represented as one tandem duplication pair. The *Arabidopsis *genome contains a group of seven tandemly duplicated *ARF *genes that thus far have not been observed in other plant species including *Populus *and rice.

### Comparative analysis of *Populus *and *Arabidopsis ARF *gene families

**Figure 5:**
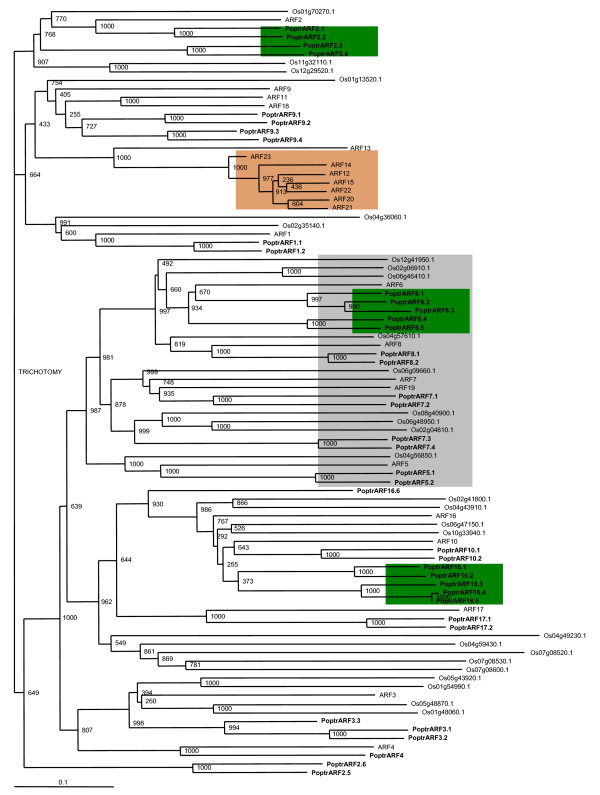
**Phylogenetic analysis of predicted full-length ARF protein sequences using Neighbor-Joining method**. Amino acids sequences of full-length predicted proteins were aligned using MUSCLE program. Tree was produced as described in methods. Bootstrap support is indicated at each node. Green and orange boxes represent sustained expansion of subgroups in *Populus *and *Arabidopsis*, respectively. Grey box represents the Q-rich activator ARF subgroup.

Phylogenetic analysis using known and predicted *Populus*, *Arabidopsis *and rice ARF protein sequences shows distinct gene family histories even between the two dicots (Figure 5). The ratio of activator ARFs (defined by the Q-rich middle region) in *Arabidopsis *and *Populus *is 1:2.6 whereas the ratio of repressor and other ARFs is 1:1.4, indicating a two-fold enrichment of activator ARFs during *Populus *evolution.

A pair of *Populus *genes, *PoptrARF7.3 *and *PoptrARF 7.4*, was found to group closely with three *OsARF *genes but had no obvious *Arabidopsis *orthologs. The loss-of-function *ARF7 *mutant displays altered leaf expansion, lateral root formation [30] and hypocotyl phototropism [31] in *Arabidopsis*. *PoptrARF7.3 *has EST support from a tension wood library (See Additional file 6), and microarray data indicated that *PoptrARF7.3 *has higher expression in xylem tissue (See Additional files 7, 8 and 9). It is possible that analogous to AtARF7's role in auxin dependent differential growth in aerial plant form [31], *PoptrARF7.3 *may be involved in differential growth in woody stems in response to tension stress.

At least three different singleton *ArabidopsisARF *genes (*AtARF2, 6 *and *16*) were found to cluster with four or more *Populus *genes each. *PoptrARF6.4 *and *PoptrARF6.5 *are likely co-orthologs of an ancestral gene lost in Arabidopsis. *AtARF 6 *and its *Populus *co-orthologs have Q-rich middle regions. Potato *ARF6*, which is similar to *AtARF6*, is reported to be involved in meristem activation [32]. Sprouting buds, apical meristem and leaf tips were reported to have the highest *ARF6 *transcript levels. While *PoptrARF6.1 *has EST support from cambial zone and tension wood tissue libraries, *PoptrARF6.4*, has expression support from a dormant bud library and *PoptrARF6.2 *has EST support from floral bud libraries. Furthermore, our microarray experiments indicated that the five members of the *PoptrARF6 *subgroup showed differential patterns of expression. *PoptrARF6.1 *and *6.4 *were expressed at low levels, with peaks in mature leaves and phloem-cortex samples. In contrast, *PoptrARF6.2 *and *6.3 *were strongly expressed across most tissue types, with particularly strong expression in xylem, phloem, and vegetative and reproductive meristems. Finally, *PoptrARF6.5 *was not significantly expressed in any of the tissues tested (See Additional files 7 and 8). Interestingly, *AtARF6 *and *8 *(double mutant) are associated with floral development in *Arabidopsis *[33]. They also display reduced stature, possibly due to reduced apical meristem activity. Though further experimental validation is required, preliminary information suggests that this subgroup has functional roles in controlling meristematic activity in distinct tissues and developmental stages.

*AtARF2 *negatively regulates differential growth in *Arabidopsis *hypocotyls [34]. *AtARF2 *mutants are reported to have defective floral structures [35]. T-DNA insertion mutants of *AtARF2 *have larger rosette leaves and reduced number and size of other aerial organs including inflorescences [33]. *AtARF2 *T-DNA insertion mutants also exhibit extra cell division and expansion in seeds and other vegetative and floral organs[35]. *Populus *has four putative *AtARF2 *orthologs (Figure 5). *PoptrARF2.5 *and *PoptrARF2.6 *are likely co-orthologs of an ancestral gene lost in Arabidopsis. Based on microarray and EST support, *PoptrARF2.1 *and 2.2 appear to express almost ubiquitously, with particularly strong expression in xylem and phloem. *PoptrARF2.3 *and *PoptrARF2.4 *were expressed in vegetative and floral buds, as well as in the cambial (cell division) zone. Tissue distribution of *Populus *ESTs and array results suggest that these sequence orthologs may possess similar functional contexts in *Populus *and *Arabidopsis*.

Characterization of the *ettin (ett*) mutant revealed that *AtARF3 *is involved in floral meristem, gynoecium, stamen and perianth patterning [36]. *Populus *has three *ARF3*-like genes out of which, *PoptrARF3.1 and 3.2 *had some EST and microarray expression support from vegetative and reproductive buds, but surprisingly these genes were most strongly expressed in xylem and phloem. *PoptrARF3.3 *was not included on the microarray because the gene model was artificially truncated in the initial annotation.

*Monopteros *(*mp*), a loss-of-function *AtARF5 *mutant, has a severely malformed embryonic axis and vascular system, and defective inflorescences where lateral flowers are completely lacking or reduced in number [37]. *Populus *has two putative orthologs of *AtARF5*, *PoptrARF5.1 *and *5.2*, and RT-PCR and microarray results indicate slightly higher expression in roots compared to stem and leaves (See Additional files 7, 8 and 13). *Populus *EST and microarray data show that *PoptrARF5.2 *is highly expressed in floral buds. It remains to be determined if these co-orthologs could be considered as sub-functionalized with respect to *AtARF5 *(or common ancestor) or if they play additional roles in tree development.

### Evolution, divergence and regulation of *Aux/IAA *and *ARF *gene families

Genome-wide duplications followed by a series of reciprocal tandem terminal fusions have resulted in a dramatic reorganization of the duplicated genome segments in *Populus*. Ensuing gene loss and expansion events have contributed toward divergence in gene family structures between the dicots, *Arabidopsis *and *Populus *[13,38]**. **Moreover, the modes of expansion, either through tandem or segmental duplication, differ between members of each gene family.

Phylogenetic analysis revealed a few subgroups such as IAA16 and ARF4 that contained sequence representatives in *Populus *and *Arabidopsis *but not in rice indicating that these subgroups were acquired or differentially retained in dicots post-divergence from monocots.

Contrary to the *ARF *gene family, *Aux/IAA *gene family was observed to have expanded largely due to segmental duplications in *Arabidopsis *[38]. Segmental duplications have also contributed to expansion of both these gene families in rice [12,29]. Our study indicates that *PoptrIAA *as well as *PoptrARF *genes have been largely retained at a higher than average rate following the salicoid genome-wide duplication and rearrangement events. On a genome-wide scale, approximately 14,000 of the 45,000 (~32%) predicted genes are retained in duplicated pairs resulting from the salicoid duplication event [13]. The retention rates for *PoptrIAA *and *PoptrARF *families are 80% and 82% respectively. This is in line with the expectation that genes involved in transcription regulation and signal transduction are preferentially retained following duplication [39-41]. The low proportion of retained tandem duplicates (5%) in the *PoptrARF *gene family compared to the *PoptrIAA *family is potentially due to constraints associated with dimer stoichiometry maintenance for ARF transcription factor activity as tandem clusters do exist for other *Populus *gene families [13]. Even though 43% of *PoptrIAA *genes are represented in tandem clusters of two to three genes each, nearly 86% of these tandem clusters have likely expanded and been retained following chromosome-level segmental duplication or whole-genome duplication events. These observations suggest that *PoptrIAA *and *PoptrARF *transcription factor families consist of genes originating primarily from high segmental (large-scale) and secondarily from low tandem (small-scale) duplication events. Sub-functionalization, neo-functionalization and non-functionalization events associated with duplicate transcription factor genes carry a greater weight for functional ramifications such as the ability to differentially regulate auxin signal transduction pathways. It could be speculated, based primarily on our understanding of the functional role of the nearest *Arabidopsis *ortholog [33-35,42], that the presence of multiple ARF2 co-orthologs in the *Populus *genome could reflect the greater need for temporal control on cell division and expansion in the context of flowering, senescence and abscission in *Populus*, a perennial deciduous tree that exhibits seasonal dynamics and transitioning between juvenile to mature to reproductive stages across a time-span of years as opposed to days as in the annual *Arabidopsis*. Considering the complexity of ARF- and Aux/IAA- mediated regulation, the reasons for and implications of diversification will require further understanding using evolutionary systems biology studies [43].

Closely-related *ARF*s that represent pairs of sister loci have been found to have strong double-mutant phenotypes and overlapping expression domains [33]. Furthermore, interaction affinities were found to be stronger among intra-group (activator or repressor ARF groups) members when compared to inter-group members [44]. Moreover, single and double mutants resulting from *AtIAA12 *gain-of-function and *ARF5 *loss-of-function mutants have similar mutant phenotypes. It is proposed that *ARF5 *may act in a positive and *IAA12 *in negative regulatory way to control embryogenesis and root meristem development in an auxin-dependent manner [21]. RT-PCR results show that *PoptrARF5 *and *PoptrIAA12 *genes display contrasting expression patterns in roots (See Additional file 13). The high expression of *PoptrARF5 *in roots and low expression of *PoptrIAA12 *suggests that they may co-regulate root development in *Populus *in an auxin dependent manner. The potential number of heterotypic interactions between *Populus *ARFs and Aux/IAAs are likely several times greater than the number of members in these two large gene families. This may contribute to higher-order auxin signal and response mechanisms needed by perennial plants to achieve greater developmental plasticity.

The auxin response mechanism has recently been shown to be also regulated through small noncoding RNA species; microRNA (miRNA) and trans-acting short-interfering RNA (ta-siRNA). *AtARF 10*, *16 *and *17 *are known to be regulated by miR160, a miRNA group that is highly conserved across the plant kingdom [45,46] and *AtARF6 *and *AtARF8 *have shown to be regulated by miR167 [47]. Both miR160 and miR167 families are predicted to be two-fold overrepresented in the *Populus *genome when compared to *Arabidopsis *[13]. Target sequences for miR160 have been detected in *PoptrARF10.1*-*10.2*, *PoptrARF16.1–16.5 *and *PoptrARF17.1–17.2 *and miR167 targets have been found in *PoptrARF6.1–6.3 *and *PoptrARF8.1–8.2*.

*TAS3 *ta-siRNA has been shown to mediate post-transcriptional regulation of *ARF3 *and *ARF4 *gene expression [48,49] and to play a key role in juvenile to adult phase transition in *Arabidopsis *[50,51]. The ta-siRNA target site sequences were reported to be conserved among *AtARF2*, *AtARF3 *and *AtARF4 *and related rice and wheat sequences [49]. Homologous conserved sites were found to occur once in *PoptrARF2.2 *and *PoptrARF2.6 *and twice in *PoptrARF3.1*, *PoptrARF3.2 *and *PoptrARF4 *genes. GenBank database searches showed that grape, tobacco, medicago and tomato ARF3-like sequences also carry two conserved ta-siRNA target sites. The individual *PoptrARF *co-orthologs as well as the different genes represented in the miR and tasiR groups provide myriad opportunities for complex regulatory interactions of auxin-related transcription in *Populus*.

## Conclusion

This study identified the suites of *Aux/IAA *and *ARF *genes in the *Populus *genome and revealed their gene family structures. Comparative genomics with *Arabidopsis *and rice suggested that the two gene families have a common origin, including conservation of activator groups in ARF gene families of all three genomes, but have experienced separate evolutionary histories, diverging in important ways, including the lack of large clusters of tandemly duplicated *AtARF *genes from *Populus *and rice, lack of *PoptrARF7.3 *and *PoptrARF7.4 *orthologs in *Arabidopsis *and substantial expansion of certain *PoptrIAA *and *PoptrARF *subgroups through large-scale segmental duplications. Overall, the gene family structures of *Populus *and *Arabidopsis *displayed a greater degree of conservation with each other than in comparison with the monocot plant, rice.

The *Populus *genome sequence and the findings reported here provide new opportunities to facilitate postulation and exploration of hypotheses linking auxin response regulatory genes to conserved core plant processes, as well as perennial plant features such as wood development, long-distance nutrient and water movement, seasonal dynamics, and disease resistance. Targeted reverse genetics studies, high-resolution spatiotemporal expression surveys as well as investigations of *in vivo *homo- and hetero-dimerization affinities of the various *Aux/IAA *and *ARF *gene family members among taxa will need to be carried out to understand how the molecular functions of these genes translate into a diverse suite of auxin-mediated effects at the whole-plant level. Further studies on basic aspects of the functional contexts of Aux/IAA and ARF proteins will open opportunities for applications in agricultural, forestry, environmental and energy sectors. One such application includes ongoing functional genomics studies to investigate the roles of *PoptrIAA *and *PoptrARF *genes in carbon allocation and carbon sequestration.

## Methods

### Identification of *Aux/IAA *and *ARF *gene families in *Populus*

Genes were initially identified using Pfam domain IDs assigned to predicted *Populus *gene models in the JGI (DOE Joint Genome Institute, CA) annotation pipeline [13], and by using *Arabidopsis *ARF and Aux/IAA proteins [2,11] as queries in BLASTP searches of predicted *Populus *proteins (*Populus *Genome v 1.1, January 2007). *Populus *proteins identified in this initial search were used as query sequences in additional BLASTP searches of the predicted *Populus *protein set for exhaustive identification of divergent *Populus *gene family members. Redundant and invalid gene models were verified based on gene structure, intactness of conserved motifs, EST support and synteny analysis. We have also included in our study an incomplete gene model (fgenesh4_pg.C_scaffold_1006000001) representative of *PoptrARF6.3 *because this gene model is flanked by a sequence gap followed by the conserved ARF amino terminus domains, which could potentially be corrected into a complete gene model in the upcoming version of the genome. Furthermore, this gene showed very strong evidence of expression based on microarray analyses. Sequence conservation and microsynteny analysis of *Populus *gene models with *Populus *homeologous (duplicated) genomic regions and the *Arabidopsis *genome was conducted using the Vista Browser tool with default curve calculation parameters; nucleotide sequence 'conservation identity' of 70% and 'minimum conservation width' of 100 bp [52]. One gene model per locus, which included some JGI annotated models representing the promoted or reference set, were used in this study. It should also be noted that in the current version of the genome (v 1.1), some of the scaffolds could potentially represent haplotypes and not unique unassembled genomic regions. Gene nomenclature is based on the consensus standard established by the International *Populus *Genome Consortium (IPGC) to distinguish *Populus trichocarpa *from other *Populus *species.

### Chromosomal mapping of *PoptrIAA *and *PoptrARF *genes and estimation of duplicate genes

Information on chromosomal location was gathered from the *Populus *genome browser [53]. Chromosomal locations were determined by integrating the genome assembly with a microsatellite-based genetic map for *P*. *trichocarpa *× *P*. *deltoides *[13,54]. The physical chromosomal location was represented in a graphical output by scaling the 19 chromosomes (depicting regions of genome-wide duplications as in Tuskan *et al*., 2006) followed by scale-guided positioning or mapping of the loci. Identification of homeologous chromosome segments resulting from whole-genome duplication events was accomplished as described in Tuskan et al., 2006. Briefly, global alignments were performed using the double-affine Smith-Waterman algorithm. Genetic distances between pairs were calculated as the proportion of four-fold degenerate nucleotide sites that underwent transversions (4DTV distance). 4DTV stands for four-fold synonymous (degenerative) third-codon transversion. It represents a transversion in the third nucleotide position within four codons that does not result in a change in corresponding amino acid identity within the protein it codes for. Such an estimate of synonymous mutation rate within a transcribed region of a gene but not in region that experiences selection is a conserved means of estimating divergence within the more recent evolutionary past. Distances corresponding to the 'salicoid' whole-genome duplication events were delineated based on discrete peaks in 4DTV distributions. Duplicated segments were defined as regions on different linkage groups or scaffolds containing six or more homeologous pairs with similar 4DTV values, with fewer than 25 nonhomeologous genes intervening. Gene pairs resulting from the 'salicoid' duplication (apparently common to the Salicaceae) were defined by 4DTV values between 0.04 and 0.17. Microsynteny in flanking regions of segmental duplicates was verified using the Genome Browser. Tandemly duplicated genes that matched the same homeolog were only counted once for this analysis.

### Sequence and phylogenetic analysis

Conserved protein motifs were determined from CD-searches [55] and using MEME-MAST programs [56,57]. Sequence identity between two genes was determined using the bl2seq tool [58]. Multiple sequence alignments were performed using MUSCLE sequence alignment program [59]. Phylogenetic trees were constructed in two ways using amino acid sequence alignments of conserved regions or full-length sequences of all predicted proteins in *Aux/IAA *and *ARF *gene families of *Populus*, *Arabidopsis *[2,9,11] and rice [12,29]. *Arabidopsis *and rice sequences were obtained from TAIR and TIGR databases. Unrooted PHYLIP trees with 1000 bootstraps were generated by Neighbor-Joining method using ClustalX 1.83 program. Phylograms were visualized in TreeView v1.6.6. Bayesian phylogenetic analysis of conserved, collated and aligned Aux/IAA (See Additional file 14) and ARF amino acid sequences (See Additional file 15) was performed using the MRBAYES (version 3.1.2) package [60,61]. We used the WAG substitution frequency matrix [62] with among-sites rate variation modeled by means of a discrete γ distribution with four equally probable categories. Two independent runs of 1–2 million Monte Carlo Markov Chain generations with four chains each were run. Trees were sampled every 100 generations and stationary phase and burnin value was determined by plotting the likelihood scores against number of generations. Posterior probabilities calculated from consensus are shown on branches.

### RNA isolation and real-time PCR analysis

Leaf, stem and root tissue material were collected from greenhouse-grown *P*. *trichocarpa *(Nisqually-1)plants and immediately frozen in liquid nitrogen. Total RNA was extracted from plant samples using plant RNeasy kit (Qiagen, CA) followed with DNase I treatment. The quality and quantity of RNA was evaluated using a Nanodrop spectrophotometer and gel electrophoresis. cDNA was synthesized from two micrograms of RNA using SuperScriptIII (Invitrogen, CA) according to manufacturers' instructions. Real-time PCR was carried out using cDNA, primer pairs (See Additional file 16) and iQ SYBR mix (BioRad, CA). Samples were run in triplicate in an iCycler real-time PCR machine (BioRad, CA) and normalized with respect to18S threshold cycle values. Fold change was calculated by the formula 2^-ΔΔCt^, where ΔΔCt is the difference between ΔCt of a gene in a given tissue and the lowest value ΔCt for that gene in any of the three tissue types. ΔCt was estimated by the formula; Ct of gene of interest – geometric mean of ΔCt of 18S RNA gene (control gene). EST information was obtained from GenBank and the *Populus *EST database, PopulusDB [63].

### Microarray analysis

We used a whole genome microarray that we designed together with NimbleGen Systems, Inc. The array targets 55,794 predicted transcripts from the poplar genome sequencing project. There are three different isothermal 60 mer probes per target, designed for maximum specificity. All probes are replicated once on the array for a total of over 385,000 probes synthesized *in situ *on glass slides [64].

Tissues for microarray hybridizations were obtained from field and greenhouse-grown trees, and tissue collection methods varied depending on the tissue type. All tissue was obtained from *Populus trichocarpa *clone Nisqually-1, except for floral tissue and seeds, which were collected from trees growing in the wild near Corvallis, Oregon. Tissues and abbreviations are listed in Additional file 17. Two biological replicates were used for each of 12 tissue types, and three biological replicates were used for xylem and phloem samples. RNA was extracted as described for RT-PCR analyses. Labeling, hybridization, and scanning were carried out by NimbleGen using their standard expression array protocols.

Array data (See Additional file 18) were normalized using the NimbleGen microarray data processing pipeline (NMPP) [65]. We used a two step normalization procedure starting with an initial quantile normalization among replicates within each tissue, and then a global normalization to adjust all tissues to a similar baseline. Gene-level analyses were performed using the mean normalized fluorescence values for all probes and replicates. For negative controls we used probes targeting 3,149 transposable elements that were contaminants in the initial release of *Populus *gene models. This approach is warranted because the vast majority of these elements will be quiescent at any particular time, an assumption that is supported by the significantly lower hybridization signals from these probes compared to all other groups of targets on the array (data not shown). For each experiment we defined the expression threshold as the 95^th ^percentile of the fluorescence values for all negative control targets. Relative expression of individual genes was assessed as fold-change from this negative control threshold value. A two-fold change was considered to be evidence of significant expression.

## Authors' contributions

UCK carried out the sequence, phylogenetic and expression analyses and drafted the manuscript. SPD contributed to sequence analysis and together with AMB generated the microarray data and carried out data analysis. GAT contributed to the conception of the study. All authors read and approved the final manuscript.

## Supplementary Material

Additional File 1 List of all *ARF *and *Aux/IAA *genes predicted in the *P. trichocarpa *genome. Click here for file

Additional File 2 Summary of Vista conservation- microsynteny analysis. "yes" and "no" refer to existence of at least 70% identity within a 100 bp sliding window between Poplar v.1.0, Poplar duplicates and *Arabidopsis thaliana *sequences. Click here for file

Additional File 3 Multiple sequence alignment of full-length amino acid sequences of predicted *Populus*, *Arabidopsis *and rice Aux/IAA proteins. Sequences were aligned using MUSCLE program. Consensus sequence is indicated at the bottom of the alignment. Click here for file

Additional File 4 Multiple sequence alignment of conserved regions of predicted *Populus*, *Arabidopsis *and rice Aux/IAA protein sequences. Sequences were aligned using MUSCLE program. Consensus sequence is indicated at the bottom of the alignment. Click here for file

Additional File 5 Prediction of conserved domains in full-length amino acid sequences of predicted *Populus*, *Arabidopsis *and rice Aux/IAA proteins. Conserved domains were predicted using the MEME and MAST programs. Motif number 6 represents the conserved domain I, Motif number 3 represents the conserved domain II, Motif number 2 represents the conserved domain III and Motif numbers 1, 4 and 5 represent the conserved domain IV. Click here for file

Additional File 6 EST support for *Populus Aux/IAA *and *ARF *genes. Numbers indicate the number of ESTs represented in each tissue library. Source: the *Populus *EST database, PopulusDB. Click here for file

Additional File 7 Fold change data from microarray expression analyses for *PoptrIAA *and *PoptrARF *genes. Data are presented as normalized expression values of target genes relative to the 95^th ^percentile of expression values for transposable element targets (presumably unexpressed). Genes with a greater than 2-fold difference from negative controls are highlighted in bold. Sample abbreviations are defined in Additional file 17. Click here for file

Additional File 8 Normalized raw data from microarray expression analyses for *PoptrIAA *and *PoptrARF *genes. Data were normalized using quantile normalization within replicates and then across tissue types. Data are presented as relative expression values. Also included are 95^th ^percentile of normalized expression for negative control probes. Sample abbreviations are defined in Additional file 17. Click here for file

Additional File 9 Temperature diagram representation of microarray results. Figures are temperature diagrams with intensity of signal calculated in columns, which are derived from means for all data for a particular tissue type. The bottom line in each figure represents the negative control. Red means higher expression, dark blue means lower expression. Sample abbreviations are defined in Additional file 17. Click here for file

Additional File 10 Multiple sequence alignment of full-length amino acid sequences of predicted *Populus*, *Arabidopsis *and rice ARF proteins. Sequences were aligned using MUSCLE program. Consensus sequence is indicated at the bottom of the alignment. Click here for file

Additional File 11 Multiple sequence alignment of conserved regions of predicted *Populus*, *Arabidopsis *and rice ARF protein sequences. Sequences were aligned using MUSCLE program. Consensus sequence is indicated at the bottom of the alignment. Click here for file

Additional File 12 Prediction of conserved domains in full-length amino acid sequences of predicted *Populus*, *Arabidopsis *and rice ARF proteins. Conserved domains were predicted using the MEME and MAST programs. Motif numbers 1, 2, 3, 4, 7 and 11 represent the conserved B3 domain. Motif numbers 6, 8 and 12 represent the conserved auxin response domain and Motif numbers 5 and 10 represent the conserved C-terminal Aux/IAA domain (III and IV). Click here for file

Additional File 13 Expression analysis of *PoptrARF5 *and *PoptrIAA12 *genes using real-time RT-PCR. * Fold Change is represented relative to lowest value observed for the gene. Lowest value was determined by comparison of relative threshold cycle values for a specific gene across leaf, stem and root samples. Fold change was calculated by the formula 2^-ΔΔCt^, where ΔΔCt is the difference between ΔCt of a gene is a given tissue and the lowest value ΔCt for that gene in any of the three tissue types. ΔCt was estimated by the formula; (Ct of gene of interest) – (geometric mean of ΔCt of 18S RNA gene, control gene). Click here for file

Additional File 14 Bayesian phylogenetic analysis of conserved regions of predicted Aux/IAA protein sequences using MRBAYES. Amino acid sequences of full-length predicted proteins were aligned using MUSCLE program. Tree was produced using conserved collated regions (See Additional file 4) as described in methods. Posterior probabilities calculated from consensus are shown on branches. Green boxes represent sustained expansion of subgroups in *Populus*. Click here for file

Additional File 15 Bayesian phylogenetic analysis of conserved regions of predicted ARF protein sequences using MRBAYES. Amino acid sequences of full-length predicted proteins were aligned using MUSCLE program. Tree was produced using conserved collated regions (See Additional file 11) as described in methods. Posterior probabilities calculated from consensus are shown on branches. Green and orange boxes represent sustained expansion of subgroups in *Populus *and *Arabidopsis*, respectively. Grey box represents the Q-rich activator ARF subgroup. Click here for file

Additional File 16 Nucleotide sequences of primers used in RT-PCR experiments. Click here for file

Additional File 17 Tissues used for microarray experiments. A description of sources of RNA and collection methods for array experiments. Click here for file

Additional File 18 Microarray data for *PoptrIAA *and *PoptrARF *gene families from the NimbleGen *Populus *whole genome microarray version 1.0. Data are means among all probes for the specified target, normalized as described in the text. Different columns for the same tissue type are biological replicates. Negative controls are 95th percentile of expression levels for 3155 Transposable Elements from the *Populus *genome. Click here for file
