# Supplementary material for: Genome-wide analysis of Aux/IAA and ARF gene families in Populus trichocarpa
Source: BMC Plant Biol. 2007 Nov 6;7:59. doi: 10.1186/1471-2229-7-59 (PMC2174922; doi:10.1186/1471-2229-7-59)
Supplement: Additional File 3 — Multiple sequence alignment of full-length amino acid sequences of predicted Populus, Arabidopsis and rice Aux/IAA proteins. Sequences were aligned using MUSCLE program. Consensus sequence is indicated at the bottom of the alignment. [file 1471-2229-7-59-S3.pdf]

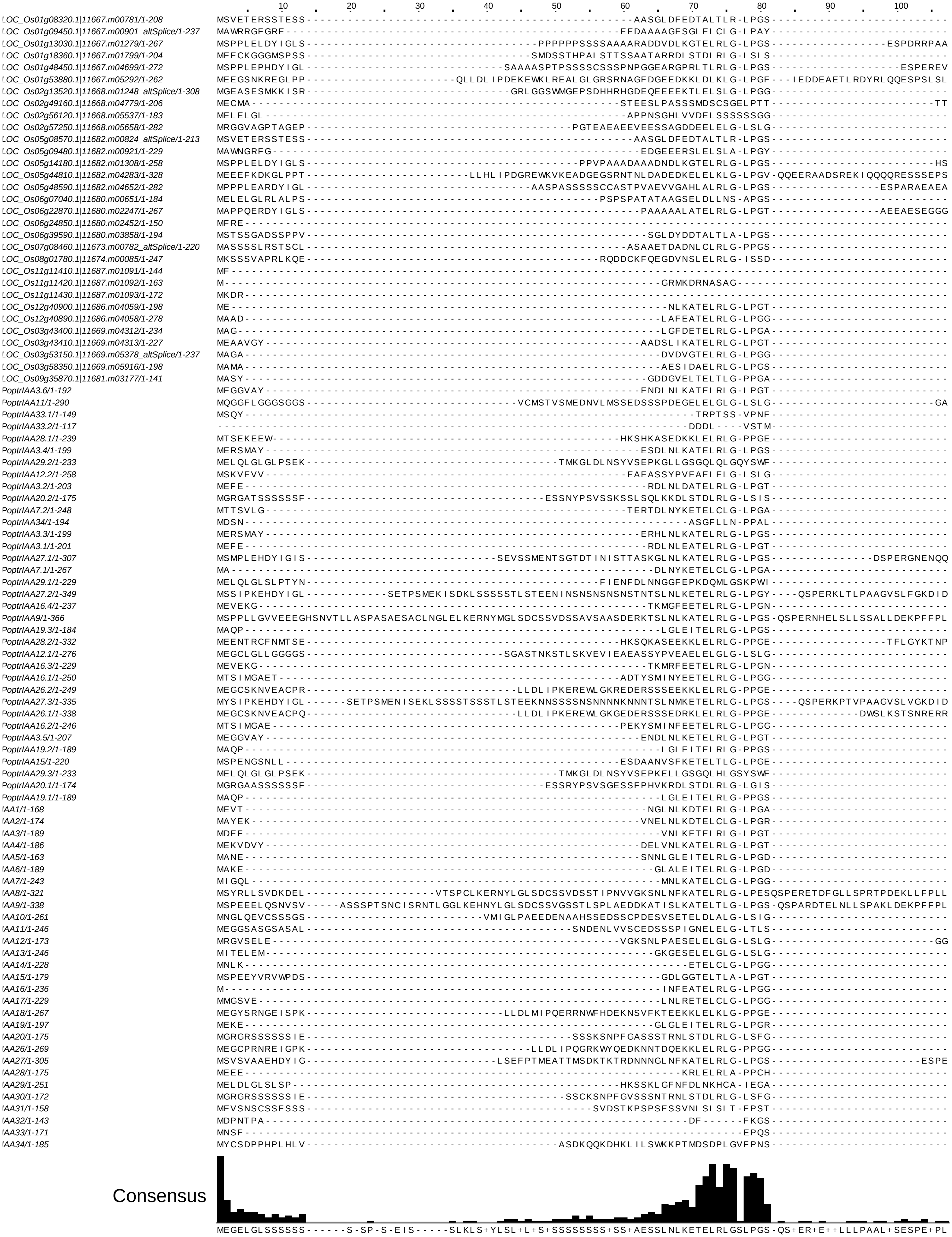

10 20 30 40 50 60 70 80 90 100
LOC_Os01g08320.1|11667.m00781/1-208 MSVETERSSTESS - AASGLDFEDTALTLR-LPGS -
LOC_Os01g09450.1|11667.m00901_altSplice/1-237 MAWRRGFGRE - EEDAAAAGESGLELCLG-LPAY -
LOC_Os01g13030.1|11667.m01279/1-267 MSPPLELDYIGLS - PPPPPPSSSSAAAARADDVDLKGTELRLG-LPGS - ESPDRRPAA
LOC_Os01g18360.1|11667.m01799/1-204 MEECKGGGMSPSS - SMDSSTHPALSTTSSAATARRDLSTDLRLG-LSLS -
LOC_Os01g48450.1|11667.m04699/1-272 MSPPLEPHDYIGL - SAAAASPTPSSSSCSSSPNPGGEARGPRLTLRLG-LPGS - ESPEREV
LOC_Os01g53880.1|11667.m05292/1-262 MEEGSNKREGLPP - QLLDLIPDEKEWKLREALGLGRSRNAGFDGEEDKKLDLKLG-LPGF - IEDDEAETLRDYRLQQESPSLSL
LOC_Os02g13520.1|11668.m01248_altSplice/1-308 MGEASESMKKISR - GRLGGSWMGEPSDHHRHGDEQEEEKTLELSLG-LPGG -
LOC_Os02g49160.1|11668.m04779/1-206 MECMA - STEESLPASSSMDSCSGELPTT - TT
LOC_Os02g56120.1|11668.m05537/1-183 MELELGL - APPNSGHLVVDELSSSSSSGG -
LOC_Os02g57250.1|11668.m05658/1-282 MRGGVAGPTAGEP - PGTEAEAEEVEESSAGDDEELELG-LSLG -
LOC_Os05g08570.1|11682.m00824_altSplice/1-213 MSVETERSSTESS - AASGLDFEDTALTLR-LPGS -
LOC_Os05g09480.1|11682.m00921/1-229 MAWNGRFG - EDGEEERSLELSLA-LPGY -
LOC_Os05g14180.1|11682.m01308/1-258 MSPPLELDYIGLS - PPVPAAADAAADNDLKGTELRLG-LPGS - HS
LOC_Os05g44810.1|11682.m04283/1-328 MEEEFKDKGLPPT - LLHLIPDGREWKVKEADGEGSRNTNLDADEDKELELKLG-LPGV-QQEERAADSREKIQQQQRESSSEPS
LOC_Os05g48590.1|11682.m04652/1-282 MPPPLEARDYIGL - AASPASSSSSCCASTPVAEVVGAHLALRLG-LPGS - ESPARAEAEA
LOC_Os06g07040.1|11680.m00651/1-184 MELELGLRLALPS - PSPSPATATAAGSELDLLNS-APGS -
LOC_Os06g22870.1|11680.m02247/1-267 MAPPQERDYIGLS - PAAAAALATELRLG-LPGT - AEEAESEGGG
LOC_Os06g24850.1|11680.m02452/1-150 MFRE -
LOC_Os06g39590.1|11680.m03858/1-194 MSTSSGADSSPPV - SGLDYDDTALTLA-LPGS -
LOC_Os07g08460.1|11673.m00782_altSplice/1-220 MASSSSLRSTSCL - ASAAETDADNLCLRLG-PPGS -
LOC_Os08g01780.1|11674.m00085/1-247 MKSSSVAPRLKQE - RQDDCKFQEGDVNSLELRLG-ISSD -
LOC_Os11g11410.1|11687.m01091/1-144 MF -
LOC_Os11g11420.1|11687.m01092/1-163 M - GRMKDRNASAG -
LOC_Os11g11430.1|11687.m01093/1-172 MKDR -
LOC_Os12g40900.1|11686.m04059/1-198 ME - NLKATELRLG-LPGT -
LOC_Os12g40890.1|11686.m04058/1-278 MAAD - LAFEATELRLG-LPGG -
LOC_Os03g43400.1|11669.m04312/1-234 MAG - LGFDETELRLG-LPGA -
LOC_Os03g43410.1|11669.m04313/1-227 MEAAVGY - AADSLIKATELRLG-LPGT -
LOC_Os03g53150.1|11669.m05378_altSplice/1-237 MAGA - DVDVGTELRLG-LPGG -
LOC_Os03g58350.1|11669.m05916/1-198 MAMA - AESIDAELRLG-LPGS -
LOC_Os09g35870.1|11681.m03177/1-141 MASY - GDDGVELTELTLG-PPGA -
PoptrIAA3.6/1-192 MEGGVAY - ENDLNLKATELRLG-LPGT -
PoptrIAA11/1-290 MQGGFLGGGSGGS - VCMSTVSMEDNVLMSSEDSSSPDEGELELGLG-LSLG - GA
PoptrIAA33.1/1-149 MSQY - TRPTSS-VPNF -
PoptrIAA33.2/1-117 - DDDL----VSTM -
PoptrIAA28.1/1-239 MTSEKEEW - HKSHKASEDKKLELRLG-PPGE -
PoptrIAA3.4/1-199 MERSMAY - ESDLNLKATELRLG-LPGS -
PoptrIAA29.2/1-233 MELQLGLGLPSEK - TMKGLDLNSYVSEPKGLLGSGQLQLGQYSWF -
PoptrIAA12.2/1-258 MSKVEVV - EAEASSYPVEAELELG-LSLG -
PoptrIAA3.2/1-203 MEFE - RDLNLDATELRLG-LPGT -
PoptrIAA20.2/1-175 MGRGATSSSSSSF - ESSNYPSVSSKSSLSQLKKDLSTDLRLG-LSIS -
PoptrIAA7.2/1-248 MTTSVLG - TERTDLNYKETELCLG-LPGA -
PoptrIAA34/1-194 MDSN - ASGFLLN-PPAL -
PoptrIAA3.3/1-199 MERSMAY - ERHLNLKATELRLG-LPGS -
PoptrIAA3.1/1-201 MEFE - RDLNLEATELRLG-LPGT -
PoptrIAA27.1/1-307 MSMPLEHDYIGIS - SEVSSMENTSGTDTINISTTASKGLNLKATELRLG-LPGS - DSPERGNENQQ
PoptrIAA7.1/1-267 MA - DLNYKETELCLG-LPGA -
PoptrIAA29.1/1-229 MELQLGLSLPTYN - FIENFDLNNGGFEPKDQMLGSKPWI -
PoptrIAA27.2/1-349 MSSIPKEHDYIGL - SETPSMEKISDKLSSSSSTLSTEENINSNSNSNSNSTNTSLNKETELRLG-LPGY----QSPERKLTLPAAGVSLFGKDID
PoptrIAA16.4/1-237 MEVEKG - TKMGFEETELRLG-LPGN -
PoptrIAA9/1-366 MSPPLLGVVEEEGHSNVTLLASPASAESACLNGLELKERNYMGLSDCSSVDSSAVSAASDERKTSLNLKATELRLG-LPGS-QSPERNHELSLLSSALLDEKPFFPL
PoptrIAA19.3/1-184 MAQP - LGLEITELRLG-LPGS -
PoptrIAA28.2/1-332 MEENTRCFNMTSE - HKSQKASEEKKLELRLG-PPGE - TFLGYKTNP
PoptrIAA12.1/1-276 MEGCLGLLGGGGS - SGASTNKSTLSKVEVIEAEASSYPVEAELELGLG-LSLG -
PoptrIAA16.3/1-229 MEVEKG - TKMRFEETELRLG-LPGN -
PoptrIAA16.1/1-250 MTSIMGAET - ADTYSMINYEETELRLG-LPGG -
PoptrIAA26.2/1-249 MEGCSKNVEACPR - LLDLIPKEREWLGKREDERSSSEEKKLELRLG-PPGE -
PoptrIAA27.3/1-335 MYSIPKEHDYIGL - SETPSMENISEKLSSSSTSSSTLSTEEKNNSSSSNSNNNNKNNNTSLNMKETELRLG-LPGS----QSPERKPTVPAAGVSLVGKDID
PoptrIAA26.1/1-338 MEGCSKNVEACPQ - LLDLIPKEREWLGKGEDERSSSEDRKLELRLG-PPGE - DWSLKSTSNRERR
PoptrIAA16.2/1-246 MTSIMGAE - PEKYSMINFEETELRLG-LPGG -
PoptrIAA3.5/1-207 MEGGVAY - ENDLNLKETELRLG-LPGT -
PoptrIAA19.2/1-189 MAQP - LGLEITELRLG-PPGS -
PoptrIAA15/1-220 MSPENGSNLL - ESDAANVSFKETELTLG-LPGE -
PoptrIAA29.3/1-233 MELQLGLGLPSEK - TMKGLDLNSYVSEPKELLGSGQLHLGSYSWF -
PoptrIAA20.1/1-174 MGRGAASSSSSSF - ESSRYPSVSGESSFPHVKRDLSTDLRLG-LGIS -
PoptrIAA19.1/1-189 MAQP - LGLEITELRLG-PPGS -
IAA1/1-168 MEVT - NGLNLKDTELRLG-LPGA -
IAA2/1-174 MAYEK - VNELNLKDTELCLG-LPGR -
IAA3/1-189 MDEF - VNLKETELRLG-LPGT -
IAA4/1-186 MEKVDVY - DELVNLKATELRLG-LPGT -
IAA5/1-163 MANE - SNNLGLEITELRLG-LPGD -
IAA6/1-189 MAKE - GLALEITELRLG-LPGD -
IAA7/1-243 MIGQL - MNLKATELCLG-LPGG -
IAA8/1-321 MSYRLLSVDKDEL - VTSPCLKERNYLGLSDCSSVDSSTIPNVVGKSNLNFKATELRLG-LPESQSPERETDFGLLSPRTPDEKLLFPLL
IAA9/1-338 MSPEEELQSNVSV----ASSSPTSNCISRNTLGGLKEHNYLGLSDCSSVGSSTLSPLAEDDKATISLKATELTLG-LPGS-QSPARDTELNLLSPAKLDEKPFFPL
IAA10/1-261 MNGLQEVCSSSGS - VMIGLPAEEDENAAHSSEDSSCPDESVSETELDLALG-LSIG -
IAA11/1-246 MEGGSASGSASAL - SNDENLVVSCEDSSSPIGNELELG-LTLS -
IAA12/1-173 MRGVSELE - VGKSNLPAESELELGLG-LSLG - GG
IAA13/1-246 MITELEM - GKGESELELGLG-LSLG -
IAA14/1-228 MNLK - ETELCLG-LPGG -
IAA15/1-179 MSPEEYVRVWPDS - GDLGGTELTLA-LPGT -
IAA16/1-236 M - INFEATELRLG-LPGG -
IAA17/1-229 MMGSVE - LNLRETELCLG-LPGG -
IAA18/1-267 MEGYSRNGEISPK - LLDLMIPQERRNWFHDEKNSVFKTEEKKLELKLG-PPGE -
IAA19/1-197 MEKE - GLGLEITELRLG-LPGR -
IAA20/1-175 MGRGRSSSSSSIE - SSSKSNPFGASSSTRNLSTDLRLG-LSFG -
IAA26/1-269 MEGCPRNREIGPK - LLDLIPQGRKWYQEDKNNTDQEKKLELRLG-PPGG -
IAA27/1-305 MSVSVAAEHDYIG - LSEFPTMEATTMSDKTKTRDNNNGLNFKATELRLG-LPGS - ESPE
IAA28/1-175 MEEE - KRLELRLA-PPCH -
IAA29/1-251 MELDLGLSLSP - HKSSKLGFNFDLNKHCA-IEGA -
IAA30/1-172 MGRGRSSSSSSIE - SSCKSNPFGVSSSNTRNLSTDLRLG-LSFG -
IAA31/1-158 MEVSNSCSSFSSS - SVDSTKPSPSESSVNLSLSLT-FPST -
IAA32/1-143 MDPNTPA - DF------FKGS -
IAA33/1-171 MNSF - EPQS -
IAA34/1-185 MYCSDPPHPLHLV - ASDKQQKDHKLILSWKKPTMDSDPLGVFPNS -
Consensus
MEGELGLSSSSSS-----S-SP-S-EIS----SLKLS+YLSL+L+S+SSSSSSS+SS+AESSLNLKETELRLGSLPGS-QS+ER+E++LLLPAAL+SESPE+PL

```
                                                      110        120        130        140        150        160        170        180        190        200        210
LOC_Os01g08320.1|11667.m00781/1-208                   -------LAAAAAPDPDRKRSSPSSSDAADA------------------------------------------ADNSSPLAAAA------------------
LOC_Os01g09450.1|11667.m00901_altSplice/1-237         -----------FSSSSSSKPSEGSTAAPAFAL---------------------------------------RSNGTNASKPSGAAA------------------
LOC_Os01g13030.1|11668.m01279/1-267                   IAAAAATATTLELLPAKGAKRVFPDEAALTPPT------------------------------AAAGKGKAARREGEEVGAEEE-------------DKKVA
LOC_Os01g18360.1|11667.m01799/1-204                   ----------TSSSSSLLQAAAAAAAAADDSIP------------------------------------------------------------------------
LOC_Os01g48450.1|11667.m04699/1-272                   VAAGLTLGPLPPTTTKAASKRAFPDSSPRHGA-------------------------------SSGSVAAAAACQDKAAPAAA------------------
LOC_Os01g53880.1|11667.m05292/1-262                   SFFPKHSKTTSSTTTTTGAKRGFIDTVEDKTEGYNDQKQQARAGCGKELAVEEMI-------------AAVSERKKGCCPPPPPPHGA---------PATPARNRP
LOC_Os02g13520.1|11668.m01248_altSplice/1-308         GWRAACRDKGTTTKHSIAAAAAADDDDGDKSSMLSLGYSTLVSHSQGK------------------ANKNKGSPEEEEAHPPPATG--------NNALASNNNG
LOC_Os02g49160.1|11668.m04779/1-206                   TAPAQSTASSGCRPPATAAKRRSLISTDLRLG---------------------------------------------------------------------------
LOC_Os02g56120.1|11668.m05537/1-183                   -----GSGSAPVSASSAGKRGFREAFQETL------------------------------LLFDDGSCCNTSDDDC---------------------------
LOC_Os02g57250.1|11668.m05658/1-282                   ---SKKQQQQHAPCRILTARDLQPAAALSPDSSVSSSS---------------------------PAAAAAAGGKRAEGPTATTS--------------PGTV
LOC_Os05g08570.1|11682.m00824_altSplice/1-213         -----------SSSSSSSSSSSSSSSPSEPD--------------------------------RKRASATDDDPDNRLGSTAT------------------
LOC_Os05g09480.1|11682.m00921/1-229                   ---------FSSSGLQGNTSTAADGAKGNDG------------------------------------------------------------------------
LOC_Os05g14180.1|11682.m01308/1-258                   PDRSPPAATLDLLPAAKGAKRGFSDEARPLPASAAAAAAA--------------------------GKGKKAAAGEEDEDAEEEDK--------------KVAA
LOC_Os05g44810.1|11682.m04283/1-328                   IGCFPTHSKPTTSIGTTGAKRGFFAIVGATLEGYNQSHRDTEECGKELTLGDE----------------NMAGERKKGCCPSPPCSAAA----------------HSS
LOC_Os05g48590.1|11682.m04652/1-282                   VVVDAALTLGPAPPPRGGAKRGFVDSLDRSEGRRAA---------------------------ATAGDDERGVREEEEEEKGL--------------GEAA
LOC_Os06g07040.1|11680.m00651/1-184                   ----------------CRKRGFEEALGGF--------------------------------KTDDDNDDGNGRGGDGDSDG------------------
LOC_Os06g22870.1|11680.m02247/1-267                   GGGTDAAPLTLELLPKGGAKRGFADAIVGGPA------------------------------GQRREAAGGKAAAAAAEAEE-------------EEEKK
LOC_Os06g24850.1|11680.m02452/1-150                   -----------------------------------------------------------------------------------------------------------
LOC_Os06g39590.1|11680.m03858/1-194                   ---------SSSSSSTADPERKRAAHADHADA-------------------------------------------------------------------------
LOC_Os07g08460.1|11673.m00782_altSplice/1-220         ----SITTTTTTGGADPAAKRSLGAKRSLEST----------------------------------DSMASGTGTSAAGDEHDDDT------------------
LOC_Os08g01780.1|11674.m00085/1-247                   ---------NDQISGGGAASPWLGVGVHPWS------------------------------LAARQGKAALEQAHQRPNEC----------------AVQ
LOC_Os11g11410.1|11687.m01091/1-144                   -----------------------------------------------------------------------------------------------------------
LOC_Os11g11420.1|11687.m01092/1-163                   -----------------------------------------------------------------------------------------------------------
LOC_Os11g11430.1|11687.m01093/1-172                   -----------------------------------------------------------------------------------------------------------
LOC_Os12g40900.1|11686.m04059/1-198                   ----EEEAAPPPSTPRAGSKRALAGEPDQAKI-----------------------------------------------------------------------
LOC_Os12g40890.1|11686.m04058/1-278                   ---GGDGDAAAAAARSSSGKRGFAETIDLKLKLEPAAAAVDDDDDKEEAAADDRE-----------KKVDIVGADNDDASPPAAAAAGGMKRSPSQSSVVTAAAD
LOC_Os03g43400.1|11669.m04312/1-234                   ---------GELAARSSGKRGFAETIDLKLKLQPAAPAAVSG------------------------EEGAQEDKEDADAAAAAADEKMSMKRSASQSSVVTAEPD
LOC_Os03g43410.1|11669.m04313/1-227                   --------ADDLPSTPRGKKRAAAAEDNNAN----------------------------------AAAADDDEHDAVEAA------------------
LOC_Os03g53150.1|11669.m05378_altSplice/1-237         ------GGGAAEAAAKAAKRGFEETIDLKLKLPTAGMEEAA-------------------------AGKAEAPAAEKAKRPAEAAA-----------------AD
LOC_Os03g58350.1|11669.m05916/1-198                   --------GGGDGVAAKKRRSASSTVKSEAS-----------------------------------GTACCGGAGARDVED------------------
LOC_Os09g35870.1|11681.m03177/1-141                   -----------------------------------------------------------------------------------------------------------
PoptrIAA3.6/1-192                                     ----SCTNEEQAVSGARNNKRPLPETREERGA---------------------------------KGKSDPRHDDQET------------------
PoptrIAA11/1-290                                      SGFKDFGQRSSQQYARILTAKDLPSKVSSSSCCSSTTSSSSST-----------------------LSRANATAGTKRAADSVSAS------------------
PoptrIAA33.1/1-149                                    -------------------------------------------------------------MAKPITFPGFEDDDVVSTMI------------------
PoptrIAA33.2/1-117                                    -----------------------------------------------------------------------------------------------------------
PoptrIAA28.1/1-239                                    -----ISLVYKTNPTTHGAKRVLEHTVGAKPS-------------------------------------EGYWFTDTDEKQYKRAA------------------
PoptrIAA3.4/1-199                                     ----DEPEKPSTTPSVRSNKRASPEISEE----------------------------------SRSKGSSSVSSNVENGERDS------------------
PoptrIAA29.2/1-233                                    -----------STNANDKKRSFIDAFEESSGNEDGPQTLP----------------------LLVWNNQPNDEDDFPKDLDN------------HSSNSCA
PoptrIAA12.2/1-258                                    ------------SGGRGGGGGKGKANARGERGR-------------------------------ILTAKDFPSVRAADSVSHEG------------------
PoptrIAA3.2/1-203                                     -ATKQSEKQTPNSNLAKSNKRSLPDMNEEPAGS------------------------------SRENSSTVSSNDKKSHDQET------------------
PoptrIAA20.2/1-175                                    -----------------TSQQENPST-------------------------------------------------------------------------------
PoptrIAA7.2/1-248                                     ----VGAKNEVETPNKATGKRGFAETVDLKLNLQAKEGVMD--------------------------LNENIKNITSKDKNHLPAVT-----------------IK
PoptrIAA34/1-194                                      ------------HSTYYQPREDDGIIDLGLS------------------------------------LRTLKPEAYHPSGHLVGLEG------------------
PoptrIAA3.3/1-199                                     ----DEPEKPSTTPSVRSNKRASPEISEES---------------------------------RSKGSSSLSSNVENSEGDDA------------------
PoptrIAA3.1/1-201                                     -ATEQLEKQTPNSNVTKSNKRSLPDMNEDSAG-------------------------------RRESSSVSSNDKKSHEQET------------------
PoptrIAA27.1/1-307                                    LGFSLNNNNSKDKSFVSGARRGFSVAIHGGSANWVFSGNAG-------------------------SDPNFSLRGANSGKEGFPHSSKPVVQENKSQVDGANTNG
PoptrIAA7.1/1-267                                     ----VGVKNEVETPNKATGKRGFAETVDLKLNLQAKEGVMD--------------------------LNENIKNIASKDKNHLPADT-----------------IK
PoptrIAA29.1/1-229                                    ---------SCEDGNYLDNKRSFEVAFEKNIKDASQELP-------------------------LLLWSGQPNDEDDWNGEKKI-----------------SRS
PoptrIAA27.2/1-349                                    TNNTNGYPLRPLKNLVSGTKRGFSDAIVGSSGKWVFSGSNGSEVDLGKGAILFSPR-----------GDNGNSQKSCVAGPAKKDDVAQSPKPVQEKISQVAANE
PoptrIAA16.4/1-237                                    ---------GGGAEGEMVRKRGFSETVDLKLKLS--------------------------------SKESGADPNHEKTSSLQREK--------------NLLAT
PoptrIAA9/1-366                                       HPSNDGHYSSTQKNVVSGNKRVFSDAMDEFSESKFLSNSEVNAMLSPRPSPNMGLKPGMLENLGVQQAKVKEIVAPKAGQERPHAANE--------TRPLRNSSAN
PoptrIAA19.3/1-184                                    ---------DDGHKNDKKRVFSEVSGEANS----------------------------------------------------------------------TT
PoptrIAA28.2/1-332                                    TTHGAKRVLQHPVGAKTSEGNWFTDTNVKQCKKFSCYQEEAEKVFSSPWLSSSLHSSSFHREAKKELQKPKPSFLQCSKVEELQYPDKMACSTPASASFSVTTDGAN
PoptrIAA12.1/1-276                                    --------------SGGGGKGKANAWGECGR-----------------------------------ILTAKDFPSVRAADSVSHEG------------------
PoptrIAA16.3/1-229                                    -------GGGGTEGGEFARKRGFSETVDLKLN----------------------------------LSSKEGGIDPNHEKTQREKN--------------LLAT
PoptrIAA16.1/1-250                                    -----ASNGNDGEAAKGNGKRGFSETVDLKLNL----------------------------------STKETGKDGSDQEKVVMKEK-----------TVAPRPN
PoptrIAA26.2/1-249                                    -----------DWSLKNTSNRERHESQLNSFG-----------------------------------YFTNGNQQTHKFPSSAENSH------------------
PoptrIAA27.3/1-335                                    TNNTNAYSLIPVKNLVSGAKRVFSDAIDGSTGKWVFSGGDNGNPQKSRV------------------AGPAKKDVAQSPKPVQEKNS------------QVAANE
PoptrIAA26.1/1-338                                    ERHKSQLLSFGYFTNGNQQTHKFPSSAENSHVWFNKKQQGKVPPSYLNFPSSSTPPTATATQQSLP--VMDKESSQPCCTKVVVELPQCAEKKAFSTPAPANTAVPN
PoptrIAA16.2/1-246                                    -----IGNGNDGEVAKSNGKRGFSETVDLKLN-----------------------------------LSTKESGKGGDEEKVMKEKT-----------VAPPAST
PoptrIAA3.5/1-207                                     ----GCTNEKGVSGARNNKRPFPETREEGGA---------------------------------NGKSDAQHDDQETASAPNTY----------------S
PoptrIAA19.2/1-189                                    -----------ENGPKNEKKRVFSELSGEANS----------------------------------------------------------------------TT
PoptrIAA15/1-220                                      -------SRGLALIEKTSGKRGFLETVDLNLGRSSN-------------------------------VDSDHNKYSGESETDVPNTA------------------
PoptrIAA29.3/1-233                                    -----------STNDNDKKRSFIDASEESSRNEDVPRTLP----------------------LLVWNNQPNEEDDPPKDLDN------------HCNYSFS
PoptrIAA20.1/1-174                                    -----------------TSRQDNPST-------------------------------------------------------------------------------
PoptrIAA19.1/1-189                                    -----------ENGPKNEKKRVFSELSGEANS----------------------------------------------------------------------TT
IAA1/1-168                                            ----QEEQQLELSCVRSNNKRKNNDSTEES-------------------------------------------------------------------------
IAA2/1-174                                            --TEKIKEEQEVSCVKSNNKRLFEETRDEEES--------------------------------------------------------------------
IAA3/1-189                                            --DNVCEAKERVSCCNNNNKRVLSTDTEKEIE-----------------------------------------SSSRKTET------------------
IAA4/1-186                                            ---------EETVSCGKSNKRVLPEATEKEIE---------------------------------------STGKTETAS------------------
IAA5/1-163                                            ---------IVVSGESISGKKRASPEVEIDLK-------------------------------------------------------------------------
IAA6/1-189                                            -----NYSEISVCGSSKKKKRVLSDMMT--------------------------------------SSALDTENENSVVSSVEDES------------------
IAA7/1-243                                            -----AEAVESPAKSAVGSKRGFSETVDLMLNL----------------------------------QSNKEGSVDLKNVSAVPKEK--------------TTLK
IAA8/1-321                                            PSKDNGSATTGHKNVVSGNKRGFADTWDEFSGVKGSVRPGGGINMMLS--------------------PKVKDVSKSIQEERSHAKGG------------------
IAA9/1-338                                            LPSKDEICSSSQKNNASGNKRGFSDTMDQFAEAKSSVYTEKNW-------------------------MFPEAAATQSVTKKDVPQNI--------PKGQSSTTNN
IAA10/1-261                                           ------------------RRKVRSSLSSSSSS--------------------------------LTRESGTKRSADSSPAAASN------------------
IAA11/1-246                                           ----------------LGRKGYRDCRVYADD----------------------------------SSSSSSSSSLSRASVIAGIK-----------------RT
IAA12/1-173                                           AWKERGRILTAKDFPSVGSKRSAESSSHQGAS--------------------------------------------------------------------
IAA13/1-246                                           -----------GGTAAKIGKSGGGGAWGERGR----------------------------------LLTAKDFPSVGSKRAADSAS------------------H
IAA14/1-228                                           -----TETVESPAKSGVGNKRGFSETVDLKLNLQSNKQ-----------------------------GHVDLNTNGAPKEKTFLKDP------------------
IAA15/1-179                                           ------PTNASEGPKKFGNKRRFLETVDLKLG------------------------------------------EAHENNYISSM------------------
IAA16/1-236                                           -------NHGGEMAGKNNGKRGFSETVDLKLNL----------------------------------SSTAMDSVSKVDLENMKEKV------------------
IAA17/1-229                                           ----------DTVAPVTGNKRGFSETVDLKLNLNNEPANKEGS------------------------TTHDVVTFDSKEKSACPKDP------------------
IAA18/1-267                                           --------EDDDESMIRHMKKEPKDKSILSLA--------------------------------------GKYFSPSSTKTTSHK------------------
IAA19/1-197                                           -----------DVAEKMMKKRAFTEMNMTSSG-----------------------------------SNSDQCESGVVSSGGDAEKV------------------
IAA20/1-175                                           --------------TSSGTQYFNGGYGYS------------------------------------------------------------------------
IAA26/1-269                                           -----DEEDHSAIKKKNTEIRNIKKETEDKSFH------------------------------CFNGNHFSPSNKTTSVPHIS------------------
IAA27/1-305                                           RVDSRFLALNKSSCPVSGAKRVFSDAINDSNKWVFSPG---------------------------STTATGDVGSGSGPRTSVVKDGKSTTFTKPAVPVKEKKS
IAA28/1-175                                           --------QFTSNNNINGSKQKSSTKETSFLS---------------------------------------------------------------------
IAA29/1-251                                           -----------ASCLGTEKLRFEATFGLGNVEENCYMPKQR------------------------LFALNGQPNEEDEDPLESES-----------------SI
IAA30/1-172                                           ---------------SSSGQYYN----------------------------------------------------------------------------------
IAA31/1-158                                           -----------------------------------------------------------------------------------------------------------
IAA32/1-143                                           ---------SKFHTYYSQTKKGGGVIDLGLS------------------------------------LRTIQHETYLPPARMIGLDG------------------
IAA33/1-171                                           ---------------QDSLQRRFHQDNSTTQQ----------------------------------------------------------------------
IAA34/1-185                                           ---------PKYHPYYSQTTEFGGVIDLGLS------------------------------------LRTIQHEIYHSSGQRYCSNE------------------
```

Consensus

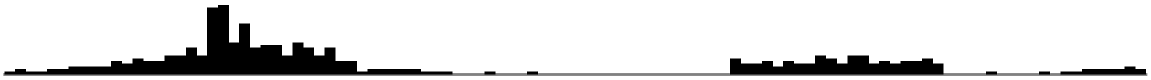

```
TA++DGEAL+SESS+KSGNKRGFSETVDLSLSL+SASGSE+++++K++-AS++-P------------LSTASGSPGSEDEKPVEAEAA+S+K++S+P+SVVAAAAT
```

```
                                                 220        230        240        250        260        270        280        290        300        310
LOC_Os01g08320.1|11667.m00781/1-208              -DAPPAPKARVVGWPPVR------SFRKNALA---------------------------------------AKFVKVAVDGAPYLRKVDLEAYSGYDQLL
LOC_Os01g09450.1|11667.m00901_altSplice/1-237    ------AAPVVGWPPVR------SFRRNLAS---------SSSSSSKQAPPPPSSSPQNGDKASKDGGAEK----GMFVKINMDGVPIGRKVDLAAYGGYAQLS
LOC_Os01g13030.1|11667.m01279/1-267              APPQPAAKAQVVGWPPVR------SYRKNTMA--------------------TNQIKSNKEDVDAKQGQG----FLYVKVSMDGAPYLRKVDLKTYKNYKDMS
LOC_Os01g18360.1|11667.m01799/1-204              --STPRNSQVHADWPPIK------PFLRSALQ-------------------------KASAAGGGGARRRR----TLFVKVYMEGVPIGRKLDLLLLDGYDSLL
LOC_Os01g48450.1|11667.m04699/1-272              ---PPAAKAQVVGWPPVR------NYRKNTLA---------------------ASASKGKGEDKGTAEGG----PLYVKVSMDGAPYLRKVDLKMYSSYEDLS
LOC_Os01g53880.1|11667.m05292/1-262              QTQGRGAAAPVVGWPPIR------SFRRNLAS---------------SSSSKHSPEPQNDNANAKVTLTCKK----NPLVKINMDGIPIGRKIDLAAYNSYDGLS
LOC_Os02g13520.1|11668.m01248_altSplice/1-308    CFQTRSPSTPVVGWPPIR------TFRRNLAT-----------------SSKASLELQNGKKAAKAEEIKR----APFIKINMDGVPIGRKIDLNAFDSYEKLS
LOC_Os02g49160.1|11668.m04779/1-206              --LTLSSVVHIDGNNP--------STPRSSLT-------------------TATVTADRGGGGGGHGRRR----SLFVKVYMEGVPIGRKLDLLPLDGYKGLV
LOC_Os02g56120.1|11668.m05537/1-183              ----RRRKKTVVGWPPVS------SARRACGG---------------------------------------ANYVKVKKEGDAIGRKVDLALHSSYDELA
LOC_Os02g57250.1|11668.m05658/1-282              ASGHPHSSFGVVGWPPIR------QFRMNSLF-----NQAKENTSETDTKKTATNESDVQKDKEEGEKKGRV----AGWVKVNMDGEVIGRKVDLNAHRSYKTLA
LOC_Os05g08570.1|11682.m00824_altSplice/1-213    -ESPPSPKARVVGWPPVR------AFRKNALA-----------------------------ALAAASSSK----AKFVKVAVDGAPYLRKVDLEAYRGYDQLL
LOC_Os05g09480.1|11682.m00921/1-229              -FKASRPAAPVVGWPPVR------SFRRNLAS-----------------SSSSSKPPRGGRDAAAAATGGKV----ARFVKVNMDGVPIGRKVDLAAHGGYGELS
LOC_Os05g14180.1|11682.m01308/1-258              APQAPAAKAQVVGWPPVR------SYRKNTMA--------------------TNQLKSSKEDAEAKQGQG----FLYVKVSMDGAPYLRKVDLKTYKNYKELS
LOC_Os05g44810.1|11682.m04283/1-328              NPQGRGAIPPVVGWPPIR------SFRRNLTN-------------GSSFKQSPERQNDEADDKAKPICKK----RPLVKINMDGIPIGRKVDLQIYDSYQKLS
LOC_Os05g48590.1|11682.m04652/1-282              AGAPRAAKAQVVGWPPVR------SYRKNTLA---------------------ASATKTKGEDQGKSEVG----CCYVKVSMDGAPYLRKVDLKTYSSYEDLS
LOC_Os06g07040.1|11680.m00651/1-184              --EMGNKRRKLVGWPPVK------CLHRRRDG---------------------------------GCG----GGYVKVKMEGLAIGRKLDLSILGSYAELL
LOC_Os06g22870.1|11680.m02247/1-267              KAQAPAAKAQVVGWPPVR------SYRKNTMA----------------MSQPALKGKDDGEAKQAPASG----CLYVKVSMDGAPYLRKVDLKMYKNYKELS
LOC_Os06g24850.1|11680.m02452/1-150              --NRAASSAQLVGWPPVR------TFRKNLST------PKPADADDLMNKMKPCSDEGHGSRDAAQERRPSS----TMFVKVNLEGYAVGRKIDLKAHRSYDSLS
LOC_Os06g39590.1|11680.m03858/1-194              --KPPSPKARAVGWPPVR------AYRRNALR---------------------------------EDSAR----AKLVKVAVDGAPYLRKVDLAAHAGYAPLL
LOC_Os07g08460.1|11673.m00782_altSplice/1-220    ---AAPAKAQVVGWPPVR------AYRRNTFH----------------QAAAAAAATKKGGDEKQKQQQQGG----GLYVKVSMDGAPYLRKVDLKMCKGYRELR
LOC_Os08g01780.1|11674.m00085/1-247              RENRAASSAQLVGWPPVR------AFRKNLST------PKPADADDLMNKVKLCSDEGHGSRCAAQERRSSS----TMFVKVNLEGYAVGRKIDLKAHRSYDSLS
LOC_Os11g11410.1|11687.m01091/1-144              ------AADETTAPPPR-----------------------------------------SAAAATEASRTL----NMFAKVHMDGYKVGRKINLRAHRNYDSLR
LOC_Os11g11420.1|11687.m01092/1-163              ------PEVKPAGLSPSR-----------------------------------------------------FVKVFMHGEPFERKINLAIHNNYDSLS
LOC_Os11g11430.1|11687.m01093/1-172              --NASAEPVVKPGLSPSR-----------------------------------------------------FVKVFMHGEPFGRKINLALHNNYDSLS
LOC_Os12g40900.1|11686.m04059/1-198              -KPAAAAKAQVVGWPPVR------SYRKSCLQ-----------PTTTTTKSKPPPAAAAAETQQKEDVAGAG----GLFVKVSMDGAPYLRKIDLKVYKGYRELR
LOC_Os12g40890.1|11686.m04058/1-278              PEKPAPAKAQVVGWPPVR------SYRKNILA-----------------VQADKGKDAADGGGDKSGAGAAA----AAFVKVSMDGAPYLRKVDLKMYKSYLELS
LOC_Os03g43400.1|11669.m04312/1-234              PDKPRAPKAQVVGWPPVR------SYRKNVLA-------------------------------EKCKA----AALVKVSMDGAPYLRKIDVAMYKSYPELS
LOC_Os03g43410.1|11669.m04313/1-227              ---PPVAKAQVVGWPPVR------SYRKSCFQQQSAAASKSKAAVSSCNNKDEPITKNAAPAPAASSAAAANG----GSLVKVSMDGAPYLRKIDLRMYKGYRELR
LOC_Os03g53150.1|11669.m05378_altSplice/1-237    AEKPPAPKAQAVGWPPVR------SFRRNIMT-----------VQSVKSKKEEADKQQQQPAANASGSNS----SAFVKVSMDGAPYLRKVDLKMYNSYKDLS
LOC_Os03g58350.1|11669.m05916/1-198              -GASPASKVQVVGWPPVG------SYRRSTFQ-------------SSSSSTAAAAKGKGGGETDQGRKNKGG----GLYVKVSMDGAPYLRKVDLRMYGGYRELR
LOC_Os09g35870.1|11681.m03177/1-141              -SARRARRGRKNGHPPPS------SSMIQA-----------------------------------------AYFVKVSMDGTPYLRKVDVAAYGDYLELV
PoptrIAA3.6/1-192                                ---APAPKAQIVGWPPIR------SYRKNTLQ-------------------------PKKAEAEAAA----GMYVKVSMDGAPYLRKIDLKVYKGYPELL
PoptrIAA11/1-290                                 ---NGAASSQVVGWPPIR------SHRMHIMV--NQAKSQATEEFNSMNKRKNAVEEKVGNKNINIGNTKTRT----SLFVKVNMDGTLIGRKVDLNAHGCYETLA
PoptrIAA33.1/1-149                               -------------PPVT------------------------------------------------------------VALEGRSICQRISLHKHESYHSLA
PoptrIAA33.2/1-117                               ------------IPPVT------------------------------------------------------------VVLEGRSICQRISLHKHASYHSLA
PoptrIAA28.1/1-239                               ------LAPVVGWPPIR------SFRKNLAG-----------SSTPKLVSESRNKPPKEGSSLKPDSFRN----DLFVKINMEGVPIGRKINLNAYDSYEKLS
PoptrIAA3.4/1-199                                ---APPAKAQVVGWPPIR------SYRKNCLQ-------------------------PKKNDQVDGA----GMYVKVSVDGAPYLRKIDLKVYKSYPELL
PoptrIAA29.2/1-233                               SNKSDGESDWIVGWPPIK-------FKKKKLS-------------RQSSRALEINRAVDNGYEDCQARTSK----YMYIKVKMEGVGIARKIDVSLHHSFPTLK
PoptrIAA12.2/1-258                               --GSPTAGSQVVGWPPIR------AYRMNSLVNQAKAARAEEDKGIGEKDISKDNLKKKICNGNKTSAPSNEKGH--LGFVKVNMDGIPIGRKVDLNAHACYETLA
PoptrIAA3.2/1-203                                ---APPIKAQVVGWPPIR------SYRKNCLQ-------------------------AKKLEAEAA----GLYVKVSMDGAPYLRKIDLKVYKGYPELL
PoptrIAA20.2/1-175                               -----PSDQQLSDWPPIK------PFLRKALA-------------------------SEENECSSA----TFFVKVYMEGIPIGRKLNLLAHDGYHDLI
PoptrIAA7.2/1-248                                DPAKPPAKAQVVGWPPVR------SYRKNVMA--------------------QKNASEEGEKASTGGSS----AAFVKVCMDGAPYLRKVDLKMYRSYQELS
PoptrIAA34/1-194                                 ------YGDLMDWPRAN------SPLKHSTS-----------------SYIRFTPQDCDEEAEGVQSKDR----WAYVKVNMDGVIVGRKICMLDHGGYSSLA
PoptrIAA3.3/1-199                                ---PPAKAQVVGWPPIR------SYRKNCLQ-------------------------PKKNDRVDGA----GMYVKVSVDGAPYLRKIDLKVYRSYPELL
PoptrIAA3.1/1-201                                ---APPTKTQVVGWPPIR------SYRKNCLQ-------------------------ARKLEAEAA----GLYVKVSMDGAPYLRKIDLKVYKGYPELL
PoptrIAA27.1/1-307                               HGAAPASKAQVVGWPPIR------SFRKNTMA--------------------SHLSKNDDGAEVKSGSG----CLYVKVSMDGAPYLRKVDLKTFGSYMELS
PoptrIAA7.1/1-267                                DPAKPPAKAQVVGWPPVR------SYRKNVLAQKNASEEGFRAQVVGWPPLRSYRKNVLTQKNASEEGDKASTGGSSAAFVKVCMDGAPYLRKVDLKMYKSYQELS
PoptrIAA29.1/1-229                               INKDDEEENQVVGWPPIK------SWRKKVLH-----------HQHQAGHVVNSTRMATAGNYEYGTGSN----SKYVKVKMEGVAITRKIDLRLYNSYQTLT
PoptrIAA27.2/1-349                               NSSAPAAKAQVVGWPPIR------SFRKNTMA--------------------SSLVKNNEDVEGKSGYG----CLYVKVSMDGAPYLRKVDLKTYSNYLELS
PoptrIAA16.4/1-237                               DPAKPPAKAQVVGWPPVR------SFRKNMLA--------------------VQKSSTDQECEKVPGGN----ATFVKVSMDGAPYLRKVDLKMYKTYQELS
PoptrIAA9/1-366                                  NSSAPAPKAQVVGWPPIK------SFRKNSLA----------------------TTSKNTEEVDGKAGPG----ALFIKVSMDGAPYLRKVDLRNYSAYQELS
PoptrIAA19.3/1-184                               DDRKVQTKSQVVGWPPVC------SYRKNIS--------------------------NERDRHHETS----KIYVKVSMDGAPFLRKVDLGMHKEYSDLV
PoptrIAA28.2/1-332                               SCHKRCALATVVGWPPIR------SFRKNIAS-----------SSTSKMVSELPNKTSEEGSSLKPDSFRN----DLFVKINMEGVPIGRKINLNAYDSYEKLS
PoptrIAA12.1/1-276                               --GSPTAGSQVVGWPPIR------AYRMNSLVSQAKAARAEEEKGIGEKDKSKENLKKKICNGNKTNATGNEKGH--LGFVKVNMDGVPIGRKVDLNAHACYETLA
PoptrIAA16.3/1-229                               DPAKPPAKAQVVGWPPVR------SFRKNMLA--------------------VQKSSTDQESTDKVPGGN----ATFVKVSMDGAPYLRKVDLKMYKTYHELS
PoptrIAA16.1/1-250                               DPAKPPSKAQVVGWPPVR------SFRKNVMA------------------VQKNSNDEGEKASSSGTTGT----AAFVKVSMDGAPYLRKVDLKLYKSYQELS
PoptrIAA26.2/1-249                               ---VXTAPGPVVGWPPIR------SFRKNLAS-----------------SSGSYSKPTVESQNKPVETCKK----GLFVKINMEGVPIGRKVDLKAYDTYEKLS
PoptrIAA27.3/1-335                               NSSAPAAKTQVVGWPPIR------SFRKNTMA--------------------SSLAKNNEDVDGKSGYG----YLYVKVSMDGAPYLRKVDLKTYGNYLELS
PoptrIAA26.1/1-338                               SSQKRIAPGPVVGWPPIR------SFRKNLAT-----------------SSGSNSKPTFESQNKPAGTCKK----GLFVKINMEGVPIGRKVDLKAYDSYEKLS
PoptrIAA16.2/1-246                               DPAKPPAKAQVVGWPPIR------SFRKNVMA--------------------VQKNSNDNGEKSGSSGTG----VAFVKVSMDGAPYLRKVDLKLYKSYQELS
PoptrIAA3.5/1-207                                FDMHATCRVQIVGWPPIR------SYRKNSLQ-------------------------PKKAEDEAAA----GMYVKVSMDGAPYLRKIDLKVYKGYPELL
PoptrIAA19.2/1-189                               DGRKTQTTSQVVGWPPVC------SYRKKNSF-------------------------NEKDSHETS----KIYVKVSMDGAPFLRKVDLGMHKEYSDLV
PoptrIAA15/1-220                                 --KPPAAKAQVVGWPPVR------AYRKNAMK---------------------------------------S----CKYVKVAVDGAPYLRKVDLEMYNSYQQLL
PoptrIAA29.3/1-233                               SNKSDGESDGIVGWPPIK-------FKRKKLS-------------RQNSRVLEVNRAVDNGCEDCQARSSN----SMYIKVKMEGVGIARKIDVSVYRCFPTLK
PoptrIAA20.1/1-174                               -----PSEQLLDWPPIK------PSPGKAVT-------------------------SEENECCSS----TFFVKVYMEGIQIGRKLNLLAHDGYHDLI
PoptrIAA19.1/1-189                               DGRKTQTTSQVVGWPPVC------SYRKKNSF-------------------------NEKDSHETS----KIYVKVSMDGAPFLRKVDLGMHKEYSDLV
IAA1/1-168                                       --APPPAKTQIVGWPPVR------SNRKNNNN------------------------------------KN----VSYVKVSMDGAPYLRKIDLKMYKNYPELL
IAA2/1-174                                       ---TPPTKTQIVGWPPVR------SSRKNNNS---------------------------------------VSYVKVSMDGAPYLRKIDLKTYKNYPELL
IAA3/1-189                                       ---SPPRKAQIVGWPPVR------SYRKNNIQ-------------------------SKKNESEHEGQ----GIYVKVSMDGAPYLRKIDLSCYKGYSELL
IAA4/1-186                                       -----PPKAQIVGWPPVR------SYRKNNVQ-------------------------TKKSESEGQ----GNYVKVSMDGAPYLRKIDLTMYKQYPELM
IAA5/1-163                                       ---CEPAKKSQVVGWPPVC------SYRRKNSL-------------------------------ERTK----SSYVKVSVDGAAFLRKIDLEMYKCYQDLA
IAA6/1-189                                       ---LPVVKSQAVGWPPVC------SYRRKKNN-------------------------EEASKA----IGYVKVSMDGVPYMRKIDLGSSNSYINLV
IAA7/1-243                                       DPSKPPAKAQVVGWPPVR------NYRKNMMT--------------QQKTSSGAEEASSEKAGNFGGGAAG----AGLVKVSMDGAPYLRKVDLKMYKSYQDLS
IAA8/1-321                                       LNNAPAAKAQVVGWPPIR------SYRKNTMA--------------------SSTSKNTDEVDGKPGLG----VLFVKVSMDGAPYLRKVDLRTYTSYQQLS
IAA9/1-338                                       SSSPPAAKAQIVGWPPVR------SYRKNTLA--------------------TTCKNSDEVDGRPGSG----ALFVKVSMDGAPYLRKVDLRSYTNYGELS
IAA10/1-261                                      ----ATRQVAVGWPPLR------TYRINSLVNQAKSLATEGGLSSGIQKETTKSVVVAAKNDDACFIKSSRT----SMLVKVTMDGVIIGRKVDLNALDSYAALE
IAA11/1-246                                      ADSMAATSGQVVGWPPIR------TYRMNSMV----------NQAKASATEDPNLEISQAVNKNRSDSTKMRN----SMFVKVTMDGIPIGRKIDLNAHKCYESLS
IAA12/1-173                                      ---PPRSSQVVGWPPIG------LHRMNSLV---NNQAMKAARAEEGDGEKKVVKNDELKDVSMKVNPKVQG----LGFVKVNMDGVGIGRKVDMRAHSSYENLA
IAA13/1-246                                      AGSSPPRSSQVVGWPPIG------SHRMNSLV---------NNQATKSAREEEEAGKKKVKDDEPKDVTKKVNGKVQGFIKVNMDGVAIGRKVDLNAHSSYENLA
IAA14/1-228                                      --SKPPAKAQVVGWPPVR------NYRKNVMA--------------------NQKSGEAEEAMSSGGGT----VAFVKVSMDGAPYLRKVDLKMYTSYKDLS
IAA15/1-179                                      -----VTNDQLVGWPPVA------TARKTVRR-----------------------------------------KYVKVALDGAAYLRKVDLGMYDCYGQLF
IAA16/1-236                                      --VKPPAKAQVVGWPPVR------SFRKNVMS---------GQKPTTGDATEGNDKTSGSSGATSSASACAT----VAYVKVSMDGAPYLRKIDLKLYKTYQDLS
IAA17/1-229                                      --AKPPAKAQVVGWPPVR------SYRKNVMV-------------------------SCQKSSGGPEA----AAFVKVSMDGAPYLRKIDLRMYKSYDELS
IAA18/1-267                                      ---RTAPGPVVGWPPVR------SFRKNLAS----GSSSKLGNDSTTSNGVTLKNQKCDAAAKTTEPKRQG----GMFVKINMYGVPIGRKVDLSAHNSYEQLS
IAA19/1-197                                      -NDSPAAKSQVVGWPPVC------SYRKKNSC-------------------------KEASTTKVG----LGYVKVSMDGVPYLRKMDLGSSQGYDDLA
IAA20/1-175                                      ----------VAAPAVE------DAEYVAAV-------------------------EEEEENECNSVG----SFYVKVNMEGVPIGRKIDLMSLNGYRDLI
IAA26/1-269                                      --QKRTAPGPVVGWPPVR------SFRKNLAS-----------TSSSKLGNESSHGGQINKSDDGEKQVETKKE----GMFVKINMDGVPIGRKVDLNAYNSYEQLS
IAA27/1-305                                      SATAPASKAQVVGWPPIR------SFRKNSMA------------------SSQSQKPGNNSETEEAEAKSGPEQPCLYVKVSMEGAPYLRKIDLKTYKSYLELS
IAA28/1-175                                      --NNRVEVAPVVGWPPVR------SSRRNLTA-----------------------QLKEEMKKKESDEEK----ELYVKINMEGVPIGRKVNLSAYNNYQQLS
IAA29/1-251                                      VYDDEEENSEVVGWPPVKTCMIKYGSYHHRHIRNHHHCPYHHRGRRITAMNNNISNPTTATVGSSSSSISSRS----SMYVKVKMDGVAIARKVDIKLFNSYESLT
IAA30/1-172                                      -------GGDNHEYDGVG------AAEEMMIM-------------------------EEEEQNECNSVG----SFYVKVNMEGVPIGRKIDLLSLNGYHDLI
IAA31/1-158                                      ----SPQREARQDWPPIK------SRLRDTLK-------------------------GRRLLRRGDDT----SLFVKVYMEGVPIGRKLDLCVFSGYESLL
IAA32/1-143                                      -------YGELIDWSQPS------YNSITQLK-----------------SEDTGHQRLAQGYYNNEGESRGK----YAYVKVNLDGLVVGRKVCLVDQGAYATLA
IAA33/1-171                                      ---PRDTTTPFIPKPASK------NHNNSNSS-----------------SGAAGRSFQGFGLNVEDDLVSSV----VPPVTVVLEGRSICQRISLHKHGSYQSLA
IAA34/1-185                                      ----------------------GYRRK---------------------------------------------------WGYVKVTMDGLVVGRKVCVLDHGSYSTLA
```

Consensus

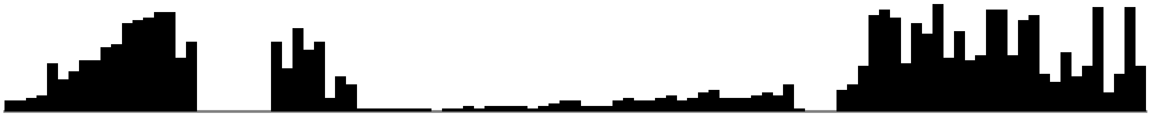

```
DPA+PAAKAQVVGWPPVR------SYRKNTLANQAKAAS++KAQASSTQNSSS+SPNK+SKN+EEKKGESGSGGH--AL+VKVSMDGAPYLRKVDLKAYKSYEELS
```

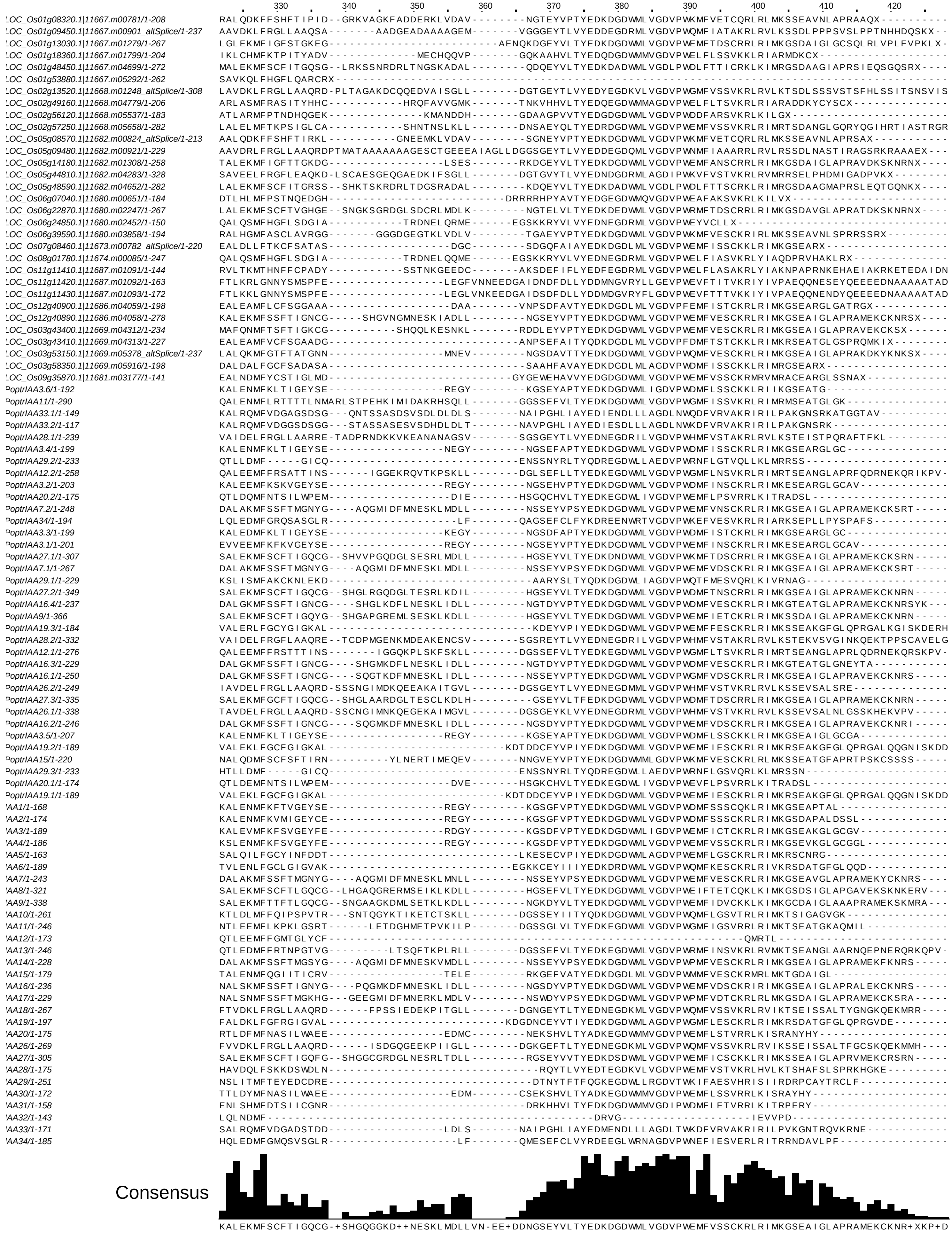

330 340 350 360 370 380 390 400 410 420
LOC_Os01g08320.1|11667.m00781/1-208 RALQDKFFSHFTIPID--GRKVAGKFADDERKLVDAV-------NGTEYVPTYEDKDGDWMLVGDVPWKMFVETCQRLRLMKSSEAVNLAPRAAQX---------
LOC_Os01g09450.1|11667.m00901_altSplice/1-237 AAVDKLFRGLLAAQSA------AADGEADAAAAGEM------VGGGEYTLVYEDDEGDRMLVGDVPWQMFIATAKRLRVLKSSDLPPPSVSLPTNHHDQSKX--
LOC_Os01g13030.1|11667.m01279/1-267 LGLEKMFIGFSTGKEG----------------------AENQKDGEYVLTYEDKDGDWMLVGDVPWEMFTDSCRRLRIMKGSDAIGLGCSQLRLVPLFVPKLX-
LOC_Os01g18360.1|11667.m01799/1-204 IKLCHMFKTPITYADV------------MECHQQVP------GQKAAHVLTYEDQDGDWMMVGDVPWELFLSSVKKLRIARMDKCX------------------
LOC_Os01g48450.1|11667.m04699/1-272 MALEKMFSCFITGQSG--LRKSSNRDRLTNGSKADAL-------QDQEYVLTYEDKDADWMLVGDLPWDLFTTICRKLKIMRGSDAAGIAPRSIEQSGQSRX----
LOC_Os01g53880.1|11667.m05292/1-262 SAVKQLFHGFLQARCRX--
LOC_Os02g13520.1|11668.m01248_altSplice/1-308 LAVDKLFRGLLAAQRD-PLTAGAKDCQQEDVAISGLL------DGTGEYTLVYEDYEGDKVLVGDVPWGMFVSSVKRLRVLKTSDLSSSVSTSFHLSSITSNSVIS
LOC_Os02g49160.1|11668.m04779/1-206 ARLASMFRASITYHHC----------HRQFAVVGMK------TNKVHHVLTYEDQEGDWMMAGDVPWELFLTSVKRLRIARADDKYCYSCX------------
LOC_Os02g56120.1|11668.m05537/1-183 ATLARMFPTNDHQGEK-------------KMANDDH------GDAAGPVVTYEDGDGDWMLVGDVPWDDFARSVKRLKILGX---------------------
LOC_Os02g57250.1|11668.m05658/1-282 LALELMFTKPSIGLCA----------SHNTNSLKLL------DNSAEYQLTYEDRDGDWMLVGDVPWEMFVSSVKRLRIMRTSDANGLGQRYQGIHRTIASTRGR
LOC_Os05g08570.1|11682.m00824_altSplice/1-213 AALQDKFFSHFTIRKL---------GNEEMKLVDAV-------SGNEYVPTYEDKDGDWMLVGDVPWKMFVETCQRLRIMKSSEAVNLAPRSAX----------
LOC_Os05g09480.1|11682.m00921/1-229 AAVDRLFRGLLAAQRDPTMATAAAAAAAGESCTGEEEAIAGLLDGGSGEYTLVYEDDEGDQMLVGDVPWNMFIAAARRLRVLRSSDLNASTIRAGSRKRAAAEX---
LOC_Os05g14180.1|11682.m01308/1-258 TALEKMFIGFTTGKDG---------------LSES------RKDGEYVLTYEDKDGDWMLVGDVPWEMFANSCRRLRIMKGSDAIGLAPRAVDKSKNRNX----
LOC_Os05g44810.1|11682.m04283/1-328 SAVEELFRGFLEAQKD-LSCAESGEQGAEDKIFSGLL------DGTGVYTLVYEDNDGDRMLAGDIPWKVFVSTVKRLRVMRRSELPHDMIGADPVKX--------
LOC_Os05g48590.1|11682.m04652/1-282 LALEKMFSCFITGRSS--SHKTSKRDRLTDGSRADAL-------KDQEYVLTYEDKDADWMLVGDLPWDLFTTSCRKLRIMRGSDAAGMAPRSLEQTGQNKX----
LOC_Os06g07040.1|11680.m00651/1-184 DTLHLMFPSTNQEDGH----------------------DRRRRHPYAVTYEDGEGDWMQVGDVPWEAFAKSVKRLKILVX----------------------
LOC_Os06g22870.1|11680.m02247/1-267 LALEKMFSCFTVGHGE--SNGKSGRDGLSDCRLMDLK-------NGTELVLTYEDKEDWMLVGDVPWRMFTDSCRRLRIMKGSDAVGLAPRATDKSKNRNX----
LOC_Os06g24850.1|11680.m02452/1-150 QALQSMFHGFLSDGIA----------TRDNELQRME-----EGSKKRYVLVYEDNEGDRMLVGDVPWEYVCLLX-----------------------------
LOC_Os06g39590.1|11680.m03858/1-194 RALHGMFASCLAVRGG------GGGDGEGTKLVDLV-------TGAEYVPTYEDKDGDWMLVGDVPWKMFVESCKRIRLMKSSEAVNLSPRRSSRX---------
LOC_Os07g08460.1|11673.m00782_altSplice/1-220 EALDLLFTKCFSATAS-----------------DGC------SDGQFAIAYEDKDGDLMLVGDVPWEMFISSCKKLRIMKGSEARX-----------------
LOC_Os08g01780.1|11674.m00085/1-247 QALQSMFHGFLSDGIA----------TRDNELQQME-----EGSKKRYVLVYEDNEGDRMLVGDVPWELFIASVKRLYIAQDPRVHAKLRX-------------
LOC_Os11g11410.1|11687.m01091/1-144 RVLTKMTHNFFCPADY----------SSTNKGEEDC------AKSDEFIFLYEDFEGDRMLVGDVPWELFLASAKRLYIAKNPAPRNKEHAEIAKRKETEDAIDN
LOC_Os11g11420.1|11687.m01092/1-163 FTLKRLGNNYSMSPFE----------------LEGFVNNEEDGAIDNDFDLLYDDMNGVRYLLGEVPWEVFTITVKRIYIVPAEQQNESEYQEEEDNAAAAATAD
LOC_Os11g11430.1|11687.m01093/1-172 FTLKKLGNNYSMSPFE----------------LEGLVNKEEDGAIDSDFDLLYDDMDGVRYFLGDVPWEVFTTTVKKIYIVPAEQQNENDYQEEEDNAAAAATAD
LOC_Os12g40900.1|11686.m04059/1-198 EALEAMFLCFSGGAAA-----------------DAA------VNPSDFAVTYEDKDGDLMLVGDVPFEMFISTCKRLRIMKGSEARGLGATRGX----------
LOC_Os12g40890.1|11686.m04058/1-278 KALEKMFSSFTIGNCG-----SHGVNGMNESKIADLL-------NGSEYVPTYEDKDGDWMLVGDVPWEMFVESCKRLRIMKGSEAIGLAPRAMEKCKNRSX----
LOC_Os03g43400.1|11669.m04312/1-234 MAFQNMFTSFTIGKCG-----------SHQQLKESNKL-------RDDLEYVPTYEDKDGDWMLVGDVPWEMFVESCKRLRIMKGSEAIGLAPRAVEKCKSX------
LOC_Os03g43410.1|11669.m04313/1-227 EALEAMFVCFSGAADG-----------------------------ANPSEFAITYQDKDGDLMLVGDVPFDMFTSTCKKLRIMKRSEATGLGSPRQMKIX--------
LOC_Os03g53150.1|11669.m05378_altSplice/1-237 LALQKMFGTFTATGNN----------------MNEV-------NGSDAVTYEDKDGDWMLVGDVPWQMFVESCKRLRIMKGSEAIGLAPRAKDKYKNKSX----
LOC_Os03g58350.1|11669.m05916/1-198 DALDALFGCFSADASA--------------------------SAAHFAVAYEDKDGDLMLAGDVPWDMFISSCKKLRIMRGSEARX-----------------
LOC_Os09g35870.1|11681.m03177/1-141 EALNDMFYCSTIGLMD-----------------------GYGEWEHAVVYEDGDGDWMLVGDVPWEMFVSSCKRMRVMRACEARGLSSNAX-----------
PoptrIAA3.6/1-192 KALENMFKLTIGEYSE----------------REGY-------KGSEYAPTYEDKDGDWMLIGDVPWDMFLSSCKKLRIIKGSEATG-----------------
PoptrIAA11/1-290 QALENMFLRTTTTLNMARLSTPEHKIMIDAKRHSQLL------GGSSEFVLTYEDKDGDWMLVGDVPWGMFISSVKRLRIMRMSEATGLGK--------------
PoptrIAA33.1/1-149 KALRQMFVDGAGSDSG---QNTSSASDSVSDLDLDLS------NAIPGHLIAYEDIENDLLLAGDLNWQDFVRVAKRIRILPAKGNSRKATGGTAV----------
PoptrIAA33.2/1-117 KALRQMFVDGGSDSGG---STASSASESVSDHDLDLT------NAVPGHLIAYEDIESDLLLAGDLNWKDFVRVAKRIRILPAKGNSRK----------------
PoptrIAA28.1/1-239 VAIDELFRGLLAARRE-TADPRNDKKVKEANANAGSV------SGSGEYTLVYEDNEGDRILVGDVPWHMFVSTAKRLRVLKSTEISTPQRAFTFKL---------
PoptrIAA3.4/1-199 KALENMFKLTIGEYSE----------------NEGY-------NGSEFAPTYEDKDGDWMLVGDVPWDMFISSCKRLRIMKGSEARGLGC--------------
PoptrIAA29.2/1-233 QTLLDMF-----GICQ-------------------------ENSSNYRLTYQDREGDWLLAEDVPWRNFLGTVQLLKLMRRSS--------------------
PoptrIAA12.2/1-258 QALEEMFFRSATTINS------IGGEKRQVTKPSKLL------DGLSEFLLTYEDKEGDWMLVGDVPWGMFLNSVKRLRIMRTSEANGLAPRFQDRNEKQRIKPV-
PoptrIAA3.2/1-203 KALEEMFKSKVGEYSE----------------REGY-------NGSEHVPTYEDKDGDWMLVGDVPWDMFINSCKRLRIMKESEARGLGCAV------------
PoptrIAA20.2/1-175 QTLDQMFNTSILWPEM-----------------DIE------HSGQCHVLTYEDKEGDWLIVGDVPWEMFLPSVRRLKITRADSL-------------------
PoptrIAA7.2/1-248 DALAKMFSSFTMGNYG----AQGMIDFMNESKLMDLL-------NSSEYVPSYEDKDGDWMLVGDVPWEMFVNSCKRLRIMKGSEAIGLAPRAMEKCKSRT-----
PoptrIAA34/1-194 LQLEDMFGRQSASGLR-----------------LF-------QAGSEFCLFYKDREENWRTVGDVPWKEFVESVKRLRIARKSEPLLPYSPAFS----------
PoptrIAA3.3/1-199 KALEDMFKLTIGEYSE----------------KEGY-------NGSDFAPTYEDKDGDWMLVGDVPWDMFISTCKRLRIMKGSEARGLGC--------------
PoptrIAA3.1/1-201 EVVEEMFKFKVGEYSE----------------REGY-------NGSEYVPTYEDKDGDWMLVGDVPWEMFINSCKRLRIMKESEARGLGCAV------------
PoptrIAA27.1/1-307 SALEKMFSCFTIGQCG--SHVVPGQDGLSESRLMDLL-------HGSEYVLTYEDKNDWMLVGDVPWDTFTDSCRRLRIMKGSEAIGLAPRAMEKCKSRN-----
PoptrIAA7.1/1-267 DALAKMFSSFTMGNYG----AQGMIDFMNESKLMDLL-------NSSEYVPSYEDKDGDWMLVGDVPWEMFVDSCKRLRIMKGSEAIGLAPRAMEKCKSRT-----
PoptrIAA29.1/1-229 KSLISMFAKCKNLEKD--------------------------AARYSLTYQDKDGDWLIAGDVPWQTFMESVQRLKIVRNAG-------------------
PoptrIAA27.2/1-349 SALEKMFSCFTIGQCG--SHGLRGQDGLTESRLKDIL-------HGSEYVLTYEDKDGDWMLVGDVPWDMFTNSCRRLRIMKGSEAIGLAPRAMEKCKNRN-----
PoptrIAA16.4/1-237 DALGKMFSSFTIGNCG----SHGLKDFLNESKLIDLL-------NGTDYVPTYEDKDGDWMLVGDVPWEMFVESCKRLRIMKGTEATGLAPRAMEKCKNRSYK---
PoptrIAA9/1-366 SALEKMFSCFTIGQYG--SHGAPGREMLSESKLKDLL-------HGSEYVLTYEDKDGDWMLVGDVPWEMFIETCKRLRIMKSSDAIGLAPRAMEKCKNRN-----
PoptrIAA19.3/1-184 VALERLFGCYGIGKAL--------------------------KDEYVPIYEDKDGDWMLVGDVPWEMFFESCKRLRIMKSSEAKGFGLQPRGALQQGNISKDERH
PoptrIAA28.2/1-332 VAIDELFRGFLAAQRE-TCDPMGENKMDEAKENCSV------SGSREYTLVYEDNEGDRILVGDVPWHMFVSTAKRLRVLKSTEKVSVGINKQEKTPPSCAVELG
PoptrIAA12.1/1-276 QALEEMFFRSTTTINS-------IGGQKPLSKFSKLL------DGSSEFVLTYEDKEGDWMLVGDVPWGMFLTSVKRLRIMRTSEANGLAPRLQDRNEKQRSKPV-
PoptrIAA16.3/1-229 DALGKMFSSFTIGNCG----SHGMKDFLNESKLIDLL-------NGTDYVPTYEDKDGDWMLVGDVPWEMFVESCKRLRIMKGTEATGLGNEYTA--------
PoptrIAA16.1/1-250 DALGKMFSSFTIGNCG----SQGTKDFMNESKLIDLL-------NSSEYVPTYEDKDGDWMLVGDVPWGMFVDSCKRLRIMKGSEAIGLAPRAVEKCKNRS-----
PoptrIAA26.2/1-249 IAVDELFRGLLAAQRD-SSSNGIMDKQEEAKAITGVL------DGSGEYTLVYEDNEGDMMLVGDVPWHMFVSTVKRLRVLKSSEVSALSRE-----------
PoptrIAA27.3/1-335 SALEKMFGCFTIGQCG--SHGLAARDGLTESCLKDLH--------GSEYVLTFEDKDGDWMLVGDVPWDMFTDSCRRLRIMKGSEAIGLAPRAMEKCKNRN-----
PoptrIAA26.1/1-338 TAVDELFRGLLAAQRD-SSCNGIMNKQEGEKAIMGVL------DGSGEYKLVYEDNEGDRMLVGDVPWHMFVSTVKRLRVLKSSEVSALNLGSSKHEKVPV-----
PoptrIAA16.2/1-246 DALGKMFSSFTIGNCG----SQGMKDFMNESKLIDLL-------NGSDYVPTYEDKDGDWMLVGDVPWEMFVDSCKRLRIMKGSEAIGLAPRAVEKCKNRI-----
PoptrIAA3.5/1-207 KALENMFKLTIGEYSE----------------REGY-------KGSEYAPTYEDKDGDWMLVGDVPWDMFLSSCKKLRIMKGSEAIGLGCGA------------
PoptrIAA19.2/1-189 VALEKLFGCFGIGKAL-------------------------KDTDDCEYVPIYEDKDGDWMLVGDVPWEMFIESCKRLRIMKRSEAKGFGLQPRGALQQGNISKDD
PoptrIAA15/1-220 NALQDMFSCFSFTIRN---------YLNERTIMEQEV------NNGVEYVPTYEDKDGDWMMLGDVPWKMFVESCKRLRLMKSSEATGFAPRTPSKCSSSS-----
PoptrIAA29.3/1-233 HTLLDMF-----GICQ-------------------------ENSSNYRLTYQDREGDWLLAEDVPWRNFLGSVQRLKLMRSSN--------------------
PoptrIAA20.1/1-174 QTLDEMFNTSILWPEM-----------------DVE------HSGKCHVLTYEDKEGDWLIVGDVPWEVFLPSVRRLKITRADSL-------------------
PoptrIAA19.1/1-189 VALEKLFGCFGIGKAL-------------------------KDTDDCEYVPIYEDKDGDWMLVGDVPWEMFIESCKRLRIMKRSEAKGFGLQPRGALQQGNISKDD
IAA1/1-168 KALENMFKFTVGEYSE----------------REGY-------KGSGFVPTYEDKDGDWMLVGDVPWDMFSSSCQKLRIMKGSEAPTAL---------------
IAA2/1-174 KALENMFKVMIGEYCE----------------REGY-------KGSGFVPTYEDKDGDWMLVGDVPWDMFSSSCKRLRIMKGSDAPALDSSL------------
IAA3/1-189 KALEVMFKFSVGEYFE----------------RDGY-------KGSDFVPTYEDKDGDWMLIGDVPWEMFICTCKRLRIMKGSEAKGLGCGV------------
IAA4/1-186 KSLENMFKFSVGEYFE----------------REGY-------KGSDFVPTYEDKDGDWMLVGDVPWEMFVSSCKRLRIMKGSEVKGLGCGGL-----------
IAA5/1-163 SALQILFGCYINFDDT-----------------------------LKESECVPIYEDKDGDWMLAGDVPWEMFLGSCKRLRIMKRSCNRG------------------
IAA6/1-189 TVLENLFGCLGIGVAK-------------------------EGKKCEYIIIYEDKDRDWMLVGDVPWQMFKESCKRLRIVKRSDATGFGLQQD-----------
IAA7/1-243 DALAKMFSSFTMGNYG----AQGMIDFMNESKLMNLL-------NSSEYVPSYEDKDGDWMLVGDVPWEMFVESCKRLRIMKGSEAVGLAPRAMEKYCKNRS-----
IAA8/1-321 SALEKMFSCFTLGQCG--LHGAQGRERMSEIKLKDLL-------HGSEFVLTYEDKDGDWMLVGDVPWEIFTETCQKLKIMKGSDSIGLAPGAVEKSKNRV---
IAA9/1-338 SALEKMFTTFTLGQCG--SNGAAGKDMLSETKLKDLL-------NGKDYVLTYEDKDGDWMLVGDVPWEMFIDVCKKLKIMKGCDAIGLAAAPRAMEKSKMRA---
IAA10/1-261 KTLDLMFFQIPSPVTR---SNTQGYKTIKETCTSKLL------DGSSEYIITYQDKDGDWMLVGDVPWQMFLGSVTRLRIMKTSIGAGVGK--------------
IAA11/1-246 NTLEEMFLKPKLGSRT-----LETDGHMETPVKILP------DGSSGLVLTYEDKEGDWMLVGDVPWGMFIGSVRRLRIMKTSEATGKAQMIL------------
IAA12/1-173 QTLEEMFFGMTGLYCF---QMRTL---------------------------
IAA13/1-246 QTLEDMFFRTNPGTVG---------LTSQFTKPLRLL------DGSSEFVLTYEDKEGDWMLVGDVPWRMFINSVKRLRVMKTSEANGLAARNQEPNERQRKQPV-
IAA14/1-228 DALAKMFSSFTMGSYG----AQGMIDFMNESKVMDLL-------NSSEYVPSYEDKDGDWMLVGDVPWPMFVESCKRLRIMKGSEAIGLAPRAMEKFKNRS-----
IAA15/1-179 TALENMFQGIITICRV-----------------TELE-------RKGEFVATYEDKDGDLMLVGDVPWMMFVESCKRMRLMKTGDAIGL----------------
IAA16/1-236 NALSKMFSSFTIGNYG----PQGMKDFMNESKLIDLL-------NGSDYVPTYEDKDGDWMLVGDVPWEMFVDSCKRIRIMKGSEAIGLAPRALEKCKNRS-----
IAA17/1-229 NALSNMFSSFTMGKHG---GEEGMIDFMNERKLMDLV-------NSWDYVPSYEDKDGDWMLVGDVPWPMFVDTCKRLRLMKGSDAIGLAPRAMEKCKSRA-----
IAA18/1-267 FTVDKLFRGLLAAQRD------FPSSIEDEKPITGLL------DGNGEYTLTYEDNEGDKMLVGDVPWQMFVSSVKRLRVIKTSEISSALTYGNGKQEKMRR----
IAA19/1-197 FALDKLFGFRGIGVAL-------------------------KDGDNCEYVTIYEDKDGDWMLAGDVPWGMFLESCKRLRIMKRSDATGFGLQPRGVDE--------
IAA20/1-175 RTLDFMFNASILWAEE-----------------EDMC------NEKSHVLTYADKEGDWMMVGDVPWEMFLSTVRRLKISRANYHY-----------------
IAA26/1-269 FVVDKLFRGLLAAQRD------ISDGQGEEKPIIGLL------DGKGEFTLTYEDNEGDKMLVGDVPWQMFVSSVKRLRVIKSSEISSALTFGCSKQEKMMH----
IAA27/1-305 SALEKMFSCFTIGQFG--SHGGCGRDGLNESRLTDLL-------RGSEYVVTYEDKSDWMLVGDVPWEMFICSCKKLRIMKSSEAIGLAPRVMEKCRSRN-----
IAA28/1-175 HAVDQLFSKKDSWDLN--------------------------RQYTLVYEDTEGDKVLVGDVPWEMFVSTVKRLHVLKTSHAFSLSPRKHGKE---------
IAA29/1-251 NSLITMFTEYEDCDRE---------------------------DTNYTFTFQGKEGDWLLRGDVTWKIFAESVHRISIIRDRPCAYTRCLF------------
IAA30/1-172 TTLDYMFNASILWAEE-----------------EDM------CSEKSHVLTYADKEGDWMMVGDVPWEMFLSSVRRLKISRAYHY-----------------
IAA31/1-158 ENLSHMFDTSIICGNR--------------------------DRKHHVLTYEDKDGDWMMVGDIPWDMFLETVRRLKITRPERY------------------
IAA32/1-143 LQLNDMF---------------------------------------DRVG--------------IEVVPD-----------------------------
IAA33/1-171 SALRQMFVDGADSTDD----------------LDLS------NAIPGHLIAYEDMENDLLLAGDLTWKDFVRVAKRIRILPVKGNTRQVKRNE-----------
IAA34/1-185 HQLEDMFGMQSVSGLR-----------------LF-------QMESEFCLVYRDEEGLWRNAGDVPWNEFIESVERLRITRRNDAVLPF--------------
Consensus
KALEKMFSCFTIGQCG-+SHGQGGKD++NESKLMDLLVN-EE+DDNGSEYVLTYEDKDGDWMLVGDVPWEMFVSSCKRLRIMKGSEAIGLAPRAMEKCKNR+XKP+D

	430 440 450 460
LOC_Os01g08320.1|11667.m00781/1-208	-----------------------------------
LOC_Os01g09450.1|11667.m00901_altSplice/1-237	-----------------------------------
LOC_Os01g13030.1|11667.m01279/1-267	-----------------------------------
LOC_Os01g18360.1|11667.m01799/1-204	-----------------------------------
LOC_Os01g48450.1|11667.m04699/1-272	-----------------------------------
LOC_Os01g53880.1|11667.m05292/1-262	-----------------------------------
LOC_Os02g13520.1|11668.m01248_altSplice/1-308	ALFX-------------------------------
LOC_Os02g49160.1|11668.m04779/1-206	-----------------------------------
LOC_Os02g56120.1|11668.m05537/1-183	-----------------------------------
LOC_Os02g57250.1|11668.m05658/1-282	SX---------------------------------
LOC_Os05g08570.1|11682.m00824_altSplice/1-213	-----------------------------------
LOC_Os05g09480.1|11682.m00921/1-229	-----------------------------------
LOC_Os05g14180.1|11682.m01308/1-258	-----------------------------------
LOC_Os05g44810.1|11682.m04283/1-328	-----------------------------------
LOC_Os05g48590.1|11682.m04652/1-282	-----------------------------------
LOC_Os06g07040.1|11680.m00651/1-184	-----------------------------------
LOC_Os06g22870.1|11680.m02247/1-267	-----------------------------------
LOC_Os06g24850.1|11680.m02452/1-150	-----------------------------------
LOC_Os06g39590.1|11680.m03858/1-194	-----------------------------------
LOC_Os07g08460.1|11673.m00782_altSplice/1-220	-----------------------------------
LOC_Os08g01780.1|11674.m00085/1-247	-----------------------------------
LOC_Os11g11410.1|11687.m01091/1-144	X----------------------------------
LOC_Os11g11420.1|11687.m01092/1-163	EDVDGNHWWRNHLWSVGPILQX-------------
LOC_Os11g11430.1|11687.m01093/1-172	EDGDGAAADDGVAAAADDVDDVAGYTSNDDPSFDX
LOC_Os12g40900.1|11686.m04059/1-198	-----------------------------------
LOC_Os12g40890.1|11686.m04058/1-278	-----------------------------------
LOC_Os03g43400.1|11669.m04312/1-234	-----------------------------------
LOC_Os03g43410.1|11669.m04313/1-227	-----------------------------------
LOC_Os03g53150.1|11669.m05378_altSplice/1-237	-----------------------------------
LOC_Os03g58350.1|11669.m05916/1-198	-----------------------------------
LOC_Os09g35870.1|11681.m03177/1-141	-----------------------------------
PoptrIAA3.6/1-192	-----------------------------------
PoptrIAA11/1-290	-----------------------------------
PoptrIAA33.1/1-149	-----------------------------------
PoptrIAA33.2/1-117	-----------------------------------
PoptrIAA28.1/1-239	-----------------------------------
PoptrIAA3.4/1-199	-----------------------------------
PoptrIAA29.2/1-233	-----------------------------------
PoptrIAA12.2/1-258	-----------------------------------
PoptrIAA3.2/1-203	-----------------------------------
PoptrIAA20.2/1-175	-----------------------------------
PoptrIAA7.2/1-248	-----------------------------------
PoptrIAA34/1-194	-----------------------------------
PoptrIAA3.3/1-199	-----------------------------------
PoptrIAA3.1/1-201	-----------------------------------
PoptrIAA27.1/1-307	-----------------------------------
PoptrIAA7.1/1-267	-----------------------------------
PoptrIAA29.1/1-229	-----------------------------------
PoptrIAA27.2/1-349	-----------------------------------
PoptrIAA16.4/1-237	-----------------------------------
PoptrIAA9/1-366	-----------------------------------
PoptrIAA19.3/1-184	-----------------------------------
PoptrIAA28.2/1-332	R----------------------------------
PoptrIAA12.1/1-276	-----------------------------------
PoptrIAA16.3/1-229	-----------------------------------
PoptrIAA16.1/1-250	-----------------------------------
PoptrIAA26.2/1-249	-----------------------------------
PoptrIAA27.3/1-335	-----------------------------------
PoptrIAA26.1/1-338	-----------------------------------
PoptrIAA16.2/1-246	-----------------------------------
PoptrIAA3.5/1-207	-----------------------------------
PoptrIAA19.2/1-189	RD---------------------------------
PoptrIAA15/1-220	-----------------------------------
PoptrIAA29.3/1-233	-----------------------------------
PoptrIAA20.1/1-174	-----------------------------------
PoptrIAA19.1/1-189	RD---------------------------------
IAA1/1-168	-----------------------------------
IAA2/1-174	-----------------------------------
IAA3/1-189	-----------------------------------
IAA4/1-186	-----------------------------------
IAA5/1-163	-----------------------------------
IAA6/1-189	-----------------------------------
IAA7/1-243	-----------------------------------
IAA8/1-321	-----------------------------------
IAA9/1-338	-----------------------------------
IAA10/1-261	-----------------------------------
IAA11/1-246	-----------------------------------
IAA12/1-173	-----------------------------------
IAA13/1-246	-----------------------------------
IAA14/1-228	-----------------------------------
IAA15/1-179	-----------------------------------
IAA16/1-236	-----------------------------------
IAA17/1-229	-----------------------------------
IAA18/1-267	-----------------------------------
IAA19/1-197	-----------------------------------
IAA20/1-175	-----------------------------------
IAA26/1-269	-----------------------------------
IAA27/1-305	-----------------------------------
IAA28/1-175	-----------------------------------
IAA29/1-251	-----------------------------------
IAA30/1-172	-----------------------------------
IAA31/1-158	-----------------------------------
IAA32/1-143	-----------------------------------
IAA33/1-171	-----------------------------------
IAA34/1-185	-----------------------------------
Consensus	RD-DG------------------------------
