# Supplementary material for: Genome-wide analysis of Aux/IAA and ARF gene families in Populus trichocarpa
Source: BMC Plant Biol. 2007 Nov 6;7:59. doi: 10.1186/1471-2229-7-59 (PMC2174922; doi:10.1186/1471-2229-7-59)
Supplement: Additional File 4 — Multiple sequence alignment of conserved regions of predicted Populus, Arabidopsis and rice Aux/IAA protein sequences. Sequences were aligned using MUSCLE program. Consensus sequence is indicated at the bottom of the alignment. [file 1471-2229-7-59-S4.pdf]

```
                                                          10        20        30        40        50        60        70        80        90
LOC_Os01g08320.1|11667.m00781/1-208               TALTLR-LPGSKARVVGWPPVRSFRKNALAFVKVAVDGAPYLRKVDLEAYSGYDQLLRALQDKFFEYVPTYEDKDGDWMLVGDVPWKMFVETCQRLRLMKS
LOC_Os01g09450.1|11667.m00901_altSplice/1-237     LELCLG-LPAYAAPVVGWPPVRSFRRNLASFVKINMDGVPIGRKVDLAAYGGYAQLSAAVDKLFREYTLVYEDDEGDRMLVGDVPWQMFIATAKRLRVLKS
LOC_Os01g13030.1|11667.m01279/1-267               TELRLG-LPGSKAQVVGWPPIRSYRKNTMAYVKVSMDGAPYLRKVDLKTYKNYKDMSLGLEKMFIEYVLTYEDKDGDWMLVGDVPWEMFTDSCRRLRIMKG
LOC_Os01g18360.1|11667.m01799/1-204               TDLRLG-LSLSSQVHADWPPIKPFLRSALQFVKVYMEGVPIGRKLDLLLLDGYDSLLIKLCHMFKAHVLTYEDQDGDWMMVGDVPWELFLSSVKKLRIARM
LOC_Os01g48450.1|11667.m04699/1-272               LTLRLG-LPGSKAQVVGWPPVRNYRKNTLAYVKVSMDGAPYLRKVDLKMYSSYEDLSMALEKMFSEYVLTYEDKDADWMLVGDLPWDLFTTICRKLKIMRG
LOC_Os01g53880.1|11667.m05292/1-262               LDLKLG-LPGFAAPVVGWPPIRSFRRNLASLVKINMDGIPIGRKIDLAAYNSYDGLSSAVKQLFH------------------------------------
LOC_Os02g13520.1|11668.m01248_altSplice/1-308     LELSLG-LPGGSTPVVGWPPVRTFRRNLATFIKINMDGVPIGRKIDLNAFDSYEKLSLAVDKLFREYTLVYEDYEGDKVLVGDVPWGMFVSSVKRLRVLKT
LOC_Os02g49160.1|11668.m04779/1-206               MDSCSGELPTTVVHIDGNNP--STPRSSLTFVKVYMEGVPIGRKLDLLPLDGYKGLVARLASMFRHHVLTYEDQEGDWMMAGDVPWELFLTSVKRLRIARA
LOC_Os02g56120.1|11668.m05537/1-183               DELSSSSSSGGKKTVVGWPPVSSARRACGGYVKVKKEGDAIGRKVDLALHSSYDELAATLARMFPGPVVTYEDGDGDWMLVGDVPWDDFARSVKRLKILGX
LOC_Os02g57250.1|11668.m05658/1-282               EELELG-LSLGSFGVVGWPPIRQFRMNSLFWKVKNMDGEVIGRKVDLNAHRSYKTLALALELMFTEYQLTYEDRDGDWMLVGDVPWEMFVSSVKRLRIMRT
LOC_Os05g08570.1|11682.m00824_altSplice/1-213     TALTLR-LPGSKARVVGWPPVRAFRKNALAFVKVAVDGAPYLRKVDLEAYRGYDQLLAALQDKFFEYVPTYEDKDGDWMLVGDVPWKMFVETCQRLRLMKS
LOC_Os05g09480.1|11682.m00921/1-229               LELSLA-LPGYAAPVVGWPPVRSFRRNLASFVKVNMDGVPIGRKVDLAAHGGYGELSAAVDRLFREYTLVYEDDEGDQMLVGDVPWNMFIAAARRLRVLRS
LOC_Os05g14180.1|11682.m01308/1-258               TELRLG-LPGSKAQVVGWPPIRSYRKNTMAYVKVSMDGAPYLRKVDLKTYKNYKDLSTALEKMFIEYVLTYEDKDGDWMLVGDVPWEMFANSCRRLRIMKG
LOC_Os05g44810.1|11682.m04283/1-328               LELKLG-LPGVIPPVVGWPPIRSFRRNLTNLVKINMDGIPIGRKVDLQIYDSYQKLSSAVEELFRVYTLVYEDNDGDRMLAGDIPWKVFVSTVKRLRVMRR
LOC_Os05g48590.1|11682.m04652/1-282               LALRLG-LPGSKAQVVGWPPVRSYRKNTLAYVKVSMDGAPYLRKVDLKTYSSYEDLSLALEKMFSEYVLTYEDKDADWMLVGDLPWDLFTTSCRKLRIMRG
LOC_Os06g07040.1|11680.m00651/1-184               LDLLNS-APGSRRKLVGWPPVKCLHRRDGYVKVKMEGLAIGRKLDLSILGSYAELLDTLHLMFPPYAVTYEDGEGDWMQVGDVPWEAFAKSVKRLKILVX
LOC_Os06g22870.1|11680.m02247/1-267               TELRLG-LPGTKAQVVGWPPIRSYRKNTMAYVKVSMDGAPYLRKVDLKMYKNYKELSLALEKMFSELVLTYEDKDEDWMLVGDVPWRMFTDSCRRLRIMKG
LOC_Os06g24850.1|11680.m02452/1-150               -----------SAQLVGWPPVRTFRKNLSTFVKVNLEGYAVGRKIDLKAHRSYDSLSQALQSMFHRYVLVYEDNEGDRMLVGDVPWEYVCLLX--------
LOC_Os06g39590.1|11680.m03858/1-194               TALTLA-LPGSKARAVGWPPVRAYRRNALRLVKVAVDGAPYLRKVDLAAHAGYAPLLRALHGMFAEYVPTYEDKDGDWMLVGDVPWKMFVESCKRIRLMKS
LOC_Os07g08460.1|11673.m00782_altSplice/1-220     LCLRLG-PPGSKAQVVGWPPVRAYRRNTFHYVKVSMDGAPYLRKVDLKMCKGYRELREALDLLFTQFAIAYEDKDGDLMLVGDVPWEMFISSCKKLRIMKG
LOC_Os08g01780.1|11674.m00085/1-247               LELRLG-ISSDSAQLVGWPPVRAFRKNLSTFVKVNLEGYAVGRKIDLKAHRSYDSLSQALQSMFHRYVLVYEDNEGDRMLVGDVPWELFIASVKRLYIAQD
LOC_Os11g11410.1|11687.m01091/1-144               -----------AADETTAPPPR--------FAKVHMDGYKVGRKINLRAHRNYDSLRRVLTKMTHEFIFLYEDFEGDRMLVGDVPWELFLASAKRLYIAKN
LOC_Os11g11420.1|11687.m01092/1-163               RNASAG-----EVKPAGLSPSR--------FVKVFMHGEPFERKINLAIHNNYDSLSFTLKRLGNDFDLLYDDMNGVRYLLGEVPWEVFTITVKRIYIVPA
LOC_Os11g11430.1|11687.m01093/1-172               -----------PVVKPGLSPSR--------FVKVFMHGEPFGRKINLALHNNYDSLSFTLKKLGNDFDLLYDDMDGVRYFLGDVPWEVFTTTVKKIYIVPA
LOC_Os12g40900.1|11686.m04059/1-198               TELRLG-LPGTKAQVVGWPPVRSYRKSCLQFVKVSMDGAPYLRKIDLKVYKGYRELREALEAMFLDFAVTYEDKDGDLMLVGDVPFEMFISTCKRLRIMKG
LOC_Os12g40890.1|11686.m04058/1-278               TELRLG-LPGGKAQVVGWPPVRSYRKNILAFVKVSMDGAPYLRKVDLKMYKSYLELSKALEKMFSEYVPTYEDKDGDWMLVGDVPWEMFVESCKRLRIMKG
LOC_Os03g43400.1|11669.m04312/1-234               TELRLG-LPGAKAQVVGWPPVRSFRKNVLALVKVSMDGAPYLRKIDVAMYKSYPELSMAFQNMFTEYVPTYEDKDGDWMLVGDVPWEMFVESCKRLRIMKG
LOC_Os03g43410.1|11669.m04313/1-227               TELRLG-LPGTKAQVVGWPPVRSYRKSCFQLVKVSMDGAPYLRKIDLRMYKGYRELREALEAMFVEFAITYQDKDGDLMLVGDVPFDMFTSTCKKLRIMKR
LOC_Os03g53150.1|11669.m05378_altSplice/1-237     TELRLG-LPGGKAQAVGWPPVRSFRRNIMTFVKVSMDGAPYLRKVDLKMYNSYKDLSLALQKMFGDAVTTYEDKDGDWMLVGDVPWQMFVESCKRLRIMKG
LOC_Os03g58350.1|11669.m05916/1-198               AELRLG-LPGSKVQVVGWPPVGSYRRSTFQYVKVSMDGAPYLRKVDLRMYGGYRELRDALDALFGHFAVAYEDKDGDLMLAGDVPWDMFISSCKKLRIMRG
LOC_Os09g35870.1|11681.m03177/1-141               TELTLG-PPGARGRKNGHPPPSSSMIQA--FVKVSMDGTPYLRKVDVAAYGDYLELVEALNDMFYEHAVVYEDGDGDWMLVGDVPWEMFVSSCKRMRVMRA
PoptrIAA3.6/1-192                                 TELRLG-LPGTKAQIVGWPPIRSYRKNTLQYVKVSMDGAPYLRKIDLKVYKGYPELLKALENMFKEYAPTYEDKDGDWMLIGDVPWDMFLSSCKKLRIIKG
PoptrIAA11/1-290                                  LELGLG-LSLGSSQVVGWPPIRSHRMHIMVFVKVNMDGTLIGRKVDLNAHGCYETLAQALENMFLEFVLTYEDKDGDWMLVGDVPWGMFISSVKRLRIMRM
PoptrIAA33.1/1-149                                TRPTSS-VPNF-------PPVT-----------VALEGRSICQRISLHKHESYHSLAKALRQMFVGHLIAYEDIENDLLLAGDLNWQDFVRVAKRIRILPA
PoptrIAA33.2/1-117                                DDL----VSTM------IPPVT-----------VVLEGRSICQRISLHKHASYHSLAKALRQMFVGHLIAYEDIESDLLLAGDLNWKDFVRVAKRIRILPA
PoptrIAA28.1/1-239                                LELRLG-PPGELAPVVGWPPIRSFRKNLAGFVKINMEGVPIGRKINLNAYDSYEKLSVAIDELFREYTLVYEDNEGDRILVGDVPWHMFVSTAKRLRVLKS
PoptrIAA3.4/1-199                                 TELRLG-LPGSKAQVVGWPPIRSYRKNCLQYVKVSVDGAPYLRKIDLKVYKSYPELLKALENMFKEFAPTYEDKDGDWMLVGDVPWDMFISSCKRLRIMKG
PoptrIAA29.2/1-233                                GQLQLGQYSWFSDWIVGWPPIK-FKKKKLSYIKVKMEGVGIARKIDVSLHHSFPTLKQTLLDMF-NYRLTYQDREGDWLLAEDVPWRNFLGTVQLLKLMRR
PoptrIAA12.2/1-258                                AELELG-LSLGGSQVVGWPPIRAYRMNSLVFVKVNMDGIPIGRKVDLNAHACYETLAQALEEMFFEFLLTYEDKEGDWMLVGDVPWGMFLNSVKRLRIMRT
PoptrIAA3.2/1-203                                 TELRLG-LPGTKAQVVGWPPIRSYRKNCLQYVKVSMDGAPYLRKIDLKVYKGYPELLKALEEMFKEHVPTYEDKDGDWMLVGDVPWDMFINSCKRLRIMKE
PoptrIAA20.2/1-175                                TDLRLG-LSISDQQLSDWPPIKPFLRKALAFVKVYMEGIPIGRKLNLLAHDGYHDLIQTLDQMFNCHVLTYEDKEGDWLIVGDVPWEMFLPSVRRLKITRA
PoptrIAA7.2/1-248                                 TELCLG-LPGAKAQVVGWPPVRSYRKNVMAFVKVCMDGAPYLRKVDLKMYRSYQELSDALAKMFSEYVPSYEDKDGDWMLVGDVPWEMFVNSCKRLRIMKG
PoptrIAA34/1-194                                  SGFLLN-PPALYGDLMDWPRANSPLKHSTSYVKVNMDGVIVGRKICMLDHGGYSSLALQLEDMFGEFCLFYKDREENWRTVGDVPWKEFVESVKRLRIARK
PoptrIAA3.3/1-199                                 TELRLG-LPGSKAQVVGWPPIRSYRKNCLQYVKVSVDGAPYLRKIDLKVYRSYPELLKALEDMFKDFAPTYEDKDGDWMLVGDVPWDMFISTCKRLRIMKG
PoptrIAA3.1/1-201                                 TELRLG-LPGTKTQVVGWPPIRSYRKNCLQYVKVSMDGAPYLRKIDLKVYKGYPELLEVVEEMFKEYVPTYEDKDGDWMLVGDVPWEMFINSCKRLRIMKE
PoptrIAA27.1/1-307                                TELRLG-LPGSKAQVVGWPPIRSFRKNTMAYVKVSMDGAPYLRKVDLKTFGSYMELSSALEKMFSEYVLTYEDKDNDWMLVGDVPWKMFTDSCRRLRIMKG
PoptrIAA7.1/1-267                                 TELCLG-LPGAKAQVVGWPPVRSYRKNVLAFVKVCMDGAPYLRKVDLKMYKSYQELSDALAKMFSEYVPSYEDKDGDWMLVGDVPWEMFVDSCKRLRIMKG
PoptrIAA29.1/1-229                                KDQMLGSKPWIENQVVGWPPIKSWRKKVLHYVKVKMEGVAITRKIDLRLYNSYQTLTKSLISMFARYSLTYQDKDGDWLIAGDVPWQTFMESVQRLKIVRN
PoptrIAA27.2/1-349                                TELRLG-LPGYKAQVVGWPPIRSFRKNTMAYVKVSMDGAPYLRKVDLKTYSNYLELSSALEKMFSEYVLTYEDKDGDWMLVGDVPWDMFTNSCRRLRIMKG
PoptrIAA16.4/1-237                                TELRLG-LPGNKAQVVGWPPVRSFRKNMLAFVKVSMDGAPYLRKVDLKMYKTYQELSDALGKMFSDYVPTYEDKDGDWMLVGDVPWDMFVESCKRLRIMKG
PoptrIAA9/1-366                                   TELRLG-LPGSKAQVVGWPPIKSFRKNSLAFIKVSMDGAPYLRKVDLRNYSAYQELSSALEKMFSEYVLTYEDKDGDWMLVGDVPWEMFIETCKRLRIMKS
PoptrIAA19.3/1-184                                TELRLG-LPGSKSQVVGWPPVCSYRKNISFYVKVSMDGAPFLRKIDLGMHKEYSDLVVALERLFGEYVPIYEDKDGDWMLVGDVPWEMFFESCKRLRIMKS
PoptrIAA28.2/1-332                                LELRLG-PPGELATVVGWPPIRSFRKNIASFVKINMEGVPIGRKINLNAYDSYEKLSVAIDELFREYTLVYEDNEGDRILVGDVPWHMFVSTAKRLRVLKS
PoptrIAA12.1/1-276                                LELGLG-LSLGGSQVVGWPPIRAYRMNSLVFVKVNMDGVPIGRKVDLNAHACYETLAQALEEMFFEFVLTYEDKEGDWMLVGDVPWGMFLTSVKRLRIMRT
PoptrIAA16.3/1-229                                TELRLG-LPGNKAQVVGWPPVRSFRKNMLAFVKVSMDGAPYLRKVDLKMYKTYHELSDALGKMFSDYVPTYEDKDGDWMLVGDVPWDMFVESCKRLRIMKG
PoptrIAA16.1/1-250                                TELRLG-LPGGKAQVVGWPPIRSFRKNVMAFVKVSMDGAPYLRKVDLKLYKSYRELSDALGKMFSEYVPTYEDKDGDWMLVGDVPWGMFVDSCKRLRIMKG
PoptrIAA26.2/1-249                                LELRLG-PPGEPGPVVGWPPIRSFRKNLASFVKINMEGVPIGRKVDLKAYDTYEKLSIAVDELFREYTLVYEDNEGDMMLVGDVPWHMFVSTVKRLRVLKS
PoptrIAA27.3/1-335                                TELRLG-LPGSKTQVVGWPPIRSFRKNTMAYVKVSMDGAPYLRKVDLKTYGNYLELSSALEKMFGEYVLTFEDKDGDWMLVGDVPWDMFTDSCRRLRIMKG
PoptrIAA26.1/1-338                                LELRLG-PPGEPGPVVGWPPIRSFRKNLATFVKINMEGVPIGRKVDLKAYDSYEKLSTAVDELFREYKLVYEDNEGDRMLVGDVPWHMFVSTVKRLRVLKS
PoptrIAA16.2/1-246                                TELRLG-LPGGKAQVVGWPPIRSFRKNVMAFVKVSMDGAPYLRKVDLKLYKSYQELSDALGKMFSDYVPTYEDKDGDWMLVGDVPWEMFVDSCKRLRIMKG
PoptrIAA3.5/1-207                                 TELRLG-LPGTRVQIVGWPPIRSYRKNSLQYVKVSMDGAPYLRKIDLKVYKGYPELLKALENMFKEYAPTYEDKDGDWMLVGDVPWDMFLSSCKKLRIMKG
PoptrIAA19.2/1-189                                TELRLG-PPGSTSQVVGWPPVCSYRKKNSFYVKVSMDGAPFLRKVDLGMHKEYSDLVVALEKLFGEYVPIYEDKDGDWMLVGDVPWEMFIESCKRLRIMKR
PoptrIAA15/1-220                                  TELTLG-LPGEKAQVVGWPPVRAYRKNAMKYVKVAVDGAPYLRKVDLEMYNSYQQLLNALQDMFSEYVPTYEDKDGDWMMLGDVPWKMFVESCKRLRLMKS
PoptrIAA29.3/1-233                                GQLHLGSYSWFSDGIVGWPPIK-FKRKKLSYIKVKMEGVGIARKIDVSVYRCFPTLKHTLLDMF-NYRLTYQDREGDWLLAEDVPWRNFLGSVQRLKLMRS
PoptrIAA20.1/1-174                                TDLRLG-LGISSEQLLDWPPIKPSPGKAVTFVKVYMEGIQIGRKLNLLAHDGYHDLIQTLDEMFNCHVLTYEDKEGDWLIVGDVPWEVFLPSVRRLKITRA
PoptrIAA19.1/1-189                                TELRLG-PPGSTSQVVGWPPVCSYRKKNSFYVKVSMDGAPFLRKVDLGMHKEYSDLVVALEKLFGEYVPIYEDKDGDWMLVGDVPWEMFIESCKRLRIMKR
IAA1/1-168                                        TELRLG-LPGAKTQIVGWPPVRSNRKNNNNYVKVSMDGAPYLRKIDLKMYKNYPELLKALENMFKGFVPTYEDKDGDWMLVGDVPWDMFSSSCQKLRIMKG
IAA2/1-174                                        TELCLG-LPGRKTQIVGWPPVRSSRKNNNSYVKVSMDGAPYLRKIDLKTYKNYPELLKALENMFKGFVPTYEDKDGDWMLVGDVPWDMFSSSCKRLRIMKG
IAA3/1-189                                        TELRLG-LPGTKAQIVGWPPVRSYRKNNIQYVKVSMDGAPYLRKIDLSCYKGYSELLKALEVMFKDFVPTYEDKDGDWMLIGDVPWEMFICTCKRLRIMKG
IAA4/1-186                                        TELRLG-LPGTKAQIVGWPPVRSYRKNNVQYVKVSMDGAPYLRKIDLTMYKQYPELMKSLENMFKDFVPTYEDKDGDWMLVGDVPWEMFVSSCKRLRIMKG
IAA5/1-163                                        TELRLG-LPGDKSQVVGWPPVCSYRRKNSLYVKVSVDGAAFLRKIDLEMYKCYQDLASALQILFGECVPIYEDKDGDWMLAGDVPWEMFLGSCKRLRIMKR
IAA6/1-189                                        TELRLG-LPGDKSQAVGWPPVCSYRRKKNNYVKVSMDGVPYMRKIDLGSSNSYINLVTVLENLFGEYIIIYEDKDRDWMLVGDVPWQMFKESCKRLRIVKR
IAA7/1-243                                        TELCLG-LPGGKAQVVGWPPVRNYRKNMMTLVKVSMDGAPYLRKVDLKMYKSYQDLSDALAKMFSEYVPSYEDKDGDWMLVGDVPWEMFVESCKRLRIMKG
IAA8/1-321                                        TELRLG-LPESKAQVVGWPPIRSYRKNTMAFVKVSMDGAPYLRKVDLRTYTSYQQLSSALEKMFSEFVLTYEDKDGDWMLVGDVPWEIFTETCQKLKIMKG
IAA9/1-338                                        TELTLG-LPGSKAQIVGWPPVRSYRKNTLAFVKVSMDGAPYLRKVDLRSYTNYGELSSALEKMFTDYVLTYEDKDGDWMLVGDVPWEMFIDVCKKLKIMKG
IAA10/1-261                                       LDLALG-LSIGRQVAVGWPPLRTYRINSLVLVKVTMDGVIIGRKVDLNALDSYAALEKTLDLMFFEYIITYQDKDGDWMLVGDVPWQMFLGSVTRLRIMKT
IAA11/1-246                                       NELELG-LTLSSGQVVGWPPIRTYRMNSMVFVKVTMDGIPIGRKIDLNAHKCYESLSNTLEEMFLGLVLTYEDKEGDWMLVGDVPWGMFIGSVRRLRIMKT
IAA12/1-173                                       LELGLG-LSLGSSQVVGWPPIGLHRMNSLVFVKVNMDGVGIGRKVDMRAHSSYENLAQTLEEMFF-----------------------------QMRTL--
IAA13/1-246                                       LELGLG-LSLGSSQVVGWPPIGSHRMNSLVFIKVNMDGVAIGRKVDLNAHSSYENLAQTLEDMFFEFVLTYEDKEGDWMLVGDVPWRMFINSVKRLRVMKT
IAA14/1-228                                       TELCLG-LPGGKAQVVGWPPVRNYRKNVMAFVKVSMDGAPYLRKVDLKMYTSYKDLSDALAKMFSEYVPSYEDKDGDWMLVGDVPWPMFVESCKRLRIMKG
IAA15/1-179                                       TELTLA-LPGTNDQLVGWPPVATARKTVRRYVKVALDGAAYLRKVDLGMYDCYGQLFTALENMFQEFVATYEDKDGDLMLVGDVPWMMFVESCKRMRLMKT
IAA16/1-236                                       TELRLG-LPGGKAQVVGWPPVRSFRKNVMSYVKVSMDGAPYLRKIDLKLYKTYQDLSNALSKMFSDYVPTYEDKDGDWMLVGDVPWEMFVDSCKRIRIMKG
IAA17/1-229                                       TELCLG-LPGGKAQVVGWPPVRSYRKNVMVFVKVSMDGAPYLRKIDLRMYKSYDELSNALSNMFSDYVPSYEDKDGDWMLVGDVPWPMFVDTCKRLRLMKG
IAA18/1-267                                       LELKLG-PPGEPGPVVGWPPVRSFRKNLASFVKINMYGVPIGRKVDLSAHNSYEQLSFTVDKLFREYTLTYEDNEGDKMLVGDVPWQMFVSSVKRLRVIKT
IAA19/1-197                                       TELRLG-LPGRKSQVVGWPPVCSYRKKNSCYVKVSMDGVPYLRKMDLGSSQGYDDLAFALDKLFGEYVTIYEDKDGDWMLAGDVPWGMFLESCKRLRIMKR
IAA20/1-175                                       TDLRLG-LSFG----VAAPAVEDAEYVAAVYVKVNMEGVPIGRKIDLMSLNGYRDLIRTLDFMFNSHVLTYADKEGDWMMVGDVPWEMFLSTVRRLKISRA
IAA26/1-269                                       LELRLG-PPGGPGPVVGWPPVRSFRKNLASFVKINMDGVPIGRKVDLNAYNSYEQLSFVVDKLFREFTLTYEDNEGDKMLVGDVPWQMFVSSVKRLRVIKS
IAA27/1-305                                       TELRLG-LPGSKAQVVGWPPIRSFRKNSMAYVKVSMEGAPYLRKIDLKTYKSYLELSSALEKMFSEYVVTYEDKDSDWMLVGDVPWEMFICSCKKLRIMKS
IAA28/1-175                                       LELRLA-PPCHVAPVVGWPPVRSSRRNLTAYVKINMEGVPIGRKVNLSAYNNYQQLSHAVDQLFSQYTLVYEDTEGDKVLVGDVPWEMFVSTVKRLHVLKT
IAA29/1-251                                       LNKHCA-IEGANSEVVGWPPVKSYHHRHIRYVKVKMDGVAIARKVDIKLFNSYESLTNSLITMFTNYTFTFQGKEGDWLLRGDVTWKIFAESVHRISIIRD
IAA30/1-172                                       TDLRLG-LSFGGGDNHEYDGVGAAEEMMIMYVKVNMEGVPIGRKIDLLSLNGYHDLITTLDYMFNSHVLTYADKEGDWMMVGDVPWEMFLSSVRRLKISRA
IAA31/1-158                                       LSLSLT-FPSTREARQDWPPIKSRLRDTLKFVKVYMEGVPIGRKLDLCVFSGYESLLENLSHMFDHHVLTYEDKDGDWMMVGDIPWDMFLETVRRLKITRP
IAA32/1-143                                       F------FKGSYGELIDWSQPSYNSITQLKYVKVNLDGLVVGRKVCLVDQGAYATLALQLNDMF--------DRVG-------------------IEVVPD
IAA33/1-171                                       -------EPQSTTPFIPKPASKNHNNSNSSPVTVVLEGRSICQRISLDKHGSYQSLASALRQMFVGHLIAYEDMENDLLLAGDLTWKDFVRVAKRIRILPV
IAA34/1-185                                       DSDPLGVFPNS-----------GYRRK---YVKVTMDGLVVGRKVCVLDHGSYSTLAHQLEDMFGEFCLVYRDEEGLWRNAGDVPWNEFIESVERLRITRR
```

Consensus

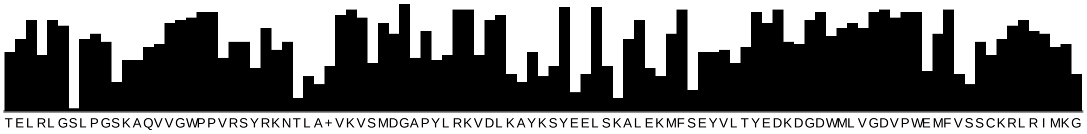

```
TELRLGSLPGSKAQVVGWPPVRSYRKNTLA+VKVSMDGAPYLRKVDLKAYKSYEELSKALEKMFSEYVLTYEDKDGDWMLVGDVPWEMFVSSCKRLRIMKG
```
