# Supplementary material for: Genome-wide analysis of Aux/IAA and ARF gene families in Populus trichocarpa
Source: BMC Plant Biol. 2007 Nov 6;7:59. doi: 10.1186/1471-2229-7-59 (PMC2174922; doi:10.1186/1471-2229-7-59)
Supplement: Additional File 5 — Prediction of conserved domains in full-length amino acid sequences of predicted Populus, Arabidopsis and rice Aux/IAA proteins. Conserved domains were predicted using the MEME and MAST programs. Motif number 6 represents the conserved domain I, Motif number 3 represents the conserved domain II, Motif number 2 represents the conserved domain III and Motif numbers 1, 4 and 5 represent the conserved domain IV. [file 1471-2229-7-59-S5.pdf]

Combined block diagrams: non-overlapping sites with p-value < 0.0001

Name	Combined p-value	Motifs
LOC_Os01g08320.1|11667.m	6.25e-97	6 3 2 5 4
LOC_Os01g09450.1|11667.m	7.85e-66	6 3 2 5 4
LOC_Os01g13030.1|11667.m	9.82e-111	6 3 2 1 4
LOC_Os01g18360.1|11667.m	5.19e-47	6 3 2 5
LOC_Os01g48450.1|11667.m	1.52e-86	6 3 2 5 4
LOC_Os01g53880.1|11667.m	2.97e-28	6 3 2
LOC_Os02g13520.1|11668.m	5.21e-59	6 3 2 5 4
LOC_Os02g49160.1|11668.m	5.47e-46	6 2 5 4
LOC_Os02g56120.1|11668.m	2.67e-47	3 2 5
LOC_Os02g57250.1|11668.m	3.32e-73	6 3 2 5 4
LOC_Os05g08570.1|11682.m	5.42e-95	6 3 2 5 4
LOC_Os05g09480.1|11682.m	9.16e-61	6 3 2 5 4
LOC_Os05g14180.1|11682.m	1.75e-110	6 3 2 1 4
LOC_Os05g44810.1|11682.m	1.56e-62	6 3 2 5 4
LOC_Os05g48590.1|11682.m	4.43e-90	6 3 2 5 4
LOC_Os06g07040.1|11680.m	1.58e-40	3 2 5
LOC_Os06g22870.1|11680.m	1.15e-102	6 3 2 5 4

LOC_Os06g24850.1|11680.m	2.52e-50	3 2 5
LOC_Os06g39590.1|11680.m	4.35e-90	6 3 2 5 4
LOC_Os07g08460.1|11673.m	8.27e-75	6 3 2 5
LOC_Os08g01780.1|11674.m	7.40e-56	3 2 5
LOC_Os11g11410.1|11687.m	1.18e-35	2 5
LOC_Os11g11420.1|11687.m	1.41e-22	2 5
LOC_Os11g11430.1|11687.m	1.16e-27	2 5
LOC_Os12g40900.1|11686.m	5.58e-98	6 3 2 5 4
LOC_Os12g40890.1|11686.m	2.00e-113	6 3 2 1 4
LOC_Os03g43400.1|11669.m	2.00e-109	6 3 2 1 4
LOC_Os03g43410.1|11669.m	3.39e-90	6 3 2 5 4
LOC_Os03g53150.1|11669.m	8.72e-108	6 3 2 1 4
LOC_Os03g58350.1|11669.m	5.65e-72	6 3 2 5
LOC_Os09g35870.1|11681.m	3.91e-71	6 2 5 4
PoptrIAA3.6	1.73e-92	6 3 2 1
PoptrIAA11	3.51e-77	6 3 2 1 4
PoptrIAA33.1	8.07e-22	2 5
PoptrIAA33.2	1.16e-21	4 5
PoptrIAA28.1	4.22e-68	6 3 2 5 4
PoptrIAA3.4	1.89e-113	6 3 2 1 4
PoptrIAA29.2	9.88e-36	3 2 5
PoptrIAA12.2	4.37e-84	3 2 1 4

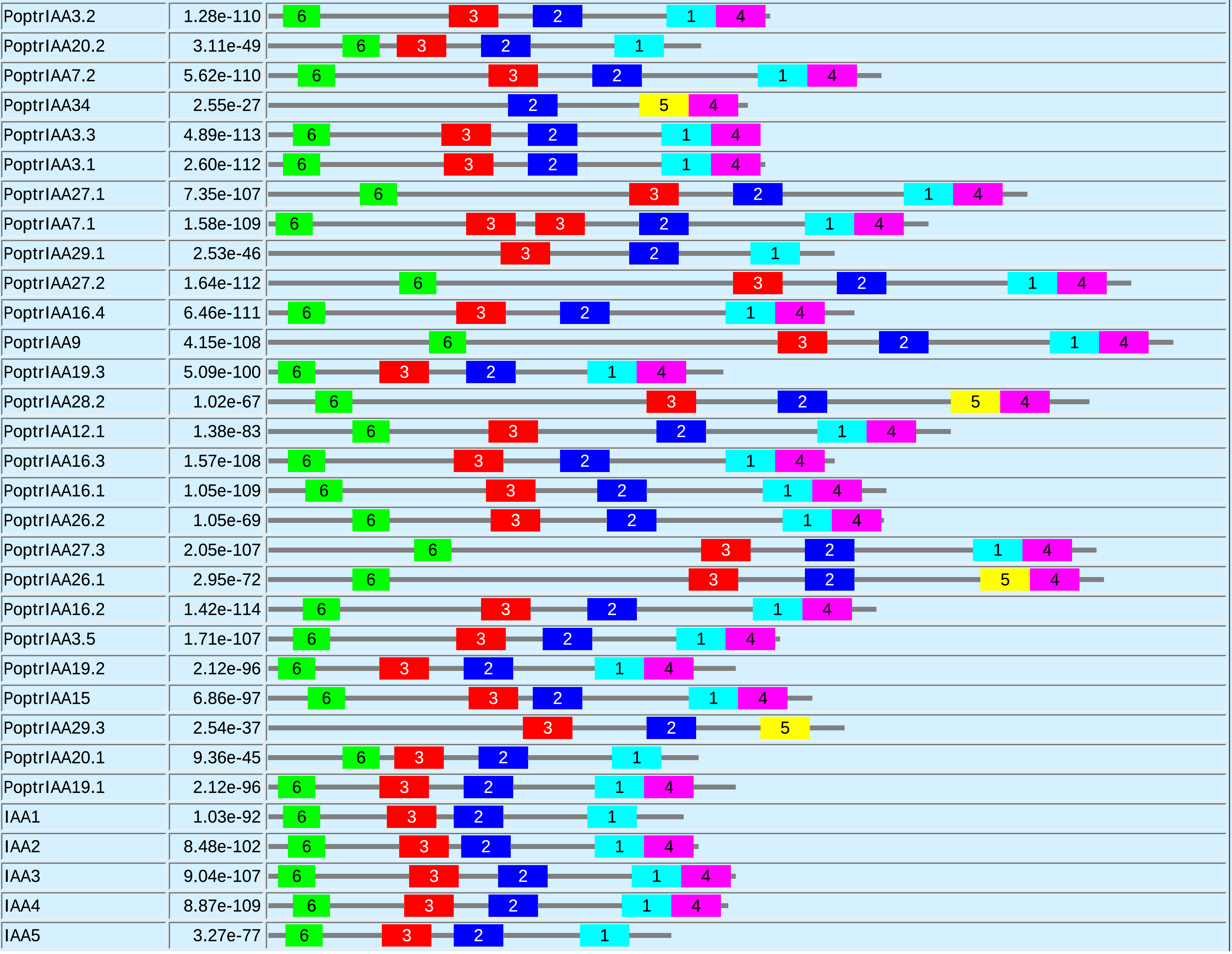

PoptrIAA3.2 1.28e-110 6 3 2 1 4
PoptrIAA20.2 3.11e-49 6 3 2 1
PoptrIAA7.2 5.62e-110 6 3 2 1 4
PoptrIAA34 2.55e-27 2 5 4
PoptrIAA3.3 4.89e-113 6 3 2 1 4
PoptrIAA3.1 2.60e-112 6 3 2 1 4
PoptrIAA27.1 7.35e-107 6 3 2 1 4
PoptrIAA7.1 1.58e-109 6 3 3 2 1 4
PoptrIAA29.1 2.53e-46 3 2 1
PoptrIAA27.2 1.64e-112 6 3 2 1 4
PoptrIAA16.4 6.46e-111 6 3 2 1 4
PoptrIAA9 4.15e-108 6 3 2 1 4
PoptrIAA19.3 5.09e-100 6 3 2 1 4
PoptrIAA28.2 1.02e-67 6 3 2 5 4
PoptrIAA12.1 1.38e-83 6 3 2 1 4
PoptrIAA16.3 1.57e-108 6 3 2 1 4
PoptrIAA16.1 1.05e-109 6 3 2 1 4
PoptrIAA26.2 1.05e-69 6 3 2 1 4
PoptrIAA27.3 2.05e-107 6 3 2 1 4
PoptrIAA26.1 2.95e-72 6 3 2 5 4
PoptrIAA16.2 1.42e-114 6 3 2 1 4
PoptrIAA3.5 1.71e-107 6 3 2 1 4
PoptrIAA19.2 2.12e-96 6 3 2 1 4
PoptrIAA15 6.86e-97 6 3 2 1 4
PoptrIAA29.3 2.54e-37 3 2 5
PoptrIAA20.1 9.36e-45 6 3 2 1
PoptrIAA19.1 2.12e-96 6 3 2 1 4
IAA1 1.03e-92 6 3 2 1
IAA2 8.48e-102 6 3 2 1 4
IAA3 9.04e-107 6 3 2 1 4
IAA4 8.87e-109 6 3 2 1 4
IAA5 3.27e-77 6 3 2 1

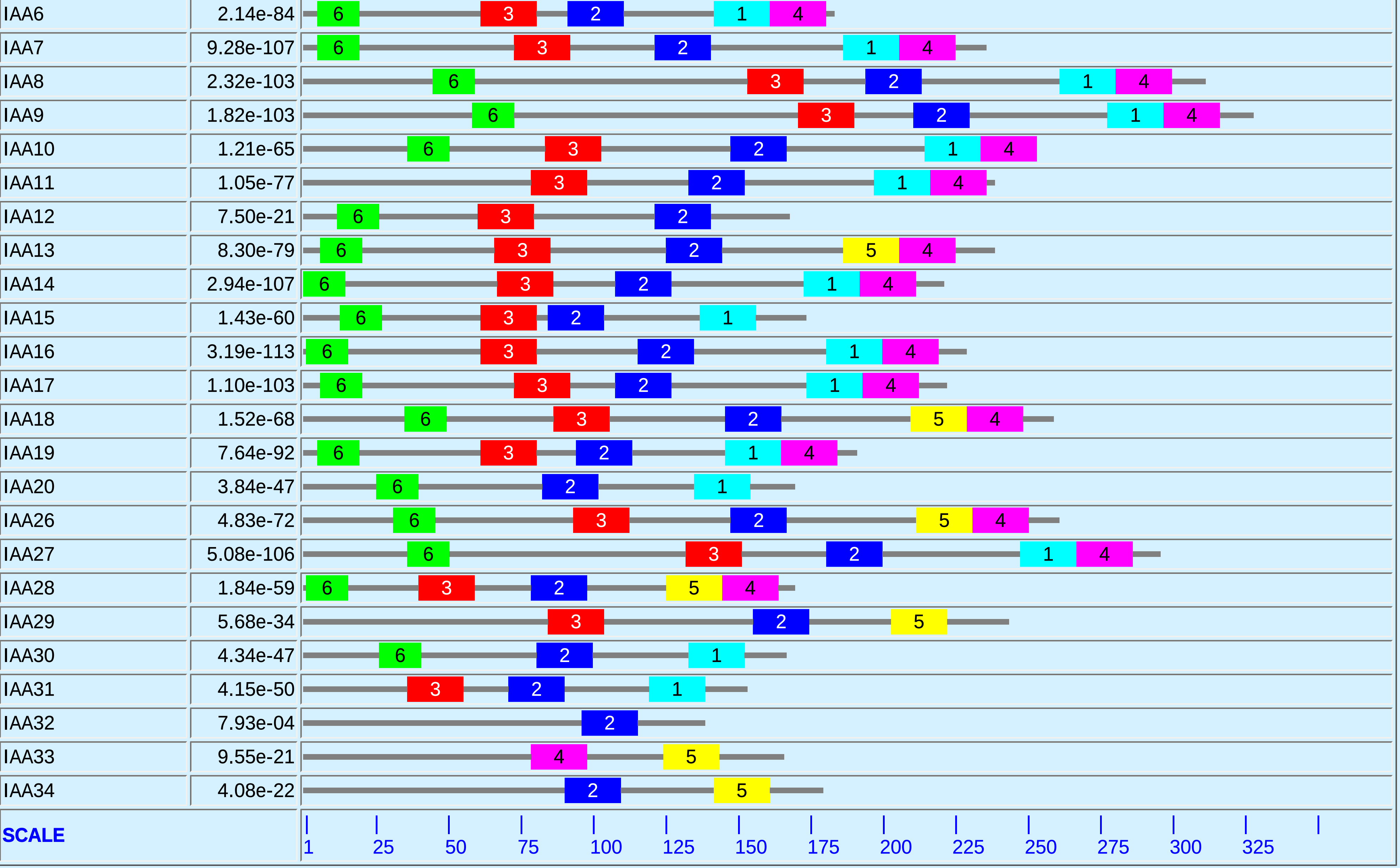
IAA6 2.14e-84 6 3 2 1 4
IAA7 9.28e-107 6 3 2 1 4
IAA8 2.32e-103 6 3 2 1 4
IAA9 1.82e-103 6 3 2 1 4
IAA10 1.21e-65 6 3 2 1 4
IAA11 1.05e-77 3 2 1 4
IAA12 7.50e-21 6 3 2
IAA13 8.30e-79 6 3 2 5 4
IAA14 2.94e-107 6 3 2 1 4
IAA15 1.43e-60 6 3 2 1
IAA16 3.19e-113 6 3 2 1 4
IAA17 1.10e-103 6 3 2 1 4
IAA18 1.52e-68 6 3 2 5 4
IAA19 7.64e-92 6 3 2 1 4
IAA20 3.84e-47 6 2 1
IAA26 4.83e-72 6 3 2 5 4
IAA27 5.08e-106 6 3 2 1 4
IAA28 1.84e-59 6 3 2 5 4
IAA29 5.68e-34 3 2 5
IAA30 4.34e-47 6 2 1
IAA31 4.15e-50 3 2 1
IAA32 7.93e-04 2
IAA33 9.55e-21 4 5
IAA34 4.08e-22 2 5
SCALE
1 25 50 75 100 125 150 175 200 225 250 275 300 325
