# Supplementary figures and images for: Genome-wide analysis of Aux/IAA and ARF gene families in Populus trichocarpa
Source: BMC Plant Biol. 2007 Nov 6;7:59. doi: 10.1186/1471-2229-7-59 (PMC2174922; doi:10.1186/1471-2229-7-59)

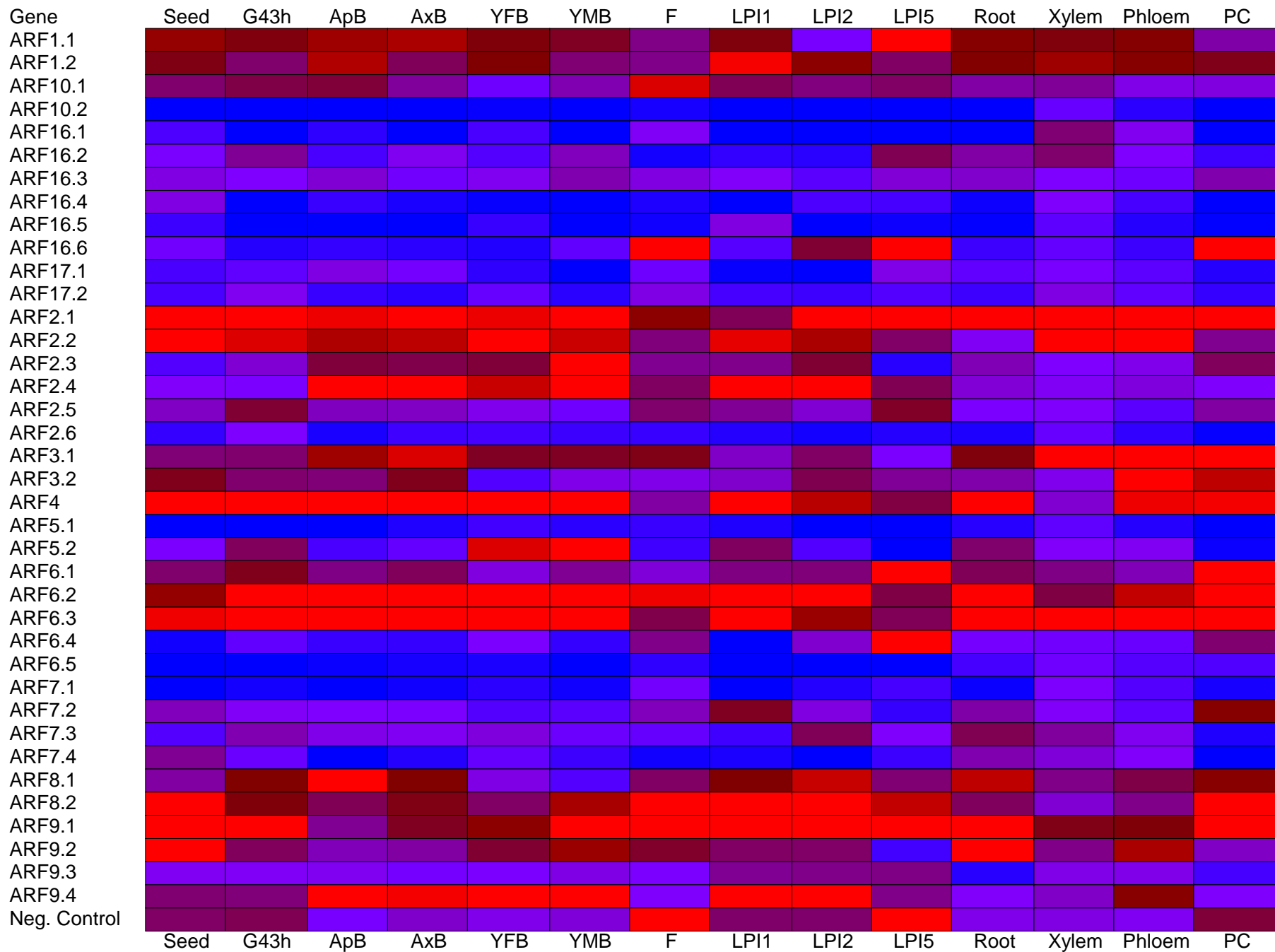

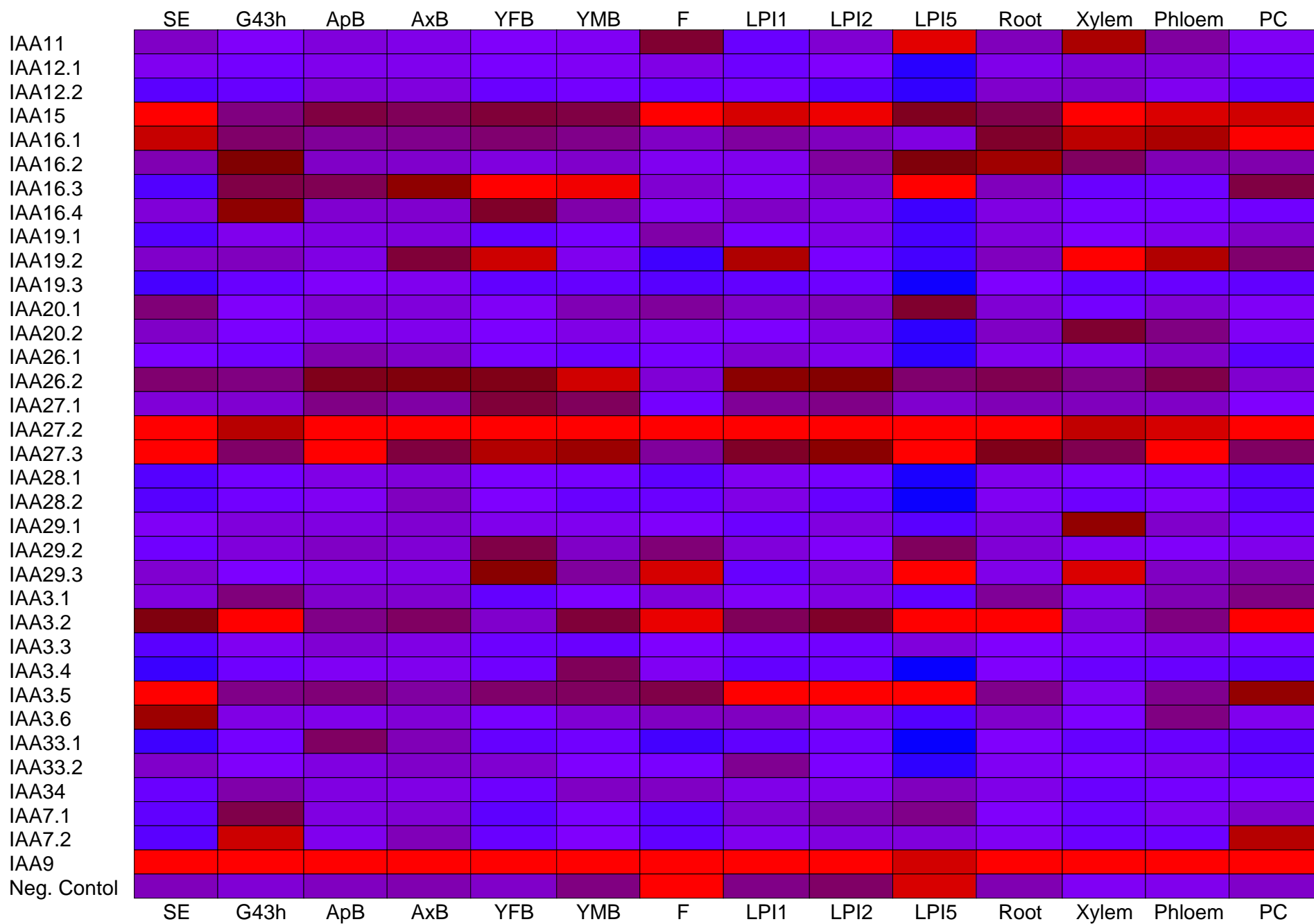

Supplement: Additional File 9 — Temperature diagram representation of microarray results. Figures are temperature diagrams with intensity of signal calculated in columns, which are derived from means for all data for a particular tissue type. The bottom line in each figure represents the negative control. Red means higher expression, dark blue means lower expression. Sample abbreviations are defined in Additional file 17. [file 1471-2229-7-59-S9.pdf]

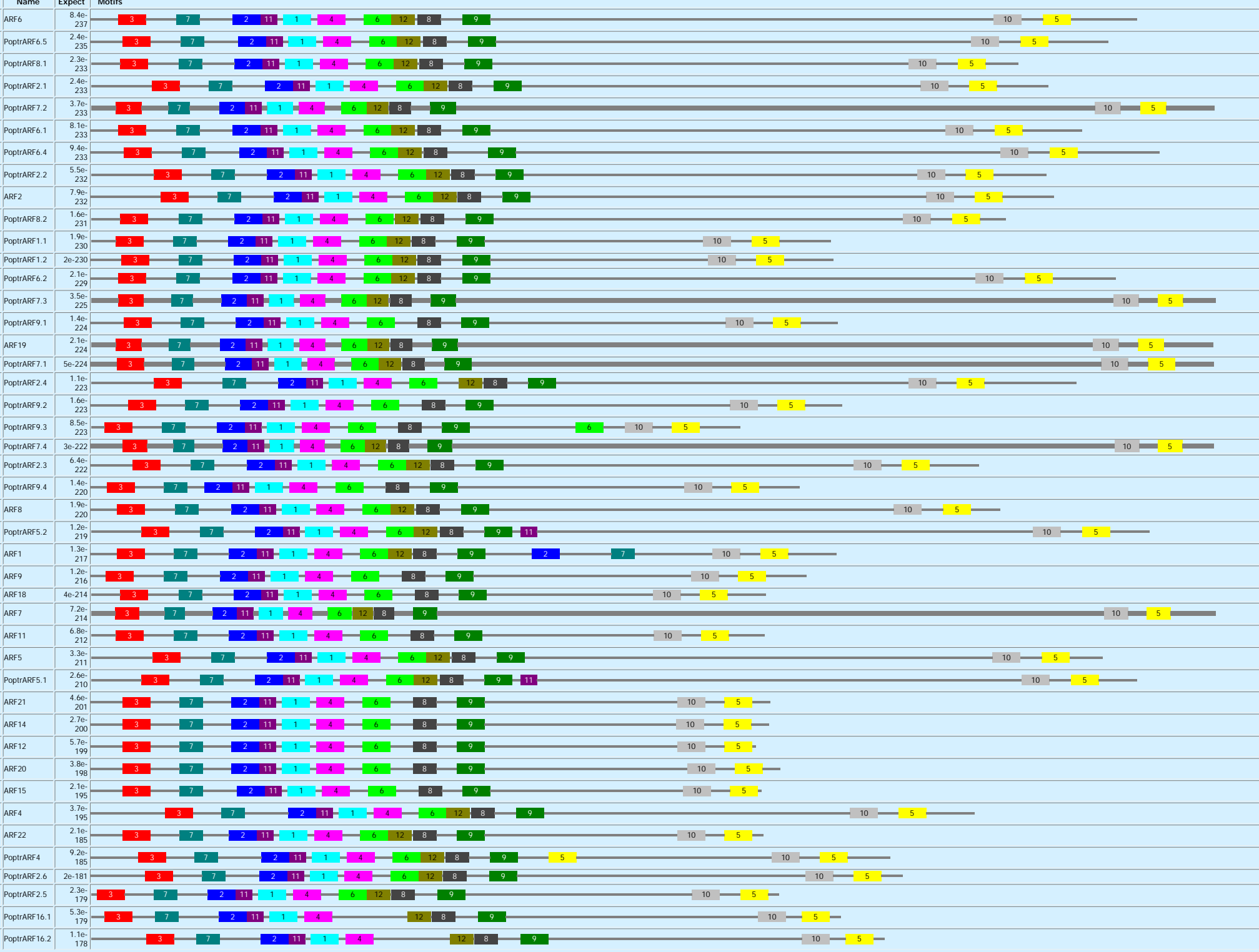

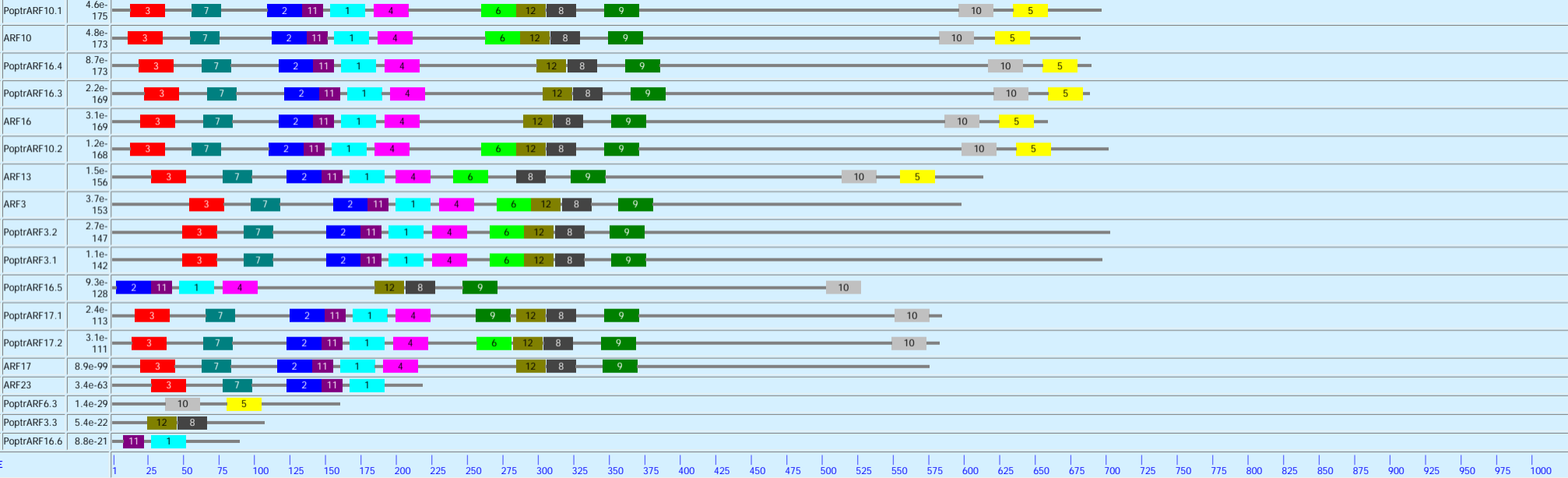

Supplement: Additional File 12 — Prediction of conserved domains in full-length amino acid sequences of predicted Populus, Arabidopsis and rice ARF proteins. Conserved domains were predicted using the MEME and MAST programs. Motif numbers 1, 2, 3, 4, 7 and 11 represent the conserved B3 domain. Motif numbers 6, 8 and 12 represent the conserved auxin response domain and Motif numbers 5 and 10 represent the conserved C-terminal Aux/IAA domain (III and IV). [file 1471-2229-7-59-S12.pdf]

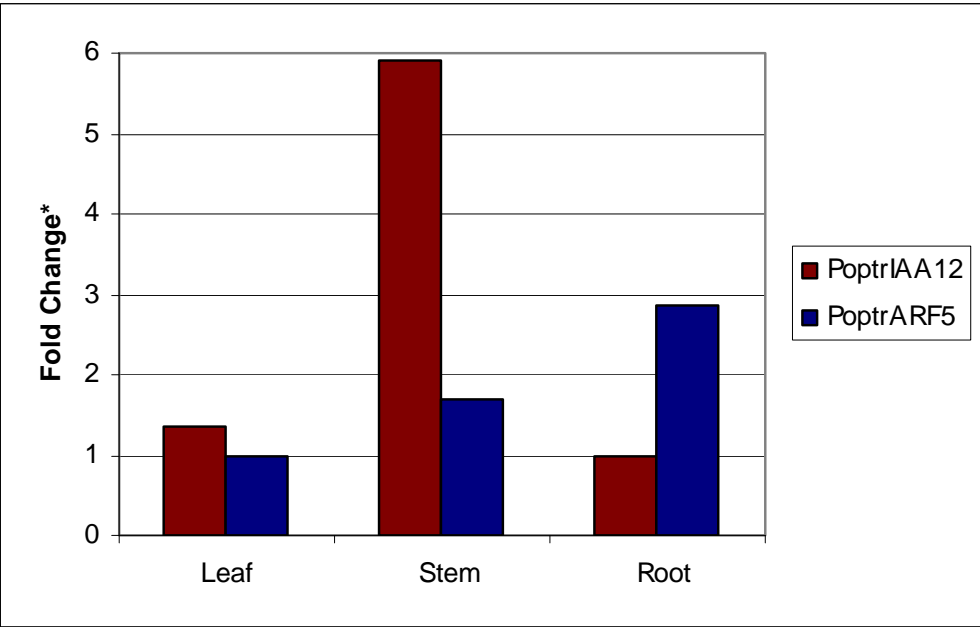

Supplement: Additional File 13 — Expression analysis of PoptrARF5 and PoptrIAA12 genes using real-time RT-PCR. * Fold Change is represented relative to lowest value observed for the gene. Lowest value was determined by comparison of relative threshold cycle values for a specific gene across leaf, stem and root samples. Fold change was calculated by the formula 2-ΔΔCt, where ΔΔCt is the difference between ΔCt of a gene is a given tissue and the lowest value ΔCt for that gene in any of the three tissue types. ΔCt was estimated by the formula; (Ct of gene of interest) – (geometric mean of ΔCt of 18S RNA gene, control gene). [file 1471-2229-7-59-S13.pdf]

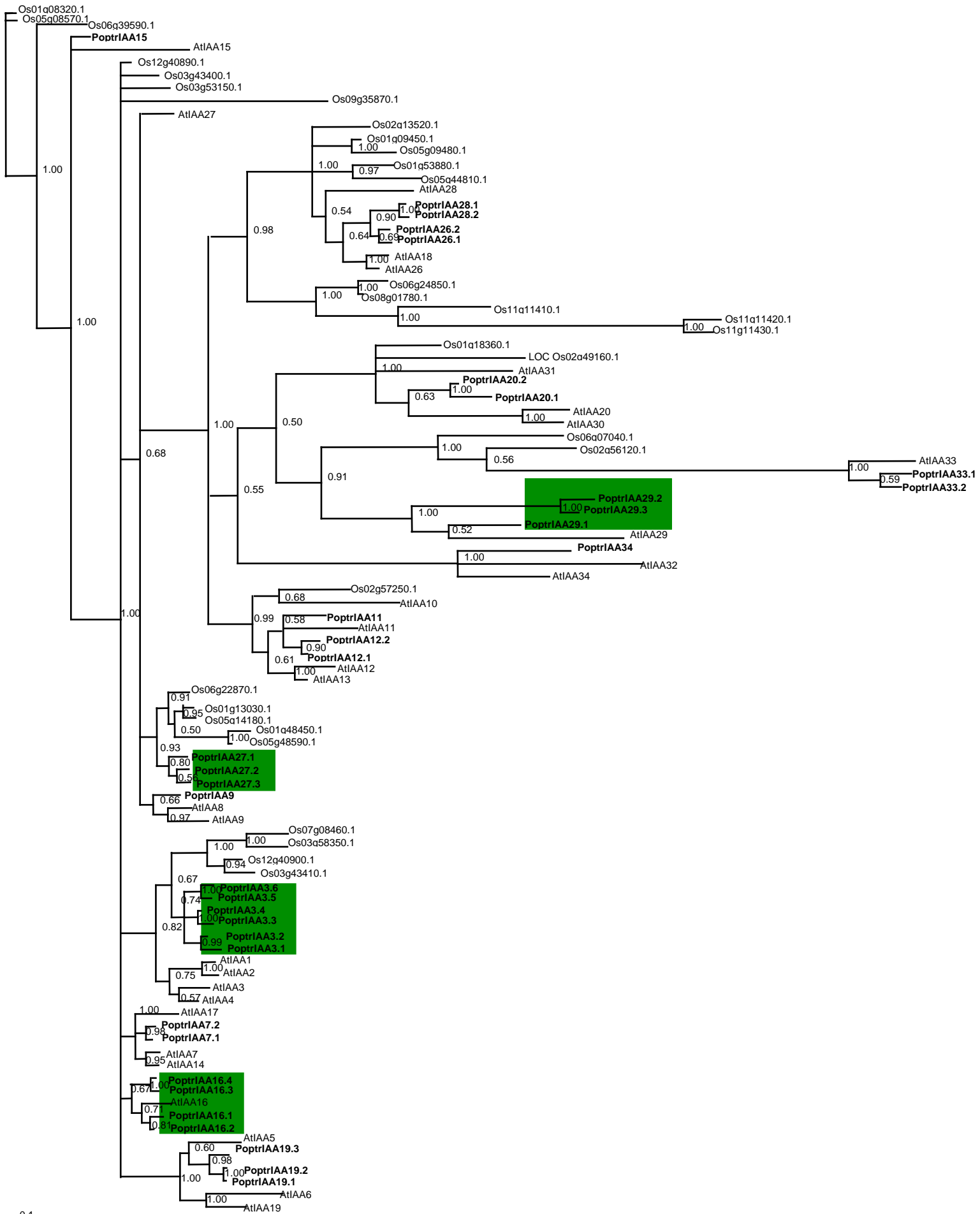

Supplement: Additional File 14 — Bayesian phylogenetic analysis of conserved regions of predicted Aux/IAA protein sequences using MRBAYES. Amino acid sequences of full-length predicted proteins were aligned using MUSCLE program. Tree was produced using conserved collated regions (See Additional file 4) as described in methods. Posterior probabilities calculated from consensus are shown on branches. Green boxes represent sustained expansion of subgroups in Populus. [file 1471-2229-7-59-S14.pdf]

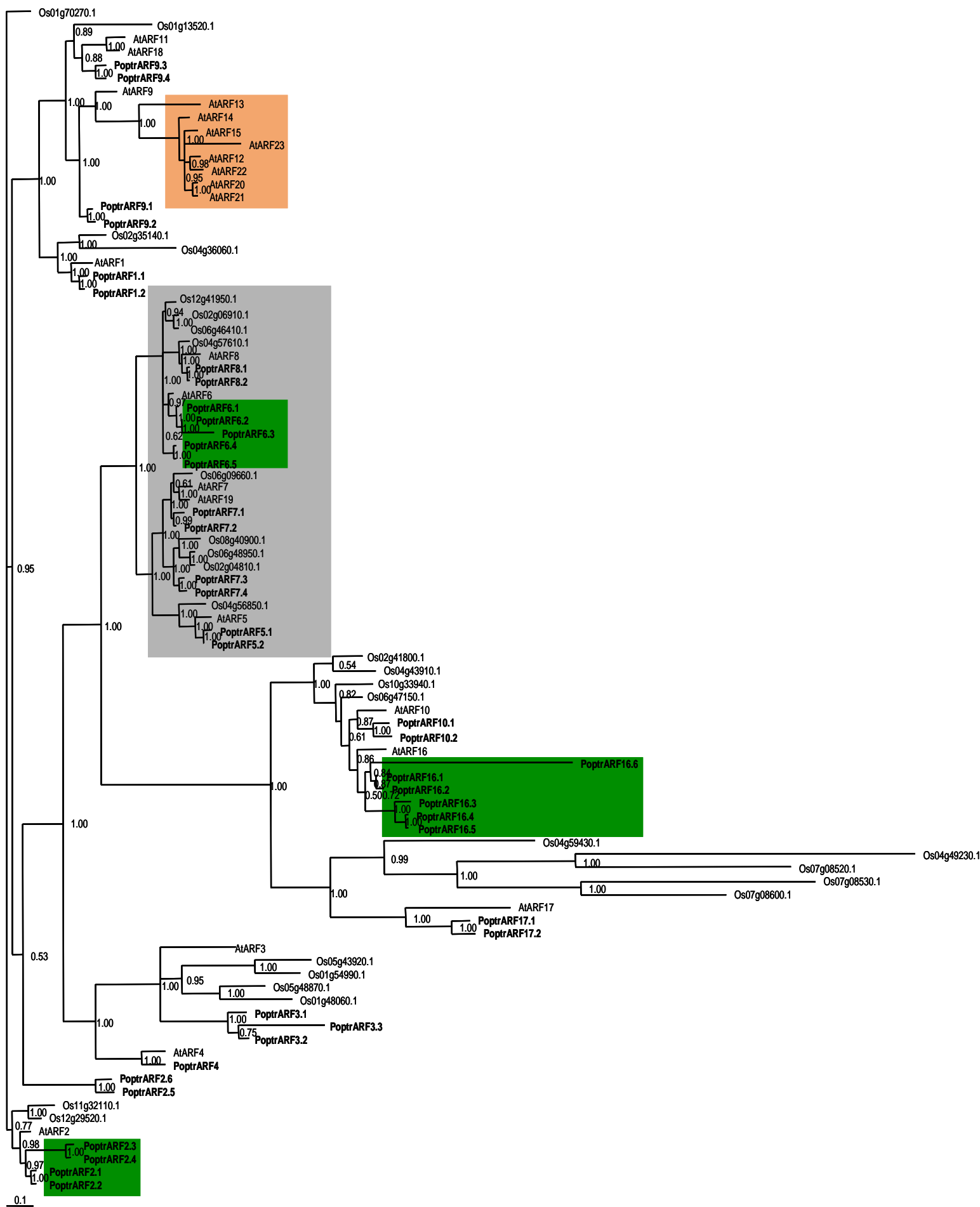

Supplement: Additional File 15 — Bayesian phylogenetic analysis of conserved regions of predicted ARF protein sequences using MRBAYES. Amino acid sequences of full-length predicted proteins were aligned using MUSCLE program. Tree was produced using conserved collated regions (See Additional file 11) as described in methods. Posterior probabilities calculated from consensus are shown on branches. Green and orange boxes represent sustained expansion of subgroups in Populus and Arabidopsis, respectively. Grey box represents the Q-rich activator ARF subgroup. [file 1471-2229-7-59-S15.pdf]
