# Supplementary material for: Genome-wide analysis of Aux/IAA and ARF gene families in Populus trichocarpa
Source: BMC Plant Biol. 2007 Nov 6;7:59. doi: 10.1186/1471-2229-7-59 (PMC2174922; doi:10.1186/1471-2229-7-59)
Supplement: Additional File 10 — Multiple sequence alignment of full-length amino acid sequences of predicted Populus, Arabidopsis and rice ARF proteins. Sequences were aligned using MUSCLE program. Consensus sequence is indicated at the bottom of the alignment. [file 1471-2229-7-59-S10.pdf]

```
                                                            10        20        30        40        50        60        70        80        90        100
LOC_Os01g70270.1|11667.m07038_altsplice/1-809      ----------------------------------------------------------------------------------------------------------
LOC_Os01g13520.1|11667.m01333/1-700                ----------------------------------------------------------------------------------------------------------
LOC_Os02g06910.1|11668.m00642/1-909                ----------------------------------------------------------------------------------------------------------
LOC_Os02g35140.1|11668.m03331/1-757                ----------------------------------------------------------------------------------------------------------
LOC_Os02g41800.1|11668.m03990/1-784                ----------------------------------------------------------------------------------------------------------
LOC_Os04g57610.1|11670.m05695/1-819                ----------------------------------------------------------------------------------------------------------
LOC_Os04g43910.1|11670.m04249/1-696                ----------------------------------------------------------------------------------------------------------
LOC_Os04g49230.1|11670.m04840/1-294                ----------------------------------------------------------------------------------------------------------
LOC_Os04g36060.1|11670.m03490/1-1674               ----------------------------------------------------------------------------------------------------------
LOC_Os05g43920.1|11682.m04181/1-700                ----------------------------------------------------------------------------------------------------------
LOC_Os06g09660.1|11680.m00929/1-1056               ----------------------------------------------------------------------------------------------------------
LOC_Os06g46410.1|11680.m04579/1-918                ----------------------------------------------------------------------------------------------------------
LOC_Os06g47150.1|11680.m04656/1-731                ----------------------------------------------------------------------------------------------------------
LOC_Os06g48950.1|11680.m04850/1-1116               ----------------------------------------------------------------------------------------------------------
LOC_Os08g40900.1|11674.m04106_altsplice/1-1056     ----------------------------------------------------------------------------------------------------------
LOC_Os11g32110.1|11687.m02952/1-812                ----------------------------------------------------------------------------------------------------------
LOC_Os12g41950.1|11686.m04180/1-900                ----------------------------------------------------------------------------------------------------------
LOC_Os12g29520.1|11686.m02861_altsplice/1-842      ----------------------------------------------------------------------------------------------------------
LOC_Os04g56850.1|11670.m05610_altsplice/1-956      ----------------------------------------------------------------------------------------------------------
LOC_Os05g48870.1|11682.m04683_altsplice/1-696      ----------------------------------------------------------------------------------------------------------
LOC_Os01g48060.1|11667.m04657_altsplice/1-723      ----------------------------------------------------------------------------------------------------------
LOC_Os01g54990.1|11667.m05421/1-656                ----------------------------------------------------------------------------------------------------------
LOC_Os10g33940.1|11676.m02996/1-699                ----------------------------------------------------------------------------------------------------------
LOC_Os02g04810.1|11668.m00419/1-1094               ----------------------------------------------------------------------------------------------------------
LOC_Os04g59430.1|11670.m05892/1-530                ----------------------------------------------------------------------------------------------------------
LOC_Os07g08520.1|11673.m00789/1-729                ----------------------------------------------------------------------------------------------------------
LOC_Os07g08530.1|11673.m00790/1-408                ----------------------------------------------------------------------------------------------------------
LOC_Os07g08600.1|11673.m00797/1-525                MPLPSCRRREQRPRQSCFQNPECHLRLRARQQRPQESAVQSTLSSSIYFSDHFVLTPPPFQITPTSIQNSTWLPIPTSPAVAAPPLPRCLGGVDGEGAPCAPPPSP
ARF1/1-665                                         ----------------------------------------------------------------------------------------------------------
ARF3/1-608                                         ----------------------------------------------------------------------------------------------------------
ARF4/1-788                                         ----------------------------------------------------------------------------------------------------------
ARF5/1-902                                         ----------------------------------------------------------------------------------------------------------
ARF6/1-933                                         ----------------------------------------------------------------------------------------------------------
ARF7/1-1165                                        ----------------------------------------------------------------------------------------------------------
ARF8/1-811                                         ----------------------------------------------------------------------------------------------------------
ARF9/1-638                                         ----------------------------------------------------------------------------------------------------------
ARF10/1-693                                        ----------------------------------------------------------------------------------------------------------
ARF11/1-601                                        ----------------------------------------------------------------------------------------------------------
ARF12/1-593                                        ----------------------------------------------------------------------------------------------------------
ARF13/1-623                                        ----------------------------------------------------------------------------------------------------------
ARF14/1-605                                        ----------------------------------------------------------------------------------------------------------
ARF15/1-598                                        ----------------------------------------------------------------------------------------------------------
ARF16/1-670                                        ----------------------------------------------------------------------------------------------------------
ARF17/1-585                                        ----------------------------------------------------------------------------------------------------------
ARF18/1-602                                        ----------------------------------------------------------------------------------------------------------
ARF19/1-1086                                       ----------------------------------------------------------------------------------------------------------
ARF20/1-615                                        ----------------------------------------------------------------------------------------------------------
ARF21/1-606                                        ----------------------------------------------------------------------------------------------------------
ARF22/1-600                                        ----------------------------------------------------------------------------------------------------------
ARF23/1-222                                        ----------------------------------------------------------------------------------------------------------
ARF2/1-859                                         ----------------------------------------------------------------------------------------------------------
PoptrARF8.1/1-827                                  ----------------------------------------------------------------------------------------------------------
PoptrARF1.1/1-660                                  ----------------------------------------------------------------------------------------------------------
PoptrARF7.3/1-1113                                 ----------------------------------------------------------------------------------------------------------
PoptrARF2.3/1-792                                  ----------------------------------------------------------------------------------------------------------
PoptrARF5.1/1-933                                  ----------------------------------------------------------------------------------------------------------
PoptrARF5.2/1-944                                  ----------------------------------------------------------------------------------------------------------
PoptrARF7.4/1-1137                                 ----------------------------------------------------------------------------------------------------------
PoptrARF16.1/1-669                                 ----------------------------------------------------------------------------------------------------------
PoptrARF1.2/1-662                                  ----------------------------------------------------------------------------------------------------------
PoptrARF9.1/1-666                                  ----------------------------------------------------------------------------------------------------------
PoptrARF2.1/1-854                                  ----------------------------------------------------------------------------------------------------------
PoptrARF16.3/1-700                                 ----------------------------------------------------------------------------------------------------------
PoptrARF3.1/1-709                                  ----------------------------------------------------------------------------------------------------------
PoptrARF6.2/1-914                                  ----------------------------------------------------------------------------------------------------------
PoptrARF6.4/1-953                                  ----------------------------------------------------------------------------------------------------------
PoptrARF17.1/1-594                                 ----------------------------------------------------------------------------------------------------------
PoptrARF2.4/1-879                                  ----------------------------------------------------------------------------------------------------------
PoptrARF16.2/1-708                                 ----------------------------------------------------------------------------------------------------------
PoptrARF2.2/1-852                                  ----------------------------------------------------------------------------------------------------------
PoptrARF10.1/1-708                                 ----------------------------------------------------------------------------------------------------------
PoptrARF3.3/1-109                                  ----------------------------------------------------------------------------------------------------------
PoptrARF9.2/1-670                                  ----------------------------------------------------------------------------------------------------------
PoptrARF6.1/1-884                                  ----------------------------------------------------------------------------------------------------------
PoptrARF4/1-713                                    ----------------------------------------------------------------------------------------------------------
PoptrARF6.3/1-163                                  ----------------------------------------------------------------------------------------------------------
PoptrARF3.2/1-714                                  ----------------------------------------------------------------------------------------------------------
PoptrARF9.3/1-579                                  ----------------------------------------------------------------------------------------------------------
PoptrARF6.5/1-907                                  ----------------------------------------------------------------------------------------------------------
PoptrARF9.4/1-632                                  ----------------------------------------------------------------------------------------------------------
PoptrARF7.1/1-1047                                 ----------------------------------------------------------------------------------------------------------
PoptrARF7.2/1-1093                                 ----------------------------------------------------------------------------------------------------------
PoptrARF8.2/1-816                                  ----------------------------------------------------------------------------------------------------------
PoptrARF16.6/1-91                                  ----------------------------------------------------------------------------------------------------------
PoptrARF16.4/1-701                                 ----------------------------------------------------------------------------------------------------------
PoptrARF16.5/1-536                                 ----------------------------------------------------------------------------------------------------------
PoptrARF10.2/1-713                                 ----------------------------------------------------------------------------------------------------------
PoptrARF17.2/1-592                                 ----------------------------------------------------------------------------------------------------------
PoptrARF2.6/1-724                                  ----------------------------------------------------------------------------------------------------------
PoptrARF2.5/1-614                                  ----------------------------------------------------------------------------------------------------------

Consensus
                                                   ----------------------------------------------------------------------------------------------------------
```

```
                                                 110       120       130       140       150       160       170       180       190       200       210
LOC_Os01g70270.1|11667.m07038_altsplice/1-809    ----------------------------------------------------------------------------------------------------------
LOC_Os01g13520.1|11667.m01333/1-700              ----------------------------------------------------------------------------------------------------------
LOC_Os02g06910.1|11668.m00642/1-909              -------------------------------------------------------------------------------------------------------MKL
LOC_Os02g35140.1|11668.m03331/1-757              ----------------------------------------------------------------------------------------------------------
LOC_Os02g41800.1|11668.m03990/1-784              ----------------------------------------------------------------------------------------------------------
LOC_Os04g57610.1|11670.m05695/1-819              ------------------------------------------------------------------------------------------------------MSSS
LOC_Os04g43910.1|11670.m04249/1-696              ----------------------------------------------------------------------------------------------------------
LOC_Os04g49230.1|11670.m04840/1-294              ----------------------------------------------------------------------------------------------------------
LOC_Os04g36060.1|11670.m03490/1-1674             ----------------------------------------------------------------------------------------------------------
LOC_Os05g43920.1|11682.m04181/1-700              ----------------------------------------------------------------------------------------------------------
LOC_Os06g09660.1|11680.m00929/1-1056             ----------------------------------------------------------------------------------------------------------
LOC_Os06g46410.1|11680.m04579/1-918              -------------------------------------------------------------------------------------------------------MRL
LOC_Os06g47150.1|11680.m04656/1-731              ----------------------------------------------------------------------------------------------------------
LOC_Os06g48950.1|11680.m04850/1-1116             ----------------------------------------------------------------------------------------------------------
LOC_Os08g40900.1|11674.m04106_altsplice/1-1056   ----------------------------------------------------------------------------------------------------------
LOC_Os11g32110.1|11687.m02952/1-812              --------------------------------------------------------------------------------------------MATAEVGGGGGEGD
LOC_Os12g41950.1|11686.m04180/1-900              ---------------------------------------------------------------------------------------------------------M
LOC_Os12g29520.1|11686.m02861_altsplice/1-842    -----------------------------------------------------------------------------------------------------MAAAA
LOC_Os04g56850.1|11670.m05610_altsplice/1-956    ----------------------------------------------------------------------------------------------MASSQEKAKTGV
LOC_Os05g48870.1|11682.m04683_altsplice/1-696    -----------------------------------------------------------------------------------------------MTGIDLNTVEE
LOC_Os01g48060.1|11667.m04657_altsplice/1-723    ----------------------------------------------------------------------------------------------MVGIDLNTVEEE
LOC_Os01g54990.1|11667.m05421/1-656              ----------------------------------------------------------------------------------------------------------
LOC_Os10g33940.1|11676.m02996/1-699              ----------------------------------------------------------------------------------------------------------
LOC_Os02g04810.1|11668.m00419/1-1094             -----------------------------------------------------------------------------------------MASMKQQQTPASSAVTA
LOC_Os04g59430.1|11670.m05892/1-530              ----------------------------------------------------------------------------------------------------------
LOC_Os07g08520.1|11673.m00789/1-729              ----------------------------------------------------------------------------------------------------------
LOC_Os07g08530.1|11673.m00790/1-408              ----------------------------------------------------------------------------------------------------------
LOC_Os07g08600.1|11673.m00797/1-525              IPAGPASSTVSAASSAPATRDYLGGITGHRLGQEDDSGGHEYTSVSGIHVPSSSGVAFVSFGQCPQEVISTDLCLGLGPIAATAPLQPPPPPAAAAAAGSPSATTP
ARF1/1-665                                       ----------------------------------------------------------------------------------------------------------
ARF3/1-608                                       -------------------------------------------------------------------------------MGGLIDLNVMETEEDETQTQTPSSASG
ARF4/1-788                                       --------------------------------------------------------------------MEFDLNTEIAEVEEEENDDVGVGVGGGTRIDKGRLGIS
ARF5/1-902                                       -------------------------------------------------------------------------------MMASLSCVEDKMKTSCLVNGGGTITTT
ARF6/1-933                                       ----------------------------------------------------------------------------------------------------------
ARF7/1-1165                                      ----------------------------------------------------------------------------------------------------------
ARF8/1-811                                       ----------------------------------------------------------------------------------------------------------
ARF9/1-638                                       ----------------------------------------------------------------------------------------------------------
ARF10/1-693                                      ----------------------------------------------------------------------------------------------------------
ARF11/1-601                                      ----------------------------------------------------------------------------------------------------------
ARF12/1-593                                      ----------------------------------------------------------------------------------------------------------
ARF13/1-623                                      ----------------------------------------------------------------------------------------------------------
ARF14/1-605                                      ----------------------------------------------------------------------------------------------------------
ARF15/1-598                                      ----------------------------------------------------------------------------------------------------------
ARF16/1-670                                      ----------------------------------------------------------------------------------------------------------
ARF17/1-585                                      ----------------------------------------------------------------------------------------------------------
ARF18/1-602                                      ----------------------------------------------------------------------------------------------------------
ARF19/1-1086                                     ----------------------------------------------------------------------------------------------------------
ARF20/1-615                                      ----------------------------------------------------------------------------------------------------------
ARF21/1-606                                      ----------------------------------------------------------------------------------------------------------
ARF22/1-600                                      ----------------------------------------------------------------------------------------------------------
ARF23/1-222                                      ----------------------------------------------------------------------------------------------------------
ARF2/1-859                                       ------------------------------------------------------------------------MASSEVSMKGNRGGDNFSSSGFSDPKETRNVSVA
PoptrARF8.1/1-827                                ----------------------------------------------------------------------------------------------------------
PoptrARF1.1/1-660                                ----------------------------------------------------------------------------------------------------------
PoptrARF7.3/1-1113                               ----------------------------------------------------------------------------------------------------------
PoptrARF2.3/1-792                                -------------------------------------------------------------------------------------------------MINGSYSVD
PoptrARF5.1/1-933                                -----------------------------------------------------------------------------------------MGSAEEKIKTGGIINGA
PoptrARF5.2/1-944                                -----------------------------------------------------------------------------------------MGSVEEKIKTGGLANGA
PoptrARF7.4/1-1137                               ------------------------------------------------------------------------------------------------------MKSP
PoptrARF16.1/1-669                               ----------------------------------------------------------------------------------------------------------
PoptrARF1.2/1-662                                ----------------------------------------------------------------------------------------------------------
PoptrARF9.1/1-666                                ---------------------------------------------------------------------------------------------------------M
PoptrARF2.1/1-854                                --------------------------------------------------------------------------------MASSEISAKANSGNIKGGGESFSSGY
PoptrARF16.3/1-700                               ----------------------------------------------------------------------------------------------------------
PoptrARF3.1/1-709                                ------------------------------------------------------------------------------------MVGMIDLNTTEEDETTPSSGSL
PoptrARF6.2/1-914                                ----------------------------------------------------------------------------------------------------------
PoptrARF6.4/1-953                                ---------------------------------------------------------------------------------------------------------M
PoptrARF17.1/1-594                               ----------------------------------------------------------------------------------------------------------
PoptrARF2.4/1-879                                ------------------------------------------------------------------------------MELRAGAGVFRWLVKVPMIIHNDFLSFS
PoptrARF16.2/1-708                               -------------------------------------------------------------------------------------MFEAKLRDGYTQVLQIPKLHS
PoptrARF2.2/1-852                                ------------------------------------------------------------------------------MASSEISAKANSGNIRGGGESFTSGYSE
PoptrARF10.1/1-708                               ----------------------------------------------------------------------------------------------------------
PoptrARF3.3/1-109                                ----------------------------------------------------------------------------------------------------------
PoptrARF9.2/1-670                                -----------------------------------------------------------------------------------------------------MAMNR
PoptrARF6.1/1-884                                ----------------------------------------------------------------------------------------------------------
PoptrARF4/1-713                                  --------------------------------------------------------------------------------------------MEIDLNHPVTEVEK
PoptrARF6.3/1-163                                ----------------------------------------------------------------------------------------------------------
PoptrARF3.2/1-714                                ------------------------------------------------------------------------------------MVGMIDLNTIEEDETTPSCGSL
PoptrARF9.3/1-579                                ----------------------------------------------------------------------------------------------------------
PoptrARF6.5/1-907                                ----------------------------------------------------------------------------------------------------------
PoptrARF9.4/1-632                                ----------------------------------------------------------------------------------------------------------
PoptrARF7.1/1-1047                               ----------------------------------------------------------------------------------------------------------
PoptrARF7.2/1-1093                               ----------------------------------------------------------------------------------------------------------
PoptrARF8.2/1-816                                ----------------------------------------------------------------------------------------------------------
PoptrARF16.6/1-91                                ----------------------------------------------------------------------------------------------------------
PoptrARF16.4/1-701                               ----------------------------------------------------------------------------------------------------------
PoptrARF16.5/1-536                               ----------------------------------------------------------------------------------------------------------
PoptrARF10.2/1-713                               ----------------------------------------------------------------------------------------------------------
PoptrARF17.2/1-592                               ----------------------------------------------------------------------------------------------------------
PoptrARF2.6/1-724                                --------------------------------------------------------------------------------------MGKKRSVAGEAKTSRSHEVG
PoptrARF2.5/1-614                                ----------------------------------------------------------------------------------------------------------

Consensus
                                                 -----------------------------------------------------------------------DL-----MMMA+I+++++++S++E++KT+GSASSA
```

```
                                                         220       230       240       250       260       270       280       290       300       310
LOC_Os01g70270.1|11667.m07038_altsplice/1-809   ---MPPAAMAPPPPPQGSSTGDPLYDELWHACAGPLV-TVPRVGDLVFYFPQGHIEQ-------------------------------------------------
LOC_Os01g13520.1|11667.m01333/1-700             ---------MSSQGAGGGVGDPELFAELWRACAGPLV-EVPQRDERVFYFLQGHLEQ-------------------------------------------------
LOC_Os02g06910.1|11668.m00642/1-909             SPSAGGVSDQPPSPPEVAEEQKCLNSELWHACAGPLV-SLPAVGSRVVYFPQGHSEQ-------------------------------------------------
LOC_Os02g35140.1|11668.m03331/1-757             -MAGSVVAAAAAAGGGTGSSCDALYRELWHACAGPLV-TVPRQGELVYYFPQGHMEQ-------------------------------------------------
LOC_Os02g41800.1|11668.m03990/1-784             ------MITFADLAEPAPGAERCVDRQLWLACAGGMC-TVPPVGAAVYYFPQGHAEH-------------------------------------------------
LOC_Os04g57610.1|11670.m05695/1-819             SAASIGPPQPPPPPAPPEEEKKCLNSELWHACAGPLV-CLPTVGTRVVYFPQGHSEQ-------------------------------------------------
LOC_Os04g43910.1|11670.m04249/1-696             ---------MELAGPTEGDGGGSVDSQLWAACAGSMS-SVPPVGAAVYYFPQGHAEQ-------------------------------------------------
LOC_Os04g49230.1|11670.m04840/1-294             ---------MEQEQDEEEEEEAEASPREIPFMTSAAAA--------------------------------------------------------------------
LOC_Os04g36060.1|11670.m03490/1-1674            -------MEMAANPGGSGTCSDALFRELWHACAGPLV-TVPKRGERVYYFPQGHMEQ-------------------------------------------------
LOC_Os05g43920.1|11682.m04181/1-700             ----MGIDLNTVEEEAEEGAAAAVCGELWHACAGPGV-ALPRRGSALVYLPQAHLAA-------------------------------------------------
LOC_Os06g09660.1|11680.m00929/1-1056            ---MKDQGSSGVSPAPGEGEKKAINSELWHACAGPLV-SLPPVGSLVVYFPQGHSEQ-------------------------------------------------
LOC_Os06g46410.1|11680.m04579/1-918             SSSSGSVLPAQAASPEAVEEQKCLNSELWHACAGPLV-SLPAVGSRVVYFPQGHSEQ-------------------------------------------------
LOC_Os06g47150.1|11680.m04656/1-731             ----MITFVDSAAKERERESDKCLDPQLWHACAGGMV-QMPPVSSKVYYFPQGHAEH-------------------------------------------------
LOC_Os06g48950.1|11680.m04850/1-1116            MAAAAAAAVVGSGCEGEKTKAPAINSELWHACAGPLV-SLPPAGSLVVYFPQGHSEQ-------------------------------------------------
LOC_Os08g40900.1|11674.m04106_altsplice/1-1056  ------------------------------------------------------MRK-------------------------------------------------
LOC_Os11g32110.1|11687.m02952/1-812             AAAAAVARAGGGGGGGGGGGEDALFTELWSACAGPLV-TVPRVGEKVFYFPQGHIEQ-------------------------------------------------
LOC_Os12g41950.1|11686.m04180/1-900             KLSPPASADMPQALPENDGEQRCLNSELWHACAGPLV-SLPVVRSRVVYFPQGHSEQ-------------------------------------------------
LOC_Os12g29520.1|11686.m02861_altsplice/1-842   TAAASPVEGLTGGGGGGGGGVDGLFVELWRACAGPLV-TVPAVGERVFYLPQGHIEQ-------------------------------------------------
LOC_Os04g56850.1|11670.m05610_altsplice/1-956   LRNAAALLDEMQLMGETQGAKKVINSELWHACAGPLV-CLPQRGSLVYYFPQGHSEQ-------------------------------------------------
LOC_Os05g48870.1|11682.m04683_altsplice/1-696   DEEEAAEEVAANGSSPAPARAGAVCLELWHACAGPVA-PLPRKGGVVVYLPQGHLEH-------------------------------------------------
LOC_Os01g48060.1|11667.m04657_altsplice/1-723   EDEEEGGATGTVTAPAEARAGGAVCLELWHACAGPVA-PLPRKGSAVVYLPQGHLEH-------------------------------------------------
LOC_Os01g54990.1|11667.m05421/1-656             --MGIDLNNTASGGEEDAPAPGPVCRDLWHACAGPVV-SLPRRGSAVVYLPQGHLSA-------------------------------------------------
LOC_Os10g33940.1|11676.m02996/1-699             -----------MKEVGEVEEVRCLDPQLWHACAGGMV-QMPAPRSRVYYFAQGHAEH-------------------------------------------------
LOC_Os02g04810.1|11668.m00419/1-1094            AAAASSSATAAVAACEGERKAAAINSELWHACAGPLV-SLPPVGSLVVYFPQGHSEQLLITVLVHYQSGWEHWAYWLPFSVALLVWSIDSLGRTNPAIIRAFLFRK
LOC_Os04g59430.1|11670.m05892/1-530             MARPPAATAPPPPPPPPPPPPPIDRLVWLACAAPLS-RIPVVGTQVSYFPEGHAEQ-------------------------------------------------
LOC_Os07g08520.1|11673.m00789/1-729             -----MAQPPDAAAAAAVPPPVVIDRDVWHACAVPYSGVLPGVGTLVYYIPHGHIEQ-------------------------------------------------
LOC_Os07g08530.1|11673.m00790/1-408             --------MAAAAAPLADDGDGIVDRDMWLACAAPNSGRLPAVGSVVFYFVDGHAAQ-------------------------------------------------
LOC_Os07g08600.1|11673.m00797/1-525             EPGHGEATPTTSSSAQFQTQAQQVTRDMWMACAAPKSGRLPTVGSLVYYFPDGHAEQ-------------------------------------------------
ARF1/1-665                                      -----MAASNHSSGKPGGVLSDALCRELWHACAGPLV-TLPREGERVYYFPEGHMEQ-------------------------------------------------
ARF3/1-608                                      SVSPTSSSSASVSVVSSNSAGGGVCLELWHACAGPLI-SLPKRGSLVLYFPQGHLEQ-------------------------------------------------
ARF4/1-788                                      PSSSSSCSSGSSSSSSSTGSASSIYSELWHACAGPLT-CLPKKGNVVVYFPQGHLEQ-------------------------------------------------
ARF5/1-902                                      TSQSTLLEEMKLLKDQSGTRKPVINSELWHACAGPLV-CLPQVGSLVYYFSQGHSEQ-------------------------------------------------
ARF6/1-933                                      ----MRLSSAGFNPQPHEGEKRVLNSELWHACAGPLV-SLPPVGSRVVYFPQGHSEQ-------------------------------------------------
ARF7/1-1165                                     ---MKAPSSNGVSPNPVEGERRNINSELWHACAGPLI-SLPPAGSLVVYFPQGHSEQ-------------------------------------------------
ARF8/1-811                                      -----MKLSTSGLGQQGHEGEKCLNSELWHACAGPLV-SLPSSGSRVVYFPQGHSEQ-------------------------------------------------
ARF9/1-638                                      ---------------MANRGGEYLYDELWKLCAGPLV-DVPQAQERVYYFPQGHMEQ-------------------------------------------------
ARF10/1-693                                     -----------------MEQEKSLDPQLWHACAGSMV-QIPSLNSTVFYFAQGHTEH-------------------------------------------------
ARF11/1-601                                     ------MANVEADFRTSGSNDDELYTELWKACAGPLV-EVPRYGERVFYFPQGHMEQ-------------------------------------------------
ARF12/1-593                                     MESGNVVNAQPELSGIIDGSKSYVYEQLWKLCAGPLC-DIPKLGEKVYYFPQGHIEL-------------------------------------------------
ARF13/1-623                                     MENNGEMNAQPELSGIIDITKTYMYEKLWNICAGPLC-VLPKPGEKVYYFPQGHIEL-------------------------------------------------
ARF14/1-605                                     MESGNVVNTQPELSGIIDGSKSYMYEQLWKLCAGPLC-DIPKLGEKVYYFPQGHIEL-------------------------------------------------
ARF15/1-598                                     METGNVVNAQPELSGIIDRSKSYMYEQLWKLCAGPLC-DIPKLGEKVYYFPQGNIEL-------------------------------------------------
ARF16/1-670                                     --------MINVMNPMKGGTEKGLDPQLWHACAGGMV-RMPPMNSKVFYFPQGHAEN-------------------------------------------------
ARF17/1-585                                     --------MSPPSATAGDINHREVDPTIWRACAGASV-QIPVLHSRVYYFPQGHVEH-------------------------------------------------
ARF18/1-602                                     --MASVEGDDDFGSSSSRSYQDQLYTELWKVCAGPLV-EVPRAQERVFYFPQGHMEQ-------------------------------------------------
ARF19/1-1086                                    ----MKAPSNGFLPSSNEGEKKPINSQLWHACAGPLV-SLPPVGSLVVYFPQGHSEQ-------------------------------------------------
ARF20/1-615                                     METGNVVNAQPELSGIIDGSKSYMYEQLWKLCAGPLC-DIPKLGENVYYFPQGNIEL-------------------------------------------------
ARF21/1-606                                     MESGNIVNAQPKLSGIIDGSKSYMYEQLWKLCAGPLC-DIPKLGENVYYFPQGNIEL-------------------------------------------------
ARF22/1-600                                     MESGNIVNAQPELSGIIDGSKSYMYEQLWKLCAGPLC-DIPKLGEKIYYFPQGNIEL-------------------------------------------------
ARF23/1-222                                     MESGNVVNVQSELSGIIDGSKSYMYEQLWKLCAGPLC-DIPKLGEKVYYFPQGHIEL-------------------------------------------------
ARF2/1-859                                      GEGQKSNSTRSAAAERALDPEAALYRELWHACAGPLV-TVPRQDDRVFYFPQGHIEQ-------------------------------------------------
PoptrARF8.1/1-827                               --MKLSTSGLGGQQAGQEGEKKCLNSELWHACAGPLV-SLPTAGSRVVYFPQGHSEQ-------------------------------------------------
PoptrARF1.1/1-660                               ------MNHTSGGNPHPGGCNDALYKELWHACAGPLV-TLPCEGERVYYFPQGHMEQ-------------------------------------------------
PoptrARF7.3/1-1113                              -MKSPANGAAAAVTNGEGVEKKSINPELWQACAGPLV-NLPAAGTHVVYFPQGHSEQ-------------------------------------------------
PoptrARF2.3/1-792                               NKRNGIARGSSSFPVAGEGSEDALYKELWHACAGPLV-TVPRQGELVYYFPQGHIEQ-------------------------------------------------
PoptrARF5.1/1-933                               QTNLLEEMKLLKEFQDQSGTRKAINSELWYACAGPLV-SLPQVGSLVYYFPQGHSEQ-------------------------------------------------
PoptrARF5.2/1-944                               QTNLLEEMKLLKEFQDQSGIRKAINSELWYACAGPLV-SLPQVGSLVYYFPQGHSEQ-------------------------------------------------
PoptrARF7.4/1-1137                              ATGAGGTATTSTATNGEGAEKKSINPELWQACAGPLV-NLPAAGTHVVYFPQGHSEQ-------------------------------------------------
PoptrARF16.1/1-669                              ---------------MRVAEKCLDSQLWHACAGSMV-QMPAVNSKVFYFPQGHAEH-------------------------------------------------
PoptrARF1.2/1-662                               -MAFAAMNHTSGGNPHAGGCNDALYKELWHACAGPLV-TLPREGELVYYFPQGHMEQ-------------------------------------------------
PoptrARF9.1/1-666                               AMNRGGGGGSFSQANSEGCGEDDLYTELWKACAGPLV-DVPKRGERVFYFPQGHMEQ-------------------------------------------------
PoptrARF2.1/1-854                               SEAMEGQKGHSTHPSSARDAETALYNELWHACAGPLV-TVPREGDHVFYFPQGHLEQ-------------------------------------------------
PoptrARF16.3/1-700                              -----MITFMEAKEILKEEADKCLDSQLWHACAGGMV-QMPAVNSKVFYFPQGHAEH-------------------------------------------------
PoptrARF3.1/1-709                               SSPSSSSAASALSASGSGSGTSPVCLELWHACAGPLI-SLPKRGSIVVYVPQGHLEQ-------------------------------------------------
PoptrARF6.2/1-914                               ----MRHSSASFNPQTQEGEKRVLNSELWHACAGPLV-SLPAVGSRVVYFAQGHSEQ-------------------------------------------------
PoptrARF6.4/1-953                               RLSSSSSSTTGFNQVTQEGDNKCLNSELWHACAGPLV-SLPHVGSRAVYFPQGHSEQ-------------------------------------------------
PoptrARF17.1/1-594                              ------------MPQPQLTELSRVDPRFWRACAGSSV-QIPAVNSRVYYFPQGHFEQ-------------------------------------------------
PoptrARF2.4/1-879                               FDFFRFYWIIAVVVLARVGSEDALYKELWHACAGPLV-TVPRQGELVYYFPQGHIEQ-------------------------------------------------
PoptrARF16.2/1-708                              GPMIREFQVMNPPKEQMRVAEKCLDSQLWHACAGSMV-QMPAVNSKVFYFPQGHAEH-------------------------------------------------
PoptrARF2.2/1-852                               AMEGQKNHSTHPSSARVVDAETALYNELWHACAGPLV-TVPREGDRVFYFPQGHIEQ-------------------------------------------------
PoptrARF10.1/1-708                              --------------MKKTDKKSLDPQLWQACAGSMV-QIPPLNTKVFYFPQGHAEH-------------------------------------------------
PoptrARF3.3/1-109                               -----------------------------------MI---------------------------------------------------------------------
PoptrARF9.2/1-670                               GIGGGGGGGSLSQGNSEGCGGDDLYTELWKACAGPLV-DVPKRGERVFYFPQGHMEQ-------------------------------------------------
PoptrARF6.1/1-884                               ----MRHSSAGFNPQTHEVEKRVLNSELWHACAGPLV-SLPAVGSRAVYFPQGHSEQ-------------------------------------------------
PoptrARF4/1-713                                 NSFCTNGDSSCSSNSSSSPVSSSIYLELWHACAGPLT-SLPKKGNVVVYFPQGHLEQ-------------------------------------------------
PoptrARF6.3/1-163                               ----------------------------------------------------------------------------------------------------------
PoptrARF3.2/1-714                               SSPSSSSAASALSASGSGSSTSSVCLELWHACAGPLI-SLPKRGSVVVYFPQGHLEQ-------------------------------------------------
PoptrARF9.3/1-579                               ---------------MGVGCDDLYTELWKACAGPLV-DVPKDGERVFYFPQGHMEQ-------------------------------------------------
PoptrARF6.5/1-907                               MRLSSSSSSTGFNQQTPEGDKKCLNSELWHACAGPLV-SLPHVGSRVVYFPQGHSEQ-------------------------------------------------
PoptrARF9.4/1-632                               -------------MAHAERGDDLYSELWKACAGPLV-DVPKAGERVFYFPQGHMEQ-------------------------------------------------
PoptrARF7.1/1-1047                              ----MKVPSNGFLPNSAEGERKSINSELWHACAGPLV-SLPPVGSLVVYFPQGHSEQ-------------------------------------------------
PoptrARF7.2/1-1093                              ----MKVPSNGFLPNFAEGERKCINSELWHACAGPLV-SLPPVGSLVVYFPQGHSEQ-------------------------------------------------
PoptrARF8.2/1-816                               --MKLSTSGLGGQQAGQEGEKKCLNSELWHACAGPLV-SLPTMGSRVVYFPQGHSEQ-------------------------------------------------
PoptrARF16.6/1-91                               ----------------------------------------------------------------------------------------------------------
PoptrARF16.4/1-701                              ---------MEAKDKLKEEADKCLDSQLWHACAGGMV-QMPAVNSKVFYFPQGHAEH-------------------------------------------------
PoptrARF16.5/1-536                              ----------------------------------------------------------------------------------------------------------
PoptrARF10.2/1-713                              --------------MKETEKKSLDPQLWQACAGSMV-HIPPLNSTVFYFPQGHAEH-------------------------------------------------
PoptrARF17.2/1-592                              -------------MPQLTELSRVDPRIWRACAGSSV-QIPTINSRVYYFPQGHLEQ-------------------------------------------------
PoptrARF2.6/1-724                               VLKTTKCFNNGLPPRNPQENKDDLYTELWHACAGPLV-YVPRAGDKVFYFPQGHMEQ-------------------------------------------------
PoptrARF2.5/1-614                               ---------------------LYTELWYACAGPLV-YVPRVGDKVFYFPQGHLEQ-------------------------------------------------
```

Consensus

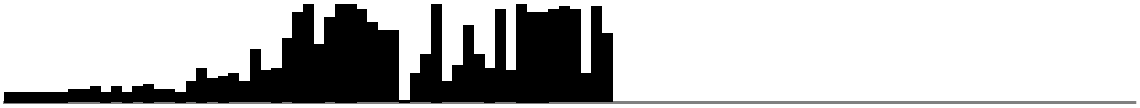

```
MESGSSVSSAGALPGG+EGEKKALYSELWHACAGPLVGSLP+VGSRV+YFPQGHSEQ-------------------------------------------------
```

```
                                                 320       330       340       350       360       370       380       390       400       410       420
LOC_Os01g70270.1|11667.m07038_altsplice/1-809    ---------------------------------VEASMN--QVADSQMRLYDLPSKL---LCRVLNVELKAEQDTDEVYAQVMLMP-EPEQN-----------EM
LOC_Os01g13520.1|11667.m01333/1-700              ---------------------------------LQEPTDP-ALLAEQIKMFQVPYKI---LCKVVNVELKAETETDEVFAQITLQP-DPDQENL---------PT
LOC_Os02g06910.1|11668.m00642/1-909              ---------------------------------VAASTNK-EMESQIPNYPNLPPQL---ICQLHNVTMHADAETDEVYAQMTLQP-LSPQELK-----------
LOC_Os02g35140.1|11668.m03331/1-757              ---------------------------------LEASTD--QQLDQHLPLFNLPSKI---LCKVVNVELRAETDSDEVYAQIMLQP-EADQNE----------LT
LOC_Os02g41800.1|11668.m03990/1-784              ---------------------------------------ALGLAAPELSAARVPALV---PCRVASVRYMADPDTDEVFARIRLVP-LRA-------------AE
LOC_Os04g57610.1|11670.m05695/1-819              ---------------------------------VAASTNK-EVEGHIPNYPNLPAQL---ICQLHDVTMHADVETDEVYAQMTLQP-LNPQEQN-----------
LOC_Os04g43910.1|11670.m04249/1-696              -----------------------------------------ASAAVDLSSARVPPLV---PCRVVAVRFMADAESDEVFAKIRLVPLRPGDAVVD--------VG
LOC_Os04g49230.1|11670.m04840/1-294              -----------------------------------------ATASSSSPTSVSPSAT----------------ASAAASTSASGSPFRSSDGA----------GA
LOC_Os04g36060.1|11670.m03490/1-1674             ---------------------------------LEASTN--QQLDQYLPMFNLPSKI---LCSVVNVELRAEADSDEVYAQIMLQP-EADQSE----------LT
LOC_Os05g43920.1|11682.m04181/1-700              --------------------------------------DGGGGEVPPAGAAAVPPHV---ACRVVGVELRADAATDEVYARLALVAEGEMLQRNFREGG----GE
LOC_Os06g09660.1|11680.m00929/1-1056             ---------------------------------VAASMH--KELDNIPGYPSLPSKL---ICKLLSLTLHADSETDEVYAQMTLQP-VNKYDRDA--------ML
LOC_Os06g46410.1|11680.m04579/1-918              ---------------------------------VAASTNK-EMESQIPNYPNLPPQL---ICQLHNVTMHADAETDEVYAQMTLQP-LSPQELK-----------
LOC_Os06g47150.1|11680.m04656/1-731              ---------------------------------------AQGHGPVEFPGGRVPALV---LCRVAGVRFMADPDTDEVFAKIRLVPVRANEQGYA--------GD
LOC_Os06g48950.1|11680.m04850/1-1116             ----------------------------------------------------------------------ADPETDEVYAQMTLQP-VTSYGKEA--------LQ
LOC_Os08g40900.1|11674.m04106_altsplice/1-1056   -----------------------------------------DADAQIPSYPNLPSKL---ICILHSVTMLADPDTDEVYARMTLQPVSNCDKET---------LL
LOC_Os11g32110.1|11687.m02952/1-812              ---------------------------------VEASTN--QVGEQRMQLYNLPWKI---LCEVMNVELKAEPDTDEVYAQLTLLP-ESKQQEDN--------GS
LOC_Os12g41950.1|11686.m04180/1-900              ---------------------------------VAASTNK-EVDAQIPNYPNLPPQL---ICQLHNVTMHADAETDEVYAQMTLQP-LSPEEQK-----------
LOC_Os12g29520.1|11686.m02861_altsplice/1-842    ---------------------------------VEASTNQ-VAEQQGAPLYNLPWKI---PCKVMNVELKAEPDTDEVYAQLTLLP-EKQQDGNGSGNGNVS-KD
LOC_Os04g56850.1|11670.m05610_altsplice/1-956    ---------------------------------VAATTRK-IPNSRIPNYPNLPSQL---LCQVHNITLHADKDTDEVYAQMTLQPVNSETDVF-----------
LOC_Os05g48870.1|11682.m04683_altsplice/1-696    ----------------------------------------LGDAPAAAAAAAVPPHV---FCRVVDVTLLADAATDEVYAQLSLVPEKEEVARR---------AD
LOC_Os01g48060.1|11667.m04657_altsplice/1-723    ----------------------------------------LGAAPGSGPGAAVPPHV---FCRVVDVSLHADAATDEVYAQVSLVADNEEVERRMREGE----DG
LOC_Os01g54990.1|11667.m05421/1-656              --------------------------------------AGAGGGIRGEVAVALPPHV---ACRVVDVELC-------VFERNLHGG-----------------GI
LOC_Os10g33940.1|11676.m02996/1-699              ----------------------------------ADGGGGAAAAAAELGPRALPPLV---LCRVEGVQFLADRDSDEVYAKIRLAPVAPGEAEFREP------DE
LOC_Os02g04810.1|11668.m00419/1-1094             MRKKVYKSNIILRNAFSMLLETAEYLLNLSFLFQVAASMQK-DVDAHVPSYPNLPSKL---ICLLHGVNLHADPDTDEVYAQMTLQP-VNTYGKEA--------LQ
LOC_Os04g59430.1|11670.m05892/1-530              -----------------------------------------CPAPLPDPLPSAHRFF---LCTITAVDLSADTTTGEPYATISLLPLRHDAPA----------PA
LOC_Os07g08520.1|11673.m00789/1-729              -----------------------------------------CAEDPALLLSRLPDPIHPVPCTVADLVLDVDAESGEAYATISLLP-----------------GS
LOC_Os07g08530.1|11673.m00790/1-408              ------------------------------------FCQFPAPLLEQLAVPG-PRVF---LCTVAAVRLRADALTNEAYAEITLDP-VADHDV----------PR
LOC_Os07g08600.1|11673.m00797/1-525              -----------------------------------------CLSRPQEPLPG--RIF---LCKVTDVRLGA-AATNEALATISLVP-IAADDHAFQ-------LQ
ARF1/1-665                                       ---------------------------------LEASMH--QGLEQQMPSFNLPSKI---LCKVINIQRRAEPETDEVYAQITLLP-ELDQSE----------PT
ARF3/1-608                                       ------------------------------------------APDFSAAIYGLPPHV---FCRILDVKLHAETTTDEVYAQVSLLPESEDIERKVREG-----II
ARF4/1-788                                       ------------------------------------DAMVSYSSPLEIPKFDLNPQI---VCRVVNVQLLANKDTDEVYTQVTLLP-LQEFSMLNGEGKE---VK
ARF5/1-902                                       ---------------------------------VAVSTRR-SATTQVPNYPNLPSQL---MCQVHNVTLHADKDSDEIYAQMSLQPVHSERDV----------FP
ARF6/1-933                                       ---------------------------------VAASTNK-EVDAHIPNYPSLHPQL---ICQLHNVTMHADVETDEVYAQMTLQP-LNAQEQK-----------
ARF7/1-1165                                      ---------------------------------VAASMQ--KQTDFIPSYPNLPSKL---ICMLHNVTLNADPETDEVYAQMTLQP-VNKYDRDA--------LL
ARF8/1-811                                       ---------------------------------VAATTNK-EVDGHIPNYPSLPPQL---ICQLHNVTMHADVETDEVYAQMTLQP-LTPEEQK-----------
ARF9/1-638                                       ---------------------------------LEASTQQ-VDLNTMKPLFVLPPKI---LCNVMNVSLQAEKDTDEVYAQITLIPVGTEVDE----------PM
ARF10/1-693                                      -----------------------------------------AHAPPDFHAPRVPPLI---LCRVVSVKFLADAETDEVFAKITLLP-LPGNDLDLEND-----AV
ARF11/1-601                                      ---------------------------------LVASTNQ-GVVDQEIPVFNLPPKI---LCRVLSVTLKAEHETDEVYAQITLQP-EEDQSE----------PT
ARF12/1-593                                      ---------------------------------VETSTR--EELNELQPICDLPSKL---QCRVIAIHLKVENNSDETYAEITLMP------------------D
ARF13/1-623                                      ---------------------------------IENSTR--DELDHIRPIFDLPSKL---RCRVVAIDRKVDKNTDEVYAQISLMP------------------D
ARF14/1-605                                      ---------------------------------VEASTR--EELNELQPICDFPSKL---QCRVIAIQLKVENNSDETYAEITLMP------------------D
ARF15/1-598                                      ---------------------------------VEASTR--EELNELQPICDLPSKL---QCRVIAIHLKVENNSDETYAKITLMP-DTTVSENL----------
ARF16/1-670                                      -----------------------------------------AYDCVDFGNLPIPPMV---LCRVLAIKYMADAESDEVFAKLRLIP-LKDDEYV---------DH
ARF17/1-585                                      ------------------------------------------CCPLLSTLPSSTSPV---PCIITSIQLLADPVTDEVFAHLILQP-MTQQQFT---------PT
ARF18/1-602                                      ---------------------------------LVASTNQ-GINSEEIPVFDLPPKI---LCRVLDVTLKAEHETDEVYAQITLQP-EEDQSE----------PT
ARF19/1-1086                                     ---------------------------------VAASMQ--KQTDFIPNYPNLPSKL---ICLLHSVTLHADTETDEVYAQMTLQP-VNKYDREA--------LL
ARF20/1-615                                      ---------------------------------VDASTR--EELNELQPICDLPSKL---QCRVIAIHLKVENNSDETYAEITLMP------------------D
ARF21/1-606                                      ---------------------------------VQASTR--EELNELQPICDLPSKL---QCRVIAIHLKVENNSDEIYAEITLMP------------------D
ARF22/1-600                                      ---------------------------------VEASTR--EELNELKPICDLPSKL---QCRVIAIQLKVENNSDETYAEITLMP------------------D
ARF23/1-222                                      ---------------------------------VEASTR--EELNELQPNCDLPSKL---QCRVIAIHLKVENNSDETYVEITLMP------------------D
ARF2/1-859                                       ---------------------------------VEASTN--QAAEQQMPLYDLPSKL---LCRVINVDLKAEADTDEVYAQITLLP-EANQDEN---------AI
PoptrARF8.1/1-827                                ---------------------------------VAATTNK-EVDAHIPNYPSLPPQL---ICQLHNVTMHADVETDEVYAQMTLQP-LTPQEQK-----------
PoptrARF1.1/1-660                                ---------------------------------LEASMH--QGMEQQMPSFNLPSKI---LCKVVNVQRRAEPETDEVYAQITLLP-EPDQSE----------VT
PoptrARF7.3/1-1113                               ---------------------------------VAASLKK-DVNAQIPNYPNLPSKL---LCLLHNVTLHADPETDEVYVQMTLQP-VSSFDKDA--------LL
PoptrARF2.3/1-792                                ---------------------------------VEASTNQ-VADDQQMPAYNLPPKI---LCRVVNVQLKAELDTDEVFAQVILLP-VAEQDVD---------LV
PoptrARF5.1/1-933                                ---------------------------------VAVSTKR-SATSQIPNYPNLPSQL---LCQVHNVTLHADKDTDEIHAQMSLQPVNSEKDV----------FP
PoptrARF5.2/1-944                                ---------------------------------VAVSTRR-SATSQIPNYPNLPSQL---LCQVHNVTLHADKDTDEIYAQMSLQPVNTEKDV----------FP
PoptrARF7.4/1-1137                               ---------------------------------VAASMKK-DVDAQIPNYPNLPSKL---LCLLHNVTLHADPETDEVYAQMTLQP-VSSFDKDA--------LL
PoptrARF16.1/1-669                               -----------------------------------------AQGSVEFGHFQIPALI---PCKVSAIKYMADPETDEVYAKIRLIP-LNNSDLMLGH------GC
PoptrARF1.2/1-662                                ---------------------------------LEASMH--QGMEPQMPLFNLPSKI---LCKVVNVQRRAEPETDEVYAQITLLP-EPDQSE----------VT
PoptrARF9.1/1-666                                ---------------------------------LEASTN--QELNQRVPLFNLPSKI---LCRVINTQLLAEQETDEVYAQITLLP-ESDQIE----------TT
PoptrARF2.1/1-854                                ---------------------------------VEASTN--QVADQQMPLYDLPPKI---LCRVVNVQLKAEPDTDEVFAQVTLLP-LHNQDEN---------AS
PoptrARF16.3/1-700                               -----------------------------------------ACEPVDFRNLPGASHT---LCRVSAIKFMADPETDEVFAKIRLVP-INSNEIDL--------DD
PoptrARF3.1/1-709                                ------------------------------------------LPDLPLGIYDLPPHV---FCRVVDVKLHAEAASDDVYAQVSLVPESEEIEQKLREG-----VF
PoptrARF6.2/1-914                                ---------------------------------VAASTNK-EVDARIPNYPSLPPQL---ICQLHNVTMHADVETDEVYAQLTLQP-LSPQEQK-----------
PoptrARF6.4/1-953                                ---------------------------------VAASTNK-EVDAHIPNYPSLPAQL---ICQLHNVTMHADVETDEVYAQMTLQP-LSPEDKK-----------
PoptrARF17.1/1-594                               -----------------------------------SSSSTAPHPPFLTNLALSKPSI---PCQISAVDFLADPVTDEVFTRLLLIPLDNPFSNLPLSFL----EP
PoptrARF2.4/1-879                                ------------------------VRFHLLPSPVEASTNQ-VADDQQMPAYNLSPKI---LCRVVNVQLKAELDTDEVFAQVILLP-ETQQDVE---------LV
PoptrARF16.2/1-708                               -----------------------------------------AQGSVDFGHFQIPALI---PCKVSAIKYMAEPETDEVYAKIRLTP-SSNSDLMFGD------GC
PoptrARF2.2/1-852                                ---------------------------------VEASTN--QVADQQMPLYNLLPKI---LCRVVNVQLKAEPDTDEVFAQVTLLP-EHNQDES---------VL
PoptrARF10.1/1-708                               -----------------------------------------SQSPVDFPQ-RIPSLV---LCRVASVKFLADPGTDEVFAKISLVP-LPDADLD---------IS
PoptrARF3.3/1-109                                ---------------------------------------------------------------------------------------------------------
PoptrARF9.2/1-670                                ---------------------------------LEASTN--QELNQRVPLFNLPSKI---LCRVIHTQLLAEQDTDEVYAQITLIP-ESDQIE----------PT
PoptrARF6.1/1-884                                ---------------------------------VAASTNK-EVNAQIPSYPSLPAQL---ICQLHNVTMHADVETDEVYAQMTLQP-LSPQEQK-----------
PoptrARF4/1-713                                  ---------------------------------LASSS---PFSHRDMPNFDLHPQI---FCKVVNVQLLANRENDEVYTRLTLLPQPEVVGQDLEGKE----LQ
PoptrARF6.3/1-163                                ---------------------------------------------------------------------------------------------------------
PoptrARF3.2/1-714                                ------------------------------------------LPDLPLAVYDLPSHV---FCRVVDVKLHAEAASDEVYAQVSLVPESEEIEQKLREG-----IF
PoptrARF9.3/1-579                                ---------------------------------LEASTN--QELNQQIPRFNLPPKI---LCRVVNIQLLAEQDTDEVYAQITLHP-EVDQTE----------PT
PoptrARF6.5/1-907                                ---------------------------------VAASTNK-EVDAHIPNYPSLPPQL---ICQLHNVTMHADVETDEVYAQMTLQP-LSQDDQK-----------
PoptrARF9.4/1-632                                ---------------------------------LEASTN--QELNQQIPRFNLPPKI---LCRVVNIQLLAEQDTDEVYAQITLHP-EVDP--------------
PoptrARF7.1/1-1047                               ---------------------------------VAASMQ--KETDFVPSYPNLTSKL---ICMLHNVTLHADVETDEVYAQMTLQP-VSKYDKEA--------LL
PoptrARF7.2/1-1093                               ---------------------------------VAASMQ--KETDFIPSYPNLPSKL---ICMLHNVTLHADVETDEVYAQMTLQP-VSKYEKEA--------LL
PoptrARF8.2/1-816                                ---------------------------------VAATTNK-EVDAHIPNYPSLPPQL---ICQLHNVTMHADVETDEVYAQMTLQP-LTPQEQK-----------
PoptrARF16.6/1-91                                -----------------------------------------------------------------------------------MVP-------------------
PoptrARF16.4/1-701                               -----------------------------------------ACEPVDFRNLPRVSHN---LCRVSDIKFMADPETDEVFAKIRLVP-INSNELDL--------DD
PoptrARF16.5/1-536                               ---------------------------------------------------------------------------------------------------------
PoptrARF10.2/1-713                               -----------------------------------------SQSPVNFPQ-RIPSLI---LCRVATVKFLADPDTDEVYAKIGFVP-LPNTDLDF--------AH
PoptrARF17.2/1-592                               -----------------------------------SSSSTAPHPPFLSNLALSKPLI---SCQISAVDFLADPVTDEVFIRLLLLP-LNNHSCNLPLSFL---EP
PoptrARF2.6/1-724                                ---------------------------------VAARMN--EEGKMEMPIYDLPYKI---LCKVVHVELKAEAGTDEVFARITLLP-VAEEDEL-----------
PoptrARF2.5/1-614                                ---------------------------------VAAFLN--EDSKTAMPIYDLPYKI---LCKVVHVQLKAEAKTDEVFAHITLLP----------------VAE
```

Consensus

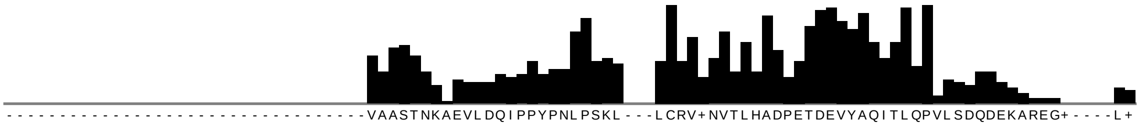

```
---------------------------------VAASTNKAEVLDQIPPYPNLPSKL---LCRV+NVTLHADPETDEVYAQITLQPVLSDQDEKAREG+----L+
```

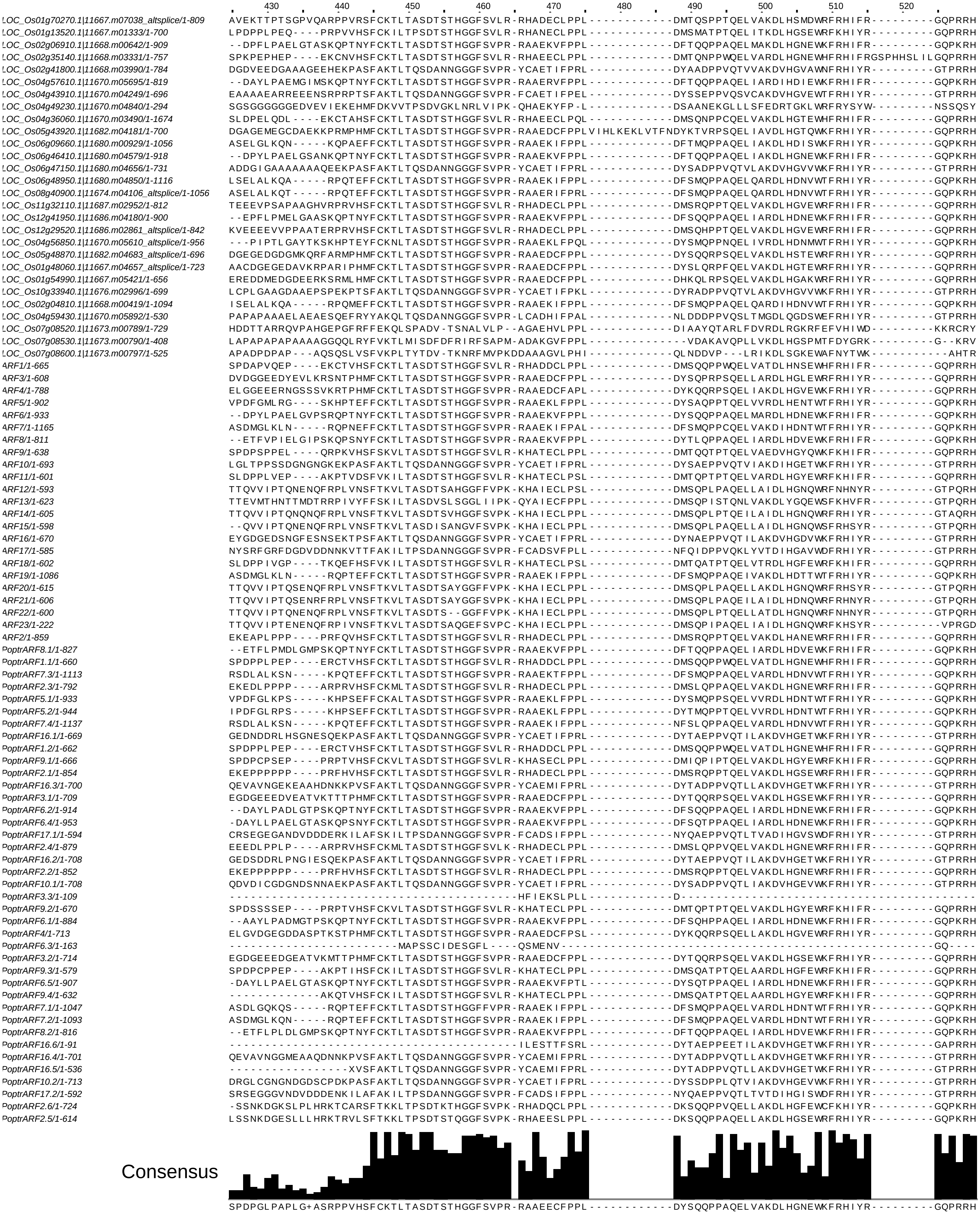
430
440
450
460
470
480
490
500
510
520
Consensus

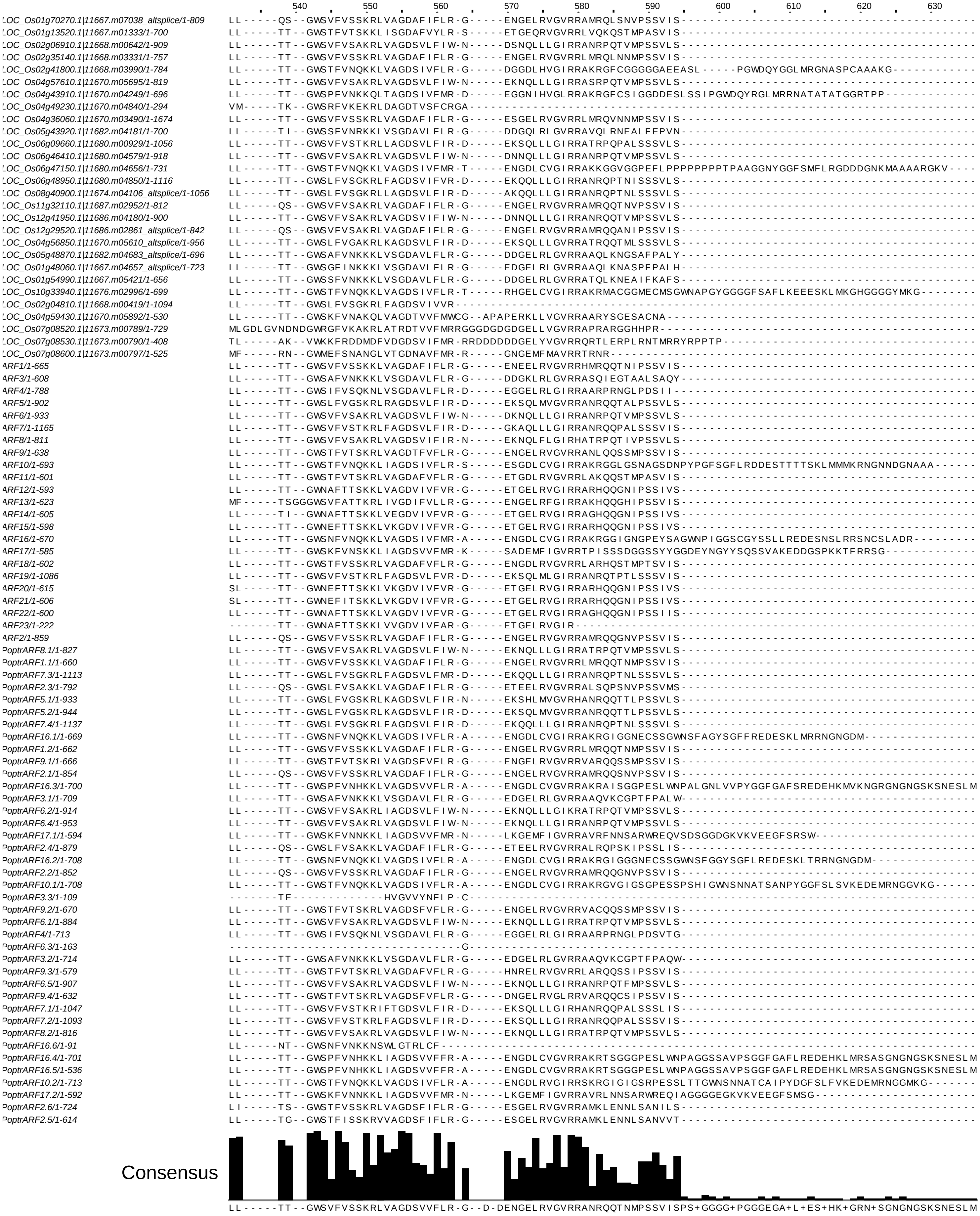
540 550 560 570 580 590 600 610 620 630
LOC_Os01g70270.1|11667.m07038_altsplice/1-809 LL-----QS--GWSVFVSSKRLVAGDAFIFLR-G-----ENGELRVGVRRAMRQLSNVPSSVIS
LOC_Os01g13520.1|11667.m01333/1-700 LL-----TT--GWSTFVTSKKLISGDAFVYLR-S-----ETGEQRVGVRRLVQKQSTMPASVIS
LOC_Os02g06910.1|11668.m00642/1-909 LL-----TT--GWSVFVSAKRLVAGDSVLFIW-N-----DSNQLLLGIRRANRPQTVMPSSVLS
LOC_Os02g35140.1|11668.m03331/1-757 LL-----TT--GWSVFVSSKRLVAGDAFIFLR-G-----ENGELRVGVRRLMRQLNNMPSSVIS
LOC_Os02g41800.1|11668.m03990/1-784 LL-----TT--GWSTFVNQKKLVAGDSIVFLR-G-----DGGDLHVGIRRAKRGFCGGGGGAEEASL-----PGWDQYGGLMRGNASPCAAAKG
LOC_Os04g57610.1|11670.m05695/1-819 LL-----TT--GWSVFVSAKRLVAGDSVLFIW-N-----EKNQLLLGIRRASRPQTVMPSSVLS
LOC_Os04g43910.1|11670.m04249/1-696 LL-----TT--GWSPFVNKKQLTAGDSIVFMR-D-----EGGNIHVGLRRAKRGFCSIGGDDESLSSIPGWDQYRGLMRRNATATATGGRTPP
LOC_Os04g49230.1|11670.m04840/1-294 VM-----TK--GWSRFVKEKRLDAGDTVSFCRGA
LOC_Os04g36060.1|11670.m03490/1-1674 LL-----TT--GWSVFVSSKRLVAGDAFIFLR-G-----ESGELRVGVRRLMRQVNNMPSSVIS
LOC_Os05g43920.1|11682.m04181/1-700 LL-----TI--GWSSFVNRKKLVSGDAVLFLR-G-----DDGQLRLGVRRAVQLRNEALFEPVN
LOC_Os06g09660.1|11680.m00929/1-1056 LL-----TT--GWSVFVSTKRLLAGDSVLFIR-D-----EKSQLLLGIRRATRPQPALSSSVLS
LOC_Os06g46410.1|11680.m04579/1-918 LL-----TT--GWSVFVSAKRLVAGDSVLFIW-N-----DNNQLLLGIRRANRPQTVMPSSVLS
LOC_Os06g47150.1|11680.m04656/1-731 LL-----TT--GWSTFVNQKKLVAGDSIVFLR-T-----ENGDLCVGIRRAKKGGVGGPEFLPPPPPPPPTPAAGGNYGGFSMFLRGDDDGNKMAAAARGKV
LOC_Os06g48950.1|11680.m04850/1-1116 LL-----TT--GWSLFVSGKRLFAGDSVIFVR-D-----EKQQLLLGIRRANRPQTNISSSVLS
LOC_Os08g40900.1|11674.m04106_altsplice/1-1056 LL-----TT--GWSLFVSGKRLLAGDSVLFIR-D-----AKQQLLLGIRRANRQPTNLSSSVLS
LOC_Os11g32110.1|11687.m02952/1-812 LL-----QS--GWSVFVSAKRLVAGDAFIFLR-G-----ENGELRVGVRRAMRQQTNVPSSVIS
LOC_Os12g41950.1|11686.m04180/1-900 LL-----TT--GWSVFVSAKRLVAGDSVIFIW-N-----DNNQLLLGIRRANRPQTVMPSSVLS
LOC_Os12g29520.1|11686.m02861_altsplice/1-842 LL-----QS--GWSVFVSAKRLVAGDAFIFLR-G-----ENGELRVGVRRAMRQQANIPSSVIS
LOC_Os04g56850.1|11670.m05610_altsplice/1-956 LL-----TT--GWSLFVGAKRLKAGDSVLFIR-D-----EKSQLLLGIRRATRPQTMLSSSVLS
LOC_Os05g48870.1|11682.m04683_altsplice/1-696 LL-----TT--GWSAFVNKKKLVSGDAVLFLR-G-----DDGELRLGVRRAAQLKNGSAFPALY
LOC_Os01g48060.1|11667.m04657_altsplice/1-723 LL-----TT--GWSGFINKKKLVSGDAVLFLR-G-----EDGELRLGVRRAAQLKNASPFPALH
LOC_Os01g54990.1|11667.m05421/1-656 LL-----TT--GWSSFVNKKKLVSGDAVLFLR-G-----DDGELRLGVRRATQLKNEAIFKAFS
LOC_Os10g33940.1|11676.m02996/1-699 LL-----TT--GWSTFVNQKKLVAGDSIVFLR-T-----RHGELCVGIRRAKRMACGGMECMSGWNAPGYGGGGFSAFLKEEESKLMKGHGGGGYMKG
LOC_Os02g04810.1|11668.m00419/1-1094 LL-----TT--GWSLFVSGKRLFAGDSVIVVR
LOC_Os04g59430.1|11670.m05892/1-530 LL-----TT--GWSKFVNAKQLVAGDTVVFMWCG--APAPERKLLVGVRRAARYSGESACNA
LOC_Os07g08520.1|11673.m00789/1-729 MLGDLGVNDNDGWRGFVKAKRLATRDTVVFMRRGGGDGDGDGELLVGVRRAPRARGGHHPR
LOC_Os07g08530.1|11673.m00790/1-408 TL-----AK--VWKKFRDDMDFVDGDSVIFMR-RRDDDDDDGELYVGVRRQRTLERPLRNTMRRYRPPTP
LOC_Os07g08600.1|11673.m00797/1-525 MF-----RN--GWMEFSNANGLVTGDNAVFMR-R-----GNGEMFMAVRRTRNR
ARF1/1-665 LL-----TT--GWSVFVSSKRLVAGDAFIFLR-G-----ENEELRVGVRRHMRQQTNIPSSVIS
ARF3/1-608 LL-----TT--GWSAFVNKKKLVSGDAVLFLR-G-----DDGKLRLGVRRASQIEGTAALSAQY
ARF4/1-788 LL-----TT--GWSIFVSQKNLVSGDAVLFLR-D-----EGGELRLGIRRAARPRNGLPDSII
ARF5/1-902 LL-----TT--GWSLFVGSKRLRAGDSVLFIR-D-----EKSQLMVGVRRANRQQTALPSSVLS
ARF6/1-933 LL-----TT--GWSVFVSAKRLVAGDSVLFIW-N-----DKNQLLLGIRRANRPQTVMPSSVLS
ARF7/1-1165 LL-----TT--GWSVFVSTKRLFAGDSVLFIR-D-----GKAQLLLGIRRANRQQPALSSSVIS
ARF8/1-811 LL-----TT--GWSVFVSAKRLVAGDSVIFIR-N-----EKNQLFLGIRHATRPQTIVPSSVLS
ARF9/1-638 LL-----TT--GWSTFVTSKRLVAGDTFVFLR-G-----ENGELRVGVRRANLQQSSMPSSVIS
ARF10/1-693 LL-----TT--GWSTFVNQKKLIAGDSIVFLR-S-----ESGDLCVGIRRAKRGGLGSNAGSDNPYPGFSGFLRDDESTTTTSKLMMMKRNGNNDGNAAA
ARF11/1-601 LL-----TT--GWSTFVTSKRLVAGDAFVFLR-G-----ETGDLRVGVRRLAKQQSTMPASVIS
ARF12/1-593 LL-----TT--GWNAFTTSKKLVAGDVIVFVR-G-----ETGELRVGIRRARHQQGNIPSSIVS
ARF13/1-623 MF-----TSGGGWSVFATTKRLIVGDIFVLLR-G-----ENGELRFGIRRAKHQQGHIPSSVIS
ARF14/1-605 LL-----TI--GWNAFTTSKKLVEGDVIVFVR-G-----ETGELRVGIRRAGHQQGNIPSSIVS
ARF15/1-598 LL-----TT--GWNEFTTSKKLVKGDVIVFVR-G-----ETGELRVGIRRARHQQGNIPSSIVS
ARF16/1-670 LL-----TT--GWSNFVNQKKLVAGDSIVFMR-A-----ENGDLCVGIRRAKRGGIGNGPEYSAGWNPIGGSCGYSSLLREDESNSLRRSNCSLADR
ARF17/1-585 LL-----TT--GWSKFVNSKKLIAGDSVVFMR-K-----SADEMFIGVRRTPISSSDGGSSYYGGDEYNGYYSQSSVAKEDDGSPKKTFRRSG
ARF18/1-602 LL-----TT--GWSTFVTSKRLVAGDAFVFLR-G-----ENGDLRVGVRRLARHQSTMPTSVIS
ARF19/1-1086 LL-----TT--GWSVFVSTKRLFAGDSVLFVR-D-----EKSQLMLGIRRANRQTPTLSSSVIS
ARF20/1-615 SL-----TT--GWNEFTTSKKLVKGDVIVFVR-G-----ETGELRVGIRRARHQQGNIPSSIVS
ARF21/1-606 SL-----TT--GWNEFITSKKLVKGDVIVFVR-G-----ETGELRVGIRRARHQQGNIPSSIVS
ARF22/1-600 LL-----TT--GWNAFTTSKKLVAGDVIVFVR-G-----ETGELRVGIRRAGHQQGNIPSSIIS
ARF23/1-222 -------TT--GWNAFTTSKKLVVGDVIVFAR-G-----ETGELRVGIR
ARF2/1-859 LL-----QS--GWSVFVSSKRLVAGDAFIFLR-G-----ENGELRVGVRRAMRQQGNVPSSVIS
PoptrARF8.1/1-827 LL-----TT--GWSVFVSAKRLVAGDSVLFIW-N-----EKNQLLLGIRRATRPQTVMPSSVLS
PoptrARF1.1/1-660 LL-----TT--GWSVFVSSKKLVAGDAFIFLR-G-----ENGELRVGVRRLMRQQTNMPSSVIS
PoptrARF7.3/1-1113 LL-----TT--GWSLFVSGKRLFAGDSVLFMR-D-----EKQQLLLGIRRANRQPTNLSSSVLS
PoptrARF2.3/1-792 LL-----QS--GWSLFVSAKKLVAGDAFIFLR-G-----ETEELRVGVRRALSQPSNVPSSVMS
PoptrARF5.1/1-933 LL-----TT--GWSLFVGSKRLKAGDSVLFIR-N-----EKSHLMVGVRHANRQQTTLPSSVLS
PoptrARF5.2/1-944 LL-----TT--GWSLFVGSKRLKAGDSVLFIR-D-----EKSQLMVGVRRANRQQTTLPSSVLS
PoptrARF7.4/1-1137 LL-----TT--GWSLFVSGKRLFAGDSVLFIR-D-----EKQQLLLGIRRANRQPTNLSSSVLS
PoptrARF16.1/1-669 LL-----TT--GWSNFVNQKKLVAGDSIVFLR-A-----ENGDLCVGIRRAKRGIGGNECSSGWNSFAGYSGFFREDESKLMRRNGNGDM
PoptrARF1.2/1-662 LL-----TT--GWSVFVSSKKLVAGDAFIFLR-G-----ENGELRVGVRRLMRQQTNMPSSVIS
PoptrARF9.1/1-666 LL-----TT--GWSTFVTSKRLVAGDSFVFLR-G-----ENGELRVGVRRVARQQSSMPSSVIS
PoptrARF2.1/1-854 LL-----QS--GWSVFVSSKRLVAGDAFIFLR-G-----ENGELRVGVRRAMRQQSNVPSSVIS
PoptrARF16.3/1-700 LL-----TT--GWSPFVNHKKLVAGDSVVFLR-A-----ENGDLCVGVRRAKRAISGGPESLWNPALGNLVVPYGGFGAFSREDEHKMVKNGRGNGNGSKSNESLM
PoptrARF3.1/1-709 LL-----TT--GWSAFVNKKKLVSGDAVLFLR-G-----EDGELRLGVRRAAQVKCGPTFPALW
PoptrARF6.2/1-914 LL-----TT--GWSVFVSAKRLIAGDSVLFIW-N-----EKNQLLLGIKRATRPQTVMPSSVLS
PoptrARF6.4/1-953 LL-----TT--GWSVFVSAKRLVAGDSVLFIW-N-----EKNQLLLGIRRANRPQTVMPSSVLS
PoptrARF17.1/1-594 LL-----TT--GWSKFVNNKKLIAGDSVVFMR-N-----LKGEMFIGVRRAVRFNNSARWREQVSDSGGDGKVKVEEGFSRSW
PoptrARF2.4/1-879 LL-----QS--GWSLFVSAKKLVAGDAFIFLR-G-----ETEELRVGVRRALRQPSKIPSSLIS
PoptrARF16.2/1-708 LL-----TT--GWSNFVNQKKLVAGDSIVFLR-A-----ENGDLCVGIRRAKRGIGGGNECSSGWNSFGGYSGFLREDESKLTRRNGNGDM
PoptrARF2.2/1-852 LL-----QS--GWSVFVSSKRLVAGDAFIFLR-G-----ENGELRVGVRRAMRQQGNVPSSVIS
PoptrARF10.1/1-708 LL-----TT--GWSTFVNQKKLVAGDSIVFLR-A-----ENGDLCVGIRRAKRGVGIGSGPESSPSHIGWNSNNATSANPYGGFSLSVKEDEMRNGGVKG
PoptrARF3.3/1-109 -------TE------------HVGVVYNFLP-C
PoptrARF9.2/1-670 LL-----TT--GWSTFVTSKRLVAGDSFVFLR-G-----ENGELRVGVRRVACQQSSMPSSVIS
PoptrARF6.1/1-884 LL-----TT--GWSVFVSAKRLVAGDSVLFIW-N-----EKNQLLLGIRRATRPQTVMPSSVLS
PoptrARF4/1-713 LL-----TT--GWSIFVSQKNLVSGDAVLFLR-G-----EGGELRLGIRRAARPRNGLPDSVTG
PoptrARF6.3/1-163 ---------------------------------G
PoptrARF3.2/1-714 LL-----TT--GWSAFVNKKKLVSGDAVLFLR-G-----EDGELRLGVRRAAQVKCGPTFPAQW
PoptrARF9.3/1-579 LL-----TT--GWSTFVTSKRLVAGDAFVFLR-G-----HNRELRVGVRRLARQQSSIPSSVIS
PoptrARF6.5/1-907 LL-----TT--GWSVFVSAKRLVAGDSVLFIW-N-----EKNQLLLGIRRANRPQTFMPSSVLS
PoptrARF9.4/1-632 LL-----TT--GWSTFVTSKRLVAGDSFVFLR-G-----DNGELRVGLRRVARQQCSIPSSVIS
PoptrARF7.1/1-1047 LL-----TT--GWSVFVSTKRIFTGDSVLFIR-D-----EKSQLLLGIRHANRQQPALSSSLIS
PoptrARF7.2/1-1093 LL-----TT--GWSVFVSTKRLFAGDSVLFIR-D-----EKSQLLLGIRRANRQQPALSSSVIS
PoptrARF8.2/1-816 LL-----TT--GWSVFVSAKRLVAGDSVLFIW-N-----EKNQLLLGIRRATRPQTVMPSSVLS
PoptrARF16.6/1-91 LL-----NT--GWSNFVNKKNSWLGTRLCF
PoptrARF16.4/1-701 LL-----TT--GWSPFVNHKKLIAGDSVVFFR-A-----ENGDLCVGVRRAKRTSGGGPESLWNPAGGSSAVPSGGFGAFLREDEHKLMRSASGNGNGSKSNESLM
PoptrARF16.5/1-536 LL-----TT--GWSPFVNHKKLVAGDSVVFFR-A-----ENGDLCVGVRRAKRTSGGGPESLWNPAGGSSAVPSGGFGAFLREDEHKLMRSASGNGNGSKSNESLM
PoptrARF10.2/1-713 LL-----TT--GWSTFVNQKKLVAGDSIVFLR-A-----ENGDLRVGIRRSKRGIGIGSRPESSLTTGWNSNNATCAIPYDGFSLFVKEDEMRNGGMKG
PoptrARF17.2/1-592 LL-----TT--GWSKFVNNKKLIAGDSVVFMR-N-----LKGEMFIGVRRAVRLNNSARWREQIAGGGGEGKVKVEEGFSMSG
PoptrARF2.6/1-724 LI-----TS--GWSTFVSSKRLVAGDSFIFLR-G-----ESGELRVGVRRAMKLENNLSANILS
PoptrARF2.5/1-614 LL-----TG--GWSTFISSKRVVAGDSFIFLR-G-----ESGELRVGVRRAMKLENNLSANVVT
Consensus
LL-----TT--GWSVFVSSKRLVAGDSVVFLR-G--D-DENGELRVGVRRANRQQTNMPSSVISPS+GGGG+PGGGEGA+L+ES+HK+GRN+SGNGNGSKSNESLM

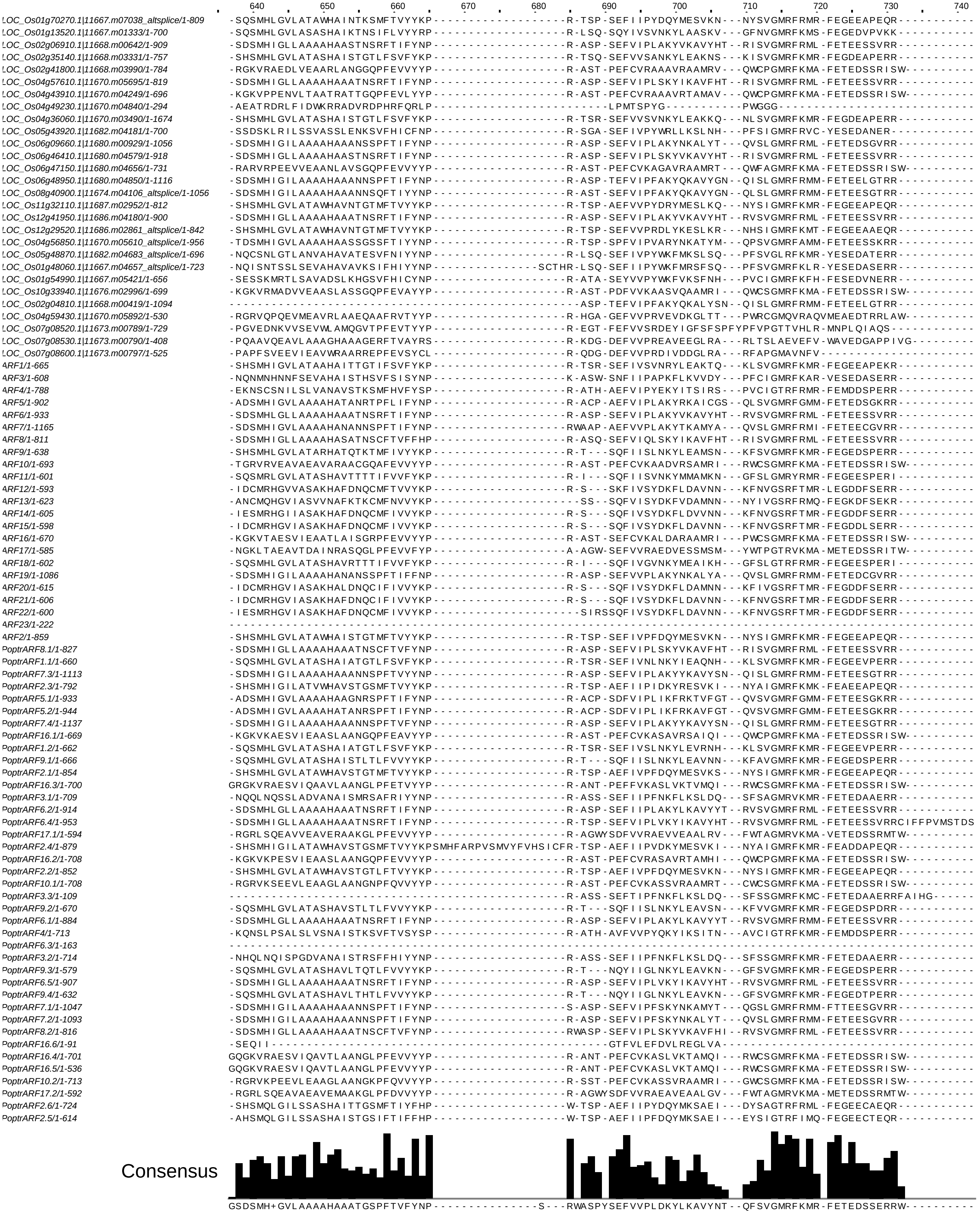
640 650 660 670 680 690 700 710 720 730 740
LOC_Os01g70270.1|11667.m07038_altsplice/1-809 -SQSMHLGVLATAWHAINTKSMFTVYYKP------------------R-TSP-SEFIIPYDQYMESVKN---NYSVGMRFRMR-FEGEEAPEQR----------
LOC_Os01g13520.1|11667.m01333/1-700 -SQSMHLGVLASASHAIKTNSIFLVYYRP------------------R-LSQ-SQYIVSVNKYLAASKV---GFNVGMRFKMS-FEGEDVPVKK----------
LOC_Os02g06910.1|11668.m00642/1-909 -SDSMHIGLLAAAAHAASTNSRFTIFYNP------------------R-ASP-SEFVIPLAKYVKAVYHT--RISVGMRFRML-FETEESSVRR----------
LOC_Os02g35140.1|11668.m03331/1-757 -SHSMHLGVLATASHAISTGTLFSVFYKP------------------R-TSQ-SEFVVSANKYLEAKNS---KISVGMRFKMR-FEGDEAPERR----------
LOC_Os02g41800.1|11668.m03990/1-784 -RGKVRAEDLVEAARLANGGQPFEVVYYP------------------R-AST-PEFCVRAAAVRAAMRV---QWCPGMRFKMA-FETEDSSRISW---------
LOC_Os04g57610.1|11670.m05695/1-819 -SDSMHIGLLAAAAHAATNSRFTIFYNP------------------R-ASP-SEFVIPLSKYIKAVFHT--RISVGMRFRML-FETEESSVRR----------
LOC_Os04g43910.1|11670.m04249/1-696 -KGKVPPENVLTAATRATTGQPFEVLYYP------------------R-AST-PEFCVRAAAVRTAMAV---QWCPGMRFKMA-FETEDSSRISW---------
LOC_Os04g49230.1|11670.m04840/1-294 -AEATRDRLFIDWKRRADVRDPHRFQRLP------------------------LPMTSPYG-----------PWGGG-------------------------
LOC_Os04g36060.1|11670.m03490/1-1674 -SHSMHLGVLATASHAISTGTLFSVFYKP------------------R-TSR-SEFVVSVNKYLEAKKQ---NLSVGMRFKMR-FEGDEAPERR----------
LOC_Os05g43920.1|11682.m04181/1-700 -SSDSKLRILSSVASSLENKSVFHICFNP------------------R-SGA-SEFIVPYWRLLKSLNH---PFSIGMRFRVC-YESEDANER-----------
LOC_Os06g09660.1|11680.m00929/1-1056 -SDSMHIGILAAAAHAAANSSPFTIFYNP------------------R-ASP-SEFVIPLAKYNKALYT---QVSLGMRFRML-FETEDSGVRR----------
LOC_Os06g46410.1|11680.m04579/1-918 -SDSMHIGLLAAAAHAASTNSRFTIFYNP------------------R-ASP-SEFVIPLSKYVKAVYHT--RISVGMRFRML-FETEESSVRR----------
LOC_Os06g47150.1|11680.m04656/1-731 -RARVRPEEVVEAANLAVSGQPFEVVYYP------------------R-AST-PEFCVKAGAVRAAMRT---QWFAGMRFKMA-FETEDSSRISW---------
LOC_Os06g48950.1|11680.m04850/1-1116 -SDSMHIGILAAAAHAAANNSPFTIFYNP------------------R-ASP-TEFVIPFAKYQKAVYGN--QISLGMRFRMM-FETEELGTRR----------
LOC_Os08g40900.1|11674.m04106_altsplice/1-1056 -SDSMHIGILAAAAHAAANNSQFTIYYNP------------------R-AST-SEFVIPFAKYQKAVYGN--QLSLGMRFRMM-FETEESGTRR----------
LOC_Os11g32110.1|11687.m02952/1-812 -SHSMHLGVLATAWHAVNTGTMFTVYYKP------------------R-TSP-AEFVVPYDRYMESLKQ---NYSVGMRFKMR-FEGEEAPEQR----------
LOC_Os12g41950.1|11686.m04180/1-900 -SDSMHIGILAAAAHAATNSRFTIFYNP------------------R-ASP-SEFVIPLAKYVKAVYHT--RVSVGMRFRML-FETEESSVRR----------
LOC_Os12g29520.1|11686.m02861_altsplice/1-842 -SHSMHLGVLATAWHAVNTGTMFTVYYKP------------------R-TSP-SEFVVPRDLYKESLKR---NHSIGMRFKMT-FEGEEAAEQR----------
LOC_Os04g56850.1|11670.m05610_altsplice/1-956 -TDSMHIGVLAAAAHAASSGSSFTIYYNP------------------R-TSP-SPFVIPVARYNKATYM---QPSVGMRFAMM-FETEESSKRR----------
LOC_Os05g48870.1|11682.m04683_altsplice/1-696 -NQCSNLGTLANVAHAVATESVFNIYYNP------------------R-LSQ-SEFIVPYWKFMKSLSQ---PFSVGLRFKMR-YESEDATERR----------
LOC_Os01g48060.1|11667.m04657_altsplice/1-723 -NQISNTSSLSEVAHAVAVKSIFHIYYNP--------------SCTHR-LSQ-SEFIIPYWKFMRSFSQ---PFSVGMRFKLR-YESEDASERR----------
LOC_Os01g54990.1|11667.m05421/1-656 -SESSKMRTLSAVADSLKHGSVFHICYNP------------------R-ATA-SEYVVPYWKFVKSFNH---PVCIGMRFKFH-FESEDVNERR----------
LOC_Os10g33940.1|11676.m02996/1-699 -KGKVRMADVVEAASLASSGQPFEVAYYP------------------R-AST-PDFVVKAASVQAAMRI---QWCSGMRFKMA-FETEDSSRISW---------
LOC_Os02g04810.1|11668.m00419/1-1094 --ASP-TEFVIPFAKYQKALYSN--QISLGMRFRMM-FETEELGTRR----------
LOC_Os04g59430.1|11670.m05892/1-530 -RGRVQPQEVMEAVRLAAEQAAFRVTYYP------------------R-HGA-GEFVVPRVEVDKGLTT---PWRCGMQVRAQVMEAEDTRRLAW---------
LOC_Os07g08520.1|11673.m00789/1-729 -PGVEDNKVVSEVWLAMQGVTPFEVTYYP------------------R-EGT-FEFVVSRDEYIGFSFSPFYPFVPGTTVHLR-MNPLQIAQS----------
LOC_Os07g08530.1|11673.m00790/1-408 -PQAAVQEAVLAAAGHAAAGERFTVAYRS------------------R-KDG-DEFVVPREAVEEGLRA---RLTSLAEVEFV-WAVEDGAPPIVG--------
LOC_Os07g08600.1|11673.m00797/1-525 -PAPFSVEEVIEAVWRAARREPFEVSYCL------------------R-QDG-DEFVVPRDIVDDGLRA---RFAPGMAVNFV--------------------
ARF1/1-665 -SHSMHIGVLATAAHAITTGTIFSVFYKP------------------R-TSR-SEFIVSVNRYLEAKTQ---KLSVGMRFKMR-FEGEEAPEKR----------
ARF3/1-608 -NQNMNHNNFSEVAHAISTHSVFSISYNP------------------K-ASW-SNFIIPAPKFLKVVDY---PFCIGMRFKAR-VESEDASERR----------
ARF4/1-788 -EKNSCSNILSLVANAVSTKSMFHVFYSP------------------R-ATH-AEFVIPYEKYITSIRS---PVCIGTRFRMR-FEMDDSPERR----------
ARF5/1-902 -ADSMHIGVLAAAAHATANRTPFLIFYNP------------------R-ACP-AEFVIPLAKYRKAICGS--QLSVGMRFGMM-FETEESGKRR----------
ARF6/1-933 -SDSMHIGLLAAAAHAATNSRFTIFYNP------------------R-ASP-SEFVIPLAKYVKAVYHT--RVSVGMRFRML-FETEESSVRR----------
ARF7/1-1165 -SDSMHIGVLAAAAHANANNSPFTIFYNP-----------------RWAAP-AEFVVPLAKYTKAMYA---QVSLGMRFRMI-FETEECGVRR----------
ARF8/1-811 -SDSMHIGLLAAAAHASATNSCFTVFFHP------------------R-ASQ-SEFVIQLSKYIKAVFHT--RISVGMRFRML-FETEESSVRR----------
ARF9/1-638 -SHSMHLGVLATARHATQTKTMFIVYYKP------------------R-T---SQFIISLNKYLEAMSN---KFSVGMRFKMR-FEGEDSPERR----------
ARF10/1-693 -TGRVRVEAVAEAVARAACGQAFEVVYYP------------------R-AST-PEFCVKAADVRSAMRI---RWCSGMRFKMA-FETEDSSRISW---------
ARF11/1-601 -SQSMRLGVLATASHAVTTTTIFVVFYKP------------------R-I---SQFIISVNKYMMAMKN---GFSLGMRYRMR-FEGEESPERI----------
ARF12/1-593 -IDCMRHGVVASAKHAFDNQCMFIVVYKP------------------R-S---SKFIVSYDKFLDAVNN---KFNVGSRFTMR-LEGDDFSERR----------
ARF13/1-623 -ANCMQHGVIASVVNAFKTKCMFNVVYKP-------------------SS--SQFVISYDKFVDAMNN---NYIVGSRFRMQ-FEGKDFSEKR----------
ARF14/1-605 -IESMRHGIIASAKHAFDNQCMFIVVYKP------------------R-S---SQFIVSYDKFLDVVNN---KFNVGSRFTMR-FEGDDFSERR----------
ARF15/1-598 -IDCMRHGVIASAKHAFDNQCMFIVVYKP------------------R-S---SQFIVSYDKFLDAVNN---KFNVGSRFTMR-FEGDDLSERR----------
ARF16/1-670 -KGKVTAESVIEAATLAISGRPFEVVYYP------------------R-AST-SEFCVKALDARAAMRI---PWCSGMRFKMA-FETEDSSRISW---------
ARF17/1-585 -NGKLTAEAVTDAINRASQGLPFEVVFYP------------------A-AGW-SEFVVRAEDVESSMSM---YWTPGTRVKMA-METEDSSRITW---------
ARF18/1-602 -SQSMHLGVLATASHAVRTTTIFVVFYKP------------------R-I---SQFIVGVNKYMEAIKH---GFSLGTRFRMR-FEGEESPERI----------
ARF19/1-1086 -SDSMHIGILAAAAHANANSSPFTIFFNP------------------R-ASP-SEFVVPLAKYNKALYA---QVSLGMRFRMM-FETEDCGVRR----------
ARF20/1-615 -IDCMRHGVIASAKHALDNQCIFIVVYKP------------------R-S---SQFIVSYDKFLDAMNN---KFIVGSRFTMR-FEGDDFSERR----------
ARF21/1-606 -IDCMRHGVIASAKHAFDNQCIFIVVYKP------------------R-S---SQFIVSYDKFLDAVNN---KFNVGSRFTMR-FEGDDFSERR----------
ARF22/1-600 -IESMRHGVIASAKHAFDNQCMFIVVYKP------------------SIRSSQFIVSYDKFLDAVNN---KFNVGSRFTMR-FEGDDFSERR----------
ARF23/1-222 --
ARF2/1-859 -SHSMHLGVLATAWHAISTGTMFTVYYKP------------------R-TSP-SEFIVPFDQYMESVKN---NYSIGMRFKMR-FEGEEAPEQR----------
PoptrARF8.1/1-827 -SDSMHIGLLAAAAHAATNSCFTVFYNP------------------R-ASP-SEFVIPLSKYVKAVFHT--RISVGMRFRML-FETEESSVRR----------
PoptrARF1.1/1-660 -SQSMHLGVLATASHAIATGTLFSVFYKP------------------R-TSR-SEFIVNLNKYIEAQNH---KLSVGMRFKMR-FEGEEVPERR----------
PoptrARF7.3/1-1113 -SDSMHIGILAAAAHAAANNSPFTVYYNP------------------R-ASP-SEFVIPLAKYYKAVYSN--QISLGMRFRMM-FETEESGTRR----------
PoptrARF2.3/1-792 -SHSMHLGILATVWHAVSTGSMFTVYYKP------------------R-TSP-AEFIIPIDKYRESVKI---NYAIGMRFKMK-FEAEEAPEQR----------
PoptrARF5.1/1-933 -ADSMHIGVLAAAAHAAGNRSPFTIFYNP------------------R-ACP-SDFVIPLIKFRKTVFGT--QVSVGMRFGMM-FETEESGKRR----------
PoptrARF5.2/1-944 -ADSMHIGVLAAAAHATANRSPFTIFYNP------------------R-ACP-SDFVIPLIKFRKAVFGT--QVSVGMRFGMM-FETEESGKRR----------
PoptrARF7.4/1-1137 -SDSMHIGILAAAAHAANNSPFTVFYNP------------------R-ASP-SEFVIPLAKYYKAVYSN--QISLGMRFRMM-FETEESGTRR----------
PoptrARF16.1/1-669 -KGKVKAESVIEAASLAANGQPFEAVYYP------------------R-AST-PEFCVKASAVRSAIQI---QWCPGMRFKMA-FETEDSSRISW---------
PoptrARF1.2/1-662 -SQSMHLGVLATASHAIATGTLFSVFYKP------------------R-TSR-SEFIVSLNKYLEVRNH---KLSVGMRFKMR-FEGEEVPERR----------
PoptrARF9.1/1-666 -SQSMHLGVLATASHAISTLTLFVVYYKP------------------R-T---SQFIISLNKYLEAVNN---KFAVGMRFKMR-FEGEDSPERR----------
PoptrARF2.1/1-854 -SHSMHLGVLATAWHAVSTGTMFTVYYKP------------------R-TSP-AEFIVPFDQYMESVKS---NYSIGMRFKMR-FEGEEAPEQR----------
PoptrARF16.3/1-700 GRGKVRAESVIQAAVLAANGLPFETVYYP------------------R-ANT-PEFFVKASLVKTVMQI---RWCSGMRFKMA-FETEDSSRISW---------
PoptrARF3.1/1-709 -NQQLNQSSLADVANAISMRSAFRIYYNP------------------R-ASS-SEFIIPFNKFLKSLDQ---SFSAGMRVKMR-FETEDAAERR----------
PoptrARF6.2/1-914 -SDSMHLGLLAAAAHAATNSRFTIFYNP------------------R-ASP-SEFVIPLAKYVKAVYYT--RVSVGMRFRML-FETEESSVRR----------
PoptrARF6.4/1-953 -SDSMHIGILAAAAHAATNSRFTIFYNP------------------R-TSP-SEFVIPLVKYIKAVYHT--RVSVGMRFRML-FETEESSVRRCIFFPVMSTDS
PoptrARF17.1/1-594 -RGRLSQEAVVEAVERAAKGLPFEVVYYP------------------R-AGWYSDFVVRAEVVEAALRV---FWTAGMRVKMA-VETEDSSRMTW---------
PoptrARF2.4/1-879 -SHSMHIGILATAWHAVSTGSMFTVYYKPSMHFARPVSMVYFVHSICFR-TSP-AEFIIPVDKYMESVKI---NYAIGMRFKMR-FEADDAPEQR----------
PoptrARF16.2/1-708 -KGKVKPESVIEAASLAANGQPFEVVYYP------------------R-AST-PEFCVRASAVRTAMHI---QWCPGMRFKMA-FETEDSSRISW---------
PoptrARF2.2/1-852 -SHSMHLGVLATAWHAVSTGTLFTVYYKP------------------R-TSP-AEFIVPFDQYMESVKN---NYSIGMRFKMR-FEGEEAPEQR----------
PoptrARF10.1/1-708 -RGRVKSEEVLEAAGLAANGNPFQVVYYP------------------R-AST-PEFCVKASSVRAAMRT---CWCSGMRFKMA-FETEDSSRISW---------
PoptrARF3.3/1-109 --R-ASS-SEFTIPFNKFLKSLDQ---SFSSGMRFKMC-FETEDAAERRFAIHG------
PoptrARF9.2/1-670 -SQSMHLGVLATASHAVSTLTLFVVYYKP------------------R-T---SQFIISLNKYLEAVSN---KFVVGMRFKMR-FEGEDSPDRR----------
PoptrARF6.1/1-884 -SDSMHLGLLAAAAHAATNSRFTIFYNP------------------R-ASP-SEFVIPLAKYLKAVYYT--RVSVGMRFRMR-FETEESSVRR----------
PoptrARF4/1-713 -KQNSLPSALSLVSNAISTKSVFTVSYSP------------------R-ATH-AVFVVPYQKYIKSITN---AVCIGTRFKMR-FEMDDSPERR----------
PoptrARF6.3/1-163 --
PoptrARF3.2/1-714 -NHQLNQISPGDVANAISTRSFFHIYYNP------------------R-ASS-SEFIIPFNKFLKSLDQ---SFSSGMRFKMR-FETEDAAERR----------
PoptrARF9.3/1-579 -SQSMHLGVLATASHAVLTQTLFVVYYKP------------------R-T---NQYIIGLNKYLEAVKN---GFSVGMRFKMR-FEGEDSPERR----------
PoptrARF6.5/1-907 -SDSMHIGLLAAAAHAATNSRFTIFYNP------------------R-ASP-SEFVIPLVKYIKAVYHT--RVSVGMRFRML-FETEESSVRR----------
PoptrARF9.4/1-632 -SQSMHLGVLATASHAVLTHTLFVVYYKP------------------R-T---NQYIIGLNKYLEAVKN---GFSVGMRFKMR-FEGEDTPERR----------
PoptrARF7.1/1-1047 -SDSMHIGILAAAAHAAANNSPFTIFYNP------------------S-ASP-SEFVIPFSKYNKAMYT---QGSLGMRFRMM-FTTEESGVRR----------
PoptrARF7.2/1-1093 -SDSMHIGILAAAAHAAANNSPFTIFYNP------------------R-ASP-SEFVIPFSKYNKALYT---QVSLGMRFRMM-FETEESGVRR----------
PoptrARF8.2/1-816 -SDSMHIGLLAAAAHAATNSCFTVFYNP-----------------RWASP-SEFVIPLSKYVKAVFHI--RVSVGMRFRML-FETEESSVRR----------
PoptrARF16.6/1-91 -SEQII---GTFVLEFDVLREGLVA----------------------------------
PoptrARF16.4/1-701 GQGKVRAESVIQAVTLAANGLPFEVVYYP------------------R-ANT-PEFCVKASLVKTAMQI---RWCSGMRFKMA-FETEDSSRISW---------
PoptrARF16.5/1-536 GQGKVRAESVIQAVTLAANGLPFEVVYYP------------------R-ANT-PEFCVKASLVKTAMQI---RWCSGMRFKMA-FETEDSSRISW---------
PoptrARF10.2/1-713 -RGRVKPEEVLEAAGLAANGKPFQVVYYP------------------R-SST-PEFCVKASSVRAAMRT---GWCSGMRFKMA-FETEDSSRISW---------
PoptrARF17.2/1-592 -RGRLSQEAVAEAVEMAAKGLPFDVVYYP------------------R-AGWYSDFVVRAEAVEAALGV---FWTAGMRVKMA-METEDSSRMTW---------
PoptrARF2.6/1-724 -SHSMQLGILSSASHAITTGSMFTIYFHP------------------W-TSP-AEFIIPYDQYMKSAEI---DYSAGTRFRML-FEGEECAEQR----------
PoptrARF2.5/1-614 -AHSMQLGILSSASHAISTGSIFTIFFHP------------------W-TSP-AEFIIPFDQYMKSAEI---EYSIGTRFIMQ-FEGEECTEQR----------
Consensus
GSDSMH+GVLAAAAHAATGSPFTVFYNP--------------S---RWASPYSEFVVPLDKYLKAVYNT--QFSVGMRFKMR-FETEDSSERRW---------

```
                                                      750       760       770       780       790       800       810       820       830       840
LOC_Os01g70270.1|11667.m07038_altsplice/1-809   -----FTGTIIGSENLDPV----WPESSWRSLKVRWDEPS-TIP-RPDR-VSPWKI----EPASSPPVN-----------------------------------
LOC_Os01g13520.1|11667.m01333/1-700             -----FSGTIVGEGDLSL----QWSGSEWKSLKVQWDEVT-NVN-GPER-VSPWEI----ETCDGTAPAINVP-------------------------------
LOC_Os02g06910.1|11668.m00642/1-909             -----YMGTITGISDLDPV---RWMNSHWRSVKVGWDEST-AGE-RQPR-VSLWEI----EPLTTFPMYP----------------------------------
LOC_Os02g35140.1|11668.m03331/1-757             -----FSGTIIGVGSMSTS---PWANSDWRSLKVQWDEPS-VVP-RPDR-VSPWEL----EPLAVSNSQPS---------------------------------
LOC_Os02g41800.1|11668.m03990/1-784             -----FMGTVASVQVADPI---RWPQSPWRLLQVTWDEPD-LLQ-NVKR-VSPWLV----ELVSSMPAINLSSF------------------------------
LOC_Os04g57610.1|11670.m05695/1-819             -----YMGTITEVSDADPV---RWPSSYWRSVKVGWDEST-AGE-RPPR-VSLWEI----EPLTTFPMYP----------------------------------
LOC_Os04g43910.1|11670.m04249/1-696             -----FMGTVAGVQASDPV---RWPQSPWRLLQVTWDEPE-LLQ-NVKR-VCPWLV----ELVSSMPNLHLPSF------------------------------
LOC_Os04g49230.1|11670.m04840/1-294             ----------AGASSCRPR--------------------------RPPR-------------------------------------------------------
LOC_Os04g36060.1|11670.m03490/1-1674            -----FSGTIIGIGSVPAMSKSPWADSDWKSLKVQWDEPS-AIV-RPDR-VSPWEL----EPLDASNPQPPQ--------------------------------
LOC_Os05g43920.1|11682.m04181/1-700             -----SAGLISGISEVDPI---RWPGSRWKCLLVRWDDST-DSS-HQNR-VSPWEI----ERVGGSVSVTHSL-------------------------------
LOC_Os06g09660.1|11680.m00929/1-1056            -----YMGTITGIGDLDPV---RWKNSHWRNLQVGWDEST-ASE-RRTR-VSIWEI----EPVATPFYICPPPF------------------FRPKLPKQPGMP
LOC_Os06g46410.1|11680.m04579/1-918             -----YMGTITGISDLDAA---RWPNSHWRSVKVGWDEST-AGE-RQPR-VSLWEI----EPLTTFPMYP----------------------------------
LOC_Os06g47150.1|11680.m04656/1-731             -----FMGTVSAVQVADPI---RWPNSPWRLLQVSWDEPD-LLQ-NVKR-VSPWLV----ELVSNMPAIHLAPF------------------------------
LOC_Os06g48950.1|11680.m04850/1-1116            -----YMGTITGISDLDPV---RWKNSQWRNLQVGWDESA-AGE-RRNR-VSIWEI----EPVAAPFFICPPPF--------------------FGAKRPRQLD
LOC_Os08g40900.1|11674.m04106_altsplice/1-1056  -----YMGTITGISDLDPV---RWKTSHWRNIQVAWDEAA-PTE-RRTR-VSLWEI----EPIIAPFFIYPSPL------------------FTAKRPRLPGMT
LOC_Os11g32110.1|11687.m02952/1-812             -----FTGTIVGMGDSDPA---GWPESKWRSLKVRWDEAS-SIP-RPER-VSPWQI----EPAVSPPVN-----------------------------------
LOC_Os12g41950.1|11686.m04180/1-900             -----YMGTITSISDLDSV---RWPNSHWRSVKVGWDEST-TGD-KQPR-VSLWEI----EPLTTFPMYP----------------------------------
LOC_Os12g29520.1|11686.m02861_altsplice/1-842   -----FTGTIVGVGDSDPS---GWADSKWRSLKVRWDEAA-SVP-RPDR-VSPWQI----EPANSPSPVN----------------------------------
LOC_Os04g56850.1|11670.m05610_altsplice/1-956   -----YTGTVVGISDYDPM---RWPNSKWRNLQVEWDEHG-YGE-RPER-VSIWDI----ETPENTLVFPSS--------------------------------
LOC_Os05g48870.1|11682.m04683_altsplice/1-696   -----YTGIITGSGDTDPM----WHGSKWKCLLVRWDDDA-EFR-RPNR-VSPWEI----ELTSSVSGSHLS--------------------------------
LOC_Os01g48060.1|11667.m04657_altsplice/1-723   -----RTGIIIGSREADPM----WHGSKWKCLVVKWDDDV-ECR-RPNG-VSPWEI----ELSGSVSGSHLS--------------------------------
LOC_Os01g54990.1|11667.m05421/1-656             ------SGMIAGVSEVDPI---RWPGSKWRSLLVRWEDAT-DCN-SQNR-VSPWEI----EIVGGSISVAHSLS------------------------------
LOC_Os10g33940.1|11676.m02996/1-699             -----FMGTISSVQVADPN---RWPNSPWRLLQVTWDEPD-LLQ-NVKC-VSPWLV----ELVSSIPPIHLGPF------------------------------
LOC_Os02g04810.1|11668.m00419/1-1094            -----YMGTITGISDLDPV---------------GWDESA-AGE-RRNR-VSIWEI----EPVAAPFFLCPQPF---------------------FGVKRPRQL
LOC_Os04g59430.1|11670.m05892/1-530             -----LNGTLTNLRH----------QQIWRTLEVEWDASAASSS-MKNRFVNPWQV----QPVDF---------------------------------------
LOC_Os07g08520.1|11673.m00789/1-729             -----ISGTVRTFDHLRP----------WRMLEVDWDQAASPISYRIHRQVNSWQVLRQPQPAATTSAVRIRDAI-------------VA--------------
LOC_Os07g08530.1|11673.m00790/1-408             -----PRGKVTAIAT----------GQLWRNLEIVWDGNS-----EMDMSANFWQV----RPVEEVDISPS---------------------------------
LOC_Os07g08600.1|11673.m00797/1-525             -----------------------------------------------------WAV------------------------------------------------
ARF1/1-665                                      -----FSGTIVGVQENKSS---VWHDSEWRSLKVQWDEPS-SVF-RPER-VSPWEL----EPLVANSTPSSQ--------------------------------
ARF3/1-608                                      -----SPGIISGISDLDPI---RWPGSKWRSLLVRWDDIV-ANG-HQQR-VSPWEI----EPSGSISNSG----------------------------------
ARF4/1-788                                      -----CAGVVTGVCDLDPY---RWPNSKWRCLLVRWDESF-VSD-HQER-VSPWEI----DPSVSLPHLS----------------------------------
ARF5/1-902                                      -----YMGTIVGISDLDPL---RWPGSKWRNLQVEWDEPG-CND-KPTR-VSPWDI----ETPESLFIFPSL--------------------------------
ARF6/1-933                                      -----YMGTITGICDLDPT---RWANSHWRSVKVGWDEST-AGE-RQPR-VSLWEI----EPLTTFPMYP----------------------------------
ARF7/1-1165                                     -----YMGTVTGISDLDPV---RWKNSQWRNLQIGWDESA-AGD-RPSR-VSVWDI----EPVLTPFYICPPPF------------------FRPRFSGQPGMP
ARF8/1-811                                      -----YMGTITGISDLDSV---RWPNSHWRSVKVGWDEST-AGE-RQPR-VSLWEI----EPLTTFPMYP----------------------------------
ARF9/1-638                                      -----YSGTVIGVKDCSP----HWKDSKWRCLEVHWDEPA-SIS-RPNK-VSPWEI----EPFVNSENVP----------------------------------
ARF10/1-693                                     -----FMGTVSAVQVADPI---RWPNSPWRLLQVAWDEPD-LLQ-NVKR-VSPWLV----ELVSNMPTIHLSP-------------------------------
ARF11/1-601                                     -----FTGTIIGSGDLSS----QWPASKWRSLQIQWDEPS-SIQ-RPNK-VSPWEI----EPFSPSALTPTP--------------------------------
ARF12/1-593                                     -----CFGTIIGVSDFSP----HWKCSEWRSLEVQWDEFT-SFP-GPKK-VSPWDI----EHLMPAINVP----------------------------------
ARF13/1-623                                     -----YDGTIIGVNDMSP----HWKDSEWRSLKVQWDELS-PFL-RPNQ-VSPWDI----EHLIPSSDIS----------------------------------
ARF14/1-605                                     -----SFGTIIGVSDFSP----HWKCSEWRSLEVQWDEFA-SFP-RPNQ-VSPWDI----EHLTPWSNVS----------------------------------
ARF15/1-598                                     -----YFGTIIGVSNFSP----HWKCSDWRSLEVQWDEFA-SFL-RPNK-VSPWEI----EHLMPALNVP----------------------------------
ARF16/1-670                                     -----FMGTVSAVNVSDPI---RWPNSPWRLLQVAWDEPD-LLQ-NVKR-VNPWLV----ELVSNVHPIPLTSF------------------------------
ARF17/1-585                                     -----FQGIVSSTYQETG----PWRGSPWKQLQITWDEPE-ILQ-NVKR-VNPWQV----EIAAHATQLHTP--------------------------------
ARF18/1-602                                     -----FTGTIVGSGDLSS----QWPASKWRSLQVQWDEPT-TVQ-RPDK-VSPWEI----EPFLATSPISTPA-------------------------------
ARF19/1-1086                                    -----YMGTVTGISDLDPV---RWKGSQWRNLQVGWDEST-AGD-RPSR-VSIWEI----EPVITPFYICPPPF------------------FRPKYPRQPGMP
ARF20/1-615                                     -----YFGTIIGVNDFSP----HWKCSEWRSLEVQWDEFA-SFS-RPNK-VSPWEI----EHLMSALNVP----------------------------------
ARF21/1-606                                     -----YFGTIIGVSDFSP----HWKCSEWRSLEVQWDEFA-SFS-RPNK-VSPWEI----EHLVPALNVP----------------------------------
ARF22/1-600                                     -----YFGTIIGVSDFSP----HWKCSEWRNLEVQWDEFA-SFS-RPNK-VSPWEI----EHLMPALNVP----------------------------------
ARF23/1-222                                     --------------------------------------------------------------------------------------------------------
ARF2/1-859                                      -----FTGTIVGIEESDPT---RWPKSKWRSLKVRWDETS-SIP-RPDR-VSPWKV----EPALAPPALS----------------------------------
PoptrARF8.1/1-827                               -----YMGTITGISDLDPV---RWPNSHWRSVKVGWDEST-AGE-RQPR-VSLWEI----EPLTTFPMYP----------------------------------
PoptrARF1.1/1-660                               -----FSGTIVGVGDNISS---GWADSEWRSLKVHWDEPS-SIL-RPER-VSPWDL----EPLVATTPSN----------------------------------
PoptrARF7.3/1-1113                              -----HMGTITGISDLDAV---RWKNSQWRNLQVGWDEST-AGE-RRNR-VSIWEI----EPVTAPFFICPPPF-----------------FRSKHPRQPGMPD
PoptrARF2.3/1-792                               -----FSGTVIGVEEADPK---KWPRSKWRCLKVRWDETS-PVH-RPDR-VSPWKI----ERALAPSLDPVPGC------------------------------
PoptrARF5.1/1-933                               -----YMGTIVGISDLDPL---RWPGSKWRNLQVEWDEPG-CSD-KQNR-VSSWEI----ETPESLFIF-----------------------------------
PoptrARF5.2/1-944                               -----YMGTIVGISDLDPL---RWPGSKWRNLQVEWDEPG-CSD-KQNR-VSSWEI----ETPESLFIF-----------------------------------
PoptrARF7.4/1-1137                              -----HMGTITGISDLDAV---RWKNSQWRNLQVGWDEST-AGE-RRNR-VSIWEI----EPVTAPFFICPPPFLDLSVQGNQECQWMASSGLYYQIDKTVLLWD
PoptrARF16.1/1-669                              -----FMGTISSVQVADPI---RWPNSPWRLLQVAWDEPD-LLH-NVKR-VSPWLV----ELVSNMPAIHLSPF------------------------------
PoptrARF1.2/1-662                               -----FSGTIVGVGDNISS---GWADSDWRSLKVQWDEPS-SIM-RPER-VSHWEL----EPLVATTPSN----------------------------------
PoptrARF9.1/1-666                               -----FSGTIVGVEDFSP----HWNDSKWRSLKVQWDEPA-SIS-RPDR-VSPWEI----EPCVASVPANLS--------------------------------
PoptrARF2.1/1-854                               -----FTGTIVGIEDADPS---RWKDSKWRCLKVRWDETS-TIP-RPDR-VSPWKI----EPALAPPALN----------------------------------
PoptrARF16.3/1-700                              -----FMGTVCSVQDADPL---CWPGSPWRLLQVTWDEPD-LLQ-NVKR-VSPWLV----ELASHMSAIHLSPF------------------------------
PoptrARF3.1/1-709                               -----YTGLITGISELDPT---RWPGSKWKCLLVRWDDTE--AN-RHSR-VSPWEV----EPSGSVSGSGSGSISSSN--------------------------
PoptrARF6.2/1-914                               -----YMGTITGISDLDVA---RWPNSLWRSVKVGWDEST-AGE-RQPR-VSLWEI----EPLTTFPMYP----------------------------------
PoptrARF6.4/1-953                               IMFDSRYMGTITGISDLDPA---RWPNSHWRSVKVGWDEST-AGE-RQPR-VSLWEI----EPLTTFPMYP----------------------------------
PoptrARF17.1/1-594                              -----FQGTVSGTGLPDCG---AWRGSPWRMLQITWDEPE-VLQ-NVKR-VSPWQV----EFVATTLPLQDA--------------------------------
PoptrARF2.4/1-879                               -----FSGTVIGVEEADPK---KWPRSNWRCLKVHWDETS-PVH-RPDR-VSPWKV----EPALAPSMDPV---------------------------------
PoptrARF16.2/1-708                              -----FMGTISSVQFADPI---RWPNSPWRLLQVAWDEPD-LLQ-NVKR-VSPWLA----ELVSNMPAIHLSPF------------------------------
PoptrARF2.2/1-852                               -----FTGTIVGIEDADPG---RWKNSKWRCLKVRWDETS-TMP-RPER-VSPWKI----EPALAPPALN----------------------------------
PoptrARF10.1/1-708                              -----FMGTVASVQVADPD---RWPNSPWRLLQVTWDEPD-LLQ-TVKC-VSPWLV----ELVSNMPVIHLSPF------------------------------
PoptrARF3.3/1-109                               -----YTGIITGVSELDPA---RWPGSKWKCLLVSW--------------------------------------------------------------------
PoptrARF9.2/1-670                               -----FSGTIVGVEDFSP----HWNDSKWRSLKVQWDEPA-PIP-RPDR-VSPWEI----EPYLSS--------------------------------------
PoptrARF6.1/1-884                               -----YMGTITGISDLDVV---RWPNSQWRSVKVGWDEST-DGE-RQPR-VSLWEI----EPLTTFPTYP----------------------------------
PoptrARF4/1-713                                 -----CSGVVTGTADLDPY---KWPNSKWRCLMVRWDEDV-ISD-HQER-VSPWEI----DASVSLPPLI----------------------------------
PoptrARF6.3/1-163                               ---------------------------------------------NPPS-------------------------------------------------------
PoptrARF3.2/1-714                               -----YTGIITGVSELDPA---RWPGSKWKCLLVRWDDRE--AN-RLSR-VSPWEV----EPSGSGSISSSN--------------------------------
PoptrARF9.3/1-579                               -----FTGTIVGVGDISP----EWSGSIWRSLKIQWDEPA-TIQ-RPER-VSPWDI----EPFAAPASPNL---------------------------------
PoptrARF6.5/1-907                               -----YMGTITGISDLDPV---RWPNSHWRSVKVGWDEST-AGE-RQPR-VSLWEI----EPLTTFPMYP----------------------------------
PoptrARF9.4/1-632                               -----FTGTIVGVGDISP----EWSGSIWRSLKIQWDEPA-TIQ-RPER-VSPWDI----EPFAAPASPNL---------------------------------
PoptrARF7.1/1-1047                              -----YMGTITGISDLDPV---RWKNSQWRNLQVGWDEST-ASE-RPNR-VSIWEI----EPVVTPFYICPPPF------------------FRPKFPKQPGMP
PoptrARF7.2/1-1093                              -----YMGTITGISDMDPV---RWKNSQWRNLQVGWDEST-AGE-RPSR-VSIWEI----EPVVTPFYIYPPPF------------------FRPKFPKQPGMP
PoptrARF8.2/1-816                               -----YMGTITGTSDLDPV---RWPNSHWRSVKVGWDEST-AGE-RQPR-VSLWEI----EPLTSFPMYP----------------------------------
PoptrARF16.6/1-91                               --------------------------------------------------------------------------------------------------------
PoptrARF16.4/1-701                              -----FMGTVCSVQAADSL---WWPHSPWRLLQVTWDEPD-LLQ-NVKR-VSPWLV----ELASNMAAIHFPPF------------------------------
PoptrARF16.5/1-536                              -----FMGTVCSVQAADSL---WWPHSPWRLLQVTWDEPD-LLQ-NVKR-VSPWLV----ELASNMAAIHFPPF------------------------------
PoptrARF10.2/1-713                              -----FMGTVTSVQVADPV---RWPNSPWRLLQVAWDEPD-LLQ-NVKR-VSPWLV----ELVSNMPVIHLSPF------------------------------
PoptrARF17.2/1-592                              -----FQGTVSGTGLPDSG---AWRGSPWRMLQITWDEPE-VLQ-NAKR-VSPWQV----EFVATTPQLQAA--------------------------------
PoptrARF2.6/1-724                               -----FEGTVVGTEDVDHI---RWPNSEWRILKVKWDAASEPFV-HQER-VSPWNI----EPIEPIRKKHASRLH-------------LHK-------------
PoptrARF2.5/1-614                               -----CEGTVVGAEDVDHI---RWPNSEWRILKVKWDAASDAFV-HPER-VSPWNI----EPIEPIRKKHAS--------------------------------
```

Consensus

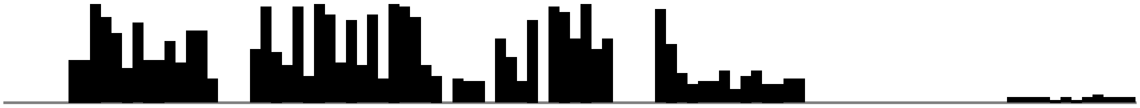

```
-----FMGTITGVSDLDPV---RWPNSKWRSLKVGWDEPT-AGE-RPNR-VSPWEI----EPVS+PP+IPLSPF------------------FRPKFPKQPGMP
```

```
                                                   850       860       870       880       890       900       910       920       930       940       950
LOC_Os01g70270.1|11667.m07038_altsplice/1-809      -----------------------------PLPLSRVKRPR---------PNAPPASPES-----------PI-----------------------LTKEAA-----
LOC_Os01g13520.1|11667.m01333/1-700                -----------------------------LQSATKNKRPR---------EPSE------------------T-----------------------IDLQSL-----
LOC_Os02g06910.1|11668.m00642/1-909                -----------------------------SPFPLRLKRPW---------PTGLPS------------------------------------------LYGGKE-----
LOC_Os02g35140.1|11668.m03331/1-757                -----------------------------PQPPARNKRAR---------PPAS------------------N-----------------------SIAPEL-----
LOC_Os02g41800.1|11668.m03990/1-784                -----------------------------SPPRKKPRILA---------YPEF------------------P-----------------------FEGQLL-----
LOC_Os04g57610.1|11670.m05695/1-819                -----------------------------SLFPLRVKHPW---------YSGVASLHDD-------SNALMW-----------------------LRGVAGEGGFQ
LOC_Os04g43910.1|11670.m04249/1-696                -----------------------------SPPRKKPRNPP---------YAEL------------------P-----------------------LEGQIF-----
LOC_Os04g49230.1|11670.m04840/1-294                ----------------------------------------------------------------------------------------------------------
LOC_Os04g36060.1|11670.m03490/1-1674               -----------------------------PPLRNKRARPPASPSVVAELPPSFDVDSDQISQPSNGNKSDAPGTSSERSPLESQSRQVRSCTKVIMQGMAVGRAVD
LOC_Os05g43920.1|11682.m04181/1-700                -----------------------------SSGSKRTKLHF---------PQGSLDTP----------------------------------------FLNGNG-----
LOC_Os06g09660.1|11680.m00929/1-1056               DDENEVESAFKRAMPWLADDFALKDVQ--SALFPGLSLVQ---------WMAMQQNPQMLTAASQTVQSPYL-----------------------NSNALA-----
LOC_Os06g46410.1|11680.m04579/1-918                -----------------------------SPFPLRLKRPW---------PTGL-----------------PS-----------------------LHGGKD-----
LOC_Os06g47150.1|11680.m04656/1-731                -----------------------------SPPRKKLCVPL---------YPEL------------------P-----------------------IDGQFP-----
LOC_Os06g48950.1|11680.m04850/1-1116               DESSEMENLLKRAMPWLGEEICIKDPQTQNTIMPGLSLVQ---------WMNMNMQQSS------------S-----------------------FANTAM-----
LOC_Os08g40900.1|11674.m04106_altsplice/1-1056     DDETEMDGLLKRAMPWVGEEICKKDLNIQNSVVPGLNLAQ---------WMNMQHS--------------SS-----------------------LPGTVV-----
LOC_Os11g32110.1|11687.m02952/1-812                -----------------------------PLPVPRTKRLR---------PNATALPADS-----------SA-----------------------IAKEAA-----
LOC_Os12g41950.1|11686.m04180/1-900                -----------------------------SAFPLRLKRPW------ASGLPMHGMFNGGGNDDFARYSSLMW-----------------------LRDGNR-----
LOC_Os12g29520.1|11686.m02861_altsplice/1-842      -----------------------------PLPAPRTKRAR---------PNVLASSPDL-----------SA-----------------------VNKEVA-----
LOC_Os04g56850.1|11670.m05610_altsplice/1-956      -----------------------------TLNSKRQCLPG---------YGVSVPGMEIGSANMSSFPRAQG-----------------------NPYGSL-----
LOC_Os05g48870.1|11682.m04683_altsplice/1-696      -----------------------------TPHSKRLKPCL---------PHVNPEYM----------------------------------------VPRGGG-----
LOC_Os01g48060.1|11667.m04657_altsplice/1-723      -----------------------------TPHSKRLKSCF---------PQVNPDIV----------------------------------------LPNGSV-----
LOC_Os01g54990.1|11667.m05421/1-656                -----------------------------ASSSKRTKLCP---------QGNLDVP-----------------------------------------ALYGNG-----
LOC_Os10g33940.1|11676.m02996/1-699                -----------------------------SSPRKKLRVPP---------HPDFPP------------------------------------------FEGHLL-----
LOC_Os02g04810.1|11668.m00419/1-1094               DDESEMENLFKRAMPWLGEEVCIKDTQNQNSTAPGLSLVQ---------WMNMNRQQSS------------S-----------------------LANTAA-----
LOC_Os04g59430.1|11670.m05892/1-530                -----------------------------PPLPMGLKISN------------------------------------------------------------------
LOC_Os07g08520.1|11673.m00789/1-729                -----------------------------TPQVQIMALPR---------------------------------P-----------------------PPPTTT-----
LOC_Os07g08530.1|11673.m00790/1-408                -----------------------------TPPPKRLKNCE------------------------------------------------------------------
LOC_Os07g08600.1|11673.m00797/1-525                ----------------------------------------------------------------------------------------------------------
ARF1/1-665                                         -----------------------------PQPPQRNKRPR---------PPGL------------------P-----------------------SPATGP-----
ARF3/1-608                                         -----------------------------SFVTTGPKRSR---------IGFSSGKPDI------------P-----------------------VSEGIR-----
ARF4/1-788                                         -----------------------------IQSSPRPKRPW---------AGLLDTTPPG-----------NP-----------------------ITKRGG-----
ARF5/1-902                                         -----------------------------TSGLKRQLHPS--YFAGETEWGSLIKRPLI---------RVPD-----------------------SANGIM-----
ARF6/1-933                                         -----------------------------SPFPLRLKRPW---------PPGLPSFHGLKEDDMGMSMSSPL-----------------------MWDRGL-----
ARF7/1-1165                                        DDETDMESALKRAMPWLDNSLEMKDPS--STIFPGLSLVQ---------WMNMQQQNG-------------Q-----------------------LPSAAA-----
ARF8/1-811                                         -----------------------------SLFPLRLKRPW---------HAGTSSLPDG---RGDLGSGLTW-----------------------LRGGGG-----
ARF9/1-638                                         -----------------------------KSVMLKNKRPR---------QVSEVSA-----------------------------------------LDVGIT-----
ARF10/1-693                                        -----------------------------FSPRKKIRIPQ---------PFEF------------------P-----------------------FHGTK------
ARF11/1-601                                        -----------------------------TQQQSKSKRSR---------------------------------P-----------------------ISEITG-----
ARF12/1-593                                        -----------------------------RSFLLKNKRLR--------------------------------EV-----------------------NEIGSS-----
ARF13/1-623                                        -----------------------------QSSLKKKKH-----------WLQL--------------------------------------------NEIGAT-----
ARF14/1-605                                        -----------------------------RSSFLKNKRSR--------------------------------EV-----------------------NEIGSS-----
ARF15/1-598                                        -----------------------------RSSFLKNKRLR--------------------------------EV-----------------------NEFGSS-----
ARF16/1-670                                        -----------------------------SPPRKKMRLPQ---------HPDYNN------------------------------------------LINSIP-----
ARF17/1-585                                        -----------------------------FPPAKRLKYPQ---------PGGG------------------F-----------------------LSGDDG-----
ARF18/1-602                                        -----------------------------QQPQSKCKRSR---------------------------------P-----------------------IEPSVK-----
ARF19/1-1086                                       DDELDMENAFKRAMPWMGEDFGMKDAQ--SSMFPGLSLVQ---------WMSMQQN--------------NP-----------------------LSGSAT-----
ARF20/1-615                                        -----------------------------RSSLLKNKRLR--------------------------------ET-----------------------LEYLLN-----
ARF21/1-606                                        -----------------------------RSSLLKNKRLR--------------------------------EV-----------------------NEFGSS-----
ARF22/1-600                                        -----------------------------RPSLLKNKRLR--------------------------------EV-----------------------NEIGSS-----
ARF23/1-222                                        ----------------------------------------------------------------------------------------------------------
ARF2/1-859                                         -----------------------------PVPMPRPKRPR---------SNIAPSSPDS-----------SM-----------------------LTREGT-----
PoptrARF8.1/1-827                                  -----------------------------SLFPLRLKRPW---------HPGSPSLLDE------ASNGLMW-----------------------LRGGSG-----
PoptrARF1.1/1-660                                  -----------------------------SQPMQRNKRPR---------PSVLP----------------SP-----------------------TANLSA-----
PoptrARF7.3/1-1113                                 DDSTDFDSLFKRTMPWLGDDIYMKD----PQVLPGLSLAQ---RMNMQQNPSLANSMQPNYMQSLSGSVLQN-----------------------LPGGDL-----
PoptrARF2.3/1-792                                  -----------------------------QSKRHRSNMAT--------------------------------SS-----------------------ADSSAP-----
PoptrARF5.1/1-933                                  -----------------------------PSLTSGLKRPLQSGFLGDTEWGGLVKKPLA----------------------------------------LLPGSG-----
PoptrARF5.2/1-944                                  -----------------------------PSLTSGLKRPLQSGFLGDSEWGSLVKKPLS------------W-----------------------LPGSGN-----
PoptrARF7.4/1-1137                                 DDSSDFDSLFKRTMPWLGDEFCMKD----PQALPGLSLVQ---------WMNMQQNPSLANSMQPNYMQSLSGSVLQN-----------------LPGADL-----
PoptrARF16.1/1-669                                 -----------------------------SPPRKKLRLPQ---------PPDF------------------P-----------------------LLGQIP-----
PoptrARF1.2/1-662                                  -----------------------------SQPVQRNKRAR---------PYVIP----------------SP-----------------------TADLSA-----
PoptrARF9.1/1-666                                  -----------------------------QPVQPKNKRPR--------------------------------PP-----------------------IEIPAF-----
PoptrARF2.1/1-854                                  -----------------------------PLPMPRPKRPR---------ANMVPSSPDS-----------SV-----------------------LTREGS-----
PoptrARF16.3/1-700                                 -----------------------------SSPRKKLRLPQ---------HPDF------------------P-----------------------IDGQFP-----
PoptrARF3.1/1-709                                  -----------------------------NSMAPGLKRSR---------SGLPSLKAEF------------P-----------------------IPDGIG-----
PoptrARF6.2/1-914                                  -----------------------------STFPLRLKRPW---------TPGLHSFHGKLLYTILRALMDGI-----------------------KDDDLG-----
PoptrARF6.4/1-953                                  -----------------------------SAFPMRLKRPW---------PSGLPSFHGLKDDDLSINSPMMW-----------------------LQGGVGDLGVH
PoptrARF17.1/1-594                                 -----------------------------SPPMKKLRYPN------------------------------------------------------------------
PoptrARF2.4/1-879                                  -----------------------------SGCRLKRHRPN---------TVTS-----------------SA-----------------------DSSALT-----
PoptrARF16.2/1-708                                 -----------------------------SPPRKKLRLPQ---------PPDF------------------P-----------------------LLGQIP-----
PoptrARF2.2/1-852                                  -----------------------------PLPLPRPKRPR---------ANMVPSSPDS-----------SV-----------------------LTRDGS-----
PoptrARF10.1/1-708                                 -----------------------------SPPRKKLRFPQ---------QLDFP-------------------------------------------LDGQFQ-----
PoptrARF3.3/1-109                                  ----------------------------------------------------------------------------------------------------------
PoptrARF9.2/1-670                                  -----------------------------TASTRNSGLTQ---------SHDL-----------------TQ-----------------------LSVTGE-----
PoptrARF6.1/1-884                                  -----------------------------SPFPLRLKRPW---------PPGLPSFHGL-----------GI-----------------------KDDDLG-----
PoptrARF4/1-713                                    -----------------------------IQSSPRLKKLR---------TGLQAAPPDK------------P-----------------------IAGGGG-----
PoptrARF6.3/1-163                                  ----------------------------------------------------------------------------------------------------------
PoptrARF3.2/1-714                                  -----------------------------NFMAPGLKRSR---------SGLPSSKAEF------------P-----------------------IPDGIG-----
PoptrARF9.3/1-579                                  -----------------------------TQQVMKTKRPR---------PTDI------------------------------------------------------
PoptrARF6.5/1-907                                  -----------------------------SAFPMRLKRPW---------PSGLPSFHGLQDGDLNINSPMMW-----------------------LQGGVGDLGVQ
PoptrARF9.4/1-632                                  -----------------------------TQQVVKSKRPR---------SVDI------------------P-----------------------TSGSIT-----
PoptrARF7.1/1-1047                                 NDESDTENAFKRAVPWLGDEFGKKDAA--SSIFPGLSLVQ---------WMSMQQNNQF-----------QA-----------------------AQSGFF-----
PoptrARF7.2/1-1093                                 DDDSDIENAFKRAMPWLGDEFGMKDPP--SSIFPGLSLVQ---------WMSMQQNNQF-----------PA-----------------------TQSGLF-----
PoptrARF8.2/1-816                                  -----------------------------SLFPLRLKRPW---------HPGSSSLLDS---RDEASNGLIW-----------------------LRGGSG-----
PoptrARF16.6/1-91                                  ----------------------------------------------------------------------------------------------------------
PoptrARF16.4/1-701                                 -----------------------------SSPRKKLRLPQ---------HLDF------------------P-----------------------IDGQFP-----
PoptrARF16.5/1-536                                 -----------------------------SSPRKKLRLPQ---------HLDF------------------P-----------------------IDGQFP-----
PoptrARF10.2/1-713                                 -----------------------------SPPRKKSRFPQ---------QLGF------------------P-----------------------LDLQFQ-----
PoptrARF17.2/1-592                                 -----------------------------FPPMKKLRYPN------------------------------------------------------------------
PoptrARF2.6/1-724                                  -----------------------------MECIADKSLPR---------------------------------------------------------FLISVK-----
PoptrARF2.5/1-614                                  -----------------------------LPTIAHLLSPP---------NPEW------------------N-----------------------FSPIGK-----
```

Consensus

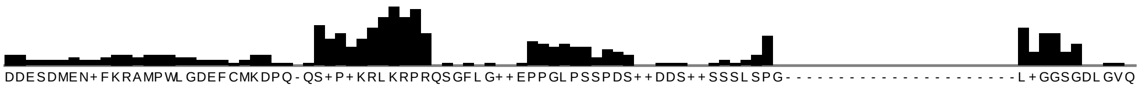

```
DDESDMEN+FKRAMPWLGDEFCMKDPQ-QS+P+KRLKRPRQSGFLG++EPPGLPSSPDS++DDS++SSSLSPG----------------------L+GGSGDLGVQ
```

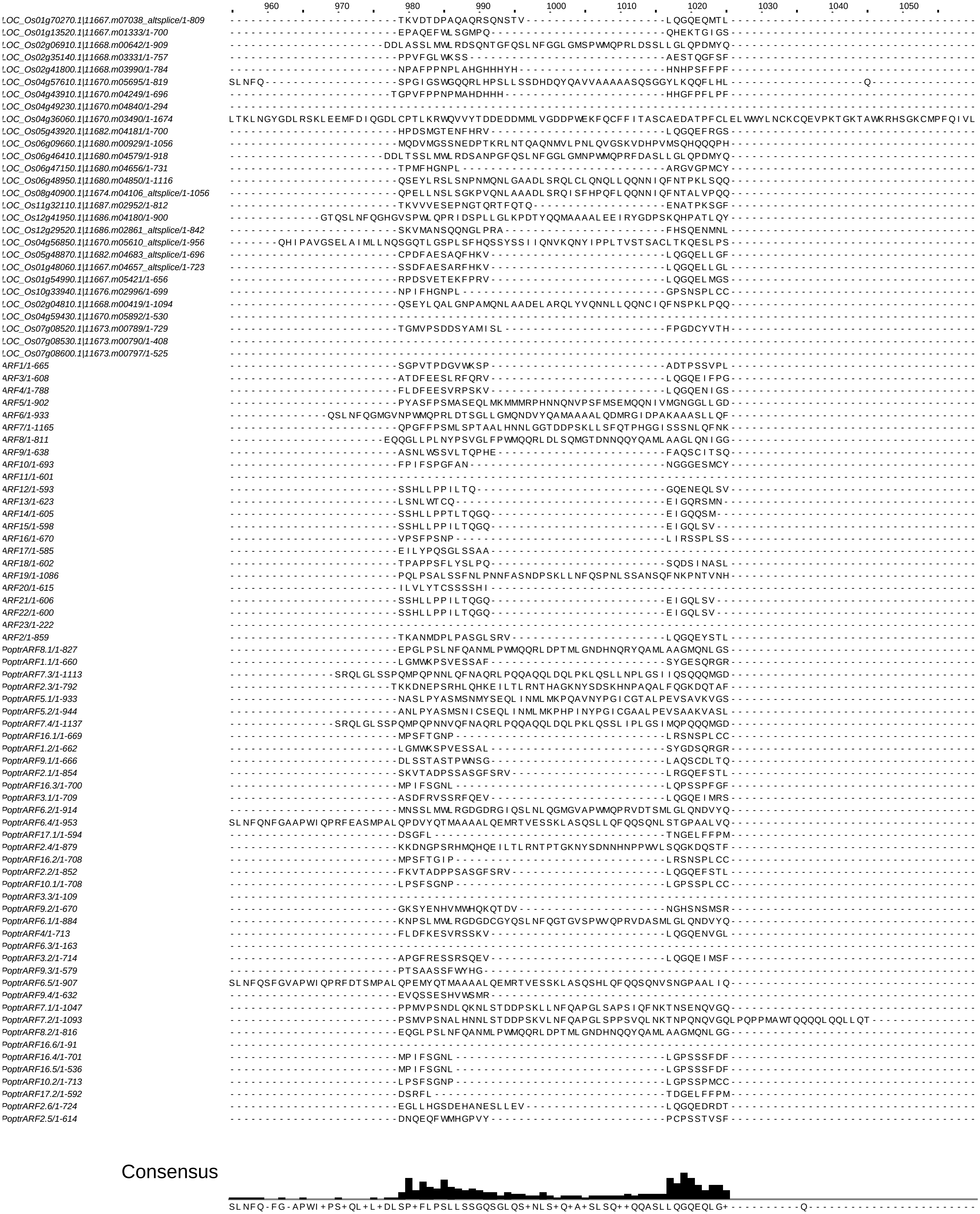

960
970
980
990
1000
1010
1020
1030
1040
1050
Consensus
SLNFQ-FG-APWI+PS+QL+L+DLSP+FLPSLLSSGQSGLQS+NLS+Q+A+SLSQ++QQASLLQGQEQLG+---------Q---------------------

```
                                                               1070      1080      1090      1100      1110      1120      1130      1140      1150      1160
LOC_Os01g70270.1|11667.m07038_altsplice/1-809  -----------------------------------------------RSNLTESNDSDVTAHKPMMWSPSPNAAKAHPLTFQQRPPMDNWMQLGRRETDFKDVRSG
LOC_Os01g13520.1|11667.m01333/1-700            -----------------------------------------------------------SEPNCISGHQVVWPGEHPGYGAVSSSVCQNPLVLESWLKD-------
LOC_Os02g06910.1|11668.m00642/1-909            ---------------------------------------------TIAAAAALQNTTKQVSPAMLQFQQPQNIVGRSSLLSSQILQQAQPQFQQMYHQNINGNSIQ
LOC_Os02g35140.1|11668.m03331/1-757            --------------------------------------------------------------SGLQRTQELY----------------------------------
LOC_Os02g41800.1|11668.m03990/1-784            --------------------------------------------------------PDVSAPAGIQGARHAQFG--------------------------------
LOC_Os04g57610.1|11670.m05695/1-819            ---------------------------------------------QPMQSPQEHCNLNPLLQQQILQQASQQQIINPDAQNIQTMLSPSAIQQQLQQLQQMQQVQN
LOC_Os04g43910.1|11670.m04249/1-696            -------------------------------------------------------PDSSAQPAGIQGARHAQFA--------------------------------
LOC_Os04g49230.1|11670.m04840/1-294            ----------------------------------------------------------------------------------------------------------
LOC_Os04g36060.1|11670.m03490/1-1674           CFGRSLGVLVWCPDLGDSVGGGAERRRTGGVERRRREPVTRRRGGKERRRPSWRALAANRRRGEAANQRRGEAATQRGGSDPAGGLRRRGEAAKRRHGEAANRWRE
LOC_Os05g43920.1|11682.m04181/1-700            -------------------------------------------------------RSHGVVCSESPGVPNFQSPDNRRFSADMRGYMMPASG--------------
LOC_Os06g09660.1|11680.m00929/1-1056           -----------------------------------------------QLSQQQQVQPSQQSSVVLQQHQAQLLQQNAIHLQQQQEHLQRQQSQPAQQLKAASSLHS
LOC_Os06g46410.1|11680.m04579/1-918            ----------------------------------------------TIAATAFQDPTKQVSPTILQFQQPQNIGGRANTLLPSQILQQVQPQFQQQQYLQNINETT
LOC_Os06g47150.1|11680.m04656/1-731            --------------------------------------------------------FPDGTPAGIQGARHAQFG--------------------------------
LOC_Os06g48950.1|11680.m04850/1-1116           -------------------------------------------------MQPVNELAKAGIPLNQLGVSTKPQEQIHDASNLQRQQPSMNHMLPLSQAQTNLGQAQ
LOC_Os08g40900.1|11674.m04106_altsplice/1-1056 ------------------------------------------------NQQTEQLAKVIPTPNQLGSVIIPQKVVQDCNSEQRQHVVTQPVQGSQPNINIPQPQLV
LOC_Os11g32110.1|11687.m02952/1-812            ----------------------------------------------------GNSSELESAQKSIMRPSGFDREKNNTPIQWKLGSDGRMQMSKPESYSEMLSGFQ
LOC_Os12g41950.1|11686.m04180/1-900            -----------------------------------------------QQTHNLNSGLNSLFASHVLGQVQFQPQQSPLQVVQQGHCQNTGDSGFLQGQLPRLQLHN
LOC_Os12g29520.1|11686.m02861_altsplice/1-842  ------------------------------------------------RSRFGDSNELNTSQKLTMWSSGSNQEKNNVSVQRELGSQSWMQMRRPDGSSEILSGFQ
LOC_Os04g56850.1|11670.m05610_altsplice/1-956  ---------------------------------------------DDAQHQFHMANMQNGDLEGSEVQPVIDSISESKLNATSRDPRNTDSYTSRSTSEQNSKGEP
LOC_Os05g48870.1|11682.m04683_altsplice/1-696  ---------------------------------------------------KSHGGTAAATSQPCEARHLQYIDERSCSSDASNSILGVPRLGDR-----------
LOC_Os01g48060.1|11667.m04657_altsplice/1-723  ------------------------------------------------------------------KTRDGTVNTASQATEARNFQYTDERSCSINMSNNILGVPR
LOC_Os01g54990.1|11667.m05421/1-656            -----------------------------------------------------RTHRATCSPQSIDITKSKSFDAWRFLTDTRSCMLGSSTSR-------------
LOC_Os10g33940.1|11676.m02996/1-699            --------------------------------------------------------YPDTAPAGIQGARHAQFG--------------------------------
LOC_Os02g04810.1|11668.m00419/1-1094           ------------------------------------------------MQTMNDLSKAAIPLNQLGAIINPQDQKQDAVNHQRQQNSIQVIPLSQAQSNLVQAQVI
LOC_Os04g59430.1|11670.m05892/1-530            ----------------------------------------------------------------------------------------------------------
LOC_Os07g08520.1|11673.m00789/1-729            -------------------------------------------------------RPLPAARDPVGGQREFCFFDKKLSPSDAAANGGGSGAL-------------
LOC_Os07g08530.1|11673.m00790/1-408            ----------------------------------------------------------------------------------------------------------
LOC_Os07g08600.1|11673.m00797/1-525            ----------------------------------------------------------------------------------------------------------
ARF1/1-665                                     ----------------------------------------------------------------------------------------------------------
ARF3/1-608                                     --------------------------------------------------------FINTCSDGGAGARRGRFK--------------------------------
ARF4/1-788                                     --------------------------------------------------------ASPSQGFDVMNRRILDFAMQSHANPVLVSSRVKDRFG-------------
ARF5/1-902                                     -------------------------------------------------MKMQQPLMMNQKSEMVQPQNKLTVNPSASNTSGQEQNLSQSMSAPAKPENSTLSGCS
ARF6/1-933                                     ----------------------------------------------QNSPGFSMQSPSLVQPQMLQQQLSQQQQQLSQQQQQQQQLSQQQQQQLSQQQQQQLSQQQ
ARF7/1-1165                                    ------------------------------------------------QNQQAPMSQLPQPPTTLSQQQQLQQLLHSSLNHQQQQSQSQQQQQQQQLLQQQQQLQS
ARF8/1-811                                     ------------------------------------------------------GDPLRQQFVQLQEPHHQYLQQSASHNSDLMLQQQQQQQASRHLMHAQTQIMS
ARF9/1-638                                     ----------------------------------------------------------------------------------------------------------
ARF10/1-693                                    -----------------------------------------------------LSNDNNNAPAGIQGARQAQQL--------------------------------
ARF11/1-601                                    ----------------------------------------------------------------------------------------------------------
ARF12/1-593                                    ----------------------------------------------------------------------------------------------------------
ARF13/1-623                                    ----------------------------------------------------------------------------------------------------------
ARF14/1-605                                    ----------------------------------------------------------------------------------------------------------
ARF15/1-598                                    ----------------------------------------------------------------------------------------------------------
ARF16/1-670                                    --------------------------------------------------------VLDNVPVGLQGARH------------------------------------
ARF17/1-585                                    ----------------------------------------------------------------------------------------------------------
ARF18/1-602                                    ----------------------------------------------------------------------------------------------------------
ARF19/1-1086                                   -----------------------------------------------------ISQQMQAQPAMVKSQQQQQQQQQQHQHQQQQLQQQQQLQMSQQQVQQQGIYNN
ARF20/1-615                                    -----------------------------------------------------------LPPILTQGQEIGQLSV-------------------------------
ARF21/1-606                                    ----------------------------------------------------------------------------------------------------------
ARF22/1-600                                    ----------------------------------------------------------------------------------------------------------
ARF23/1-222                                    ----------------------------------------------------------------------------------------------------------
ARF2/1-859                                     ------------------------------------------------RTKHTESVECDAPENSVVWQSSADDDKVDVVSGSRRYGSENWMSSARHEPTYTDLLSG
PoptrARF8.1/1-827                              ------------------------------------------------------GDPLRQQFMQLQQPFQYLQQSSGHNPLLQLQQQQQQQAIQQSIPHNILQAQN
PoptrARF1.1/1-660                              ----------------------------------------------------------------------------------------------------------
PoptrARF7.3/1-1113                             -----------------------------------------------ITQQSRQNMMAQTLPSSQVQAQLLQPQTLAQTNNILQQQPSIQSHQLLRNLPQTLHQQQ
PoptrARF2.3/1-792                              --------------------------------------------------DNRKLGPDDKIPQVMHGAKLMNLTTGPGTLHESYESTHPFFELNSDDVDRP-----
PoptrARF5.1/1-933                              ---------------------------------------LDVKNMQAAINQTPQLNQSGITPIENQNYSQICLDQSNAMNSYSSKANVAGKSLSLSKVENQASVGG
PoptrARF5.2/1-944                              ----------------------------------------DVKNMQATINQMPQLNQSGVTSVENQNYSQICLDQSDAIISSSLKINVAGKSFSSSEVENQASVGV
PoptrARF7.4/1-1137                             -----------------------------------------------ITQQSRQNLMAQTLPSSQVQAQLLQPQTLAQTNNILQQQPSIQSHQLLRNLPQTLHHQQ
PoptrARF16.1/1-669                             --------------------------------------------------------VSDNIPAGIQGARHAQFG--------------------------------
PoptrARF1.2/1-662                              ----------------------------------------------------------------------------------------------------------
PoptrARF9.1/1-666                              ----------------------------------------------------------------------------------------------------------
PoptrARF2.1/1-854                              ------------------------------------------------RGNFEEGNESDVAEKSVLWPPSADDEKIDVLSSSRRFGSEWWISSARQEPTYTDLLSG
PoptrARF16.3/1-700                             --------------------------------------------------------LPNNIPAGMQGARHAHYG--------------------------------
PoptrARF3.1/1-709                              ---------------------------------------------------------------GIRGS--------------------------------------
PoptrARF6.2/1-914                              -----------------------------------------------TMATAAFQEMRALDPSKSSAASLLQFQQHQNLPIRNAALMQPLMLQQSPSQQAFLQGVQ
PoptrARF6.4/1-953                              ---------------------------------------------------RQMLQQSNLQNAFLQNFQENQASTQTQLLQQQLQQHIQYTDQQQQQQQRHQPQHQ
PoptrARF17.1/1-594                             ----------------------------------------------------------------------------------------------------------
PoptrARF2.4/1-879                              --------------------------------------------------DNRRFEPDDRIPYLMHGPKLMNPTTGTGTLLKSYESSRPFFGLFSDDVDQPSKL--
PoptrARF16.2/1-708                             --------------------------------------------------------VSDNIPAGIQGARHAQFE--------------------------------
PoptrARF2.2/1-852                              ------------------------------------------------RGTFAESNESNAAEKSVMWPSSADDEKIDVLSTSRRFGSERWMSSARHEPTCTDLLSG
PoptrARF10.1/1-708                             --------------------------------------------------------LSDNTPAGIQGARHAQFG--------------------------------
PoptrARF3.3/1-109                              ----------------------------------------------------------------------------------------------------------
PoptrARF9.2/1-670                              ----------------------------------------------------------------------------------------------------------
PoptrARF6.1/1-884                              -----------------------------------------------TMATAAFQEMRTLDPSKSSAASFLQFQQHQNLPTRSAALMQPRVQENKHQSQTPSQSHL
PoptrARF4/1-713                                ----------------------------------------------------------------------------------------------------------
PoptrARF6.3/1-163                              ----------------------------------------------------------------------------------------------------------
PoptrARF3.2/1-714                              ----------------------------------------------------------NALYDGVDGQNQHPSEIRSCFPGYHSSGIAALGSGIRDS---------
PoptrARF9.3/1-579                              ----------------------------------------------------------------------------------------------------------
PoptrARF6.5/1-907                              ---------------------------------------------------RQMLQQSNLQHALLQNFQENQASTQAQFLQQHLQHRNQYTGQQLQQHQPQLQQVQ
PoptrARF9.4/1-632                              ----------------------------------------------------------------------------------------------------------
PoptrARF7.1/1-1047                             -----------------------------------------------LRRPPMAWTQQQQLQQLLQTSINQQQPPYPQQPQPQLQHVVSPNQIPNQNFQKPFVYSQ
PoptrARF7.2/1-1093                             ---------------------------------------------PINQQQPPYPHQQQQQPISQQQQHQHWPQQQQQQQPQLQPPQIRQPPPQIQQHQQQQQIFQ
PoptrARF8.2/1-816                              --------------------------------------------------------GDPLRQQFMQLQQPFQYPQQSSSPNPLLQLQQQHQAMQQSIPHNILQPQN
PoptrARF16.6/1-91                              ----------------------------------------------------------------------------------------------------------
PoptrARF16.4/1-701                             --------------------------------------------------------LPHNTPAGMQGARHAHYG--------------------------------
PoptrARF16.5/1-536                             --------------------------------------------------------LPHNTPAGMQGARHAHYG--------------------------------
PoptrARF10.2/1-713                             --------------------------------------------------------LSDNTPAGIQGARHAQFG--------------------------------
PoptrARF17.2/1-592                             ----------------------------------------------------------------------------------------------------------
PoptrARF2.6/1-724                              ----------------------------------------------------SANQFGAFKPPPVPHLTSPPNPDWNRSPIGRDNQLQFWMGGPI-----------
PoptrARF2.5/1-614                              -------------------------------------------------------------PGGNIAR--------------------------------------
```

Consensus

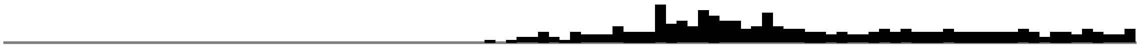

```
----------------------------------------DVKNMQ++RQQF+Q+N+S+N+PAG+QGARHAQFQQQSQL+QQQQQQQQQQQSQQRQQ+QQQLQ+QQ
```

```
                                                   1170       1180       1190       1200       1210       1220       1230       1240       1250       1260       1270
LOC_Os01g70270.1|11667.m07038_altsplice/1-809      SQS--------------------------FGDSPGFFMQN------------------------------------------------------------------
LOC_Os01g13520.1|11667.m01333/1-700                -----------------------------FNSSNKGVSPT------------------------------------------------------------------
LOC_Os02g06910.1|11668.m00642/1-909                GHSQPEY----------------------LQQPLQHCQSF----------------------------------------------------------NEQKPQLQ
LOC_Os02g35140.1|11668.m03331/1-757                -----------------------------PSSPNPIFSTS------------------------------------------------------------------
LOC_Os02g41800.1|11668.m03990/1-784                -----------------------------PSLSDLHLTHL------------------------------------------------------------------
LOC_Os04g57610.1|11670.m05695/1-819                DQKQ-------------------------KIQPDQSYQVP-----------------------------------------------------------------T
LOC_Os04g43910.1|11670.m04249/1-696                -----------------------------SPFPEFHIGNL------------------------------------------------------------------
LOC_Os04g49230.1|11670.m04840/1-294                -----------------------------------------------------------------------------------------------------------
LOC_Os04g36060.1|11670.m03490/1-1674               EAVRRGGDPSERWRTGGVERRRREPATRRTGSSGVGFFHASSSLIAGANGGSDGGGGARTLSWGTWWRRLGRLAPTVALGWCGRWSTREEGIIDVGKRIHVGPPF
LOC_Os05g43920.1|11682.m04181/1-700                -----------------------------PPQRNTEFTYQ------------------------------------------------------------------
LOC_Os06g09660.1|11680.m00929/1-1056               VEQHKLKEQTSGGQVASQAQMLNQI----FPPSSSQLQQL--------------------------------------------GLPKSPTHRQGLTGLPIAGSLQ
LOC_Os06g46410.1|11680.m04579/1-918                IQGHAQSEFLQQQLQRCQS----------FTEQKPQLQTQ-----------------------------------------------------------QQQQESQ
LOC_Os06g47150.1|11680.m04656/1-731                -----------------------------ISLSDLHLNKL------------------------------------------------------------------
LOC_Os06g48950.1|11680.m04850/1-1116               VLVQNQMQQQHASSTQGQQPATSQPLLLPQQQQQQQQQQQ------------------------------------------QQQQQQQQQKLLQQQQQQLLLQQQ
LOC_Os08g40900.1|11674.m04106_altsplice/1-1056     VQAQLQQPQVILQAQLQQPQVVVQAQLQQTQPSVQSHTVL--------------------------------------------QGGLQQIQLLQQQQPHVQHQQI
LOC_Os11g32110.1|11687.m02952/1-812                PPKD-------------------------VQIPQGFCSLP--------------------------------------------------------------EQIT
LOC_Os12g41950.1|11686.m04180/1-900                TQ---------------------------QLLKEQELQQQ----------------------------------------------------------------QR
LOC_Os12g29520.1|11686.m02861_altsplice/1-842      PLKD-------------------------TRNPLSSFPSQ----------------------------------------------------------------IS
LOC_Os04g56850.1|11670.m05610_altsplice/1-956      RGKTRRSKKGLPHKTVSEKSDLSSAPSWICDNQQVGLESK--------------------LVGCDEQVNCGNIEDSSGALTQGNFVGQPHGHQVEQKGVLSPPKVE
LOC_Os05g48870.1|11682.m04683_altsplice/1-696      -----------------------------APLGNPGFSYH------------------------------------------------------------------
LOC_Os01g48060.1|11667.m04657_altsplice/1-723      LGVK-------------------------TPSGNPGFSYH------------------------------------------------------------------
LOC_Os01g54990.1|11667.m05421/1-656                -----------------------------LPVQYSGYTHQ------------------------------------------------------------------
LOC_Os10g33940.1|11676.m02996/1-699                -----------------------------LPLTDHQLNKL------------------------------------------------------------------
LOC_Os02g04810.1|11668.m00419/1-1094               VQNQMQQQKPSPTQNPQRINGQRLLLSHQQKDQNLQLQQQ----------------------------------------LLLQQKQQLQQQQQQQQQNQQQLNKS
LOC_Os04g59430.1|11670.m05892/1-530                -----------------------------NNISAPVCNGD------------------------------------------------------------------
LOC_Os07g08520.1|11673.m00789/1-729                -----------------------------FVIPKPSAAEH------------------------------------------------------------------
LOC_Os07g08530.1|11673.m00790/1-408                -----------------------------------------------------------------------------------------------------------
LOC_Os07g08600.1|11673.m00797/1-525                -----------------------------------------------------------------------------------------------------------
ARF1/1-665                                         -----------------------------FSPPAKAATFG------------------------------------------------------------------
ARF3/1-608                                         -----------------------------------------------------------------------------------------------------------
ARF4/1-788                                         -----------------------------EFVDATGVNPA------------------------------------------------------------------
ARF5/1-902                                         SGRV-------------------------QHGLEQSMEQA------------------------------------------------------------------
ARF6/1-933                                         QQQLSQQQQQQAYLGVPETHQPQSQAQSQSNNHLSQQQQQ------------------------------------------VVDNHNPSASSAAVVSAMSQFGSA
ARF7/1-1165                                        QQHSNNNQSQSQQQQQLLQQQQQQQLQQQHQQPLQQQTQQ---------------------------QQLRTQPLQSHSHPQPQQLQQHKLQQLQVPQNQLYNGQQ
ARF8/1-811                                         -----------------------------ENLPQQNMRQE--------------------------------------------------------VSNQPAGQQQ
ARF9/1-638                                         -----------------------------WSSPQQCHRDA------------------------------------------------------------------
ARF10/1-693                                        -----------------------------FGSPSPSLLSD------------------------------------------------------------------
ARF11/1-601                                        -----------------------------SPVASSFLSSF------------------------------------------------------------------
ARF12/1-593                                        -----------------------------ASPMNISLRYR------------------------------------------------------------------
ARF13/1-623                                        -----------------------------SPISVPEFSYP------------------------------------------------------------------
ARF14/1-605                                        -----------------------------ATPMNISLRYR------------------------------------------------------------------
ARF15/1-598                                        -----------------------------ASPMNISLLYR------------------------------------------------------------------
ARF16/1-670                                        -----------------------------NAHQYYGLSSS------------------------------------------------------------------
ARF17/1-585                                        -----------------------------APDPSPSMFSY------------------------------------------------------------------
ARF18/1-602                                        -----------------------------KLFQDPSLERI------------------------------------------------------------------
ARF19/1-1086                                       GTIAVANQVSCQSPNQPTGFSQSQLQQQSMLPTGAKMTHQ-------------------------------------------------------NINSMGNKGLS
ARF20/1-615                                        -----------------------------ASPMNTSLRYR------------------------------------------------------------------
ARF21/1-606                                        -----------------------------ASPMNISLRYR------------------------------------------------------------------
ARF22/1-600                                        -----------------------------ASPMNISLTYR------------------------------------------------------------------
ARF23/1-222                                        -----------------------------------------------------------------------------------------------------------
ARF2/1-859                                         FGTNI------------------------DPSHGQRIPFY------------------------------------------------------------------
PoptrARF8.1/1-827                                  QIST-------------------------ESLPRHLLQQQ--------------------------------------------------------------LNNQ
PoptrARF1.1/1-660                                  -----------------------------DPYPSPNFSTT------------------------------------------------------------------
PoptrARF7.3/1-1113                                 QNQQQHIMGQNQQQSLMQSQLSDQVNQH-MQMSDNQIQSQ--------------------------------------------LMQKLQQQQQSVSAQQSAMHQA
PoptrARF2.3/1-792                                  -----------------------------SKLNETGMFNC------------------------------------------------------------------
PoptrARF5.1/1-933                                  VDGK-------------------------FKAKPEHLPDQ-------------------------------------------------------LSQPTSTGECI
PoptrARF5.2/1-944                                  GERKLKAETEH------------------LPDQLSQLTST--------------------------------------------------------------GECI
PoptrARF7.4/1-1137                                 QQNQQQHIMGQNQQQSLMQSQLSDHVNQHMQISDNHIQLQ--------------------------------------------LLQKLQQQQQSLLAQQSAMQQA
PoptrARF16.1/1-669                                 -----------------------------LSSSDLHFNKL------------------------------------------------------------------
PoptrARF1.2/1-662                                  -----------------------------DLYSSPNFSTT------------------------------------------------------------------
PoptrARF9.1/1-666                                  -----------------------------LSVTGGWLSSP------------------------------------------------------------------
PoptrARF2.1/1-854                                  FGA--------------------------NADSSHGFGAP-----------------------------------------------------------------F
PoptrARF16.3/1-700                                 -----------------------------LPLSDLNLNKL------------------------------------------------------------------
PoptrARF3.1/1-709                                  -----------------------------IPTSENSFKGI------------------------------------------------------------------
PoptrARF6.2/1-914                                  ENKHQSQPQSQTPTRSHLIHQLQHQHSLDSPEQQQPLLQQ----------------------------------------------QHLADQQIPNVVSAISQYAS
PoptrARF6.4/1-953                                  HQHQHQQVQ--------------------QPKQLNELSAQ--------------------------------------------------QQIPNVISALPHLTSV
PoptrARF17.1/1-594                                 -----------------------------SDLTNSRTGHM------------------------------------------------------------------
PoptrARF2.4/1-879                                  -----------------------------TSDEKSMFNWH------------------------------------------------------------------
PoptrARF16.2/1-708                                 -----------------------------LSSSDLHFNKL------------------------------------------------------------------
PoptrARF2.2/1-852                                  FGT--------------------------NSDSFHGFGAP-----------------------------------------------------------------F
PoptrARF10.1/1-708                                 -----------------------------ISLSDFQFKKK------------------------------------------------------------------
PoptrARF3.3/1-109                                  -----------------------------------------------------------------------------------------------------------
PoptrARF9.2/1-670                                  -----------------------------TQTEGGWLASP------------------------------------------------------------------
PoptrARF6.1/1-884                                  IQQQLLHHHLLDSP---------------QQQQQPFLQQQ-----------------------------------------------QLADQQIPNGVSAISQYAS
PoptrARF4/1-713                                    -----------------------------LSPVYRCQEIC------------------------------------------------------------------
PoptrARF6.3/1-163                                  -----------------------------------------------------------------------------------------------------------
PoptrARF3.2/1-714                                  -----------------------------IATSNNSYKGI------------------------------------------------------------------
PoptrARF9.3/1-579                                  -----------------------------------------------------------------------------------------------------------
PoptrARF6.5/1-907                                  QPKQL------------------------NELSAPQQIPN-------------------------------------------------------VISALPHLTSV
PoptrARF9.4/1-632                                  -----------------------------QKEIDSNLNNN------------------------------------------------------------------
PoptrARF7.1/1-1047                                 QQQQQQQQLLASNIQSQSIPSPNRSSYQLTSLPQDSQFHQ-----------------------------------------------------QMEQQSNFSHRQQ
PoptrARF7.2/1-1093                                 PPTLNDSVQPVVYSQLQQQQQLLASNTQSQSIPSANKSSY------------------------------------PLTSLPQDSQLHQQMEQQSNLSQRQQQTQL
PoptrARF8.2/1-816                                  QISS-------------------------DSLPRHLLQQQ--------------------------------------------------------------LNNQ
PoptrARF16.6/1-91                                  -----------------------------------------------------------------------------------------------------------
PoptrARF16.4/1-701                                 -----------------------------LPLSDPHLNKL------------------------------------------------------------------
PoptrARF16.5/1-536                                 -----------------------------LPLSDPHLNKL------------------------------------------------------------------
PoptrARF10.2/1-713                                 -----------------------------ISLSDIQFNNK------------------------------------------------------------------
PoptrARF17.2/1-592                                 -----------------------------SDLTNSTMGHT------------------------------------------------------------------
PoptrARF2.6/1-724                                  -----------------------------YPCPSNTVSFP----------------------------------------------------------------GG
PoptrARF2.5/1-614                                  -----------------------------LGNPNSWCSTP------------------------------------------------------------------
```

Consensus

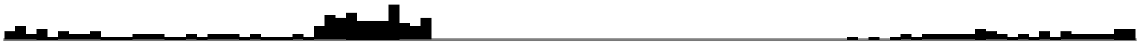

```
QQKQ+QQQQQQQQQ+QQQQQLSQQLQQQLSLSNPSLSQQ-----------------------------------PS-+LPQ-QQLQQLQQQQQQVQQ+QSQLQQQ
```

```
                                              1280       1290       1300       1310       1320       1330       1340       1350       1360       1370
LOC_Os01g70270.1|11667.m07038_altsplice/1-809  FDEAPNRLTSFKNQFQDQGSARHFSDPYYYVSPQPSLTVESSTQMHTDSKELHFWNGQSTVYGNSRDRPQNFRFEQNSSSWLNQSFARPEQPRVIR---------
LOC_Os01g13520.1|11667.m01333/1-700            ------------LSEISQKIFQVTSNEARIATWPARSAYQAEEPT--------------------------------------------SKLSSNTAACGYR---------
LOC_Os02g06910.1|11668.m00642/1-909            PQQQQQESHQQPQHQQMQQQKHLSNFQTVPNALSVFSQLSSTPQSTPSTLQTVSPFSQQHNFPDTNISCLSPSNVSSMHDTLRSFPSEAASDLPG---------
LOC_Os02g35140.1|11668.m03331/1-757            ----------LNVGFSTKNEPSALSNKHFYWPMRETR-----------------------------------------------ANSYSASISKVPS---------
LOC_Os02g41800.1|11668.m03990/1-784            ---------QSSLMYPGLRRPDHVGPTSIPPPRISTDLTM--------------------------------------------GSSPPARALSMGA---------
LOC_Os04g57610.1|11670.m05695/1-819            SAVLPSPTSLPSHLREKFGFSDPNANSSSFITSSSSDNMLDSSFLQGS------------------------------------SKAVDLSRFNQPV---------
LOC_Os04g43910.1|11670.m04249/1-696            -------QPNLMLYAGIRLPPADRAAPAPRPPRIIISTDLTIG-----------------------------------------SPGKPDDAACSPS---------
LOC_Os04g49230.1|11670.m04840/1-294            ---------------------------------------------------------------------------------------------------------
LOC_Os04g36060.1|11670.m03490/1-1674           GEGNRRRSTLVLPHVRSAPFHAVVPGAVGGQRRQIVAAAVAEADGNENGNKHFTCHRENANYGLKSASENKIELLRGAHSWADSNLIEDVLASVNN-------DV
LOC_Os05g43920.1|11682.m04181/1-700            ---------PIGFSESLGFPEVLQGQEMSQVVPLFRGATFGARTQN--------------------------------------DRVVSANSVHRSA---------
LOC_Os06g09660.1|11680.m00929/1-1056           QPTLTQTSQVQQAAEYQQALLQSQQQQQQLQLQQLSQPEVQLQLLQKIQQQNMLSQLNPQHQSQLIQQLSQKSQEILQQQILQHQFGGSDSIGQLK---------
LOC_Os06g46410.1|11680.m04579/1-918            QQQQQQSQCMQVPQHQQMQQQKNMTNYQSVPNALSPFSQLSSPSQSSPMTLQTVLPFSQPQSYPDTSMSSLSPSNTSTMHNALRPFSSEAPSHLSM---------
LOC_Os06g47150.1|11680.m04656/1-731            -----------QSSLSPHGLHQLDHGMQPRIAAGLIIG--------------------------------------------HPAARDDISCLLT---------
LOC_Os06g48950.1|11680.m04850/1-1116           QQLSKMPAQLSSLANQQFQLTDQQLQLQLLQKLQQQQQSLLSQPAVTLAQLPLIQEQQKLLLDMQQQLSNSQTLSQQQMMPQQSTKVPSQNTPLPL--------P
LOC_Os08g40900.1|11674.m04106_altsplice/1-1056 PQQLHHQQQQTQQLQPVQQVQQSVQEHQQIKIQPVHVSMDASMNTQVADHQMKLQLLKALQPQQPLISEQQKMLLDLQQQVINSQSAPQQCVQVTN--QAISLHNS
LOC_Os11g32110.1|11687.m02952/1-812            AGHSNFWHTVNAQYQDQQSNHNMFPSSWSFMPPNTRLGLNKQNYSMIQEAGVLSQRPGNTKFGNGVYAALPGRGTEQYSGGWFGHMMPNSHMDDTQ---------
LOC_Os12g41950.1|11686.m04180/1-900            QHVLQEQSSQEMQQQLPSSDHHVADVASESGSAPQAQSSLLSGSSFYNQNLLEGNSDPPLHLHNNFHNFSNQEASNLLILPRSSQLMASDGWPSKR---------
LOC_Os12g29520.1|11686.m02861_altsplice/1-842  GNRSNTWNTINVHYPDQNANHNMYPGTWSLMPPNTGFGVNQQNYLMTPDITLPQRSLNAKFGGNGAFTSLRAHGIDQRSSGWLGHIEPSSHIDDAS---------
LOC_Os04g56850.1|11670.m05610_altsplice/1-956  SSKSPDGGKSVNSFPNQGCFSQFIDGLDWMTQPSYYQDSNVIQPAGVSENIFSSSADIPPSMIADTMETFQASCLSDCLPNSIQEFISSPDLNSLT--------F
LOC_Os05g48870.1|11682.m04683_altsplice/1-696  ---------CSGFGESHRLQKVLQGQELFRPYRGTLVDASMGS-----------------------------------------NGFHQQDSPRAPG---------
LOC_Os01g48060.1|11667.m04657_altsplice/1-723  ---------CSGFGESQRFQEVLQGQEVFRPYRGGTLSDACIRG----------------------------------------SGFRQPDGNHAPG---------
LOC_Os01g54990.1|11667.m05421/1-656            ---------SVSFGESIGFPEVLQGQEISQTVPPFQGMLPDACSAK--------------------------------------SRYELKNYVCTPA---------
LOC_Os10g33940.1|11676.m02996/1-699            ---------HLGLLHSGSFNRLDAITPPSRISKGFVVS----------------------------------------------SAPAHDNISCLLS---------
LOC_Os02g04810.1|11668.m00419/1-1094           LGQLVNLASQQSKLFDEELQLQILQKLQQQSLMSQSTSTLSQPPLIQEQQKLITDMQKQLSNSHSLAQQQMMPQQEIKPSLQATPLLPTVQQEQQQ---------
LOC_Os04g59430.1|11670.m05892/1-530            ---------------SLLVPPILMHPQPQPPADIQGARHNNG------------------------------------------HAYADIPSSSTPS---------
LOC_Os07g08520.1|11673.m00789/1-729            ---------VLPRIPDLRVTNLQGGRWEFGHTWSDADTDRRSSSHTLAAGW---------------------------------SAFVKAKRLCVGD---------
LOC_Os07g08530.1|11673.m00790/1-408            ---------------------------------------------------------------------------------------------------------
LOC_Os07g08600.1|11673.m00797/1-525            ---------------------------------------------------------------------------------------------------------
ARF1/1-665                                     ------------HGGNKSFGVSIGSAFWPTNADSAAESFA--------------------------------------------SAFNNESTEKKQT---------
ARF3/1-608                                     ---------GTEFGDSYGFHKVLQGQETVPAYSITDHRQQ--------------------------------------------HGLSQRNIWCGPF---------
ARF4/1-788                                     ---------CSGVMDLDRFPRVLQGQEICSLKSFPQFAGFSPAAPNPFAYQANK------------------------------SSYYPLALHGIRS---------
ARF5/1-902                                     -----SQVTTSTVCNEEKVNQLLQKPGASSPVQADQCLDITHQIYQPQSDPINGFSFLETDELTSQVSSFQSLAGSYKQPFILSSQDSSAVVLPD---------
ARF6/1-933                                     SQPNTSPLQSMTSLCHQQSFSDTNGGNNPISPLHTLLSNFSQDESSQLLHLTRTNSAMTSSGWPSKRPAVDSSFQHSGAGNNNTQSVLEQLGQSHT---------
ARF7/1-1165                                    AAQQHQSQQASTHHLQPQLVSGSMASSVITPPSSSLNQSFQQQQQQSKQLQQAHHHLGASTSQSSVIETSKSSSNLMSAPPQETQFSRQVEQQPPGLNGQNQQTL
ARF8/1-811                                     QLQQPDQNAYLNAFKMQNGHLQQWQQQSEMPSPSFMKSDFTDSSNKFATTASPASGDGNLLNFSITGQSVLPEQLTTEGWSPKASNTFSEPLSLPQ---------
ARF9/1-638                                     -------------NEDAKKSDWLNNSYSVSNVAKDSTLN---------------------------------------------DQMVSPVEQKKPE---------
ARF10/1-693                                    -------LNLSSYTGNNKLHSPAMFLSSFNPRHHHYQAR---------------------------------------------DSENSNNISCSLT---------
ARF11/1-601                                    ---------SQSHESNPSVKLLFQDPATERNSN-------------------------------------------------KSVFSSGLQCKIT---------
ARF12/1-593                                    ----------DATEDAMNPSKLLMSYPVQPMPKLNYN-----------------------------------------------NQMVTEMEENITT---------
ARF13/1-623                                    ----------NAIEDSKFLSGLLLNHSLLAIPNENYNS----------------------------------------------DQMIQPRKEDITT---------
ARF14/1-605                                    ----------DITEDAMTPSRLLMSYPVQPMAKLNY------------------------------------------------NNVVTPIEENITT---------
ARF15/1-598                                    ----------ETTEDAMNPSRLLMSYPVQPMPKRNYN-----------------------------------------------NQMVTQIEENLTT---------
ARF16/1-670                                    ----------------DLHHYYLNRPPPPPPPSSLQLSPSLGLRN---------------------------------------IDTKNEKGFCFLT---------
ARF17/1-585                                    ----------------STFPAGMQGARQYDFGSFNP------------------------------------------------TGFIGGNPPQLFT---------
ARF18/1-602                                    --------------SGGYSSNNSFKPETPPPP-------------------------------------------------------------------------
ARF19/1-1086                                   QMTSFAQEMQFQQQLEMHNSSQLLRNQQEQSSLHSLQQNLSQNPQQLQMQQQSSKPSPSQQLQLQLLQKLQQQQQQQSIPPVSSSLQPQLSALQQTQSHQLQQLLS
ARF20/1-615                                    ----------DTTEDAMNPSRLLMSYPVQPMPKLNYN-----------------------------------------------NQMVTQIEENITT---------
ARF21/1-606                                    ----------DTTEAAMNPSRLLMSYPVQPMPKLNYN-----------------------------------------------NQMVTQIEENITT---------
ARF22/1-600                                    ----------DTTEDVMNPSRLLMSYPVQPMPKLNYN-----------------------------------------------NQMVTQIEENITT---------
ARF23/1-222                                    ---------------------------------------------------------------------------------------------------------
ARF2/1-859                                     DHSSSPSMPAKRILSDSEGKFDYLANQWQMIHSGLSLKLHESPKVPAATDASLQGRCNVKYSEYPALNGLSTENAGGNWPIRPRALNYYEEVVNAQ---------
PoptrARF8.1/1-827                              PGDQAHQHQHIYHDGLQIQTDQLQRQQSNLPSPSFSKTEYMDSSSKFSATNTPMQNML--------------------------GSLCSEGSVNLLD---------
PoptrARF1.1/1-660                              -----------AKANSLSFCGNSQVTSVSPNSMYRPNQVESVT-----------------------------------------DSFAPVVNKDLGE---------
PoptrARF7.3/1-1113                             GQLGQLQDSQRQLLDASQSFSRSMTPSQMLEIPQTTPTSLPQPNTIPQQMTKNNNQTNTRFSHLPQQLKPQQQHSGIMLLSEMAGHMGLPPSSMAN---------
PoptrARF2.3/1-792                              --------HSAPLMYLGHPFNMMASRMEVHVAKDKDIQQQRGSWF---------------------------------------SPLPYADNSSHPS---------
PoptrARF5.1/1-933                              VQKPISCPMTQQNATNHLVFQNQNQGQSQLQASLWPMQALTESSLLNSQQIRASLADATTPNCSLPFLDAGEWISHPMSIDSMCRSGPLSMFGLQD---------
PoptrARF5.2/1-944                              VQKPSSCPMTQQNATNHLAFQNQNQGHSQLQTNLWPAQAFPESSLLNSQQILSPLADATAPNCSLPFLDADEWISHPMSLAGMYRSGPLSMFGSQD---------
PoptrARF7.4/1-1137                             GQLGQLQDSQRQLLDASQSFSRSMAPSQMLEIPQTAPTSLPQPNTIPQQLTKNNNQNNVRFSHPPQQPKLQQQHTGILPLSEMAGHMGLLPSSMAN---------
PoptrARF16.1/1-669                             ---------------QAGLFPVDFQRLDRAAPPSRISNSNFVG-----------------------------------------NTQNSESISCLLT---------
PoptrARF1.2/1-662                              ---------AKVNSLGFRGNSQVASVSHNSMHWPNRVESVT-------------------------------------------DSFAPVVNKDSGE---------
PoptrARF9.1/1-666                              ----------HVNVSQHLFQEAMEDSKSVSPWPVFVGYSTPL------------------------------------------SSKSKNDPIPDPS---------
PoptrARF2.1/1-854                              VDQTAGGANPMKKHLSDQGQFNLLASPWSIMSPGLSLKLSESNSRVPIQGSSDVTYQSRENIRYSAFSEYPMLHGLRVEQSHGNCMMPPPPSHFDN---------
PoptrARF16.3/1-700                             ----------HTGLLRAGFPPLLDHTASLTKASNIQTIQ---------------------------------------------KPILSEGVSCELT---------
PoptrARF3.1/1-709                              ----------GFNESYRFHKVLQGQEIFPRSPYRRIPNANKAREN---------------------------------------CGLGLSDGVQRSS---------
PoptrARF6.2/1-914                              ATQSLTPPLQAISLCQQHSFSDSNGNLVTSPVVSPLQSLLGSFPQDETSHLFNFPRTNPLTTSSGWPSKRAAVDPLISSVAPQCMMSQVEQLGPPQ---------
PoptrARF6.4/1-953                              APSHSPSLQAIPSQCQQQAFSEPLGNLIAASGVSSVPSIMGSLPQDRGHLLNSNGSNPVSSSALLSKQAAFGPQLSSGAAPGVLPQVEQSGTTQSA---------
PoptrARF17.1/1-594                             ---------NASMLNYSTFPAGMQGARQDPFSTFSL------------------------------------------------SNLISENASQVFG---------
PoptrARF2.4/1-879                              ---------SAPMMYTGHPFNMLASSMEVQVAKDKDTQQQHGSRF---------------------------------------LPFPYADSSPHPS---------
PoptrARF16.2/1-708                             --------------QSGLFPVDFQRRDHAASPSRISSGNFMG------------------------------------------NTKKSENISCLLT---------
PoptrARF2.2/1-852                              VDQTAVAANPTKKHLSDQGQFNLLASPWSIMSSGLLLKLSESNTKVPVQGSDVTYQARANVFSEYPVLQGHRVEQSHKNWMMHPPPSHFDNHANSR---------
PoptrARF10.1/1-708                             ----------------LQSGLFLSSLQRFNPRTKNSENYPTG------------------------------------------HPDSNKNLSCLLT---------
PoptrARF3.3/1-109                              ---------------------------------------------------------------------------------------------------------
PoptrARF9.2/1-670                              ----------HVNVSQHPFQDAMEDSKSVSPWPVFSGYS---------------------------------------------TPLSSKSKNDAIL---------
PoptrARF6.1/1-884                              ASQSLTPPLQAISLCQQHSYSDSNGNPATSPAVSSLQSLLDSFPPNESSHLLSWPRINPLVTSSGWPSKRAAVESLTSSGAPQCMVTQVEQLGPLH---------
PoptrARF4/1-713                                -----------SLSSVGALGKPNTGRSSFQMYPGPR------------------------------------------------PAFYPVAAESLRS---------
PoptrARF6.3/1-163                              ---------------------------------------------------------------------------------------------------------
PoptrARF3.2/1-714                              -----------GFNESYRFHKVFQGQEIFPSSPYGRIPNANEAREN--------------------------------------CSLGFSDGVQRSS---------
PoptrARF9.3/1-579                              ------------------------------------------------------------------------------------STQSHEQSNCLIN---------
PoptrARF6.5/1-907                              APSQSPSLQPISSQCQQQAFSEPLGNSIAASDVSSMHSVIGSLSQDGGSHLLNSNGSNPVISPALLSKRAAIDPQLSSGAAH-CALPQVEQLRTTQ---------
PoptrARF9.4/1-632                              ------------GSCNSRARPEGIWPSSSHMNSIISGF----------------------------------------------ASSISRQSNGLIN---------
PoptrARF7.1/1-1047                             TQLQQSPLLLLQQNPSQRVQPQPHQQIQQLSQPDNSEQQLHLQLLQNLQQQQQQQLLSPESLLLQSQKLQQQQQTHQQNQQLHQSPLTQNQQPLGS---------
PoptrARF7.2/1-1093                             QQSPLLHSNSNSNNCNNYHSPATLSNSFTFSYYRSCSSISSCPPLQAQFCSLSSYSNNNSRPTSNTSNFSSHLCHRISSHLGSNSFSTAALMQTQS---------
PoptrARF8.2/1-816                              PDDQAQQHQHAYHDGLHIQTDLLQRQQSNLPSPSFSKTEYMDSSPKFTVSTTPMQNIL--------------------------GSLCTEGSGNLLD---------
PoptrARF16.6/1-91                              ---------------------------------------------------------------------------------------------------------
PoptrARF16.4/1-701                             ----------QTGLLRTGFPPLLDHTASLTKASNVQTIP---------------------------------------------KPSMCEDVSCELT---------
PoptrARF16.5/1-536                             ----------QTGLLRTGFPPLLDHTASLTKASNVQTIP---------------------------------------------KPSMCEDVSCELT---------
PoptrARF10.2/1-713                             ---------------QQSGMFLSSLQRFNPHSRNSETYLTG-------------------------------------------HTNSNENISCLLT---------
PoptrARF17.2/1-592                             ---------NASMLNYSTFPAGMQGARQDPFSTFGL------------------------------------------------SNFISENAPQVFS---------
PoptrARF2.6/1-724                              NIARLGIPNSRHSTFNSYGVHDNAVGSRSLSVPNVSHNSGSQKWRG--------------------------------------SELKHANEVPLAA---------
PoptrARF2.5/1-614                              --------------SSYGVHDNAVGSRSLSVPFVSHNSGSQKW-----------------------------------------RGFELKHANEVPL---------
```

Consensus

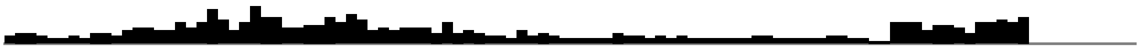

```
QQQ+Q+Q+QQQQSLLDSQGFSQLLQGQQSLPPSSSQSSLSSGSLQNSQQLLSS+S+NPLSSSS+TQ+SSQSQSLSSSQP++SSFVSED+SCLPT-----Q--S
```

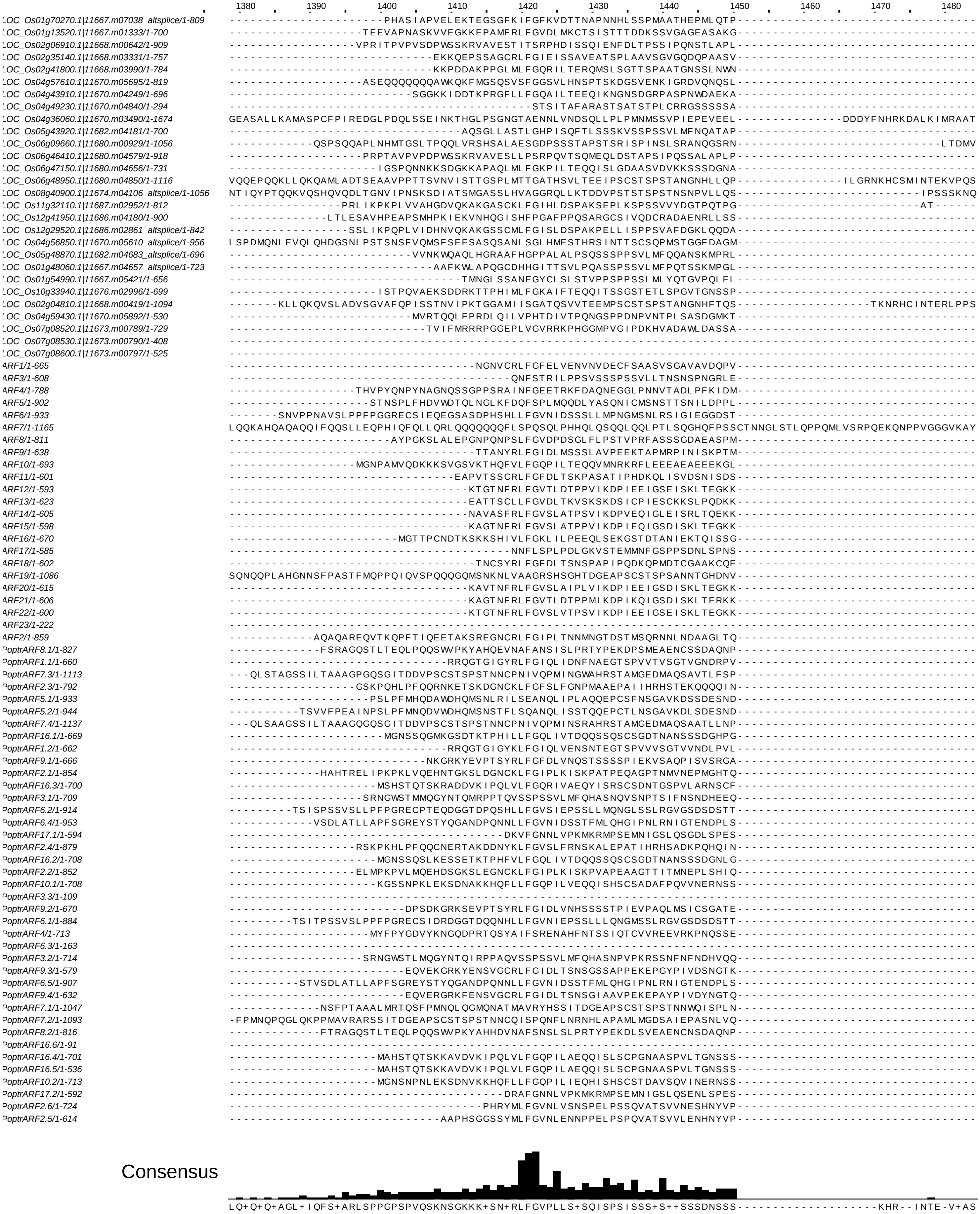

1380 1390 1400 1410 1420 1430 1440 1450 1460 1470 1480
LOC_Os01g70270.1|11667.m07038_altsplice/1-809
LOC_Os01g13520.1|11667.m01333/1-700
LOC_Os02g06910.1|11668.m00642/1-909
LOC_Os02g35140.1|11668.m03331/1-757
LOC_Os02g41800.1|11668.m03990/1-784
LOC_Os04g57610.1|11670.m05695/1-819
LOC_Os04g43910.1|11670.m04249/1-696
LOC_Os04g49230.1|11670.m04840/1-294
LOC_Os04g36060.1|11670.m03490/1-1674
LOC_Os05g43920.1|11682.m04181/1-700
LOC_Os06g09660.1|11680.m00929/1-1056
LOC_Os06g46410.1|11680.m04579/1-918
LOC_Os06g47150.1|11680.m04656/1-731
LOC_Os06g48950.1|11680.m04850/1-1116
LOC_Os08g40900.1|11674.m04106_altsplice/1-1056
LOC_Os11g32110.1|11687.m02952/1-812
LOC_Os12g41950.1|11686.m04180/1-900
LOC_Os12g29520.1|11686.m02861_altsplice/1-842
LOC_Os04g56850.1|11670.m05610_altsplice/1-956
LOC_Os05g48870.1|11682.m04683_altsplice/1-696
LOC_Os01g48060.1|11667.m04657_altsplice/1-723
LOC_Os01g54990.1|11667.m05421/1-656
LOC_Os10g33940.1|11676.m02996/1-699
LOC_Os02g04810.1|11668.m00419/1-1094
LOC_Os04g59430.1|11670.m05892/1-530
LOC_Os07g08520.1|11673.m00789/1-729
LOC_Os07g08530.1|11673.m00790/1-408
LOC_Os07g08600.1|11673.m00797/1-525
ARF1/1-665
ARF3/1-608
ARF4/1-788
ARF5/1-902
ARF6/1-933
ARF7/1-1165
ARF8/1-811
ARF9/1-638
ARF10/1-693
ARF11/1-601
ARF12/1-593
ARF13/1-623
ARF14/1-605
ARF15/1-598
ARF16/1-670
ARF17/1-585
ARF18/1-602
ARF19/1-1086
ARF20/1-615
ARF21/1-606
ARF22/1-600
ARF23/1-222
ARF2/1-859
PoptrARF8.1/1-827
PoptrARF1.1/1-660
PoptrARF7.3/1-1113
PoptrARF2.3/1-792
PoptrARF5.1/1-933
PoptrARF5.2/1-944
PoptrARF7.4/1-1137
PoptrARF16.1/1-669
PoptrARF1.2/1-662
PoptrARF9.1/1-666
PoptrARF2.1/1-854
PoptrARF16.3/1-700
PoptrARF3.1/1-709
PoptrARF6.2/1-914
PoptrARF6.4/1-953
PoptrARF17.1/1-594
PoptrARF2.4/1-879
PoptrARF16.2/1-708
PoptrARF2.2/1-852
PoptrARF10.1/1-708
PoptrARF3.3/1-109
PoptrARF9.2/1-670
PoptrARF6.1/1-884
PoptrARF4/1-713
PoptrARF6.3/1-163
PoptrARF3.2/1-714
PoptrARF9.3/1-579
PoptrARF6.5/1-907
PoptrARF9.4/1-632
PoptrARF7.1/1-1047
PoptrARF7.2/1-1093
PoptrARF8.2/1-816
PoptrARF16.6/1-91
PoptrARF16.4/1-701
PoptrARF16.5/1-536
PoptrARF10.2/1-713
PoptrARF17.2/1-592
PoptrARF2.6/1-724
PoptrARF2.5/1-614
Consensus
LQ+Q+Q+AGL+IQFS+ARLSPPGPSPVQSKNSGKKK+SN+RLFGVPLLS+SQISPSISSS+S++SSSDNSSS------------------KHR--INTE-V+AS

```
                                                   1490      1500      1510      1520      1530      1540      1550      1560      1570      1580
LOC_Os01g70270.1|11667.m07038_altsplice/1-809     -------------------------------------------------------------------------SSLNQLQPVQTDCIPEVSVSTAGTATENEK-S
LOC_Os01g13520.1|11667.m01333/1-700               --------------------------------------------------------------------------------------TGSHEDSGQLSAFSKVTKE
LOC_Os02g06910.1|11668.m00642/1-909               -----------------------------PGRECLVDQDGSSDPQNHFLFGVNIDSQSLLMQDGIPSLHNENSSSTIPYSTSNFLSPSQDDYPLSQTLTTPGCLD
LOC_Os02g35140.1|11668.m03331/1-757               -------------------------------------------------------------------------------------DAESDQLSQPSHANKSDA-P
LOC_Os02g41800.1|11668.m03990/1-784               ----------------------------------------------------------------------------------TEKGASEGSGSGVIQNSPTDN-T
LOC_Os04g57610.1|11670.m05695/1-819               ----------------------------------------------------------FSPQVDSSSLLYNMVPNLTSNVSDGNLSTIPSGSTYLQNAMYGCL-D
LOC_Os04g43910.1|11670.m04249/1-696               ----------------------------------------------------------------------------------PNTSEGSDSGVTQGSPTKNTTPS
LOC_Os04g49230.1|11670.m04840/1-294               --------------------------------------------------------------------------------------------------------P
LOC_Os04g36060.1|11670.m03490/1-1674              KHSQAASNAFLRGDHAAAKELSLRAQEERSAAEELNKKAAKEIFRLRNSNSIWKLDMHGLHASEAVEVLERHLHRIEFQPPGNNAASSDEVARSEPRVSGPSIEP
LOC_Os05g43920.1|11682.m04181/1-700               ----------------------------------------------------------------------------------NHETVSGTNNKGMHVSQFASQEM
LOC_Os06g09660.1|11680.m00929/1-1056              ATPQIDNLLQEIQSKPDNRIKNDIQSKETVPIHNRHPVSDQLDASSATSFCLDESPREGFSFPPVCLDNNVQVDPRDNFLIAENVDALMPDALLSRGMASGKGMCT
LOC_Os06g46410.1|11680.m04579/1-918               ------------------------------GRGCLVDQDGNSDPQNHLLFGVNIDSQSLLMQGGIPSLQGENDSTAIPYSTSNFLSPSQNDFPLDQTLSSADCLD
LOC_Os06g47150.1|11680.m04656/1-731               -------------------------------------------------------------------------------ENTVNKSNSDVSSPRSNQNGTTDN-L
LOC_Os06g48950.1|11680.m04850/1-1116              AAPMSVPSSLEAVTATPRMMKDSPKLNHNVKQSVVASKLANAGTGSQNYVNNPPPTDYLETASSATSVWLSQNDGLLHQNFPMSNFNQPQMFKDAPPDAEIHAA-N
LOC_Os08g40900.1|11674.m04106_altsplice/1-1056    SLTTAGKTSQSSVVLGPTIEQDTKPYQNVKQTVMIPKTTEQRPATGQDCINNNPQMDYLDTSSSATSVCLSQADGSLQQNFPPSSFHQHHLLKDTVPDSEFEVT-D
LOC_Os11g32110.1|11687.m02952/1-812               -----------------------------------------------------------------------EWRRPDVTEVEKCSDPSKAMKPLDTPQPDSVP-E
LOC_Os12g41950.1|11686.m04180/1-900               ----------------------------------------------------------------SFELQDGMTSIITDANRETDTMAIPLLRYSGADLTTENTLA
LOC_Os12g29520.1|11686.m02861_altsplice/1-842     -----------------------------------------------------------------------------LEEDECSDPSKTVKPLDGAQHDSARE-K
LOC_Os04g56850.1|11670.m05610_altsplice/1-956     ---------------------------------------------------------------------------YSKLPRLKESQILSLPEIHTNSMGTSACSM
LOC_Os05g48870.1|11682.m04683_altsplice/1-696     ----------------------------------------------------------------------------------------EFGHGQLDKHENDRRVR
LOC_Os01g48060.1|11667.m04657_altsplice/1-723     -----------------------------------------------------------------------------EYIYGCLDRNENSRHFKIGPTQDMTR-T
LOC_Os01g54990.1|11667.m05421/1-656               --------------------------------------------------------------------------------------------------------A
LOC_Os10g33940.1|11676.m02996/1-699               ------------------------------------------------------------------------------------------NGNAHKTGNASDGSG
LOC_Os02g04810.1|11668.m00419/1-1094              TAPMLIPTSIDAVTATPLMTKELPKPNNNVKQSVVNSKLPNVAPGPQNCINHALQTDNLETSSSATSLCPSRTDGLVHQGFPSSNFNQHQMFKDALPDVEMEGVDP
LOC_Os04g59430.1|11670.m05892/1-530               --------------------------------------------------------------------------------------------------------I
LOC_Os07g08520.1|11673.m00789/1-729               ---------------------------------------------------------------------------------------QPFRVTYCPWQGTAEFVV
LOC_Os07g08530.1|11673.m00790/1-408               ---------------------------------------------------------------------------------------------------------
LOC_Os07g08600.1|11673.m00797/1-525               ---------------------------------------------------------------------------------------------------------
ARF1/1-665                                        ----------------------------------------------------------------------------PSNEFDSGQQSEPLNINQSDIPSGSGD-P
ARF3/1-608                                        ---------------------------------------------------------------------------------------------------------
ARF4/1-788                                        --------------------------------------------------------------------------------------MGKQKGSELNMNASSGCKL
ARF5/1-902                                        ---------------------------------------SNTVLDDFCAIKDTDFQNHPSGCLVGNNNTSFAQDVQSQITSASFADSQAFSRQDFPDNSGGTG-T
ARF6/1-933                                        ------------------------------------------------------------------------------TLPFTSSNFNNDFSGNLAMTTPSSC-I
ARF7/1-1165                                       SGITDGGDAPSSSTSPSTNNCQISSSGFLNRSQSGPAILIPDAAIDMSGNLVQDLYSKSDMRLKQELVGGQQKSKASLTDHQLEASASGTSYGLDGGENNRQQNFLA
ARF8/1-811                                        ----------------------------------------------------------------------------------------SLTDSGFQNSLYSCM-Q
ARF9/1-638                                        ---------------------------------------------------------------------------------------DSHSDPKSEISKVSEEKK
ARF10/1-693                                       ---------------------------------------------------------------------------------------------------------
ARF11/1-601                                       --------------------------------------------------------------------------------------------------TTKCQDP
ARF12/1-593                                       ------------------------------------------------------------------------------------------------------F-G
ARF13/1-623                                       ------------------------------------------------------------------------------------------------------F-D
ARF14/1-605                                       ------------------------------------------------------------------------------------------------------F-G
ARF15/1-598                                       ------------------------------------------------------------------------------------------------------F-G
ARF16/1-670                                       -------------------------------------------------------------------------------------------GSNQNGVAGREFSS
ARF17/1-585                                       ---------------------------------------------------------------------------------------------------------
ARF18/1-602                                       --------------------------------------------------------------------------------------------------------P
ARF19/1-1086                                      -SPTNFLSRNQQQGQAASVSASDSVFERASNPVQELYTKTESRISQGMMNMKSAGEHFRFKSAVTDQIDVSTAGTTYCPDVVGPVQQQQTFPLPSFGFDGDCQSHH
ARF20/1-615                                       ------------------------------------------------------------------------------------------------------F-G
ARF21/1-606                                       ------------------------------------------------------------------------------------------------------F-G
ARF22/1-600                                       ------------------------------------------------------------------------------------------------------F-G
ARF23/1-222                                       ---------------------------------------------------------------------------------------------------------
ARF2/1-859                                        ----------------------------------------------------------------------------IASPKVQDLSDQSKGSKSTNDHREQGR-P
PoptrARF8.1/1-827                                 -------------------------------------------------------TLFGANIDSSGLLLPTTVPRYSTSSIDADVSSMPLGDSGFQNSLYGCV-Q
PoptrARF1.1/1-660                                 -------------------------------------------------------------------------------------------VSLEAESDQHSE-P
PoptrARF7.3/1-1113                                -----SALETVSSNGNLVKDLLQKSEVKPSLNISKNQNPGLFSSQTYLNGVAAQIDYLDTSSSTTSVCLSQNDVHLQQNNNSLSYNPQSVLLRDASHDGELQG-D
PoptrARF2.3/1-792                                 ------------------------------------------------------------------------VASDHLKLLGSEGFLEQAKHSKHVRPEEQGN-I
PoptrARF5.1/1-933                                 ------------------QSGIYGSLNIDASNGGGSVYDRSVSSAILDEFCTLKDADLQNASDCLVGNLSSSQDVQSQITSASLADSQAFSRQDFPDNSGGTS-S
PoptrARF5.2/1-944                                 ------------------QSGIYGSLNFDASNGGGSVVDPSVSSAILDEFCTLKDADLQNASDCLVGNLSSSQDVQSQITSASLADSQAFSRQDFPDNSGGTS-S
PoptrARF7.4/1-1137                                -----SALETVSSNGNLVKDLLQKSEVKPSLNISKNQSPGFFTPQTYLNGVAAQTDYLDTSSSTTSICLSQNDVHLQQNNNSLSYNPQPMLLRDTIHDGELQA-D
PoptrARF16.1/1-669                                ------------------------------------------------------------------------------------KAISDGSGSASQQNGPLEN-S
PoptrARF1.2/1-662                                 --------------------------------------------------------------------------------------------SLEAESDQHSE-P
PoptrARF9.1/1-666                                 -------------------------------------------------------------------------------TDGRVLAALPAVDSDQKHEVSKEK-K
PoptrARF2.1/1-854                                 -------------------------------------------------------------------------PASHQLTSESDQKSEHSRGSKLADENENEK-P
PoptrARF16.3/1-700                                -----------------------------------------------------------------------------EGKLDKMAKFSEGSVSTLPHRGLSKH-S
PoptrARF3.1/1-709                                 -----------------------------------------------------------------------------------TTNTQSWFYPETHGGKFKLSSH
PoptrARF6.2/1-914                                 -----------------------------------------------------------------------------VPFSSNYMSIAGTNFSLNPAMAPSSC-I
PoptrARF6.4/1-953                                 ----------------------------------------------------------------------------MPFTASTFTSATGSDIPLTSDMTASSC-V
PoptrARF17.1/1-594                                ---------------------------------------------------------------------------------------------------------
PoptrARF2.4/1-879                                 ------------------------------------------------------------------------VASDHLQLLGSDRYLEQLKHPKHARCEEQEN-I
PoptrARF16.2/1-708                                ------------------------------------------------------------------------------------KASSDGSGSALQQNGPMEN-S
PoptrARF2.2/1-852                                 -------------------------------------------------------------------------PVSHQLTFESDQKSEQSKGSKMTDENENEK-P
PoptrARF10.1/1-708                                ---------------------------------------------------------------------------------DSNREKNSDVLRSAPGKQISQEKS
PoptrARF3.3/1-109                                 ---------------------------------------------------------------------------------------------------------
PoptrARF9.2/1-670                                 ---------------------------------------------------------------------------------GHVLGALSAVDSDQKSEVSKEQ-K
PoptrARF6.1/1-884                                 -----------------------------------------------------------------------------IPFSSNYISTAGTNFSLNPAMTPSSC-I
PoptrARF4/1-713                                   -----------------------------------------------------------------------YKTQESISAAPALCANLRNQKDDFFNGNATGCKL
PoptrARF6.3/1-163                                 ---------------------------------------------------------------------------------------------------------
PoptrARF3.2/1-714                                 ----------------------------------------------------------------------------------TATTRSWFCGPEMQGGNFKLSAH
PoptrARF9.3/1-579                                 --------------------------------------------------------------------------------------------------GLVPA-A
PoptrARF6.5/1-907                                 ----------------------------------------------------------------------------MPFAASTFTSATGSDIPLNSDMTASSC-V
PoptrARF9.4/1-632                                 ----------------------------------------------------------------------------GLVPASSEAEKAQTMDVSMSSKEQKQV-V
PoptrARF7.1/1-1047                                -----------FLNRNQQAPAMLMGDSAIEPASNLVQELQSKSEIHIKNEFPSLKGLDQLKYKGTVTDQLEASSSGTSYCLDAGTIQQNFSAPTFGLDGDVQS-H
PoptrARF7.2/1-1093                                -------------------------------------DLQNKSEIRVKNEFPSSRGLDQLKYKGAVTDQLEASSSGTSYCLDAGNIQQNFSVPTFGLDSDVQS-H
PoptrARF8.2/1-816                                 --------------------------------------------------------------TFFGLLLPTTVPRYPTSTVDTDVSSMPLGDSGFQNSLYGCV-Q
PoptrARF16.6/1-91                                 ---------------------------------------------------------------------------------------------------------
PoptrARF16.4/1-701                                -----------------------------------------------------------------------------EGNLDKMANFSDGSVSTLHRRGLPEC-S
PoptrARF16.5/1-536                                -----------------------------------------------------------------------------EGNLDKMANFSDGSVSTLHRRGLPEC-S
PoptrARF10.2/1-713                                ------------------------------------------------------------------------------DESSSKEKISDVLLSAPGKKISQVK-S
PoptrARF17.2/1-592                                ---------------------------------------------------------------------------------------------------------
PoptrARF2.6/1-724                                 ---------------------------------------------------------------------------------------------VTSQSSVSEPSK
PoptrARF2.5/1-614                                 --------------------------------------------------------------------------------------------LTSQSSVSEPS-K
```

Consensus

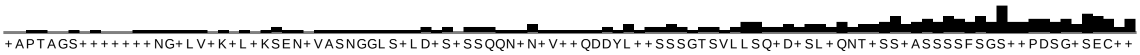

```
+APTAGS+++++++NG+LV+K+L+KSEN+VASNGGLS+LD+S+SSQQN+N+V++QDDYL++SSSGTSVLLSQ+D+SL+QNT+SS+ASSSFSGS++PDSG+SEC++
```

```
                                                   1600       1610       1620       1630       1640       1650       1660       1670       1680       1690
LOC_Os01g70270.1|11667.m07038_altsplice/1-809      GQQAQQSSKDVQ-----------------------------------------------------------------------------------SKTQVASTRSC
LOC_Os01g13520.1|11667.m01333/1-700                HIAADESPQEIQ------------------------------------------------------------------------------------SHQNYTARTR
LOC_Os02g06910.1|11668.m00642/1-909                ESGYVPCSDNAD-------------------------------------------------------------------------------------QVKRPHATF
LOC_Os02g35140.1|11668.m03331/1-757                AASSEPSPHETQ---------------------------------------------------------------------------------------SRQVRSC
LOC_Os02g41800.1|11668.m03990/1-784                SSERLQWFRENS-----------------------------------------------------------------------------------TVSELGLEPGQ
LOC_Os04g57610.1|11670.m05695/1-819                DSSGLLQNTGEN---------------------------------------------------------------------------------------DPATRTF
LOC_Os04g43910.1|11670.m04249/1-696                WSLPYFGGNNIS-----------------------------------------------------------------------------------RASEYELNPGQ
LOC_Os04g49230.1|11670.m04840/1-294                QGRGFISTRPCH----------------------------------------------------------------------------------------------
LOC_Os04g36060.1|11670.m03490/1-1674               GPGKVVFVRPIQAILEVITGIGKHSKGQASLPVAVRGFLIENGYRFDELRPGHKLLTSQPEVKNHKGIKGHSPSNLGFRRSGLQLKVTAIFGWIKGDTRTRELNPS
LOC_Os05g43920.1|11682.m04181/1-700                LSETVTWPGTQR-------------------------------------------------------------------------------------------QTP
LOC_Os06g09660.1|11680.m00929/1-1056               LTSGQRDHRDVE-------------------------------------------NELSSAAFSSQSFGVPDMSFKPGCSSDVAVTDAGMPSQGLWNNQTQRMRTF
LOC_Os06g46410.1|11680.m04579/1-918                ESGYVPCSQNSD------------------------------------------------------------------------------------QVINRPPATF
LOC_Os06g47150.1|11680.m04656/1-731                SCGGVPLCQDNK------------------------------------------------------------------------------------VLDVGLETGH
LOC_Os06g48950.1|11680.m04850/1-1116               TSNNALFGINGD-GPLGFPIGLGTDDFLSNGIDAAKYENHISTEIDNSYRIPKDAQQEISSSMVSQSFGASDMAFNSIDSTINDGGFLNRSSWP-PAAPLKRMRTF
LOC_Os08g40900.1|11674.m04106_altsplice/1-1056     PRNNLLFGVNID----GQLGLPLNADLLANDIGTDKYMDQLPGNGISNFISSKDSQQELSSSMISHSFGVADMAFNSIDSAINDTPFLNRNSRSAAGPAHQRMRTY
LOC_Os11g32110.1|11687.m02952/1-812                KPSSQQASRNMS-----------------------------------------------------------------------------------CKSQGVSTRSC
LOC_Os12g41950.1|11686.m04180/1-900                TSNCLGESGTFN-------------------------------------------------------------------------------PLNNISVNPSQGATF
LOC_Os12g29520.1|11686.m02861_altsplice/1-842      HQSCPDGTKNIQ-----------------------------------------------------------------------------------SKQQNGSSRSC
LOC_Os04g56850.1|11670.m05610_altsplice/1-956      DATEYSLDRSAK-------------------------------------------------------------------------------------PMKPPVRTY
LOC_Os05g48870.1|11682.m04683_altsplice/1-696      FGPSEGIERREQ------------------------------------------------------------------------------------RIPLQPYPTS
LOC_Os01g48060.1|11667.m04657_altsplice/1-723      DQTLRLWPHLIS---------------------------------------------------------------------------------------GKVLDEC
LOC_Os01g54990.1|11667.m05421/1-656                SKNNDKSGNDSQ-------------------------------------------------------------------------------------PALRQHKLL
LOC_Os10g33940.1|11676.m02996/1-699                SSICIGFSSQGH-----------------------------------------------------------------------------------EASDLGLEAGH
LOC_Os02g04810.1|11668.m00419/1-1094               SNSGLFGINNDN----LLGFPIETEDLLINALDSVKYQNHISTDVENNYPMQKDALQEISTSMVSQSFGQSDMAFNSIDSAINDGAFLNKNSWP-AAPLLQRMRTF
LOC_Os04g59430.1|11670.m05892/1-530                QLFGVTITSPVQ----------------------------------------------------------------------------------------------
LOC_Os07g08520.1|11673.m00789/1-729                RREEVEGSPPLA------------------------------------------------------------------------------------------PGTR
LOC_Os07g08530.1|11673.m00790/1-408                ----------------------------------------------------------------------------------------------------------
LOC_Os07g08600.1|11673.m00797/1-525                ----------------------------------------------------------------------------------------------------------
ARF1/1-665                                         EKSSLRSPQESQ---------------------------------------------------------------------------------------SRQIRSC
ARF3/1-608                                         ----------------------------------------------------------------------------------------------------------
ARF4/1-788                                         FGFSLPVETPAS------------------------------------------------------------------------------------KPQSSSKRIC
ARF5/1-902                                         SSSNVDFDDCSL--------------------------------------------------------------------------RQNSKGSSWQKIATPRVRTY
ARF6/1-933                                         DESGFLQSSENL------------------------------------------------------------------------------------GSENPQSNTF
ARF7/1-1165                                        PTFGLDGDSRNS----LLGGANVDNGFVPDTLLSRGYDSQKDLQNMLSNYGGVTNDIGTEMSTSAVRTQSFGVPNVPAISNDLAVNDAGVLGGGLWPAQTQRMRTY
ARF8/1-811                                         DTTHELLHGAGQ------------------------------------------------------------------------------------INSSNQTKNF
ARF9/1-638                                         QEPAEGSPKEVQ------------------------------------------------------------------------------------SKQSSSTRSR
ARF10/1-693                                        VARGLTWNYSLQ----------------------------------------------------------------------------------------GLETGH
ARF11/1-601                                        NSSNSPKEQKQQ----------------------------------------------------------------------------------------TSTRSR
ARF12/1-593                                        LSQTLRSPTEIQ------------------------------------------------------------------------------------NKQFSSSRTC
ARF13/1-623                                        QTQPLRSPKEVQ------------------------------------------------------------------------------------STEFNFTRSR
ARF14/1-605                                        QSQILRSPTEIQ------------------------------------------------------------------------------------SKQFSSTRTC
ARF15/1-598                                        QSQTLRSPTKIQ------------------------------------------------------------------------------------SKQFSSTRTC
ARF16/1-670                                        SDEGSPCSKKVH-------------------------------------------------------------------------------------DASGLETGH
ARF17/1-585                                        NTTNLSSGNDLV------------------------------------------------------------------------------------GNRGPLSKKV
ARF18/1-602                                        ITPTSMSEQKKQ----------------------------------------------------------------------------------------QTSRSR
ARF19/1-1086                                       PRNNLAFPGNLE-------------AVTSDPLYSQKDFQNLVPNYGNTPRDIETELSSAAISSQSFGIPSIPFKPGCSNEVGGINDSGIMNGGGLWPNQTQRMRTY
ARF20/1-615                                        QSQTLRSPIEIQ------------------------------------------------------------------------------------SKQFGSTRTC
ARF21/1-606                                        QSQTLRSPIEIQ------------------------------------------------------------------------------------SKQFSSSRTC
ARF22/1-600                                        QSQTLRSPTEIQ------------------------------------------------------------------------------------SKQFSSTRTC
ARF23/1-222                                        ----------------------------------------------------------------------------------------------------------
ARF2/1-859                                         FQTNNPHPKDAQ-------------------------------------------------------------------------------------TKTNSSRSC
PoptrARF8.1/1-827                                  DSSELLSNAGQM-------------------------------------------------------------------------------------DPPTPSGTF
PoptrARF1.1/1-660                                  EKSCLRSHQELQ---------------------------------------------------------------------------------------SRQIRSC
PoptrARF7.3/1-1113                                 PRNNILYGTNIDSQLVMPINSDHLLTKGMMGLGKDFSNNFSSGGMLTNCENSKDPQQELSSAIVSKSFGVPDMPFNSIDSTINDSSLLNRGSWAPPQQQFQRMRTY
PoptrARF2.3/1-792                                  FQASALHSKDVQ-----------------------------------------------------------------------------------GMPEGDSTRRC
PoptrARF5.1/1-933                                  SNIEFDNSNLLQ--------------------------------------------------------------------------------NNSWQQVAPRVRTY
PoptrARF5.2/1-944                                  SNVDFDNSNMLQ--------------------------------------------------------------------------------NSSWQQVAPRVRTY
PoptrARF7.4/1-1137                                 LRNNIPCGTNIDSQLTMPVSSDNLFTKGMVGLGKDFSNNFSSAGMLTSCENSKDPQQDLSSSMVSQSFGVPEMPFNSINSAINDNSCLNRGAWAPPQQQFQRMRTY
PoptrARF16.1/1-669                                 SGGRCPWYKDYQ------------------------------------------------------------------------------------KTDPGLETGH
PoptrARF1.2/1-662                                  EKSCLRSSQELQ---------------------------------------------------------------------------------------SRQIRSC
PoptrARF9.1/1-666                                  PEQLHGSSKEIQ-----------------------------------------------------------------------------------SKQSSTSTRSR
PoptrARF2.1/1-854                                  LQVGHMRMRDSH-----------------------------------------------------------------------------------GKAQNSSTRSC
PoptrARF16.3/1-700                                 SCEGLQWNKNNH-----------------------------------------------------------------------------------RKSEQSLEIGH
PoptrARF3.1/1-709                                  SDPGLRGDSQCS----------------------------------------------------------------------------------------TNPYVL
PoptrARF6.2/1-914                                  DESGFLQSMENV------------------------------------------------------------------------------------GQGNPPSRTF
PoptrARF6.4/1-953                                  DESGFLQSSENV------------------------------------------------------------------------------------DQVNPSTRTF
PoptrARF17.1/1-594                                 ----------------------------------------------------------------------------------------------------------
PoptrARF2.4/1-879                                  FQASSLYSKDVQ-----------------------------------------------------------------------------------GKPEGGSARRC
PoptrARF16.2/1-708                                 SDERSTWYKDHQ------------------------------------------------------------------------------------KTDLGLETDH
PoptrARF2.2/1-852                                  FQAGHLRTKDNH-----------------------------------------------------------------------------------GKAQNGSTRSC
PoptrARF10.1/1-708                                 CTTGFSWHQSLQ-----------------------------------------------------------------------------------NTSENGMDTGH
PoptrARF3.3/1-109                                  ----------------------------------------------------------------------------------------------------------
PoptrARF9.2/1-670                                  PEQLQKSPKEIQ-----------------------------------------------------------------------------------SKQSSTSTRSR
PoptrARF6.1/1-884                                  DDSCFLQSTENA------------------------------------------------------------------------------------SQGNPPTRTF
PoptrARF4/1-713                                    FGFSLNAETSPN-------------------------------------------------------------------------------------SQNTSKRSC
PoptrARF6.3/1-163                                  -------------------------------------------------------------------------------------------------------RTF
PoptrARF3.2/1-714                                  SEPSVKRDGQWS----------------------------------------------------------------------------------------NSPFGL
PoptrARF9.3/1-579                                  SEAERAQAMDVS----------------------------------------------------------------------------------MYSKEQKQVLSE
PoptrARF6.5/1-907                                  DESGFLQSSENV------------------------------------------------------------------------------------DQVNPSTRTF
PoptrARF9.4/1-632                                  PDTLAKESQSKQ-------------------------------------------------------------------------------------GSTTSTRTR
PoptrARF7.1/1-1047                                 PASNIDALAPDT----------------LLSREYDSQKDLQNLLVNYGGTAQDINMELSTAAISAQSFGVSNIPFKPDGSNDIAINDTGILNNGAWTNQNQRMRTY
PoptrARF7.2/1-1093                                 PRNSLPFASNID-------ALAPDTLLSRGYDSQKDLQNLLANYGGTTRDIETELSTAAISSQSFAVPNIPFKPGCSNDVAINDTGVLNNGLWTNQTNQTQRMRTY
PoptrARF8.2/1-816                                  DSSELLPNAGQM-------------------------------------------------------------------------------------DPPTPSRTF
PoptrARF16.6/1-91                                  ----------------------------------------------------------------------------------------------------------
PoptrARF16.4/1-701                                 SCEELQWNKDKH-----------------------------------------------------------------------------------QKSEPSLETGH
PoptrARF16.5/1-536                                 SCEELQWNKDKH-----------------------------------------------------------------------------------QKSEPSLETGH
PoptrARF10.2/1-713                                 CGTGFSWHQSLH-----------------------------------------------------------------------------------NTSEIGKDTGP
PoptrARF17.2/1-592                                 ----------------------------------------------------------------------------------------------------------
PoptrARF2.6/1-724                                  STSGVNSEKQCK------------------------------------------------------------------------------------NCCSAAIRSC
PoptrARF2.5/1-614                                  STSGVNSDKQCR------------------------------------------------------------------------------------NCSSAAIPSC
```

Consensus

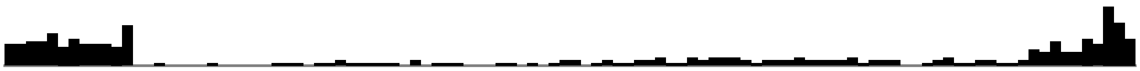

```
SSSGLLSSKEIQSQL-++GG+D-+-DLL+NGLGSDKY+N+LSG++++++R+SKD+QQELSSSM+SQSFGVPDMPFNSIDSAIND+++LNR+SWGLWKQQNGSTRTC
```

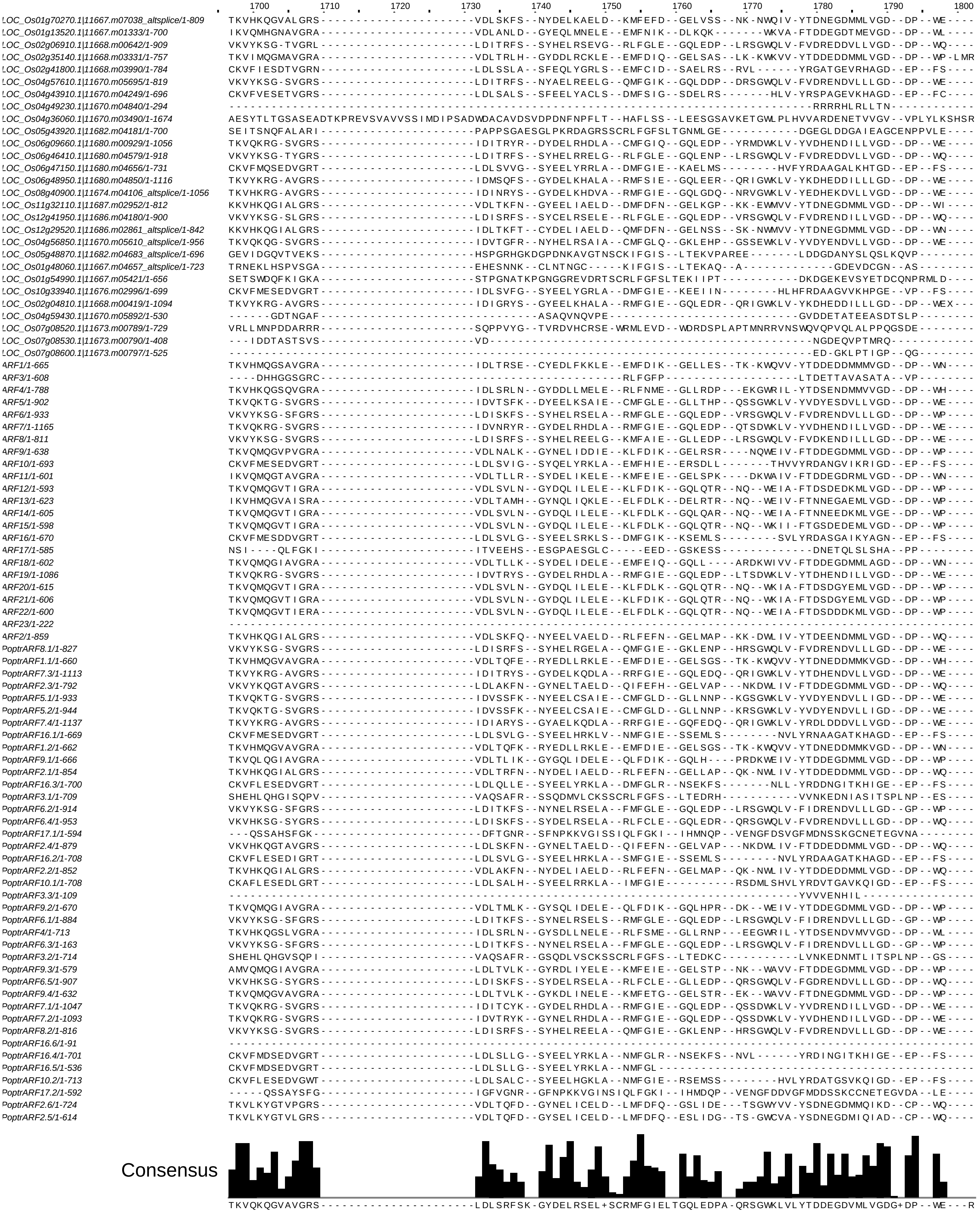

1700 1710 1720 1730 1740 1750 1760 1770 1780 1790 1800
Consensus
TKVQKQGVAVGRS--------------------LDLSRFSK-GYDELRSEL+SCRMFGIELTGQLEDPA-QRSGWKLVLYTDEDGDVMLVGDG+DP--WE---R

```
                                                       1810       1820       1830       1840       1850       1860       1870       1880       1890       1900
LOC_Os01g70270.1|11667.m07038_altsplice/1-809          -EFC-SIVR-------------------KIYIYTKE---EVQKMNSK-----------------------------------------------------------
LOC_Os01g13520.1|11667.m01333/1-700                    -EFC-QMVR-------------------KIVLYPIE---DEKKIEPH-----------------------------------------------------------
LOC_Os02g06910.1|11668.m00642/1-909                    -EFV-NSVS-------------------CIKILSPQ---EVQQMGKP-----------------------------------------------------------
LOC_Os02g35140.1|11668.m03331/1-757                    WTLT-TLLQ-------------------CLILLSRV-LVNAALLILYIQRAVLFNGLCFQIHWINAGGFGGWKEGYSTKRASGVCREP------------------
LOC_Os02g41800.1|11668.m03990/1-784                    RDFN-KKAQSAQVSYCSPVSFFIIINNDQLRVLVDS---RVLRCKNPVTTHQRLECRGPLAQGTSACSMQTQTQRCAISDLSGGLTSR------------------
LOC_Os04g57610.1|11670.m05695/1-819                    -SFV-NSVW-------------------YIKILSPE---DVHKMGKQ-----------------------------------------------------------
LOC_Os04g43910.1|11670.m04249/1-696                    -AFV-KSAR-------------------KLRILTDA---GSDNLGDX-----------------------------------------------------------
LOC_Os04g49230.1|11670.m04840/1-294                    -STL------------------------RCTTRAPX----------------------------------------------------------------------
LOC_Os04g36060.1|11670.m03490/1-1674                   GEFVFDQSWAEAYYSYGLEYYP------KLQSCVPFTPVTGQRILVRDTPYRDQVFEALVKELKSLTTKLKVSSLHITFPSEGEFSKLKDSEFLQRIGMQYHWRNR
LOC_Os05g43920.1|11682.m04181/1-700                    -LFGHSHST-------------------PGALHALC---AAAPLGMX-----------------------------------------------------------
LOC_Os06g09660.1|11680.m00929/1-1056                   -EFV-GCVK-------------------SIKILSAA---EVQQMSLD-----------------------------------------------------------
LOC_Os06g46410.1|11680.m04579/1-918                    -EFV-NSVS-------------------CIKILSPQ---EVQQMGKP---------------------------------------FE------------------
LOC_Os06g47150.1|11680.m04656/1-731                    -EFT-KTAR-------------------RLNILTDT---SGDNLASW-----------------------------------------------------------
LOC_Os06g48950.1|11680.m04850/1-1116                   -EFV-GCVK-------------------CIRILSPQ---EVQQMSLE-----------------------------------------------------------
LOC_Os08g40900.1|11674.m04106_altsplice/1-1056         -DFV-KCVR-------------------CIRILSPQ---EEMQMRLV-----------------------------------------------------------
LOC_Os11g32110.1|11687.m02952/1-812                    --FA-PVQP-------------------DGTGYLSC------YLATX-----------------------------------------------------------
LOC_Os12g41950.1|11686.m04180/1-900                    -EFA-NSVW-------------------CIKILSPQ---EVQQLVRG-------------------------------------GDGL------------------
LOC_Os12g29520.1|11686.m02861_altsplice/1-842          -EFC-NMVH-------------------KIFIYTRE---EVQKMNPG---------------------------------------AL------------------
LOC_Os04g56850.1|11670.m05610_altsplice/1-956          -EFI-NCVR-------------------CIRILSPS---EVQQMSEN-----------------------------------------------------------
LOC_Os05g48870.1|11682.m04683_altsplice/1-696          -KSLGNSCA-------------------TVSVLX------------------------------------------------------------------------
LOC_Os01g48060.1|11667.m04657_altsplice/1-723          -YHS------------------------RLQSLKPQ---MPKSLGSS-----------------------------------------------------------
LOC_Os01g54990.1|11667.m05421/1-656                    -LFGYNCST-------------------PGALHALC----AAPLGIX-----------------------------------------------------------
LOC_Os10g33940.1|11676.m02996/1-699                    -DFM-KAAR-------------------RLTIIAGD----RERIERP-----------------------------------------------------------
LOC_Os02g04810.1|11668.m00419/1-1094                   ----------------------------------------------------------------------------------------------------------
LOC_Os04g59430.1|11670.m05892/1-530                    -D--------------------------SLTNGHNQ---DGARLX-------------------------------------------------------------
LOC_Os07g08520.1|11673.m00789/1-729                    -EAAAATTSTAHAG--------------DATTSAPSLALQLQTMASS-----------------------------------------------------------
LOC_Os07g08530.1|11673.m00790/1-408                    ----------------------------RLEALIPD---NIX----------------------------------------------------------------
LOC_Os07g08600.1|11673.m00797/1-525                    -EVI------------------------SIENYATS-------IGAX-----------------------------------------------------------
ARF1/1-665                                             -EFC-GMVR-------------------KIFIYTPE---EVKKLSPK-----------------------------------------------------------
ARF3/1-608                                             ----------------------------CVEGNSMKGASAVQSNHHH-----------------------------------------------------------
ARF4/1-788                                             -DFC-NVVW-------------------KIHLYTKE---EVENANDD-----------------------------------------------------------
ARF5/1-902                                             -EFV-GCVR-------------------CIRILSPT---EVQQMSEE-----------------------------------------------------------
ARF6/1-933                                             -EFV-SSVW-------------------CIKILSPQ---EVQQMGKR--------------------------------GLELLNSAP------------------
ARF7/1-1165                                            -EFV-NCVQ-------------------SIKILSSA---EVQQMSLD-----------------------------------------------------------
ARF8/1-811                                             -SFV-NNVW-------------------YIKILSPE---DVHQMGDH-----------------------------------------------------------
ARF9/1-638                                             -EFC-NMVK-------------------RIFIWSKE---EVKKMTPG-----------------------------------------------------------
ARF10/1-693                                            -DFM-KATK-------------------RLTIKMDI---GGDNVRKT-----------------------------------------------------------
ARF11/1-601                                            -EFC-KMAK-------------------KLFIYPSD---EVKKMRSK-----------------------------------------------------------
ARF12/1-593                                            -EFC-NMVK-------------------KIFIQKRR----------------------------------------------------------------------
ARF13/1-623                                            -EFC-NMAK-------------------RIFICSKE---EIKKMKLK-----------------------------------------------------------
ARF14/1-605                                            -EFC-NMVK-------------------KIFIYSKE---EVKNLKSR-----------------------------------------------------------
ARF15/1-598                                            -EFC-NMVK-------------------RIYIQKRR----------------------------------------------------------------------
ARF16/1-670                                            -EFL-KTAR-------------------RLTILTEQ---GSESVVV------------------------------------------------------------
ARF17/1-585                                            ----------------------------SVPKHSNS---NAGSSSQG-----------------------------------------------------------
ARF18/1-602                                            -EFC-KMAK-------------------KIFIYSSD---EVKKMTTK-----------------------------------------------------------
ARF19/1-1086                                           -EFV-NCVQ-------------------NIKILSSV---EVQQMSLD-----------------------------------------------------------
ARF20/1-615                                            -EFC-KMVK-------------------KILIYSKE---EVKNLKSS-----------------------------------------------------------
ARF21/1-606                                            -EFC-KMVK-------------------KILIYSKE---EVKNLKSS-----------------------------------------------------------
ARF22/1-600                                            -EFC-NMVK-------------------KILIFKRG----GQKLEVQ-----------------------------------------------------------
ARF23/1-222                                            ----------------------------------------------------------------------------------------------------------
ARF2/1-859                                             -EFC-CMVR-------------------KIFIYTKE---EVRKMNPG-----------------------------------------------------------
PoptrARF8.1/1-827                                      -LFV-NNVW-------------------YIKILSPE---DVLKLGEQ-----------------------------------------------------------
PoptrARF1.1/1-660                                      -EFC-SMVK-------------------KIFIYASE---EVKRLSPK-----------------------------------------------------------
PoptrARF7.3/1-1113                                     -EFV-NCVR-------------------CIKILSPQ---EVQQMSLD-----------------------------------------------------------
PoptrARF2.3/1-792                                      -EFC-SMVR-------------------RIFVFTRE---EINRMEPR--------------------------------------SLN------------------
PoptrARF5.1/1-933                                      -EFV-GCVR-------------------CIRILSPS---EVQQMSEE-----------------------------------------------------------
PoptrARF5.2/1-944                                      -EFV-GCVR-------------------CIRILSPS---EVQQMSEE-----------------------------------------------------------
PoptrARF7.4/1-1137                                     -EFV-NCVR-------------------CIKILSPQ---EVQQMSLD-----------------------------------------------------------
PoptrARF16.1/1-669                                     -EFL-KTAR-------------------RLTILSDA---SSDNVGR------------------------------------------------------------
PoptrARF1.2/1-662                                      -EFC-GMVK-------------------KIFIYTSE---EVKRLSPK-----------------------------------------------------------
PoptrARF9.1/1-666                                      -EFC-NMVR-------------------RIFICSSQ---DVKKMSPG-----------------------------------------------------------
PoptrARF2.1/1-854                                      -EFV-GMVR-------------------KIVIYTRE---EVQRIKPG-----------------------------------------------------------
PoptrARF16.3/1-700                                     -NFS-KTAR-------------------RLTIVTRF---------RQ-----------------------------------------------------------
PoptrARF3.1/1-709                                      -SFLPRVGE-------------------QLHPKPPA---INNAVGSS-----------------------------------------------------------
PoptrARF6.2/1-914                                      -EFV-NSVW-------------------CIKILSPQ---EVQQMGKR-------------------------------------GLEL------------------
PoptrARF6.4/1-953                                      -EFV-NNVW-------------------YIKILSPL---EVQQMGKE----------------------GLTSAASVPSQKLSNSTSD------------------
PoptrARF17.1/1-594                                     -LEL------------------------SLTSSYTE---LLNRIDVQ-----------------------------------------------------------
PoptrARF2.4/1-879                                      -EFC-SMVR-------------------RIFIYTKE---EINRMEPR-----------------SLNLEAEGNSRSTDQMVDLENRIS------------------
PoptrARF16.2/1-708                                     -EFL-KTAR-------------------RLTILSYA---SRDNFGR------------------------------------------------------------
PoptrARF2.2/1-852                                      -EFV-GMVR-------------------KIVIYTKE---EAQKIKPG-----------------------------------------------------------
PoptrARF10.1/1-708                                     -VFM-KTAK-------------------RLTILMNR--ASGNSVGRR-----------------------------------------------------------
PoptrARF3.3/1-109                                      ----------------------------------------------------------------------------------------------------------
PoptrARF9.2/1-670                                      -EFC-NMVR-------------------RIYICSSQ---DVKRMGPG-----------------------------------------------------------
PoptrARF6.1/1-884                                      -EFV-NSVW-------------------YIKILSPQ---EVQQMGKR-------------------------------------GLEL------------------
PoptrARF4/1-713                                        -EFC-NVAT-------------------KIHIYTQE---EVEKMTLF-----------------------------------------------------------
PoptrARF6.3/1-163                                      -EFV-NSVW-------------------CIKILSPQ---EVQQMGKR-------------------------------------GLEL------------------
PoptrARF3.2/1-714                                      -SFLPRAGE-------------------HFHQSLQQ----------------------------------------------------------------------
PoptrARF9.3/1-579                                      -EFC-KMVK-------------------KIFIYSSE---EVKKTGTR-----------------------------------------------------------
PoptrARF6.5/1-907                                      -EFV-NNVW-------------------YIKILSPL---EVQQMGKE-------------------------------------GLSP------------------
PoptrARF9.4/1-632                                      -EFC-KMVK-------------------KIFIYSSE---EVKKMSTR-----------------------------------------------------------
PoptrARF7.1/1-1047                                     -EFM-SCVQ-------------------SIKILSSA---EVQQMSLD-----------------------------------------------------------
PoptrARF7.2/1-1093                                     -EFV-SCVQ-------------------SIKILSSA---EVQQMSLD-----------------------------------------------------------
PoptrARF8.2/1-816                                      -LFV-NNVW-------------------YIKILSPE---DVLKMGEQ-----------------------------------------------------------
PoptrARF16.6/1-91                                      ----------------------------------------------------------------------------------------------------------
PoptrARF16.4/1-701                                     -DFF-KTAR-------------------RLTIVTDS---SSGNVGI------------------------------------------------------------
PoptrARF16.5/1-536                                     ----------------------------------------------------------------------------------------------------------
PoptrARF10.2/1-713                                     -VFM-KTAK-------------------RLTILMNQPSTEIDDLSCR-----------------------------------------------------------
PoptrARF17.2/1-592                                     -LSLTSSYT-------------------ELLNRIDA---QCQRASPV-----------------------------------------------------------
PoptrARF2.6/1-724                                      -EFQ-LTVR-------------------RIFISPKE---DIGKLNPL-----------------------------------------------------------
PoptrARF2.5/1-614                                      -EFL-SAVH-------------------RIFICPKE---ETGKLNP------------------------------------------------------------
```

Consensus

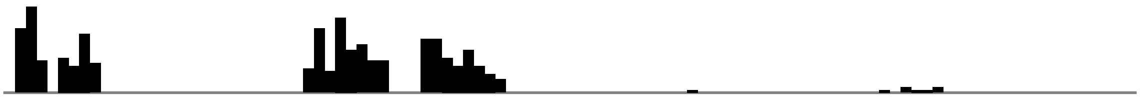

```
-EFVPNMVR-A-----------------+IKILSPE-AVEVQQMGPR-T---------------S+---G--SSRS-TS-L+GEGLEL------------------
```

```
                                                    1910      1920      1930      1940      1950      1960      1970      1980      1990      2000      2010
LOC_Os01g70270.1|11667.m07038_altsplice/1-809       ----------------------------------------------------------------------------------------------------------
LOC_Os01g13520.1|11667.m01333/1-700                 ----------------------------------------------------------------------------------------------------------
LOC_Os02g06910.1|11668.m00642/1-909                 ----------------------------------------------------------------------------------------------------------
LOC_Os02g35140.1|11668.m03331/1-757                 ----------------------------------------------------------------------------------------------------------
LOC_Os02g41800.1|11668.m03990/1-784                 ----------------------------------------------------------------------------------------------------------
LOC_Os04g57610.1|11670.m05695/1-819                 ----------------------------------------------------------------------------------------------------------
LOC_Os04g43910.1|11670.m04249/1-696                 ----------------------------------------------------------------------------------------------------------
LOC_Os04g49230.1|11670.m04840/1-294                 ----------------------------------------------------------------------------------------------------------
LOC_Os04g36060.1|11670.m03490/1-1674                DYRSFDEFLMDLKQPKRKNIRQERKKIPAQNLKMKRLRGDEIKDYFLCAFSWGRPYLTREFFHRLGEKMGDKVMLIVAEHDDKHVAGALNLIGGDTLFGRLWGCLP
LOC_Os05g43920.1|11682.m04181/1-700                 ----------------------------------------------------------------------------------------------------------
LOC_Os06g09660.1|11680.m00929/1-1056                ----------------------------------------------------------------------------------------------------------
LOC_Os06g46410.1|11680.m04579/1-918                 ----------------------------------------------------------------------------------------------------------
LOC_Os06g47150.1|11680.m04656/1-731                 ----------------------------------------------------------------------------------------------------------
LOC_Os06g48950.1|11680.m04850/1-1116                ----------------------------------------------------------------------------------------------------------
LOC_Os08g40900.1|11674.m04106_altsplice/1-1056      ----------------------------------------------------------------------------------------------------------
LOC_Os11g32110.1|11687.m02952/1-812                 ----------------------------------------------------------------------------------------------------------
LOC_Os12g41950.1|11686.m04180/1-900                 ----------------------------------------------------------------------------------------------------------
LOC_Os12g29520.1|11686.m02861_altsplice/1-842       ----------------------------------------------------------------------------------------------------------
LOC_Os04g56850.1|11670.m05610_altsplice/1-956       ----------------------------------------------------------------------------------------------------------
LOC_Os05g48870.1|11682.m04683_altsplice/1-696       ----------------------------------------------------------------------------------------------------------
LOC_Os01g48060.1|11667.m04657_altsplice/1-723       ----------------------------------------------------------------------------------------------------------
LOC_Os01g54990.1|11667.m05421/1-656                 ----------------------------------------------------------------------------------------------------------
LOC_Os10g33940.1|11676.m02996/1-699                 ----------------------------------------------------------------------------------------------------------
LOC_Os02g04810.1|11668.m00419/1-1094                ----------------------------------------------------------------------------------------------------------
LOC_Os04g59430.1|11670.m05892/1-530                 ----------------------------------------------------------------------------------------------------------
LOC_Os07g08520.1|11673.m00789/1-729                 ----------------------------------------------------------------------------------------------------------
LOC_Os07g08530.1|11673.m00790/1-408                 ----------------------------------------------------------------------------------------------------------
LOC_Os07g08600.1|11673.m00797/1-525                 ----------------------------------------------------------------------------------------------------------
ARF1/1-665                                          ----------------------------------------------------------------------------------------------------------
ARF3/1-608                                          ----------------------------------------------------------------------------------------------------------
ARF4/1-788                                          ----------------------------------------------------------------------------------------------------------
ARF5/1-902                                          ----------------------------------------------------------------------------------------------------------
ARF6/1-933                                          ----------------------------------------------------------------------------------------------------------
ARF7/1-1165                                         ----------------------------------------------------------------------------------------------------------
ARF8/1-811                                          ----------------------------------------------------------------------------------------------------------
ARF9/1-638                                          ----------------------------------------------------------------------------------------------------------
ARF10/1-693                                         ----------------------------------------------------------------------------------------------------------
ARF11/1-601                                         ----------------------------------------------------------------------------------------------------------
ARF12/1-593                                         ----------------------------------------------------------------------------------------------------------
ARF13/1-623                                         ----------------------------------------------------------------------------------------------------------
ARF14/1-605                                         ----------------------------------------------------------------------------------------------------------
ARF15/1-598                                         ----------------------------------------------------------------------------------------------------------
ARF16/1-670                                         ----------------------------------------------------------------------------------------------------------
ARF17/1-585                                         ----------------------------------------------------------------------------------------------------------
ARF18/1-602                                         ----------------------------------------------------------------------------------------------------------
ARF19/1-1086                                        ----------------------------------------------------------------------------------------------------------
ARF20/1-615                                         ----------------------------------------------------------------------------------------------------------
ARF21/1-606                                         ----------------------------------------------------------------------------------------------------------
ARF22/1-600                                         ----------------------------------------------------------------------------------------------------------
ARF23/1-222                                         ----------------------------------------------------------------------------------------------------------
ARF2/1-859                                          ----------------------------------------------------------------------------------------------------------
PoptrARF8.1/1-827                                   ----------------------------------------------------------------------------------------------------------
PoptrARF1.1/1-660                                   ----------------------------------------------------------------------------------------------------------
PoptrARF7.3/1-1113                                  ----------------------------------------------------------------------------------------------------------
PoptrARF2.3/1-792                                   ----------------------------------------------------------------------------------------------------------
PoptrARF5.1/1-933                                   ----------------------------------------------------------------------------------------------------------
PoptrARF5.2/1-944                                   ----------------------------------------------------------------------------------------------------------
PoptrARF7.4/1-1137                                  ----------------------------------------------------------------------------------------------------------
PoptrARF16.1/1-669                                  ----------------------------------------------------------------------------------------------------------
PoptrARF1.2/1-662                                   ----------------------------------------------------------------------------------------------------------
PoptrARF9.1/1-666                                   ----------------------------------------------------------------------------------------------------------
PoptrARF2.1/1-854                                   ----------------------------------------------------------------------------------------------------------
PoptrARF16.3/1-700                                  ----------------------------------------------------------------------------------------------------------
PoptrARF3.1/1-709                                   ----------------------------------------------------------------------------------------------------------
PoptrARF6.2/1-914                                   ----------------------------------------------------------------------------------------------------------
PoptrARF6.4/1-953                                   ----------------------------------------------------------------------------------------------------------
PoptrARF17.1/1-594                                  ----------------------------------------------------------------------------------------------------------
PoptrARF2.4/1-879                                   ----------------------------------------------------------------------------------------------------------
PoptrARF16.2/1-708                                  ----------------------------------------------------------------------------------------------------------
PoptrARF2.2/1-852                                   ----------------------------------------------------------------------------------------------------------
PoptrARF10.1/1-708                                  ----------------------------------------------------------------------------------------------------------
PoptrARF3.3/1-109                                   ----------------------------------------------------------------------------------------------------------
PoptrARF9.2/1-670                                   ----------------------------------------------------------------------------------------------------------
PoptrARF6.1/1-884                                   ----------------------------------------------------------------------------------------------------------
PoptrARF4/1-713                                     ----------------------------------------------------------------------------------------------------------
PoptrARF6.3/1-163                                   ----------------------------------------------------------------------------------------------------------
PoptrARF3.2/1-714                                   ----------------------------------------------------------------------------------------------------------
PoptrARF9.3/1-579                                   ----------------------------------------------------------------------------------------------------------
PoptrARF6.5/1-907                                   ----------------------------------------------------------------------------------------------------------
PoptrARF9.4/1-632                                   ----------------------------------------------------------------------------------------------------------
PoptrARF7.1/1-1047                                  ----------------------------------------------------------------------------------------------------------
PoptrARF7.2/1-1093                                  ----------------------------------------------------------------------------------------------------------
PoptrARF8.2/1-816                                   ----------------------------------------------------------------------------------------------------------
PoptrARF16.6/1-91                                   ----------------------------------------------------------------------------------------------------------
PoptrARF16.4/1-701                                  ----------------------------------------------------------------------------------------------------------
PoptrARF16.5/1-536                                  ----------------------------------------------------------------------------------------------------------
PoptrARF10.2/1-713                                  ----------------------------------------------------------------------------------------------------------
PoptrARF17.2/1-592                                  ----------------------------------------------------------------------------------------------------------
PoptrARF2.6/1-724                                   ----------------------------------------------------------------------------------------------------------
PoptrARF2.5/1-614                                   ----------------------------------------------------------------------------------------------------------
```

Consensus

```
----------------------------------------------------------------------------------------------------------
```

	2020 · 2030 · 2040 · 2050 · 2060 · 2070 · 2080 · 2090 · 2100 · 2110
LOC_Os01g70270.1|11667.m07038_altsplice/1-809	--
LOC_Os01g13520.1|11667.m01333/1-700	--
LOC_Os02g06910.1|11668.m00642/1-909	--
LOC_Os02g35140.1|11668.m03331/1-757	--
LOC_Os02g41800.1|11668.m03990/1-784	--
LOC_Os04g57610.1|11670.m05695/1-819	--
LOC_Os04g43910.1|11670.m04249/1-696	--
LOC_Os04g49230.1|11670.m04840/1-294	--
LOC_Os04g36060.1|11670.m03490/1-1674	DAYFPNLHFEACYYQNIHTTQAIEAAIELNLSKVEAGAQGEHKIQRGYLPVTTYSCHYFLDPGFGAAIGNYLVHETAQVRSCHRRKERARKKLSECHIDISAKIPT
LOC_Os05g43920.1|11682.m04181/1-700	--
LOC_Os06g09660.1|11680.m00929/1-1056	--
LOC_Os06g46410.1|11680.m04579/1-918	--
LOC_Os06g47150.1|11680.m04656/1-731	--
LOC_Os06g48950.1|11680.m04850/1-1116	--
LOC_Os08g40900.1|11674.m04106_altsplice/1-1056	--
LOC_Os11g32110.1|11687.m02952/1-812	--
LOC_Os12g41950.1|11686.m04180/1-900	--
LOC_Os12g29520.1|11686.m02861_altsplice/1-842	--
LOC_Os04g56850.1|11670.m05610_altsplice/1-956	--
LOC_Os05g48870.1|11682.m04683_altsplice/1-696	--
LOC_Os01g48060.1|11667.m04657_altsplice/1-723	--
LOC_Os01g54990.1|11667.m05421/1-656	--
LOC_Os10g33940.1|11676.m02996/1-699	--
LOC_Os02g04810.1|11668.m00419/1-1094	--
LOC_Os04g59430.1|11670.m05892/1-530	--
LOC_Os07g08520.1|11673.m00789/1-729	--
LOC_Os07g08530.1|11673.m00790/1-408	--
LOC_Os07g08600.1|11673.m00797/1-525	--
ARF1/1-665	--
ARF3/1-608	--
ARF4/1-788	--
ARF5/1-902	--
ARF6/1-933	--
ARF7/1-1165	--
ARF8/1-811	--
ARF9/1-638	--
ARF10/1-693	--
ARF11/1-601	--
ARF12/1-593	--
ARF13/1-623	--
ARF14/1-605	--
ARF15/1-598	--
ARF16/1-670	--
ARF17/1-585	--
ARF18/1-602	--
ARF19/1-1086	--
ARF20/1-615	--
ARF21/1-606	--
ARF22/1-600	--
ARF23/1-222	--
ARF2/1-859	--
PoptrARF8.1/1-827	--
PoptrARF1.1/1-660	--
PoptrARF7.3/1-1113	--
PoptrARF2.3/1-792	--
PoptrARF5.1/1-933	--
PoptrARF5.2/1-944	--
PoptrARF7.4/1-1137	--
PoptrARF16.1/1-669	--
PoptrARF1.2/1-662	--
PoptrARF9.1/1-666	--
PoptrARF2.1/1-854	--
PoptrARF16.3/1-700	--
PoptrARF3.1/1-709	--
PoptrARF6.2/1-914	--
PoptrARF6.4/1-953	--
PoptrARF17.1/1-594	--
PoptrARF2.4/1-879	--
PoptrARF16.2/1-708	--
PoptrARF2.2/1-852	--
PoptrARF10.1/1-708	--
PoptrARF3.3/1-109	--
PoptrARF9.2/1-670	--
PoptrARF6.1/1-884	--
PoptrARF4/1-713	--
PoptrARF6.3/1-163	--
PoptrARF3.2/1-714	--
PoptrARF9.3/1-579	--
PoptrARF6.5/1-907	--
PoptrARF9.4/1-632	--
PoptrARF7.1/1-1047	--
PoptrARF7.2/1-1093	--
PoptrARF8.2/1-816	--
PoptrARF16.6/1-91	--
PoptrARF16.4/1-701	--
PoptrARF16.5/1-536	--
PoptrARF10.2/1-713	--
PoptrARF17.2/1-592	--
PoptrARF2.6/1-724	--
PoptrARF2.5/1-614	--

Consensus

```
                                                      2130      2140      2150      2160      2170      2180      2190      2200      2210
LOC_Os01g70270.1|11667.m07038_altsplice/1-809      -------------------------------------------------SNAPRKDDSSENEKGHLPMPNKSDNX--------------------------
LOC_Os01g13520.1|11667.m01333/1-700                ----------------------------------------------------PKLLSSANPEQDQKTGFX-------------------------------
LOC_Os02g06910.1|11668.m00642/1-909                -------------------------------------GIELFSTSARRLGNSCDNYMSRQESRSLSTGIASVGSVEFX-----------------------
LOC_Os02g35140.1|11668.m03331/1-757                ---------------------------------------VVDAIDVEGPQGIDMLAIALRKHSLNVAQGSNLEALPFITELIIEILVVQTQRKTYIEVLSX
LOC_Os02g41800.1|11668.m03990/1-784                ---------------------------------------LQPPGEAIPLGSLALAASSLTHDDEICDLHRAAYPWLVESX---------------------
LOC_Os04g57610.1|11670.m05695/1-819                ----------------------------------------------------------GNDPRYLSX----------------------------------
LOC_Os04g43910.1|11670.m04249/1-696                -----------------------------------------------------------------------------------------------------
LOC_Os04g49230.1|11670.m04840/1-294                -----------------------------------------------------------------------------------------------------
LOC_Os04g36060.1|11670.m03490/1-1674               KKDIRYCLIMLFVQGYKDWFFLPPLINQRPRRTKLQCRKLSQTRKSFASCSLPLHLDCTGCFIRQAHHRLRKQPFDHLCSSSPRHRMAYTSRMNLKRKGKX
LOC_Os05g43920.1|11682.m04181/1-700                -----------------------------------------------------------------------------------------------------
LOC_Os06g09660.1|11680.m00929/1-1056               ----------------------------------------GDLGGVPPQTQACSASDDANAWRGX------------------------------------
LOC_Os06g46410.1|11680.m04579/1-918                ---------------------------------------LLSSAPGKRLGSSCDDYVSRQESRSLSTGIASVGSVEFX-----------------------
LOC_Os06g47150.1|11680.m04656/1-731                ------------------------------------------------WSTVAILKAKIAGFLVLVQFQGGNSVFFNX-----------------------
LOC_Os06g48950.1|11680.m04850/1-1116               ---------------------------------------GCDLGNNIPPNQACSSSDGGNAWRARCDQNSEAILKISMMKSKVEDVRYWNTAX--------
LOC_Os08g40900.1|11674.m04106_altsplice/1-1056     ----------------------------------------GDFGDSFLPNQACSSSDGGHPWRITGDX---------------------------------
LOC_Os11g32110.1|11687.m02952/1-812                -----------------------------------------------------------------------------------------------------
LOC_Os12g41950.1|11686.m04180/1-900                ---------------------------------------LSSPGARMQQSNACDDYSASHNMQNIAGNIASVAPLDYX-----------------------
LOC_Os12g29520.1|11686.m02861_altsplice/1-842      ---------------------------------------NSRSEDSRSTSVERGLVGEGLQGGLSTPSLNSENCX--------------------------
LOC_Os04g56850.1|11670.m05610_altsplice/1-956      --------------------------------------------------GMHVLNDCIQAAX--------------------------------------
LOC_Os05g48870.1|11682.m04683_altsplice/1-696      -----------------------------------------------------------------------------------------------------
LOC_Os01g48060.1|11667.m04657_altsplice/1-723      ----------------------------------------------------CATVHEQRPVVGRVVDISAVNTMIX------------------------
LOC_Os01g54990.1|11667.m05421/1-656                -----------------------------------------------------------------------------------------------------
LOC_Os10g33940.1|11676.m02996/1-699                -------------------------------------------------LIECLVEQAX------------------------------------------
LOC_Os02g04810.1|11668.m00419/1-1094               -----------------------------------------------------------------------------------------------------
LOC_Os04g59430.1|11670.m05892/1-530                -----------------------------------------------------------------------------------------------------
LOC_Os07g08520.1|11673.m00789/1-729                -----------------------------------------------SSSSAPIIPSRGSAFRIVNPRDGSQGX---------------------------
LOC_Os07g08530.1|11673.m00790/1-408                -----------------------------------------------------------------------------------------------------
LOC_Os07g08600.1|11673.m00797/1-525                -----------------------------------------------------------------------------------------------------
ARF1/1-665                                         -------------------------------------------NKLAVNARMQLKADAEENGNTEGRSSSMAGSR--------------------------
ARF3/1-608                                         -------------------------------------------------SQGRDIYAMRDMLLDIAL----------------------------------
ARF4/1-788                                         --------------------------------------------NKSCLEQAALMMEASKSSSVSQPDSSPTITRV-------------------------
ARF5/1-902                                         --------------------------------------------------GMKLLNSAGINDLKTSVS---------------------------------
ARF6/1-933                                         ---------------------------------------SSNNVDKLPSNGNCDDFGNRSDPRNLGNGIASVGGSFNY-----------------------
ARF7/1-1165                                        ----------------------------------------GNFAGVPVTNQACSGGDSGNAWRGHYDDNSATSFNR-------------------------
ARF8/1-811                                         -------------------------------------------------GEGSGGLFPQNPTHL-------------------------------------
ARF9/1-638                                         -------------------------------------------------NQLRMLLREVETTLTTTSKTDNHSN---------------------------
ARF10/1-693                                        ---------------------------------------------------WITGIRTGENGIDASTKTGPLSIFA-------------------------
ARF11/1-601                                        ----------------------------------------------------SLLGDKGTIVNLESDQRTVHV----------------------------
ARF12/1-593                                        -----------------------------------------------------------------------------------------------------
ARF13/1-623                                        -------------------------------------------------NKFFQPESKALTSSDVPPNVTDN-----------------------------
ARF14/1-605                                        ----------------------------------------------------KSLSS--------------------------------------------
ARF15/1-598                                        -----------------------------------------------------------------------------------------------------
ARF16/1-670                                        -----------------------------------------------------------------------------------------------------
ARF17/1-585                                        -----------------------------------------------------------------------------------------------------
ARF18/1-602                                        -------------------------------------------------LKISSSLENEEYGNESFENRSRG-----------------------------
ARF19/1-1086                                       ----------------------------------------GDLAAIPTTNQACSETDSGNAWKVHYEDTSAAASFNR------------------------
ARF20/1-615                                        ----------------------------------------------------KSLSS--------------------------------------------
ARF21/1-606                                        ----------------------------------------------------KSLSS--------------------------------------------
ARF22/1-600                                        -----------------------------------------------------------------------------------------------------
ARF23/1-222                                        -----------------------------------------------------------------------------------------------------
ARF2/1-859                                         ----------------------------------------TLSCRSEEEAVVGEGSDAKDAKSASNPSLSSAGNS--------------------------
PoptrARF8.1/1-827                                  -------------------------------------------------GVEPSGPNAVHRMSGGVA----------------------------------
PoptrARF1.1/1-660                                  ------------------------------------------IKLSGDEEIKGDSANANADASVNTEDRSSVVGPGC------------------------
PoptrARF7.3/1-1113                                 ----------------------------------------GDFGNSVLPNQAGSSSDNVNA----------------------------------------
PoptrARF2.3/1-792                                  ---------------------------------------LEAEGNSQSADQMVDLMGDGTSKHLPAVSP--------------------------------
PoptrARF5.1/1-933                                  --------------------------------------------------GMKLLNSANIQGINTPITEGIHA----------------------------
PoptrARF5.2/1-944                                  --------------------------------------------------GMKLLNSANIQGTNAPITEGSHAR---------------------------
PoptrARF7.4/1-1137                                 ----------------------------------------GDFGNSVLPNQACSSSDNVNA----------------------------------------
PoptrARF16.1/1-669                                 -----------------------------------------------------------------------------------------------------
PoptrARF1.2/1-662                                  -------------------------------------------IKLLAEEEVKGIGDNADAAVNTEDRSSTIGPGC-------------------------
PoptrARF9.1/1-666                                  -------------------------------------------------SKLPMLALEGEGTVLSSDSAEN------------------------------
PoptrARF2.1/1-854                                  ----------------------------------------TLNSRVNENPSGVEGEDAKEAKHLPLPSASSPLSC--------------------------
PoptrARF16.3/1-700                                 -----------------------------------------------------------------------------------------------------
PoptrARF3.1/1-709                                  --------------------------------------------------CTKAIRQHHAENYRIY-----------------------------------
PoptrARF6.2/1-914                                  ---------------------------------------LNSVPIQRLSNGSCDDYVNRQDSQNLSNAITSVGSLDCREI---------------------
PoptrARF6.4/1-953                                  ---------------------------------------GYMNRQEFRNSSNPDGYLNRQDFRNSSNGIASMGSLDYQGRCS-------------------
PoptrARF17.1/1-594                                 ----------------------------------------------------CQSASAVEACSA-------------------------------------
PoptrARF2.4/1-879                                  ---------------------------------------KHLPAVSPSECQGLCYMTGSKAGKVIQAKWLHHGQDVSFEAVAMPLF---------------
PoptrARF16.2/1-708                                 -----------------------------------------------------------------------------------------------------
PoptrARF2.2/1-852                                  ---------------------------------------ALNSKGVENPMDMEGEDDAKEAKHLPLPSACSPMNC--------------------------
PoptrARF10.1/1-708                                 --------------------------------------------------TWITGMRNAENGLEAPNKTGPLSIFA-------------------------
PoptrARF3.3/1-109                                  -----------------------------------------------------------------------------------------------------
PoptrARF9.2/1-670                                  -------------------------------------------------SKLPKLPIKGEGTVLSSDSAEN------------------------------
PoptrARF6.1/1-884                                  ---------------------------------------LNSFPIQRLSNGSCDDYANRQDSKSSSTGITSVGSLDY------------------------
PoptrARF4/1-713                                    -------------------------------------------------GSSPVIMEASKSSSVGQPDCSPTVIRV-------------------------
PoptrARF6.3/1-163                                  ---------------------------------------LNSVPIQRLSNGSCDDYVNRQDSQNLSNAITSVGSLDCREI---------------------
PoptrARF3.2/1-714                                  -----------------------------------------------------------------------------------------------------
PoptrARF9.3/1-579                                  -------------------------------------------------CKLPASSFEGEETVVSMDSDHRSDA---------------------------
PoptrARF6.5/1-907                                  ---------------------------------------AASVPCQKLSNSNSDGHMNTQGFRNSSNGIASMGSLDY------------------------
PoptrARF9.4/1-632                                  -------------------------------------------------CKLPASSFEGEGTVVSMESEHKSDA---------------------------
PoptrARF7.1/1-1047                                 ----------------------------------------GDLGNVPAPNQACSGTDSGNVWRGQ------------------------------------
PoptrARF7.2/1-1093                                 ----------------------------------------GDLGNVPVPNQACSGTNSGNVWRGQYDDNSAASFNR-------------------------
PoptrARF8.2/1-816                                  -------------------------------------------------GFESSGPNAVKG----------------------------------------
PoptrARF16.6/1-91                                  -----------------------------------------------------------------------------------------------------
PoptrARF16.4/1-701                                 -----------------------------------------------------------------------------------------------------
PoptrARF16.5/1-536                                 -----------------------------------------------------------------------------------------------------
PoptrARF10.2/1-713                                 --------------------------------------------------RWITGMRNAENGQEAPNKTGCITQCGI------------------------
PoptrARF17.2/1-592                                 --------------------------------------------------EACPT----------------------------------------------
PoptrARF2.6/1-724                                  ---------------------------------------------------SPNPSPSG------------------------------------------
PoptrARF2.5/1-614                                  -----------------------------------------------------------------------------------------------------
```

Consensus

```
---------------------------------------LGD+GNSPLPNQACSLSDAGEA+RNSSDSISSVGSLDYXEISS-E-----T-R--------X
```
