# Supplementary material for: Genome-wide analysis of Aux/IAA and ARF gene families in Populus trichocarpa
Source: BMC Plant Biol. 2007 Nov 6;7:59. doi: 10.1186/1471-2229-7-59 (PMC2174922; doi:10.1186/1471-2229-7-59)
Supplement: Additional File 11 — Multiple sequence alignment of conserved regions of predicted Populus, Arabidopsis and rice ARF protein sequences. Sequences were aligned using MUSCLE program. Consensus sequence is indicated at the bottom of the alignment. [file 1471-2229-7-59-S11.pdf]

```
                                                      10         20         30         40         50         60         70         80         90         100
LOC_Os01g70270.1|11667.m07038_altsplice/1-809    PFCKTLTASDTSTHGGFSVLRRHADECLPPLDMTQSPPTQELVAKDLHSMDWRFRHIFRGQPRRHLLQSGWSVFVSSKRLVAGDAFIFLRGENGELRVGVRRAMRQ
LOC_Os01g13520.1|11667.m01333/1-700              PFCKILTPSDTSTHGGFSVLRRHANECLPPLDMSMATPTQELITKDLHGSEWRFKHIYRGQPRRHLLTTGWSTFVTSKKLISGDAFVYLRSETGEQRVGVRRLVQK
LOC_Os02g06910.1|11668.m00642/1-909              PFCKTLTASDTSTHGGFSVPRRAAEKVFPPLDFTQQPPAQELMAKDLHGNEWKFRHIFRGQPKRHLLTTGWSVFVSAKRLVAGDSVLFIWNDSNQLLLGIRRANRP
LOC_Os02g35140.1|11668.m03331/1-757              PFCKTLTASDTSTHGGFSVLRRHAEECLPPLDMTQNPPWQELVARDLHGNEWHFRHIFRGQPRRHLLTTGWSVFVSSKRLVAGDAFIFLRGENGELRVGVRRLMRQ
LOC_Os02g41800.1|11668.m03990/1-784              PFAKTLTQSDANNGGGFSVPRYCAETIFPRLDYAADPPVQTVVAKDVHGVAWNFRHIYRGTPRRHLLTTGWSTFVNQKKLVAGDSIVFLRGDGGDLHVGIRRAKRG
LOC_Os04g57610.1|11670.m05695/1-819              PFCKTLTASDTSTHGGFSVPRRAAERVFPPLDFTQQPPAQELIARDIHDIEWKFRHIFRGQPKRHLLTTGWSVFVSAKRLVAGDSVLFIWNEKNQLLLGIRRASRP
LOC_Os04g43910.1|11670.m04249/1-696              PFAKTLTQSDANNGGGFSVPRFCAETIFPELDYSSEPPVQSVCAKDVHGVEWTFRHIYRGTPRRHLLTTGWSPFVNKKQLTAGDSIVFMRDEGGNIHVGLRRAKRG
LOC_Os04g49230.1|11670.m04840/1-294              PFDKVVTPSDVGKLNRLVIPKQHAEKYFP-LDSAANEKGLLLSFEDRTGKLWRFRYSYWNSSQSYVMTKGWSRFVKEKRLDAGDTVSFCRA--------------
LOC_Os04g36060.1|11670.m03490/1-1674             PFCKTLTASDTSTHGGFSVLRRHAEECLPQLDMSQNPPCQELVAKDLHGTEWHFRHIFRGQPRRHLLTTGWSVFVSSKRLVAGDAFIFLRGESGELRVGVRRLMRQ
LOC_Os05g43920.1|11682.m04181/1-700              AFCKTLTASDTSTHGGFSVPRRAAEDCFPPLDYKTVRPSQELIAVDLHGTQWKFRHIYRGQPRRHLLTIGWSSFVNRKKLVSGDAVLFLRGDDGQLRLGVRRAVQL
LOC_Os06g09660.1|11680.m00929/1-1056             PFCKTLTASDTSTHGGFSVPRRAAEKIFPPLDFTMQPPAQELIAKDLHDISWKFRHIYRGQPKRHLLTTGWSVFVSTKRLLAGDSVLFIRDEKSQLLLGIRRATRP
LOC_Os06g46410.1|11680.m04579/1-918              PFCKTLTASDTSTHGGFSVPRRAAEKVFPPLDFTQQPPAQELIAKDLHGNEWKFRHIFRGQPKRHLLTTGWSVFVSAKRLVAGDSVLFIWNDNNQLLLGIRRANRP
LOC_Os06g47150.1|11680.m04656/1-731              PFAKTLTQSDANNGGGFSVPRYCAETIFPRLDYSADPPVQTVLAKDVHGVVWKFRHIYRGTPRRHLLTTGWSTFVNQKKLVAGDSIVFMRTENGDLCVGIRRAKKG
LOC_Os06g48950.1|11680.m04850/1-1116             PFCKTLTASDTSTHGGFSVPRRAAEKIFPPLDFSMQPPAQELQARDLHDNVWTFRHIYRGQPKRHLLTTGWSLFVSGKRLFAGDSVIFVRDEKQQLLLGIRRANRQ
LOC_Os08g40900.1|11674.m04106_altsplice/1-1056   PFCKTLTASDTSTHGGFSVPRRAAERIFPRLDFSMQPPAQELQARDLHDNVWTFRHIYRGQPKRHLLTTGWSLFVSGKRLLAGDSVLFIRDAKQQLLLGIRRANRQ
LOC_Os11g32110.1|11687.m02952/1-812              PFCKTLTASDTSTHGGFSVLRRHADECLPPLDMSRQPPTQELVAKDLHGVEWRFRHIFRGQPRRHLLQSGWSVFVSAKRLVAGDAFIFLRGENGELRVGVRRAMRQ
LOC_Os12g41950.1|11686.m04180/1-900              PFCKTLTASDTSTHGGFSVPRRAAEKVFPPLDFSQQPPAQELIARDLHDNEWKFRHIFRGQPKRHLLTTGWSVFVSAKRLVAGDSVIFIWNDNNQLLLGIRRANRQ
LOC_Os12g29520.1|11686.m02861_altsplice/1-842    PFCKTLTASDTSTHGGFSVLRRHADECLPPLDMSQHPPTQELVAKDLHGVEWRFRHIFRGQPRRHLLQSGWSVFVSAKRLVAGDAFIFLRGENGELRVGVRRAMRQ
LOC_Os04g56850.1|11670.m05610_altsplice/1-956    PFCKNLTASDTSTHGGFSVPRRAAEKLFPQLDYSMQPPNQELIVRDLHDNMWTFRHIYRGQPKRHLLTTGWSLFVGAKRLKAGDSVLFIRDEKSQLLLGVRRATRQ
LOC_Os05g48870.1|11682.m04683_altsplice/1-696    PFCKTLTASDTSTHGGFSVPRRAAEDCFPPLDYSQQRPSQELVAKDLHSTEWRFRHIYRGQPRRHLLTTGWSAFVNKKKLVSGDAVLFLRGDDGELRLGVRRAAQL
LOC_Os01g48060.1|11667.m04657_altsplice/1-723    AFCKTLTASDTSTHGGFSVPRRAAEDCFPPLDYSLQRPFQELVAKDLHGTEWRFRHIYRGQPRRHLLTTGWSGFINKKKLVSGDAVLFLRGEDGELRLGVRRAAQL
LOC_Os01g54990.1|11667.m05421/1-656              GFCKTLTASDTSTHGGFSVPRRAAEDCFPPLDHKQLRPSQELVAKDLHGAKWRFRHIYRGQPRRHLLTTGWSSFVNKKKLVSGDAVLFLRGDDGELRLGVRRATQL
LOC_Os10g33940.1|11676.m02996/1-699              PFAKTLTQSDANNGGGFSVPRYCAETIFPKLDYRADPPVQTVLAKDVHGVVWKFRHIYRGTPRRHLLTTGWSTFVNQKKLVAGDSIVFLRTRHGELCVGIRRAKRM
LOC_Os02g04810.1|11668.m00419/1-1094             PFCKTLTASDTSTHGGFSVPRRAAEKIFPPLDFSMQPPAQELQARDIHDNVWTFRHIYRGQPKRHLLTTGWSLFVSGKRLFAGDSVIVVR---------------
LOC_Os04g59430.1|11670.m05892/1-530              PYAKQLTQSDANNGGGFSVPRLCADHIFPALNLDDDPPVQSLTMGDLQGDSWEFRHIYRGTPRRHLLTTGWSKFVNAKQLVAGDTVVFMWGPERKLLVGVRRAARY
LOC_Os07g08520.1|11673.m00789/1-729              PFEKQLSPADV-TSNALVLP-AGAEHVLPPLDIAAYQTARLFDVRDLRGKRFEFVHIWDKKRCRYMLNDGWRGFVKAKRLATRDTVVFMRGGDGELLVGVRRAPRA
LOC_Os07g08530.1|11673.m00790/1-408              PFVKTLMISDFDFRIRFSAPMADAKGVFPPL--VDAKAVQPLLVKDLHGSPMTFDYGRKG--KRVTLAKVWKKFRDDMDFVDGDSVIFMRRDDGELYVGVRRQRTL
LOC_Os07g08600.1|11673.m00797/1-525              PFVKPLTYTDV-TKNRFMVPKDAAAGVLPHIQLNDDVP---LRIKDLSGKEWAFNYTWK--AHTRMFRNGWMEFSNANGLVTGDNAVFMRRGNGEMFMAVRRTRNR
ARF1/1-665                                       PFCKTLTASDTSTHGGFSVLRRHADDCLPPLDMSQQPPWQELVATDLHNSEWHFRHIFRGQPRRHLLTTGWSVFVSSKKLVAGDAFIFLRGENEELRVGVRRHMRQ
ARF3/1-608                                       PFCKTLTASDTSTHGGFSVPRRAAEDCFPPLDYSQPRPSQELLARDLHGLEWRFRHIYRGQPRRHLLTTGWSAFVNKKKLVSGDAVLFLRGDDGKLRLGVRRASQI
ARF4/1-788                                       PFCKTLTASDTSTHGGFSVPRRAAEDCFAPLDYKQQRPSQELIAKDLHGVEWKFRHIYRGQPRRHLLTTGWSIFVSQKNLVSGDAVLFLRDEGGELRLGIRRAARP
ARF5/1-902                                       PFCKTLTASDTSTHGGFSVPRRAAEKLFPPLDYSAQPPTQELVVRDLHENTWTFRHIYRGQPKRHLLTTGWSLFVGSKRLRAGDSVLFIRDEKSQLMVGVRRANRQ
ARF6/1-933                                       PFCKTLTASDTSTHGGFSVPRRAAEKVFPPLDYSQQPPAQELMARDLHDNEWKFRHIFRGQPKRHLLTTGWSVFVSAKRLVAGDSVLFIWNDKNQLLLGIRRANRP
ARF7/1-1165                                      PFCKTLTASDTSTHGGFSVPRRAAEKIFPALDFSMQPPCQELVAKDIHDNTWTFRHIYRGQPKRHLLTTGWSVFVSTKRLFAGDSVLFIRDGKAQLLLGIRRANRQ
ARF8/1-811                                       PFCKTLTASDTSTHGGFSVPRRAAEKVFPPLDYTLQPPAQELIARDLHDVEWKFRHIFRGQPKRHLLTTGWSVFVSAKRLVAGDSVIFIRNEKNQLFLGIRHATRP
ARF9/1-638                                       PFSKVLTASDTSTHGGFSVLRKHATECLPPLDMTQQTPTQELVAEDVHGYQWKFKHIFRGQPRRHLLTTGWSTFVTSKRLVAGDTFVFLRGENGELRVGVRRANLQ
ARF10/1-693                                      PFAKTLTQSDANNGGGFSVPRYCAETIFPRLDYSAEPPVQTVIAKDIHGETWKFRHIYRGTPRRHLLTTGWSTFVNQKKLIAGDSIVFLRSESGDLCVGIRRAKRG
ARF11/1-601                                      PFVKILTASDTSTHGGFSVLRKHATECLPSLDMTQPTPTQELVARDLHGYEWRFKHIFRGQPRRHLLTTGWSTFVTSKRLVAGDAFVFLRGETGDLRVGVRRLAKQ
ARF12/1-593                                      PFTKVLTASDTSAHGGFFVPKKHAIECLPSLDMSQPLPAQELLAIDLHGNQWRFNHNYRGTPQRHLLTTGWNAFTTSKKLVAGDVIVFVRGETGELRVGIRRARHQ
ARF13/1-623                                      PFSKILTASDVSLSGGLIIPKQYAIECFPPLDMSQPISTQNLVAKDLYGQEWSFKHVFRGTPQRHMFTSGWSVFATTKRLIVGDIFVLLRGENGELRFGIRRAKHQ
ARF14/1-605                                      PFTKVLTASDTSVHGGFSVPKKHAIECLPPLDMSQPLPTQEILAIDLHGNQWRFRHIYRGTAQRHLLTIGWNAFTTSKKLVEGDVIVFVRGETGELRVGIRRAGHQ
ARF15/1-598                                      PFTKVLTASDISANGVFSVPKKHAIECLPPLDMSQPLPAQELLAIDLHGNQWSFRHSYRGTPQRHLLTTGWNEFTTSKKLVKGDVIVFVRGETGELRVGIRRARHQ
ARF16/1-670                                      PFAKTLTQSDANNGGGFSVPRYCAETIFPRLDYNAEPPVQTILAKDVHGDVWKFRHIYRGTPRRHLLTTGWSNFVNQKKLVAGDSIVFMRAENGDLCVGIRRAKRG
ARF17/1-585                                      PFAKILTPSDANNGGGFSVPRFCADSVFPLLNFQIDPPVQKLYVTDIHGAVWDFRHIYRGTPRRHLLTTGWSKFVNSKKLIAGDSVVFMRKSADEMFIGVRRTPIS
ARF18/1-602                                      PFVKILTASDTSTHGGFSVLRKHATECLPSLDMTQATPTQELVTRDLHGFEWRFKHIFRGQPRRHLLTTGWSTFVSSKRLVAGDAFVFLRGENGDLRVGVRRLARH
ARF19/1-1086                                     PFCKTLTASDTSTHGGFSVPRRAAEKIFPPLDFSMQPPAQEIVAKDLHDTTWTFRHIYRGQPKRHLLTTGWSVFVSTKRLFAGDSVLFVRDEKSQLMLGIRRANRQ
ARF20/1-615                                      PFTKVLTASDTSAYGGFFVPKKHAIECLPPLDMSQPLPAQELLAKDLHGNQWRFRHSYRGTPQRHSLTTGWNEFTTSKKLVKGDVIVFVRGETGELRVGIRRARHQ
ARF21/1-606                                      PFTKVLTASDTSAYGGFSVPKKHAIECLPPLDMSQPLPAQEILAIDLHDNQWRFRHNYRGTPQRHSLTTGWNEFITSKKLVKGDVIVFVRGETGELRVGIRRARHQ
ARF22/1-600                                      PFTKVLTASDTS--GGFFVPKKHAIECLPPLDMSQPLPTQELLATDLHGNQWRFNHNYRGTPQRHLLTTGWNAFTTSKKLVAGDVIVFVRGETGELRVGIRRAGHQ
ARF23/1-222                                      PFTKVLTASDTSAQGEFSVPCKHAIECLPPLDMSQPIPAQELIAIDLHGNQWRFKHSYR-VPRGD--TTGWNAFTTSKKLVVGDVIVFARGETGELRVGIR-----
ARF2/1-859                                       PFCKTLTASDTSTHGGFSVLRRHADECLPPLDMSRQPPTQELVAKDLHANEWRFRHIFRGQPRRHLLQSGWSVFVSSKRLVAGDAFIFLRGENGELRVGVRRAMRQ
PoptrARF8.1/1-827                                PFCKTLTASDTSTHGGFSVPRRAAEKVFPPLDFTQQPPAQELIARDLHDVEWKFRHIFRGQPKRHLLTTGWSVFVSAKRLVAGDSVLFIWNEKNQLLLGIRRATRP
PoptrARF1.1/1-660                                PFCKTLTASDTSTHGGFSVLRRHADDCLPPLDMSQQPPWQELVATDLHGNEWHFRHIFRGQPRRHLLTTGWSVFVSSKKLVAGDAFIFLRGENGELRVGVRRLMRQ
PoptrARF7.3/1-1113                               PFCKTLTASDTSTHGGFSVPRRAAEKTFPPLDFSMQPPAQELVARDLHDNVWTFRHIYRGQPKRHLLTTGWSLFVSGKRLFAGDSVLFMRDEKQQLLLGIRRANRQ
PoptrARF2.3/1-792                                PFCKMLTASDTSTHGGFSVLRRHADECLPPLDMSLQPPAQELVAKDLHGNEWRFRHIFRGQPRRHLLQSGWSLFVSAKKLVAGDAFIFLRGETEELRVGVRRALSQ
PoptrARF5.1/1-933                                PFCKALTASDTSTHGGFSVPRRAAEKLFPPLDYSMQPPSQELVVRDLHDNTWTFRHIYRGQPKRHLLTTGWSLFVGSKRLKAGDSVLFIRNEKSHLMVGVRHANRQ
PoptrARF5.2/1-944                                PFCKTLTASDTSTHGGFSVPRRAAEKLFPPLDYTMQPPTQELVVRDLHDNTWTFRHIYRGQPKRHLLTTGWSLFVGSKRLKAGDSVLFIRDEKSQLMVGVRRANRQ
PoptrARF7.4/1-1137                               PFCKTLTASDTSTHGGFSVPRRAAEKIFPPLNFSLQPPAQELVARDLHDNVWTFRHIYRGQPKRHLLTTGWSLFVSGKRLFAGDSVLFIRDEKQQLLLGIRRANRQ
PoptrARF16.1/1-669                               PFAKTLTQSDANNGGGFSVPRYCAETIFPRLDYTAEPPVQTILAKDVHGETWKFRHIYRGTPRRHLLTTGWSNFVNQKKLVAGDSIVFLRAENGDLCVGIRRAKRG
PoptrARF1.2/1-662                                PFCKTLTASDTSTHGGFSVLRRHADDCLPPLDMSQQPPWQELVATDLHGNEWHFRHIFRGQPRRHLLTTGWSVFVSSKKLVAGDAFIFLRGENGELRVGVRRLMRQ
PoptrARF9.1/1-666                                PFCKVLTASDTSTHGGFSVLRKHASECLPPLDMIQPIPTQELVAKDLHGYEWRFKHIFRGQPRRHLLTTGWSTFVTSKRLVAGDSFVFLRGENGELRVGVRRVARQ
PoptrARF2.1/1-854                                PFCKTLTASDTSTHGGFSVLRRHADECLPPLDMSRQPPTQELVAKDLHGSEWRFRHIFRGQPRRHLLQSGWSVFVSSKRLVAGDAFIFLRGENGELRVGVRRAMRQ
PoptrARF16.3/1-700                               PFAKTLTQSDANNGGGFSVPRYCAEMIFPRLDYTADPPVQTLLAKDVHGETWKFRHIYRGTPRRHLLTTGWSPFVNHKKLVAGDSVVFLRAENGDLCVGVRRAKRA
PoptrARF3.1/1-709                                PFCKTLTASDTSTHGGFSVPRRAAEDCFPPLDYTQQRPSQELVAKDLHGSEWKFRHIYRGQPRRHLLTTGWSAFVNKKKLVSGDAVLFLRGEDGELRLGVRRAAQV
PoptrARF6.2/1-914                                PFCKTLTASDTSTHGGFSVPRRAAEKVFPPLDFSQQPPAQELIARDLHDNEWKFRHIFRGQPKRHLLTTGWSVFVSAKRLIAGDSVLFIWNEKNQLLLGIKRATRP
PoptrARF6.4/1-953                                PFCKTLTASDTSTHGGFSVPRRAAEKVFPPLDFSQTPPAQELIARDLHDNEWKFRHIFRGQPKRHLLTTGWSVFVSAKRLVAGDSVLFIWNEKNQLLLGIRRANRP
PoptrARF17.1/1-594                               PFSKILTPSDANNGGGFSVPRFCADSIFPPLNYQAEPPVQTLTVADIHGVSWDFRHIYRGTPRRHLLTTGWSKFVNNKKLIAGDSVVFMRNLKGEMFIGVRRAVRF
PoptrARF2.4/1-879                                PFCKMLTASDTSTHGGFSVLKRHADECLPPLDMSLQPPVQELVAKDLHGNEWRFRHIFRGQPRRHLLQSGWSLFVSAKKLVAGDAFIFLRGETEELRVGVRRALRQ
PoptrARF16.2/1-708                               PFAKTLTQSDANNGGGFSVPRYCAETIFPRLDYTAEPPVQTILAKDVHGETWKFRHIYRGTPRRHLLTTGWSNFVNQKKLVAGDSIVFLRAENGDLCVGIRRAKRG
PoptrARF2.2/1-852                                PFCKTLTASDTSTHGGFSVLRRHADECLPPLDMSRQPPTQELVAKDLHGNEWRFRHIFRGQPRRHLLQSGWSVFVSSKRLVAGDAFIFLRGENGELRVGVRRAMRQ
PoptrARF10.1/1-708                               PFAKTLTQSDANNGGGFSVPRYCAETIFPRLDYSADPPVQTLIAKDVHGEVWKFRHIYRGTPRRHLLTTGWSTFVNQKKLVAGDSIVFLRAENGDLCVGIRRAKRG
PoptrARF3.3/1-109                                --------------------HFIEKSLPLLD----------------------------------TE-----------HVGVVYNFLPC--------------
PoptrARF9.2/1-670                                PFCKVLTASDTSTHGGFSVLRKHATECLPPLDMTQPTPTQELVAKDLHGYEWRFKHIFRGQPRRHLLTTGWSTFVTSKRLVAGDSFVFLRGENGELRVGVRRVACQ
PoptrARF6.1/1-884                                PFCKTLTASDTSTHGGFSVPRRAAEKVFPPLDFSQHPPAQELIARDLHDNEWKFRHIFRGQPKRHLLTTGWSVFVSAKRLVAGDSVLFIWNEKNQLLLGIRRATRP
PoptrARF4/1-713                                  PFCKTLTASDTSTHGGFSVPRRAAEDCFPSLDYKQQRPSQELLAKDLHGVEWRFRHIYRGQPRRHLLTTGWSIFVSQKNLVSGDAVLFLRGEGGELRLGIRRAARP
PoptrARF6.3/1-163                                -----MAPSSCIDESGFL---QSMENV------------------------------GQ----------------------------G--------------
PoptrARF3.2/1-714                                PFCKTLTASDTSTHGGFSVPRRAAEDCFPPLDYTQQRPSQELVAKDLHGSEWKFRHIYRGQPRRHLLTTGWSAFVNKKKLVSGDAVLFLRGEDGELRLGVRRAAQV
PoptrARF9.3/1-579                                PFCKILTASDTSTHGGFSVLRKHATECLPPLDMSQATPTQELAARDLHGFEWRFKHIFRGQPRRHLLTTGWSTFVTSKRLVAGDAFVFLRGHNRELRVGVRRLARQ
PoptrARF6.5/1-907                                PFCKTLTASDTSTHGGFSVPRRAAEKVFPTLDYSQTPPAQELIARDLHDNEWKFRHIFRGQPKRHLLTTGWSVFVSAKRLVAGDSVLFIWNEKNQLLLGIRRANRP
PoptrARF9.4/1-632                                PFCKILTASDTSTHGGFSVLRKHATECLPPLDMSQATPTQELAARDLHGYEWRFKHIFRGQPRRHLLTTGWSTFVTSKRLVAGDSFVFLRGDNGELRVGLRRVARQ
PoptrARF7.1/1-1047                               PFCKTLTASDTSTHGGFFVPRRAAEKIFPPLDFSMQPPAQELVARDLHDNTWTFRHIYRGQPKRHLLTTGWSVFVSTKRIFTGDSVLFIRDEKSQLLLGIRHANRQ
PoptrARF7.2/1-1093                               PFCKTLTASDTSTHGGFSVPRRAAEKIFPPLDFSMQPPAQELVARDLHDNTWTFRHIYRGQPKRHLLTTGWSVFVSTKRLFAGDSVLFIRDEKSQLLLGIRRANRQ
PoptrARF8.2/1-816                                PFCKTLTASDTSTHGGFSVPRRAAEKVFPPLDFTQQPPAQELIARDLHDVEWKFRHIFRGQPKRHLLTTGWSVFVSAKRLVAGDSVLFIWNEKNQLLLGIRRATRP
PoptrARF16.6/1-91                                P-------------------ILESTTFSRLDYTAEPPEETILAKDVHGETWKFRHIYRGAPRRHLLNTGWSNFVNKKNSWLGTRLCF-----------------
PoptrARF16.4/1-701                               PFAKTLTQSDANNGGGFSVPRYCAEMIFPRLDYTADPPVQTLLAKDVHGETWKFRHIYRGTPRRHLLTTGWSPFVNHKKLIAGDSVVFFRAENGDLCVGVRRAKRT
PoptrARF16.5/1-536                               -FAKTLTQSDANNGGGFSVPRYCAEMIFPRLDYTADPPVQTLLAKDVHGETWKFRHIYRGTPRRHLLTTGWSPFVNHKKLIAGDSVVFFRAENGDLCVGVRRAKRT
PoptrARF10.2/1-713                               PFAKTLTQSDANNGGGFSVPRYCAETIFPRLDYSSDPPLQTVIAKDVHGEVWKFRHIYRGTPRRHLLTTGWSTFVNQKKLVAGDSIVFLRAENGDLRVGIRRSKRG
PoptrARF17.2/1-592                               PFAKILTPSDANNGGGFSVPRFCADSIFPPLNYQAEPPVQTLTVTDIHGISWDFRHIYRGTPRRHLLTTGWSKFVNNKKLIAGDSVVFMRNLKGEMFIGVRRAVRL
PoptrARF2.6/1-724                                PFTKKLTPSDTKTHGGFSVPKRHADQCLPPLDKSQQPPVQELLAKDLHGFEWCFKHIYRGQPKRHLITSGWSTFVSSKRLVAGDSFIFLRGESGELRVGVRRAMKL
PoptrARF2.5/1-614                                PFTKKLTPSDTSTQGGFSVPKRHAEESLPPLDKSQQPPAQELLAKDLHGSEWRFRHIYRGQPKRHLLTGGWSTFISSKRVVAGDSFIFLRGESGELRVGVRRAMKL
```

Consensus

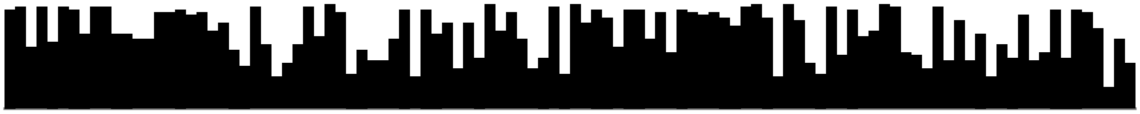

```
PFCKTLTASDTSTHGGFSVPRRAAEECFPPLDYSQQPPAQELVAKDLHGNEWKFRHIYRGQPRRHLLTTGWSVFVSSKRLVAGDSVVFLRGENGELRVGVRRANRQ
```

```
                                                        110       120       130       140       150       160       170       180       190       200       210
LOC_Os01g70270.1|11667.m07038_altsplice/1-809    LSQSMHLGVLATAWHAINTKSMFTVYYKPRTSPSEFIIPYDQYMESVKNNYSVGMRFRMRFEGEEAPEQRFTGTIIGSENLDPV-WPESSWRSLKVRWDEPSRPDR
LOC_Os01g13520.1|11667.m01333/1-700              QSQSMHLGVLASASHAIKTNSIFLVYYRPRLSQSQYIVSVNKYLAASKVGFNVGMRFKMSFEGEDVPVKKFSGTIVGEGDLSL-QWSGSEWKSLKVQWDEVTGPER
LOC_Os02g06910.1|11668.m00642/1-909              QSDSMHIGLLAAAAHAASTNSRFTIFYNPRASPSEFVIPLAKYVKAVYHRISVGMRFRMLFETEESSVRRYMGTITGISDLDPVRWMNSHWRSVKVGWDESTRQPR
LOC_Os02g35140.1|11668.m03331/1-757              LSHSMHLGVLATASHAISTGTLFSVFYKPRTSQSEFVVSANKYLEAKNSKISVGMRFKMRFEGDEAPERRFSGTIIGVGSMSTSPWANSDWRSLKVQWDEPSRPDR
LOC_Os02g41800.1|11668.m03990/1-784              FRGKVRAEDLVEAARLANGGQPFEVVYYPRASTPEFCVRAAAVRAAMRVQWCPGMRFKMAFETEDSSRISFMGTVASVQVADPIRWPQSPWRLLQVTWDEPDNVKR
LOC_Os04g57610.1|11670.m05695/1-819              QSDSMHIGLLAAAAHAAATNSRFTIFYNPRASPSEFVIPLSKYIKAVFHRISVGMRFRMLFETEESSVRRYMGTITEVSDADPVRWPSSYWRSVKVGWDESTRPPR
LOC_Os04g43910.1|11670.m04249/1-696              FKGKVPPENVLTAATRATTGQPFEVLYYPRASTPEFCVRAAAVRTAMAVQWCPGMRFKMAFETEDSSRISFMGTVAGVQASDPVRWPQSPWRLLQVTWDEPENVKR
LOC_Os04g49230.1|11670.m04840/1-294              -AEATRDRLFIDWKRRADVRDPHRFQRLP----LPMTSPYG--------PWGGG--------------------AGASSCRPR-------------------RPPR
LOC_Os04g36060.1|11670.m03490/1-1674             VSHSMHLGVLATASHAISTGTLFSVFYKPRTSRSEFVVSVNKYLEAKKQNLSVGMRFKMRFEGDEAPERRFSGTIIGIGSVPAMPWADSDWKSLKVQWDEPSRPDR
LOC_Os05g43920.1|11682.m04181/1-700              RSSDSKLRILSSVASSLENKSVFHICFNPRSGASEFIVPYWRLLKSLNHPFSIGMRFRVCYESEDANER-SAGLISGISEVDPIRWPGSRWKCLLVRWDDSTHQNR
LOC_Os06g09660.1|11680.m00929/1-1056             QSDSMHIGILAAAAHAAANSSPFTIFYNPRASPSEFVIPLAKYNKALYTQVSLGMRFRMLFETEDSGVRRYMGTITGIGDLDPVRWKNSHWRNLQVGWDESTRRTR
LOC_Os06g46410.1|11680.m04579/1-918              QSDSMHIGLLAAAAHAASTNSRFTIFYNPRASPSEFVIPLSKYVKAVYHRISVGMRFRMLFETEESSVRRYMGTITGISDLDAARWPNSHWRSVKVGWDESTRQPR
LOC_Os06g47150.1|11680.m04656/1-731              GRARVRPEEVVEAANLAVSGQPFEVVYYPRASTPEFCVKAGAVRAAMRTQWFAGMRFKMAFETEDSSRISFMGTVSAVQVADPIRWPNSPWRLLQVSWDEPDNVKR
LOC_Os06g48950.1|11680.m04850/1-1116             PSDSMHIGILAAAAHAAANNSPFTIFYNPRASPTEFVIPFAKYQKAVYGQISLGMRFRMMFETEELGTRRYMGTITGISDLDPVRWKNSQWRNLQVGWDESARRNR
LOC_Os08g40900.1|11674.m04106_altsplice/1-1056   PSDSMHIGILAAAAHAAANNSQFTIYYNPRASTSEFVIPFAKYQKAVYGQLSLGMRFRMMFETEESGTRRYMGTITGISDLDPVRWKTSHWRNIQVAWDEAARRTR
LOC_Os11g32110.1|11687.m02952/1-812              QSHSMHLGVLATAWHAVNTGTMFTVYYKPRTSPAEFVVPYDRYMESLKQNYSIGMRFKMRFEGEEAPEQRFTGTIVGMGDSDPAGWPESKWRSLKVRWDEASRPER
LOC_Os12g41950.1|11686.m04180/1-900              QSDSMHIGLLAAAAHAAATNSRFTIFYNPRASPSEFVIPLAKYVKAVYHRVSVGMRFRMLFETEESSVRRYMGTITSISDLDSVRWPNSHWRSVKVGWDESTKQPR
LOC_Os12g29520.1|11686.m02861_altsplice/1-842    QSHSMHLGVLATAWHAVNTGTMFTVYYKPRTSPSEFVVPRDLYKESLKRNHSIGMRFKMTFEGEEAAEQRFTGTIVGVGDSDPSGWADSKWRSLKVRWDEAARPDR
LOC_Os04g56850.1|11670.m05610_altsplice/1-956    QTDSMHIGVLAAAAHAASSGSSFTIYYNPRTSPSPFVIPVARYNKATYMQPSVGMRFAMMFETEESSKRRYTGTVVGISDYDPMRWPNSKWRNLQVEWDEHGRPER
LOC_Os05g48870.1|11682.m04683_altsplice/1-696    KNQCSNLGTLANVAHAVATESVFNIYYNPRLSQSEFIVPYWKFMKSLSQPFSVGLRFKMRYESEDATERRYTGIITGSGDTDPM-WHGSKWKCLLVRWDDDARPNR
LOC_Os01g48060.1|11667.m04657_altsplice/1-723    KNQISNTSSLSEVAHAVAVKSIFHIYYNPRLSQSEFIIPYWKFMRSFSQPFSVGMRFKLRYESEDASERRRTGIIIGSREADPM-WHGSKWKCLVVKWDDDVRPNG
LOC_Os01g54990.1|11667.m05421/1-656              KSESSKMRTLSAVADSLKHGSVFHICYNPRATASEYVVPYWKFVKSFNHPVCIGMRFKFHFESEDVNERR-SGMIAGVSEVDPIRWPGSKWRSLLVRWEDATSQNR
LOC_Os10g33940.1|11676.m02996/1-699              AKGKVRMADVVEAASLASSGQPFEVAYYPRASTPDFVVKAASVQAAMRIQWCSGMRFKMAFETEDSSRISFMGTISSVQVADPNRWPNSPWRLLQVTWDEPDNVKC
LOC_Os02g04810.1|11668.m00419/1-1094             ------------------------------ASPTEFVIPFAKYQKALYSQISLGMRFRMMFETEELGTRRYMGTITGISDLDPV------------GWDESARRNR
LOC_Os04g59430.1|11670.m05892/1-530              SRGRVQPQEVMEAVRLAAEQAAFRVTYYPRHGAGEFVVPRVEVDKGLTTPWRCGMQVRAQMEAEDTRRLALNGTLTNLRH-------QQIWRTLEVEWDASAMKNR
LOC_Os07g08520.1|11673.m00789/1-729              RPGVEDNKVVSEVWLAMQGVTPFEVTYYPREGTFEFVVSRDEYIGFSFSPFVPGTTVHLRMNPLQIAQS-ISGTVRTFDHLRP-------WRMLEVDWDQAARIHR
LOC_Os07g08530.1|11673.m00790/1-408              EPQAAVQEAVLAAAGHAAAGERFTVAYRSRKDGDEFVVPREAVEEGLRARLTSLAEVEFVWAVEDGAPPIPRGKVTAIAT-------GQLWRNLEIVWDGNSEMDM
LOC_Os07g08600.1|11673.m00797/1-525              -PAPFSVEEVIEAVWRAARREPFEVSYCLRQDGDEFVVPRDIVDDGLRARFAPGMAVNFV----------------------------------------------
ARF1/1-665                                       QSHSMHIGVLATAAHAITTGTIFSVFYKPRTSRSEFIVSVNRYLEAKTQKLSVGMRFKMRFEGEEAPEKRFSGTIVGVQENKSSVWHDSEWRSLKVQWDEPSRPER
ARF3/1-608                                       ENQNMNHNNFSEVAHAISTHSVFSISYNPKASWSNFIIPAPKFLKVVDYPFCIGMRFKARVESEDASERRSPGIISGISDLDPIRWPGSKWRCLLVRWDDIVHQQR
ARF4/1-788                                       REKNSCSNILSLVANAVSTKSMFHVFYSPRATHAEFVIPYEKYITSIRSPVCIGTRFRMRFEMDDSPERRCAGVVTGVCDLDPYRWPNSKWRCLLVRWDESFHQER
ARF5/1-902                                       QADSMHIGVLAAAAHATANRTPFLIFYNPRACPAEFVIPLAKYRKAICGQLSVGMRFGMMFETEDSGKRRYMGTIVGISDLDPLRWPGSKWRNLQVEWDEPGKPTR
ARF6/1-933                                       QSDSMHLGLLAAAAHAAATNSRFTIFYNPRASPSEFVIPLAKYVKAVYHRVSVGMRFRMLFETEESSVRRYMGTITGICDLDPTRWANSHWRSVKVGWDESTRQPR
ARF7/1-1165                                      QSDSMHIGVLAAAAHANANNSPFTIFYNPRAAPAEFVVPLAKYTKAMYAQVSLGMRFRMIFETEECGVRRYMGTVTGISDLDPVRWKNSQWRNLQIGWDESARPSR
ARF8/1-811                                       QSDSMHIGLLAAAAHASATNSCFTVFFHPRASQSEFVIQLSKYIKAVFHRISVGMRFRMLFETEESSVRRYMGTITGISDLDSVRWPNSHWRSVKVGWDESTRQPR
ARF9/1-638                                       QSHSMHLGVLATARHATQTKTMFIVYYKPRT--SQFIISLNKYLEAMSNKFSVGMRFKMRFEGEDSPERRYSGTVIGVKDCSP-HWKDSKWRCLEVHWDEPARPNK
ARF10/1-693                                      GTGRVRVEAVAEAVARAACGQAFEVVYYPRASTPEFCVKAADVRSAMRIRWCSGMRFKMAFETEDSSRISFMGTVSAVQVADPIRWPNSPWRLLQVAWDEPDNVKR
ARF11/1-601                                      QSQSMRLGVLATASHAVTTTTIFVVFYKPRI--SQFIISVNKYMMAMKNGFSLGMRYRMRFEGEESPERIFTGTIIGSGDLSS-QWPASKWRSLQIQWDEPSRPNK
ARF12/1-593                                      QIDCMRHGVVASAKHAFDNQCMFTVVYKPRS--SKFIVSYDKFLDAVNNKFNVGSRFTMRLEGDDFSERRCFGTIIGVSDFSP-HWKCSEWRSLEVQWDEFTGPKK
ARF13/1-623                                      QANCMQHGVIASVVNAFKTKCMFNVVYKP-SS-SQFVISYDKFVDAMNNNYIVGSRFRMQFEGKDFSEKRYDGTIIGVNDMSP-HWKDSEWRSLKVQWDELSRPNQ
ARF14/1-605                                      QIESMRHGIIASAKHAFDNQCMFIVVYKPRS--SQFIVSYDKFLDVVNNKFNVGSRFTMRFEGDDFSERRSFGTIIGVSDFSP-HWKCSEWRSLEVQWDEFARPNQ
ARF15/1-598                                      QIDCMRHGVIASAKHAFDNQCMFIVVYKPRS--SQFIVSYDKFLDAVNNKFNVGSRFTMRFEGDDLSERRYFGTIIGVSNFSP-HWKCSDWRSLEVQWDEFARPNK
ARF16/1-670                                      GKGKVTAESVIEAATLAISGRPFEVVYYPRASTSEFCVKALDARAAMRIPWCSGMRFKMAFETEDSSRISFMGTVSAVNVSDPIRWPNSPWRLLQVAWDEPDNVKR
ARF17/1-585                                      SNGKLTAEAVTDAINRASQGLPFEVVFYPAAGWSEFVVRAEDVESSMSMYWTPGTRVKMAMETEDSSRITFQGIVSSTYQETG-PWRGSPWKQLQITWDEPENVKR
ARF18/1-602                                      QSQSMHLGVLATASHAVRTTTIFVVFYKPRI--SQFIVGVNKYMEAIKHGFSLGTRFRMRFEGEESPERIFTGTIVGSGDLSS-QWPASKWRSLQVQWDEPTRPDK
ARF19/1-1086                                     TSDSMHIGILAAAAHANANSSPFTIFFNPRASPSEFVVPLAKYNKALYAQVSLGMRFRMMFETEDCGVRRYMGTVTGISDLDPVRWKGSQWRNLQVGWDESTRPSR
ARF20/1-615                                      QIDCMRHGVIASAKHALDNQCIFIVVYKPRS--SQFIVSYDKFLDAMNNKFIVGSRFTMRFEGDDFSERRYFGTIIGVNDFSP-HWKCSEWRSLEVQWDEFARPNK
ARF21/1-606                                      QIDCMRHGVIASAKHAFDNQCIFIVVYKPRS--SQFIVSYDKFLDAVNNKFNVGSRFTMRFEGDDFSERRYFGTIIGVSDFSP-HWKCSEWRSLEVQWDEFARPNK
ARF22/1-600                                      QIESMRHGVIASAKHAFDNQCMFIVVYKP-SIRSQFIVSYDKFLDAVNNKFNVGSRFTMRFEGDDFSERRYFGTIIGVSDFSP-HWKCSEWRNLEVQWDEFARPNK
ARF23/1-222                                      ----------------------------------------------------------------------------------------------------------
ARF2/1-859                                       QSHSMHLGVLATAWHAISTGTMFTVYYKPRTSPSEFIVPFDQYMESVKNNYSIGMRFKMRFEGEEAPEQRFTGTIVGIEESDPTRWPKSKWRSLKVRWDETSRPDR
PoptrARF8.1/1-827                                QSDSMHIGLLAAAAHAAATNSCFTVFYNPRASPSEFVIPLSKYVKAVFHRISVGMRFRMLFETEESSVRRYMGTITGISDLDPVRWPNSHWRSVKVGWDESTRQPR
PoptrARF1.1/1-660                                QSQSMHLGVLATASHAIATGTLFSVFYKPRTSRSEFIVNLNKYIEAQNHKLSVGMRFKMRFEGEEVPERRFSGTIVGVGDNISSGWADSEWRSLKVHWDEPSRPER
PoptrARF7.3/1-1113                               PSDSMHIGILAAAAHAAANNSPFTVYYNPRASPSEFVIPLAKYYKAVYSQISLGMRFRMMFETEESGTRRHMGTITGISDLDAVRWKNSQWRNLQVGWDESTRRNR
PoptrARF2.3/1-792                                PSHSMHIGILATVWHAVSTGSMFTVYYKPRTSPAEFIIPIDKYRESVKINYAIGMRFKMKFEAEEAPEQRFSGTVIGVEEADPKKWPRSKWRCLKVRWDETSRPDR
PoptrARF5.1/1-933                                QADSMHIGVLAAAAHAAGNRSPFTIFYNPRACPSDFVIPLIKFRKTVFGQVSVGMRFGMMFETEESGKRRYMGTIVGISDLDPLRWPGSKWRNLQVEWDEPGKQNR
PoptrARF5.2/1-944                                QADSMHIGVLAAAAHATANRSPFTIFYNPRACPSDFVIPLIKFRKAVFGQVSVGMRFGMMFETEESGKRRYMGTIVGISDLDPLRWPGSKWRNLQVEWDEPGKQNR
PoptrARF7.4/1-1137                               PSDSMHIGILAAAAHAAANNSPFTVFYNPRASPSEFVIPLAKYYKAVYSQISLGMRFRMMFETEESGTRRHMGTITGISDLDAVRWKNSQWRNLQVGWDESTRRNR
PoptrARF16.1/1-669                               IKGKVKAESVIEAASLAANGQPFEAVYYPRASTPEFCVKASAVRSAIQIQWCPGMRFKMAFETEDSSRISFMGTISSVQVADPIRWPNSPWRLLQVAWDEPDNVKR
PoptrARF1.2/1-662                                QSQSMHLGVLATASHAIATGTLFSVFYKPRTSRSEFIVSLNKYLEVRNHKLSVGMRFKMRFEGEEVPERRFSGTIVGVGDNISSGWADSDWRSLKVQWDEPSRPER
PoptrARF9.1/1-666                                QSQSMHLGVLATASHAISTLTLFVVYYKPRT--SQFIISLNKYLEAVNNKFAVGMRFKMRFEGEDSPERRFSGTIVGVEDFSP-HWNDSKWRSLKVQWDEPARPDR
PoptrARF2.1/1-854                                QSHSMHLGVLATAWHAVSTGTMFTVYYKPRTSPAEFIVPFDQYMESVKSNYSIGMRFKMRFEGEEAPEQRFTGTIVGIEDADPSRWKDSKWRCLKVRWDETSRPDR
PoptrARF16.3/1-700                               IRGKVRAESVIQAAVLAANGLPFETVYYPRANTPEFFVKASLVKTVMQIRWCSGMRFKMAFETEDSSRISFMGTVCSVQDADPLCWPGSPWRLLQVTWDEPDNVKR
PoptrARF3.1/1-709                                KNQQLNQSSLADVANAISMRSAFRIYYNPRASSSEFIIPFNKFLKSLDQSFSAGMRVKMRFETEDAAERRYTGLITGISELDPTRWPGSKWKCLLVRWDDTERHSR
PoptrARF6.2/1-914                                QSDSMHLGLLAAAAHAAATNSRFTIFYNPRASPSEFIIPLAKYLKAVYYRVSVGMRFRMLFETEESSVRRYMGTITGISDLDVARWPNSLWRSVKVGWDESTRQPR
PoptrARF6.4/1-953                                QSDSMHIGLLAAAAHAAATNSRFTIFYNPRTSPSEFVIPLVKYIKAVYHRVSVGMRFRMLFETEESSVRRYMGTITGISDLDPARWPNSHWRSVKVGWDESTRQPR
PoptrARF17.1/1-594                               NRGRLSQEAVVEAVERAAKGLPFEVVYYPRAGWSDFVVRAEVVEAALRVFWTAGMRVKMAVETEDSSRMTFQGTVSGTGLPDCGAWRGSPWRMLQITWDEPENVKR
PoptrARF2.4/1-879                                PSHSMHIGILATAWHAVSTGSMFTVYYKPRTSPAEFIIPVDKYMESVKINYAIGMRFKMRFEADDAPEQRFSGTVIGVEEADPKKWPRSNWRCLKVHWDETSRPDR
PoptrARF16.2/1-708                               IKGKVKPESVIEAASLAANGQPFEVVYYPRASTPEFCVRASAVRTAMHIQWCPGMRFKMAFETEDSSRISFMGTISSVQFADPIRWPNSPWRLLQVAWDEPDNVKR
PoptrARF2.2/1-852                                QSHSMHLGVLATAWHAVSTGTLFTVYYKPRTSPAEFIVPFDQYMESVKNNYSIGMRFKMRFEGEEAPEQRFTGTIVGIEDADPGRWKNSKWRCLKVRWDETSRPER
PoptrARF10.1/1-708                               VRGRVKSEEVLEAAGLAANGNPFQVVYYPRASTPEFCVKASSVRAAMRTCWCSGMRFKMAFETEDSSRISFMGTVASVQVADPDRWPNSPWRLLQVTWDEPDTVKC
PoptrARF3.3/1-109                                -----------------------------RASSSEFTIPFNKFLKSLDQSFSSGMRFKMCFETEDAAERRYTGIITGVSELDPARWPGSKWKCLLVSW--------
PoptrARF9.2/1-670                                QSQSMHLGVLATASHAVSTLTLFVVYYKPRT--SQFIISLNKYLEAVSNKFVVGMRFKMRFEGEDSPDRRFSGTIVGVEDFSP-HWNDSKWRSLKVQWDEPARPDR
PoptrARF6.1/1-884                                QSDSMHLGLLAAAAHAAATNSRFTIFYNPRASPSEFVIPLAKYLKAVYYRVSVGMRFRMMFETEESSVRRYMGTITGISDLDVVRWPNSQWRSVKVGWDESTRQPR
PoptrARF4/1-713                                  RKQNSLPSALSLVSNAISTKSVFTVSYSPRATHAVFVVPYQKYIKSITNAVCIGTRFKMRFEMDDSPERRCSGVVTGTADLDPYKWPNSKWRCLMVRWDEDVHQER
PoptrARF6.3/1-163                                ------------------------------------------------------------------------------------------------------NPPS
PoptrARF3.2/1-714                                KNHQLNQISPGDVANAISTRSFFHIYYNPRASSSEFIIPFNKFLKSLDQSFSSGMRFKMRFETEDAAERRYTGIITGVSELDPARWPGSKWKCLLVRWDDRERLSR
PoptrARF9.3/1-579                                QSQSMHLGVLATASHAVLTQTLFVVYYKPRT--NQYIIGLNKYLEAVKNGFSVGMRFKMRFEGEDSPERRFTGTIVGVGDISP-EWSGSIWRSLKIQWDEPARPER
PoptrARF6.5/1-907                                QSDSMHIGLLAAAAHAAATNSRFTIFYNPRASPSEFVIPLVKYIKAVYHRVSVGMRFRMLFETEESSVRRYMGTITGISDLDPVRWPNSHWRSVKVGWDESTRQPR
PoptrARF9.4/1-632                                QSQSMHLGVLATASHAVLTHTLFVVYYKPRT--NQYIIGLNKYLEAVKNGFSVGMRFKMRFEGEDTPERRFTGTIVGVGDISP-EWSGSIWRSLKIQWDEPARPER
PoptrARF7.1/1-1047                               QSDSMHIGILAAAAHAAANNSPFTIFYNPSASPSEFVIPFSKYNKAMYTQGSLGMRFRMMFTTEESGVRRYMGTITGISDLDPVRWKNSQWRNLQVGWDESTRPNR
PoptrARF7.2/1-1093                               QSDSMHIGILAAAAHAAANNSPFTIFYNPRASPSEFVIPFSKYNKALYTQVSLGMRFRMMFETEESGVRRYMGTITGISDMDPVRWKNSQWRNLQVGWDESTRPSR
PoptrARF8.2/1-816                                QSDSMHIGLLAAAAHAAATNSCFTVFYNPRASPSEFVIPLSKYVKAVFHRVSVGMRFRMLFETEESSVRRYMGTITGTSDLDPVRWPNSHWRSVKVGWDESTRQPR
PoptrARF16.6/1-91                                -SEQII--------------------------GTFVLEFDVLREGLVA----------------------------------------------------------
PoptrARF16.4/1-701                               SQGKVRAESVIQAVTLAANGLPFEVVYYPRANTPEFCVKASLVKTAMQIRWCSGMRFKMAFETEDSSRISFMGTVCSVQAADSLWWPHSPWRLLQVTWDEPDNVKR
PoptrARF16.5/1-536                               SQGKVRAESVIQAVTLAANGLPFEVVYYPRANTPEFCVKASLVKTAMQIRWCSGMRFKMAFETEDSSRISFMGTVCSVQAADSLWWPHSPWRLLQVTWDEPDNVKR
PoptrARF10.2/1-713                               IRGRVKPEEVLEAAGLAANGKPFQVVYYPRSSTPEFCVKASSVRAAMRIGWCSGMRFKMAFETEDSSRISFMGTVTSVQVADPVRWPNSPWRLLQVAWDEPDNVKR
PoptrARF17.2/1-592                               NRGRLSQEAVAEAVEMAAKGLPFDVVYYPRAGWSDFVVRAEAVEAALGVFWTAGMRVKMAMETEDSSRMTFQGTVSGTGLPDSGAWRGSPWRMLQITWDEPENAKR
PoptrARF2.6/1-724                                ESHSMQLGILSSASHAITTGSMFTIYFHPWTSPAEFIIPYDQYMKSAEIDYSAGTRFRMLFEGEECAEQRFEGTVVGTEDVDHIRWPNSEWRILKVKWDAASHQER
PoptrARF2.5/1-614                                EAHSMQLGILSSASHAISTGSIFTIFFHPWTSPAEFIIPFDQYMKSAEIEYSIGTRFIMQFEGEECTEQRCEGTVVGAEDVDHIRWPNSEWRILKVKWDAASHPER
```

Consensus

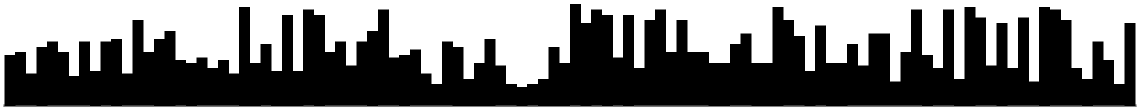

```
QSDSMH+GVLAAAAHAAATGSPFTVFYNPRASPSEFVVPLDKYLKAVYNQFSVGMRFKMRFETEDSSERRFMGTITGVSDLDPVRWPNSKWRSLKVGWDEPTRPNR
```

```
                                                  220        230        240        250        260        270        280        290
LOC_Os01g70270.1|11667.m07038_altsplice/1-809     VSPWSCTKVHKQGVALGRSVDLSKFSNYDELKAELDKMFEFQIVYTDNEGDMMLVGDDPWEEFCSIVRKIYIYTKEEVQKMNS
LOC_Os01g13520.1|11667.m01333/1-700               VSPWTRIKVQMHGNAVGRAVDLANLDGYEQLMNELEEMFNIKVAFTDDEGDTMEVGDDPWLEFCQMVRKIVLYPIEDEKKIEP
LOC_Os02g06910.1|11668.m00642/1-909               VSLWTFVKVYKSG-TVGRLLDITRFSSYHELRSEVGRLFGLQLVFVDREDDVLLVGDDPWQEFVNSVSCIKILSPQEVQQMGK
LOC_Os02g35140.1|11668.m03331/1-757               VSPWSCTKVIMQGMAVGRAVDLTRLHGYDDLRCKLEEMFDIKVVYTDDEDDMMLVGDDPWPTLTTLLQCLILLSRVNAALLIL
LOC_Os02g41800.1|11668.m03990/1-784               VSPWGQCKVFIESDTVGRNLDLSSLASFEQLYGRLSEMFCI---YRGATGEVRHAGDEPFSDFNKKAQQLRVLVDSRVLRCKN
LOC_Os04g57610.1|11670.m05695/1-819               VSLWTFVKVYKSG-SVGRSLDITRFSNYAELREELGQMFGIQLVFVDRENDVLLLGDDPWESFVNSVWYIKILSPEDVHKMGK
LOC_Os04g43910.1|11670.m04249/1-696               VCPWGQCKVFVESETVGRSLDLSALSSFEELYACLSDMFSIHLVYRSPAGEVKHAGDEPFCAFVKSARKLRILTDAGSDNLGD
LOC_Os04g49230.1|11670.m04840/1-294               -------------------------------------------RRRRHLRLLTN----STL----RCTTRAPX-------
LOC_Os04g36060.1|11670.m03490/1-1674              VSPWPSAESYTLTGSASEAWDACAVDDPDNFNPFLTHAFLSLPLVVARDENETVVGVVPLKEFVDQSWKLQSCVPFTGQRILV
LOC_Os05g43920.1|11682.m04181/1-700               VSPWTPSEITSNQFALARIPAPPSGAGLPKRDAGRSRLFGF---DGEGLDDGAIEAGNPLELFGSHSTPGALHALCAAAPLGM
LOC_Os06g09660.1|11680.m00929/1-1056              VSIWTFTKVQKRG-SVGRSIDITRYRDYDELRHDLACMFGIKLVYVDHENDILLVGDDPWEEFVGCVKSIKILSAAEVQQMSL
LOC_Os06g46410.1|11680.m04579/1-918               VSLWTFVKVYKSG-TYGRSLDITRFSSYHELRRELGRLFGLQLVFVDREDDVLLVGDDPWQEFVNSVSCIKILSPQEVQQMGK
LOC_Os06g47150.1|11680.m04656/1-731               VSPWGHCKVFMQSEDVGRTLDLSVVGSYEELYRRLADMFGI-HVYRDAAGALKHTGDEPFSEFTKTARRLNILTDTSGDNLAS
LOC_Os06g48950.1|11680.m04850/1-1116              VSIWTFTKVYKRG-AVGRSIDMSQFSGYDELKHALARMFSIKLVYKDHEDDILLLGDDPWEEFVGCVKCIRILSPQEVQQMSL
LOC_Os08g40900.1|11674.m04106_altsplice/1-1056    VSLWTYTKVHKRG-AVGRSIDINRYSGYDELKHDVARMFGIKLVYEDHEKDVLLVGDDPWEDFVKCVRCIRILSPQEEMQMRL
LOC_Os11g32110.1|11687.m02952/1-812               VSPWSCKKVHKQGIALGRSVDLTKFNGYEELIAELDDMFDFMVVYTDNEGDMMLVGDDPWI-FAPVQPDGTGYLSC---YLAT
LOC_Os12g41950.1|11686.m04180/1-900               VSLWTFVKVYKSG-SLGRSLDISRFSSYCELRSELERLFGLQLVFVDRENDILLVGDDPWQEFANSVWCIKILSPQEVQQLVR
LOC_Os12g29520.1|11686.m02861_altsplice/1-842     VSPWSCKKVHKQGIALGRSIDLTKFTCYDELIAELDQMFDFMVVYTDNEGDMMLVGDDPWNEFCNMVHKIFIYTREEVQKMNP
LOC_Os04g56850.1|11670.m05610_altsplice/1-956     VSIWTYTKVQKQG-SVGRSIDVTGFRNYHELRSAIACMFGLKLVYVDYENDVLLVGDDPWEEFINCVRCIRILSPSEVQQMSE
LOC_Os05g48870.1|11682.m04683_altsplice/1-696     VSPWTSGEVIDGQVTVEKSHSPGRHGGPDNKAVGTNKIFGI---LDDGDANYSLQSLVP--KSLNSCATVSVLX--------
LOC_Os01g48060.1|11667.m04657_altsplice/1-723     VSPWECTRNEKLHSPVSGAEHESNNKCLNTNGC---KIFGI--------GDEVDCGNAS--YHS----RLQSLKPQMPKSLGS
LOC_Os01g54990.1|11667.m05421/1-656               VSPWLLSETSWDQFKIGKASTPGNATGNGGREVDRTRLFGF---DKDGEKEVSYETDNPLDLFGNCSTPGALHALC-AAPLGI
LOC_Os10g33940.1|11676.m02996/1-699               VSPWGHCKVFMESEDVGRTIDLSVFGSYEELYGRLADMFGI-HLFRDAAGVVKHPGEVPFSDFMKAARRLTIIAGD-RERIER
LOC_Os02g04810.1|11668.m00419/1-1094              VSIWTFTKVYKRG-AVGRSIDIGRYSGYEELKHALARMFGIKLVYKDHEDDILLLGDDPWE---------------------
LOC_Os04g59430.1|11670.m05892/1-530               VNPW--------GDTNGAF-------ASAQVNQVPE--------GVDDETATEEASDLP--D------SLTNGHNQDGARLX-
LOC_Os07g08520.1|11673.m00789/1-729               VNSWTRVRLLMNPDDARRRSQPPVYGTVRDVHCRSERMLEVRVNWQVQPVQLALPPQDE--EAAATTSDATTSAPSQLQTMAS
LOC_Os07g08530.1|11673.m00790/1-408               ANFW-----IDDTASTSVSVD------------------------NGDEQVPTMRQ-----------RLEALIPDNIX----
LOC_Os07g08600.1|11673.m00797/1-525               ---W-------------------------------------ED-GKLPTIGPQG--EVI----SIENYATS----IGA
ARF1/1-665                                        VSPWSCTKVHMQGSAVGRAIDLTRSECYEDLFKKLEEMFDIQVVYTDDEDDMMMVGDDPWNEFCGMVRKIFIYTPEEVKKLSP
ARF3/1-608                                        VSPW------DHHGGSGRC----------------RLFGF---LTDETTAVASATAVP---------CVEGNSMKAVQSNHH
ARF4/1-788                                        VSPWICTKVHKQGSQVGRAIDLSRLNGYDDLLMELERLFNMRILYTDSENDMMVVGDDPWHDFCNVVWKIHLYTKEEVENAND
ARF5/1-902                                        VSPWTYTKVQKTG-SVGRSIDVTSFKDYEELKSAIECMFGLKLVYVDYESDVLLVGDDPWEEFVGCVRCIRILSPTEVQQMSE
ARF6/1-933                                        VSLWTFVKVYKSG-SFGRSLDISKFSSYHELRSELARMFGLQLVFVDRENDVLLLGDDPWPEFVSSVWCIKILSPQEVQQMGK
ARF7/1-1165                                       VSVWTYTKVQKRG-SVGRSIDVNRYRGYDELRHDLARMFGIKLVYVDHENDILLVGDDPWEEFVNCVQSIKILSSAEVQQMSL
ARF8/1-811                                        VSLWNFVKVYKSG-SVGRSLDISRFSSYHELREELGKMFAIQLVFVDKENDILLLGDDPWESFVNNVWYIKILSPEDVHQMGD
ARF9/1-638                                        VSPWSRTKVQMQGVPVGRAVDLNALKGYNELIDDIEKLFDIEIVFTDDEGDMMLVGDDPWPEFCNMVKRIFIWSKEEVKKMTP
ARF10/1-693                                       VSPWGHCKVFMESEDVGRTLDLSVIGSYQELYRKLAEMFHITHVYRDANGVIKRIGDEPFSDFMKATKRLTIKMDIGGDNVRK
ARF11/1-601                                       VSPWSRIKVQMQGTAVGRAVDLTLLRSYDELIKELEKMFEIAIVFTDDEGDRMLVGDDPWNEFCKMAKKLFIYPSDEVKKMRS
ARF12/1-593                                       VSPWTCTKVQMQGVTIGRAVDLSVLNGYDQLILELEKLFDIEIAFTDSDEDKMLVGDDPWPEFCNMVKKIFIQKRR-------
ARF13/1-623                                       VSPWSRIKVHMQGVAISRAVDLTAMHGYNQLIQKLEELFDLEIVFTNNEGAEMLVGDDPWPEFCNMAKRIFICSKEEIKKMKL
ARF14/1-605                                       VSPWTCTKVQMQGVTIGRAVDLSVLNGYDQLILELEKLFDLEIAFTNNEEDKMLVGEDPWPEFCNMVKKIFIYSKEEVKNLKS
ARF15/1-598                                       VSPWTCTKVQMQGVTIGRAVDLSVLNGYDQLILELEKLFDLKIIFTGSDEDEMLVGDDPWPEFCNMVKRIYIQKRR-------
ARF16/1-670                                       VNPWGHCKVFMESDDVGRTLDLSVLGSYEELSRKLSDMFGI-SVYRDASGAIKYAGNEPFSEFLKTARRLTILTEQGSESVVV
ARF17/1-585                                       VNPWKVNSI----QLFGKIITVEEHSESGPAESGLC---EE-----DNETQLSLSHAPP---------SVPKHSNSNAGSSSQ
ARF18/1-602                                       VSPWSRTKVQMQGIAVGRAVDLTLLKSYDELIDELEEMFEIIVVFTDDEGDMMLAGDDPWNEFCKMAKKIFIYSSDEVKKMTT
ARF19/1-1086                                      VSIWTYTKVQKRG-SVGRSIDVTRYSGYDELRHDLARMFGIKLVYTDHENDILLVGDDPWEEFVNCVQNIKILSSVEVQQMSL
ARF20/1-615                                       VSPWTCTKVQMQGVTIGRAVDLSVLNGYDQLILELEKLFDLKIAFTDSDGYEMLVGDDPWPEFCKMVKKILIYSKEEVKNLKS
ARF21/1-606                                       VSPWTCTKVQMQGVTIGRAVDLSVLNGYDQLILELEKLFDIKIAFTDSDGYEMLVGDDPWPEFCKMVKKILIYSKEEVKNLKS
ARF22/1-600                                       VSPWTCTKVQMQGVTIERAVDLSVLNGYDQLILELEELFDLEIAFTDSDDDKMLVGDDPWPEFCNMVKKILIFKRG-GQKLEV
ARF23/1-222                                       -----------------------------------------------------------------------------------
ARF2/1-859                                        VSPWSCTKVHKQGIALGRSVDLSKFQNYEELVAELDRLFEFLIVYTDEENDMMLVGDDPWQEFCCMVRKIFIYTKEEVRKMNP
PoptrARF8.1/1-827                                 VSLWTFVKVYKSG-SVGRSLDISRFSSYHELRGELAQMFGIQLVFVDRENDVLLLGDDPWELFVNNVWYIKILSPEDVLKLGE
PoptrARF1.1/1-660                                 VSPWSCTKVHMQGVAVGRAVDLTQFERYEDLLRKLEEMFDIQVVYTDNEDDMMKVGDDPWHEFCSMVKKIFIYASEEVKRLSP
PoptrARF7.3/1-1113                                VSIWTYTKVYKRG-AVGRSIDITRYSGYDELKQDLARRFGIKLVYTDHENDVLLVGDDPWEEFVNCVRCIKILSPQEVQQMSL
PoptrARF2.3/1-792                                 VSPWRCVKVYKQGTAVGRSLDLAKFNGYNELTAELDQIFEFLIVFTDDEGDMMLVGDDPWQEFCSMVRRIFVFTREEINRMEP
PoptrARF5.1/1-933                                 VSSWTYTKVQKTG-SVGRSIDVSSFKNYEELCSAIECMFGLKLVYVDYENDVLLIGDDPWEEFVGCVRCIRILSPSEVQQMSE
PoptrARF5.2/1-944                                 VSSWTYTKVQKTG-SVGRSIDVSSFKNYEELCSAIECMFGLKLVYVDYENDVLLIGDDPWEEFVGCVRCIRILSPSEVQQMSE
PoptrARF7.4/1-1137                                VSIWTYTKVYKRG-AVGRSIDIARYSGYAELKQDLARRFGIKLVYRDLDDDVLLVGDDPWEEFVNCVRCIKILSPQEVQQMSL
PoptrARF16.1/1-669                                VSPWGHCKVFMESEDVGRTLDLSVLGSYEELHRKLVNMFGI-NVYRNAAGATKHAGDEPFSEFLKTARRLTILSDASSDNVGR
PoptrARF1.2/1-662                                 VSHWSCTKVHMQGVAVGRAVDLTQFKRYEDLLRKLEEMFDIQVVYTDNEDDMMKVGDDPWNEFCGMVKKIFIYTSEEVKRLSP
PoptrARF9.1/1-666                                 VSPWSRTKVQLQGIAVGRAVDLTLIKGYGQLIDELEQLFDIEIVYTDDEGDMMLVGDDPWPEFCNMVRRIFICSSQDVKKMSP
PoptrARF2.1/1-854                                 VSPWSCTKVHKQGIALGRSVDLTRFNNYDELIAELDRLFEFLIVYTDDEDDMMLVGDDPWQEFVGMVRKIVIYTREEVQRIKP
PoptrARF16.3/1-700                                VSPWGHCKVFLESEDVGRTLDLQLLESYEELYRKLADMFGLNLLYRDDNGITKHIGEEPFSNFSKTARRLTIVTRF------R
PoptrARF3.1/1-709                                 VSPWVLSHEHLQHGISQPVVAQSAFRSSQDMVLCKSRLFGF---VVNKEDNIASITSNPESSFLRVGEQLHPKPPAINNAVGS
PoptrARF6.2/1-914                                 VSLWTFVKVYKSG-SFGRSLDITKFSNYNELRSELAFMFGLQLVFIDRENDVLLLGDGPWPEFVNSVWCIKILSPQEVQQMGK
PoptrARF6.4/1-953                                 VSLWTFVKVHKSG-SYGRSLDISKFSSYDELRSELARLFCLQLVFVDRENDVLLLGDDPWQEFVNNVWYIKILSPLEVQQMGK
PoptrARF17.1/1-594                                VSPW-----QSSAHSFGK--DFTGNRSFNPKKVGISQLFGKDSVFMDNSSKGCNETENA--LEL----SLTSSYTELLNRIDV
PoptrARF2.4/1-879                                 VSPWRCVKVHKQGTAVGRSLDLSKFNGYNELTAELDQIFEFLIVFTDDEDDMMLVGDDPWQEFCSMVRRIFIYTKEEINRMEP
PoptrARF16.2/1-708                                VSPWDHCKVFLESEDIGRTLDLSVLGSYEELHRKLASMFGI-NVYRDAAGATKHAGDEPFSEFLKTARRLTILSYASRDNFGR
PoptrARF2.2/1-852                                 VSPWSCTKVHKQGIALGRSVDLAKFNNYDELIAELDRLFEFLIVYTDDEDDMMLVGDDPWQEFVGMVRKIVIYTKEEAQKIKP
PoptrARF10.1/1-708                                VSPWGHCKAFLESEDLGRTLDLSALHSYEELRRKLAIMFGISHVYRDVTGAVKQIGDEPFSVFMKTAKRLTILMNRSGNSVGR
PoptrARF3.3/1-109                                 ---------------------------------------YVVVENHIL--------------------------------
PoptrARF9.2/1-670                                 VSPWSRTKVQMQGIAVGRAVDLTMLKGYSQLIDELEQLFDIEIVYTDDEGDMMLVGDDPWPEFCNMVRRIYICSSQDVKRMGP
PoptrARF6.1/1-884                                 VSLWTFVKVYKSG-SFGRSLDITKFSSYNELRSELSRMFGLQLVFIDRENDVLLLGDGPWPEFVNSVWYIKILSPQEVQQMGK
PoptrARF4/1-713                                   VSPWSCTKVHKQGSLVGRAIDLSRLNGYSDLLNELERLFSMRILYTDSENDVMVVGDDPWLEFCNVATKIHIYTQEEVEKMTL
PoptrARF6.3/1-163                                 ----TFVKVYKSG-SFGRSLDITKFSNYNELRSELAFMFGLQLVFIDRENDVLLLGDGPWPEFVNSVWCIKILSPQEVQQMGK
PoptrARF3.2/1-714                                 VSPWGLSHEHLQHGVSQPIVAQSAFRGSQDLVSCKSRLFGF---LVNKEDNMTLITSNPGSSFLRAGEHFHQSLQQ-------
PoptrARF9.3/1-579                                 VSPWSEAMVQMQGIAVGRALDLTVLKGYRDLIYELEKMFEIAVVFTDDEGDMMLVGDDPWPEFCKMVKKIFIYSSEEVKKTGT
PoptrARF6.5/1-907                                 VSLWTFVKVHKSG-SYGRSLDISKFSSYDELRSELARLFCLQLVFGDRENDVLLLGDDPWQEFVNNVWYIKILSPLEVQQMGK
PoptrARF9.4/1-632                                 VSPWTRTKVQMQGVAVGRALDLTVLKGYKDLINELEKMFETAVVFTDNEGDMMLVGDDPWPEFCKMVKKIFIYSSEEVKKMST
PoptrARF7.1/1-1047                                VSIWTYTKVQKRG-SVGRSIDITCYKGYDELRHDLARMFGIKLVYVDRENDILLVGDDPWEEFMSCVQSIKILSSAEVQQMSL
PoptrARF7.2/1-1093                                VSIWTYTKVQKRG-SVGRSIDVTRYKGYNELRHDLARMFGIKLVYVDHENDILLVGDDPWEEFVSCVQSIKILSSAEVQQMSL
PoptrARF8.2/1-816                                 VSLWTFVKVYKSG-SVGRSLDISRFSSYHELREELAQMFGIQLVFVDRENDVLLLGDDPWELFVNNVWYIKILSPEDVLKMGE
PoptrARF16.6/1-91                                 -----------------------------------------------------------------------------------
PoptrARF16.4/1-701                                VSPWGHCKVFMDSEDVGRTLDLSLLGSYEELYRKLANMFGL---YRDINGITKHIGEEPFSDFFKTARRLTIVTDSSSGNVGI
PoptrARF16.5/1-536                                VSPWGHCKVFMDSEDVGRTLDLSLLGSYEELYRKLANMFGL------------------------------------------
PoptrARF10.2/1-713                                VSPWGPCKVFLESEDVGWTLDLSALCSYEELHGKLANMFGI-HVYRDATGSVKQIGDEPFSVFMKTAKRLTILMNQEIDDLSC
PoptrARF17.2/1-592                                VSPW-------QSSAYSFGIGFVGNRGFNPKKVGINQLFGKDDVFMDDSSKCCNETEDALELSLSSYTELLNRIDAQCQRASP
PoptrARF2.6/1-724                                 VSPWSCTKVLKYGTVPGRSVDLTQFDGYNELICELDLMFDFYVVYSDNEGDMMQIKDCPWQEFQLTVRRIFISPKEDIGKLNP
PoptrARF2.5/1-614                                 VSPWSCTKVLKYGTVLGRSVDLTQFDGYSELICELDLMFDFCVAYSDNEGDMIQIADCPWQEFLSAVHRIFICPKEETGKLNP
```

Consensus

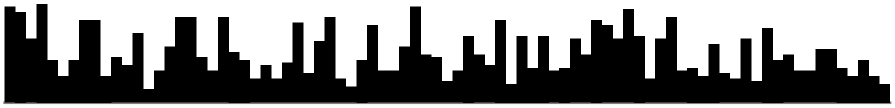

```
VSPWTCTKVQKQGVAVGRSLDLSRFSGYDELRSEL+RMFGIKLVYTDDEGDVMLVGDDPWEEFVNMVR+IKILSPEEVQQMGP
```
